# Supplementary material for: Artificial Selection on Microbiomes To Breed Microbiomes That Confer Salt Tolerance to Plants
Source: mSystems. 2021 Nov 30;6(6):e01125-21. doi: 10.1128/mSystems.01125-21 (PMC8631316; doi:10.1128/mSystems.01125-21)
Supplement: TEXT S3 [file msystems.01125-21-t0003.pdf]

SUPPLEMENTAL MATERIAL: RESULTS

Generations 1-8: Effects of differential microbiome propagation under sodium-sulfate (SOD) stress: We found a significant main effect of treatment on plant biomass over 8 generations of microbiome selection under sodium-sulfate stress (LRT: Treatment, Chisq=27.8, p<0.001; Generation, Chisq=381.8, p<0.001; Treatment x Generation, Chisq=15.2, p=0.37; Figure 2 left). Plant biomass was 75% higher in the plant-present Microbiome-Selection lines (beta=0.57 ± 0.06, z=10.0, p<0.001) than in the Fallow-Soil Control lines, and 66% higher than in the Null-Control line (beta=0.50 ± 0.07, z=7.4, p<0.001). There was no significant difference in biomass between the fallow-soil and the null-control treatments (beta=0.07 ± 0.06, z=1.1, p=0.29). The lack of a significant interaction between treatment and generation (Chisq=15.2, p=0.37) indicates that gains in plant biomass were realized quickly in the first few selection cycles, and that the advantage of the plant-present Microbiome-Selection treatment over the Fallow-Soil treatment was maintained as the concentration of sodium-sulfate was ramped up over the course of the experiment.

Generation 9, SOD-treatments: We measured total seed weight in the final Generation 9 of the experiment and found significant difference among treatments (Kruskal-Wallis Chisq=10.6, p=0.01; Figure 2 right). Total seed weight in the plant-present Microbiome-Selection lines were 168% greater compared to the null-control line, 120% greater than the Fallow-Soil-Control lines, and 205% greater than plants grown in soil that was inoculated with filtrate (0.2µm filter) from the soil of plant-present Microbiome-Selection lines (Figure 2 right; Table S5).

Table S5. Mann-Whitney pairwise comparisons of total seed weight in the sodium-sulfate (SOD) treatments. Values represent the test statistics (p-value in parentheses) for each comparison. Significant comparisons are indicated in bold. Np = Fallow-Soil microbiome-propagation control, Null=Null-Control line, Pp=Plant-present Microbiome-Selection line, PpFilt=Plant-present Microbiome-Selection line filtrate.

	Np	Null	Pp
Null	100 (0.50)		
Pp	**0 (0.02)**	**20 (0.02)**	
PpFilt	20 (0.50)	90 (0.71)	**20 (0.02)**

Generations 1-8: Effects of microbiome propagation under aluminum-sulfate (ALU) stress: Unlike the sodium-sulfate experiment, we found a significant interaction between treatment and generation under aluminum-sulfate stress (LRT: Treatment, Chisq=25.7, p<0.001; Generation, Chisq=753.7, p<0.001; Treatment x Generation, Chisq=26.6, p=0.02). The interaction was due to a drop in plant biomass in the Fallow-Soil treatment in Generations 4 and 5 (Figure 2). To calculate a conservative estimate of the effect size of our treatments on plant biomass, we re-ran the analysis excluding Generations 4 and 5, which eliminated the significant interaction between treatment and generation (LRT: Treatment, Chisq=17.8, p<0.001; Generation, Chisq=614.5, p<0.001, Treatment x Generation, Chisq=7.67, p=0.66). In the reduced dataset, we found that plant biomass in plant-present Microbiome-Selection lines were 38% larger than in fallow-soil lines (beta=0.32 ± 0.04, z=8.9, p<0.001), but not significantly different from the Null-Control line (beta=0.09 ± 0.4, z=2.3, p=0.06). Null-Control plants generated 26% greater biomass than Fallow-Soil-Control plants (beta=0.23 ± 0.04, z=5.1, p<0.001).

Generation 9, ALU-treatments: As in the sodium sulfate experiment, total seed weight in the final Generation 9 was significantly different among treatments (Kruskal-Wallis: Chisq=9, p=0.02; Figure 2 right). Total seeds weight in the plant-present Microbiome-Selection lines were 194% greater than in the fallow-soil lines, 101% greater than in the Null-Control line, and 55.4% greater than in the filtrate lines (Table S6). Plants with filtrate-inoculated soil produced total seed weights that were 89.2% greater than plants grown in the Fallow-Soil Control (Figure 2 right; Table S6).

Table S6. Mann-Whitney pairwise comparisons of total seed weight in the aluminum-sulfate (ALU) treatments. Values represent the test statistics (p-value in parentheses) for each comparison. Significant comparisons are indicated in bold. Np = Fallow-Soil microbiome-propagation control, Null=Null-Control line, Pp=Plant-present Microbiome-Selection line, PpFilt=Plant-present Microbiome-Selection line filtrate.

	Np	Null	Pp
Null	80 (0.55)		
Pp	**0 (0.02)**	**30 (0.03)**	
PpFilt	**0 (0.02)**	70 (0.29)	**20 (0.05)**

Interactions between selection history and salt stress on plant fitness: By growing plants with microbiomes from selection lines under both sodium- and aluminum-sulfate stress (*Cross-Fostering Control*), we examined whether microbiome selection produced microbiomes that conferred a salt-specific effect on plants (e.g., whether microbiomes selected to confer tolerance to SOD conferred such tolerance only under SOD stress, but not under ALU stress), or alternatively whether selected microbiomes produced a generalized improvement in plant fitness under both SOD and ALU stresses. There was a significant interaction between selection history and the type of salt stress to which plants were exposed in the last generation on seed mass (Analysis of deviance: Selection history, $F_{1,8}$=<0.01, p=0.99; Salt exposure, $F_{1,141}$=5.82, p=0.017; Selection history x Salt exposure, $F_{1,141}$=6.42, p=0.012; Figure 2 right), indicating that performance under SOD-stress or ALU-stress in Generation 9 depended upon which salt the microbiome was selected on during Generations 0-8.

We conducted post-hoc comparisons of the treatment means and found that plants grown with microbiomes selected under sodium-sulfate stress had total seed weights that were 70.1% greater when exposed to sodium-sulfate stress compared to exposure of aluminum-sulfate stress in Generation 9 (beta=108 ± 31.0, z=3.5, p=0.002). In contrast, plants grown in microbiomes selected under aluminum-sulfate stress did not differ in total seed weight, regardless of whether they were stressed with sodium- or aluminum-sulfate in Generation 9 (beta=4.2 ± 31.8, z=0.13, p=0.99). The effect of exposure to different kinds of salt stress on plant fitness thus depends upon the selection history of the soil microbiome.

Unlike total seed weight, there was no interaction between selection history and the type of salt stress on total plant biomass, however there was a trend toward plants growing larger under ALU-stress compared to SOD-stress irrespective of the selection history (Analysis of deviance: Selection history, F=0.14, p=0.72; Salt exposure, F=3.71, p=0.056; Selection history x Salt exposure, F=1.38, p=0.24).
