# Supplementary material for: Artificial Selection on Microbiomes To Breed Microbiomes That Confer Salt Tolerance to Plants
Source: mSystems. 2021 Nov 30;6(6):e01125-21. doi: 10.1128/mSystems.01125-21 (PMC8631316; doi:10.1128/mSystems.01125-21)
Supplement: TEXT S1 [file msystems.01125-21-t0001.pdf]

SUPPLEMENTAL MATERIAL: METHODS

Protocol Outline: We used a differential host-microbiome co-propagation scheme as described in Swenson *et al* (2000) and in Mueller *et al* (2005) (Figure S1), but we added to this scheme steps to enhance microbiome transmission and thus response to selection, including (a) microbiome-fractionation using size-selecting filters (Bakken & Olsen 1987; Mueller & Sachs 2015); (b) ramping of stress in successive selection cycles (Garland & Rose 2009); (c) facilitation of priority effects during microbiome assembly (Fierer *et al* 2012; Scheuring & Yu 2012) by capping pots for the first 4 days of the germination stage (i.e., we used a so-called *semi-open system*; Mueller & Sachs 2015), thus controlling in each selection cycle the initial recruitment of symbiotic bacteria into rhizosphere microbiomes of seedlings; and (d) low-carbon soil to enhance carbon-dependent host-control of microbiome assembly and persistence (Bais *et al* 2006; Bulgarelli *et al* 2013; Mueller & Sachs 2015; Coyte *et al* 2015). In each microbiome-propagation cycle ('Microbiome Generation' = Gen), we inoculated surface-sterilized seeds taken from non-evolving stock (inbred strain Bd3-1 of the grass *Brachypodium distachyon*, derived via single-seed-descent inbreeding from the source accession; Vogel *et al* 2006; Garvin *et al* 2008; Vogel & Bragg 2009; Brkljacic *et al* 2011). We chose to conduct the experiment with *B. distachyon* because it is a model for biofuel and cereal crops, including research on salt stresses and water-use efficiency (Des Marais & Juenger 2016; Des Marais *et al* 2016).

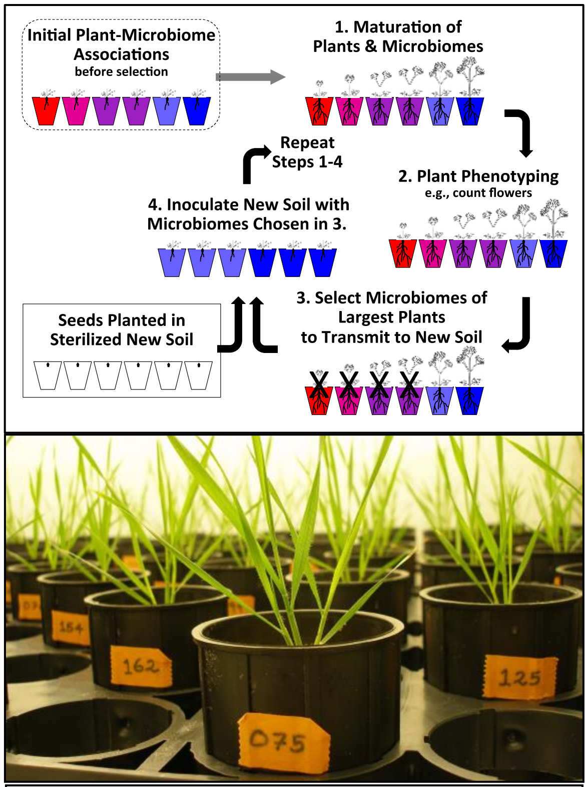

Figure S1. Top: Differential microbiome-propagation scheme to impose artificial indirect selection on rhizosphere microbiomes (figure modified from Mueller & Sachs 2015). The host-plant does not evolve because this scheme propagates only microbiomes into sterilized soil (Step 4), whereas the host is taken each cycle from a non-evolving source (stored seeds). Both evolutionary and ecological processes alter microbiomes during each cycle, but at Steps 3&4 in each cycle, experimental protocols aim to maximize evolutionary changes stemming from differential propagation of microbiomes. **Bottom:** *Brachypodium distachyon* Bd3-1 plants in our growth chamber shortly before microbiome-harvest for differential microbiome-propagation to pots/seeds of the next microbiome-generation. Photo by UGM.

We inoculated seeds with rhizosphere bacteria harvested from roots of those plants of the previous selection cycle that exhibited the greatest above-ground biomass (Figure S1). Because the plant-host could not evolve between selection-cycles (seeds were taken from non-evolving stock), whereas microbiomes could potentially evolve due to differential microbiome propagation, our selection-scheme was *one-sided selection* (Mueller & Sachs 2015). Both evolutionary and ecological processes alter microbiomes during and between selection-cycles, but our protocol aimed to maximize evolutionary changes stemming from differential microbiome-propagation at Steps 3 & 4 (Figure S1). To focus indirect selection on bacterial communities, we filtered the microbiomes harvested from rhizospheres, perpetuating only bacteria (and possibly also viruses) to the next generation, but eliminating from propagation between microbiome-generations any larger-celled soil-organisms with filters (i.e., we excluded fungi, protozoa, algae, mites, nematodes, etc. from between-plant transfers). This fractionation step distinguishes our methods from those of Swenson *et al* (2000) and from a replication of that study by Panke-Buisse *et al* (2015), both of which used differential 'whole-community' propagation to transfer between generations all organism living in soil, including the larger-celled fungi, protozoa, algae, mites, and nematodes that were excluded through size-selecting filtering in our experiment. Our complete experiment involved one baseline Generation (Generation 0, Table S1) to establish initial microbiomes in replicate pots; eight rounds of microbiome selection (i.e.,

differential microbiome-propagation) (Generations 1-8, Table S1); and one final ninth round of selection (Generation 9, Table S2) to evaluate the effects of the engineered (i.e., evolved) microbiomes on flower-production and seed-set, for a total of 10 Generations. Our entire selection experiment lasted 300 days from 3. January -29. October 2015.

Logic of Salt-Stress Ramping: We used ramping of salt-stress (Mueller & Sachs 2015) to ensure that (a) plants were neither under-stressed nor excessively over-stressed during any selection-cycle of our microbiome-selection experiment, and thus (b) facilitate that microbiomes can gradually improve under differential microbiome-propagation to confer increasingly greater salt-tolerance to plants under increasingly greater salt-stress. The experimental rationale of stress-ramping is as follows: if salt-stress is too weak, plants grow well, any salt-stress-mediating microbiomes will make little or no difference to plants, and no microbiome-mediated variation in plant-phenotype may emerge that could be used as direct target for indirect selection on microbiomes; in contrast, if salt-stress is excessive, plants suffer severely, and any observed variation in plant-phenotype may be due to microbiome-unrelated effects emerging under excessive stress, such that possible beneficial effects of salt-stress-mediating microbiomes are dwarfed and masked by the excessive stress. Stress-ramping is therefore an experimental trick that permits an experimenter to continuously adjust stress during a selection experiment, particularly in experimental evolution where the evolving effect sizes cannot be known *a priori* (i.e., in our experiment, it was not possible to predict *a priori* the approximate effect sizes attributable to beneficial microbiomes that could emerge as a result of multiple rounds of differential microbiome propagation).

Table S3 lists the ramped salt-concentrations for the two salt treatments of soils in our experiment, Na_2SO_4 (sodium-sulfate, henceforth *SOD-soil treatment*) and $Al_2(SO_4)_3$ (aluminum-sulfate, *ALU-soil treatment*). We chose the particular two salt stresses because sodium-cations are a problem in saline and sodic soils (e.g., Lodeyro & Carrillo 2015), and aluminum-cations are a problem because aluminum inhibits, at even minimum concentrations, plant growth in low-pH soils (Delhazie *et al* 1995; Aggarwal *et al* 2015). Our maximum sodium-salt stress of 75 mMolar salt-concentration sodium-sulfate of water used to hydrate soil and water plants during the experiment is not quite comparable to the salt stress of 500 mMolar sodium-chloride used by Priest *et al* (2014) because (a) the two experiments used different kinds of salts and (b) Priest *et al* spiked salt stress after initial growth of unstressed plants, whereas in our experiment the plants were salt-stressed already at the germination stage and at all times during each selection cycle.

Table S3. Salt concentrations (Millimolar = mMolar) of salt-nutrient solutions used to hydrate soil for each selection cycle (= Microbiome-Generation = Gen); the recipes to mix these solutions; and growth parameters for each Generation. In the short-cycled Generations 0-8, time was too short for plants to flower, and we quantified plant-performance by visually estimating above-ground biomass (see *Phenotyping of Plants*). In Generation 9, plants were grown for 68 days to produce seeds, and we quantified plant-performance as total seed weight per plant.

	**Microbiome-Generation (Selection Cycle)**									
	**Gen 0**	**Gen 1**	**Gen 2**	**Gen 3**	**Gen 4**	**Gen 5**	**Gen 6**	**Gen 7**	**Gen 8**	**Gen 9**
**Sodium-Sulfate Concentration**	**20 mMolar**	**30 mMolar**	**50 mMolar**	**60 mMolar**	**70 mMolar**	**75 mMolar**	**60 mMolar**	**60 mMolar**	**60 mMolar**	**60 mMolar**
**1-molar sodium-sulfate**	240 mL	360 mL	600 mL	720 mL	840 mL	900 mL	720 mL	720 mL	720 mL	1200 mL
**Dyna-Gro fertilizer**	240 mL	240 mL	240 mL	240 mL	240 mL	240 mL	240 mL	240 mL	240 mL	400 mL
**e-pure water**	12 L	12 L	12 L	12 L	12 L	12 L	12 L	12 L	12 L	20 L
**number of pots (plants)**	100	100	100	100	100	100	100	100	100	200
**Aluminum-Sulfate Concentration**	**0.02 mMolar**	**0.04 mMolar**	**0.08 mMolar**	**0.20 mMolar**	**1.0 mMolar**	**2.0 mMolar**	**1.0 mMolar**	**1.0 mMolar**	**1.5 mMolar**	**1.5 mMolar**
**1-molar aluminum-sulfate**	240 µL	480 µL	960 µL	2.4 mL	12 mL	24 mL	12 mL	12 mL	18 mL	30 mL
**Dyna-Gro fertilizer**	240 mL	240 mL	240 mL	240 mL	240 mL	240 mL	240 mL	240 mL	240 mL	400 mL
**e-pure water**	12 L	12 L	12 L	12 L	12 L	12 L	12 L	12 L	12 L	20 L
**number of pots (plants)**	100	100	100	100	100	100	100	100	100	200

**Start date (= microbiome transfer/inoculation date)**	03. Jan 2015	25. Jan 2015	14. Feb 2015	07. Mar 2015	31. Mar 2015	25. Apr 2015	27. May 2015	22. Jun 2015	20. Jul 2015	20. Aug 2015
**Number of days plants allowed to grow until microbiome harvest & transfer to next generation**	22	20	21	24	25	32	26	28	31	68
**Number of leaves of well-growing plants at day of microbiome harvest & transfer**	9-11	9-11	9-11	10-13	8-10	11-13	11-14	17-22	25-30	plants allowed to grow to seed
**Number of plants**	200	200	200	200	200	200	200	200	200	400
**Weight bin of seed weights used for planting**	5.8-5.9 mg	5.7-5.8 mg	5.5-5.6 mg	5.4-5.5 mg	5.3-5.4 mg	5.2-5.3 mg	5.1 mg	5.0 mg	4.9 mg	4.6-4.8 mg

A second pre-planned feature of our experimental design was to use ‘short-cycling’ in the initial selection-cycles (cycling at about 20-day intervals; plants grew to about the 9-13 leaf stage to grow sufficiently large root systems for microbiome harvest, but plants did not have sufficient time to flower), and then to increase lengths of selection-cycles gradually as plants became more stressed under the ramped salt-concentrations and plants needed more time to grow to the 9-13 leaf stage. Although we planned lengthening the duration of selection-cycles during our multi-generation experiment, we did not pre-plan at the beginning of our experiment the exact length of each selection-cycle, because the exact transfer dates were dependent also on time-constraints of the main experimenter (UGM) performing the microbiome-transfers. Because we increased salt-stress during the 10-Generation experiment (Table S3), plant growth was expectedly slower in later generations.

Preparation of *Brachypodium distachyon* Seeds: Prior to the start of the microbiome-selection experiment, we harvested about 6000 seeds from 36 plants (*B. distachyon* strain Bd3-1; Garvin *et al* 2008; Vogel & Bragg 2009) grown simultaneously at room temperature under constant light-cycle (14h light, 10h dark) in well-homogenized, well-watered and well-fertilized greenhouse potting soil. Seeds were air-dried at room temperature for 4 months, mixed well, then weighed individually to the nearest 0.1mg to generate groups of seeds of equal weight (binned to within 0.1mg). To reduce within-generation phenotypic variation due to differences in seed-weight-dependent maternal effects, we used seeds of only one or two adjacent weight-bins for each generation (see last row in Table S3). We used seeds of 5.9&5.8mg weight for the initial baseline Generation 0, then we used up seeds of bins of gradually decreasing seed-weight (5.9&5.8mg, 5.8&5.7mg, 5.6&5.5mg, …), as shown in the last row of Table S3 for each microbiome-generation. All microbiome selection-cycles used seeds from this stored (non-evolving) seed-stock of Bd3-1 plants, and microbiomes were therefore selected under a so-called *one-sided selection* scheme (Mueller & Sachs 2015) in the single plant-genotype background Bd3-1, such that only microbiomes can change between selection-cycles but the plant host cannot evolve.

Growth Chamber: For the multi-generation selection experiment, we grew plants under constant temperature (24°C) and constant light-cycle (20h light 4AM-midnight, 4h dark) in a walk-in growth chamber (model MTPS72; Conviron, Winnipeg, Canada) at the Welch Greenhouse Facility of the University of Texas at Austin. The chamber was not humidity-controlled, and chamber humidity therefore varied with outdoor humidity/rainfall and with any heating (in winter) affecting humidity of the air circulating in the Greenhouse Facility. Because of unusual rainfall in spring 2015, humidity was highest in the growth chamber during Generations 4 & 5, and lowest during selection-cycles 0-2 and 7-9. Unfortunately, we did not monitor exact humidity with a hygrometer in the chamber, but we recorded in a journal any days of high humidity. We grew plants on two shelves (each 120cm x 100cm) in the Conviron chamber, under fluorescent lights (Sylvania T8 fluorescent tubes spaced at 10cm, plus a center row of T2 fluorescent spiral-bulbs) generating a light-intensity of 192 µmol/m^2/s at soil level. Except for preparation of pots and planting of seeds, we performed all experimental steps for artificial microbiome selection in this chamber, including microbiome-harvesting from rhizospheres, microbiome-fractionation (filtering), and microbiome-transfers to surface-sterilized seeds planted in sterile soil (details below).

Soil & Pot Preparation: We grew plants from surface-sterilized seeds, each planted individually in the center of its own D50-Deepot (5cm pot diameter, 17.8cm depth, total volume 262ml; model D16H; Stewe & Sons, Tangent, Oregon, USA) filled with autoclaved PPC soil (Profile Porous Ceramic soil, Greens-Grade^{TM} Emerald, Natural Color; PROFILE Products LLC, Buffalo Grove, IL, USA). To permit autoclaving of soil in the Deepots prior to planting, we pressed heat-tolerant fiberglass-fill into the bottom of each pot to plug bottom-drainage holes, then compacted dry PPC soil into each pot until the soil level reached 15mm below the pot margin. Each plug consisted of a fiberglass square (9.5cm x 9.5cm) cut from an insulation-sheet (R-13 EcoTouch Insulation Roll; 38cm width; GreenGuard-certified, formaldehyde-free), then pressed firmly into the bottom of a pot. After compacting soil in all pots used for a given selection-cycle (200 pots in Generations 0-8; 400 pots in the final Generation 9) we carefully equalized soil levels between all pots.

According to the manufacturer's website (www.profileevs.com/products/soil-amendments/profile-porous-ceramic-ppc), PPC soil is a calcined, non-swelling illite, non-crystalline opal mineral; it has 74% pore space, with 39% capillary (water) pores and 35% non-capillary (air) pores; pH = 5.5; cation-exchange-capacity of 33.6 mEq/100g; and a chemical composition of 74% SiO_2, 11% Al_2O_3, 5% Fe_2O_3, and less than 5% of the remainder combining all other chemicals (e.g., CaO, MgO, K_2O, Na_2O, TiO_2). We chose PPC soil for three reasons: First, PPC has a very homogeneous consistency because of its uniform particle size; soil-quantity and soil-quality are therefore easy to standardize between pots. Second, whole root systems can be easily extracted from hydrated soil with little rupture of roots. Third, because the manufacturer exposes PPC soil to high temperature (heated in a rotary kiln at 1200 degrees Fahrenheit, then de-dusted), the soil contains minimum carbon, and we believed that such low- or no-carbon soil could facilitate a plant's ability for carbon-mediated host-control (via carbon exudates by roots; see above *Protocol Outline*) (Bais *et al* 2006; Bulgarelli *et al* 2013; Mueller & Sachs 2015; Coyte *et al* 2015) of microbiome-assembly and microbiome-stability.

Soil Hydration & Salt-Stress Treatments: After compacting soil into each pot with a wooden dowel and equalizing soil levels between all pots used in a selection-cycle, we hydrated each pot with 94ml of a fertilizer-salt solution (recipes for solutions are listed in Table S3, and are described also below). The fertilizer concentrations in this solution was identical in each selection-cycle (i.e., we added the same amount of fertilizer to soil of each microbiome generation), but we increased salt-concentrations gradually between successive selection-cycles in order to ramp salt-stress, as shown in Table S3 for the two salt-stress treatments, Na_2S0_4 (decahydrate sodium-sulfate, MW=322.2g; *SOD-soil*) and $Al_2(S0_4)_3$ (anhydrous aluminum-sulfate, MW=342.15; *ALU-soil*). We chose the particular two salt stresses because sodium is a problem in saline soils (e.g., Lodeyro & Carrillo 2015), and aluminum is a problem because it inhibits, at even minimum concentrations, plant growth in low-pH soils (Delhazie *et al* 1995; Aggarwal *et al* 2015). Because of this pH-dependent growth-attenuating effect of aluminum in soil, we suspected that it may be easier to select for a microbiome conferring tolerance to aluminum salt, for example by selecting for a microbiome that increases soil pH (i.e., artificial microbiome selection could perhaps select against acidifying bacteria in microbiomes). We therefore were able to formulate this *a priori* hypothesis on a possible pH-based mechanistic basis of a microbiome-conferred tolerance to aluminum-salt. In contrast, we did not formulate a similarly specific mechanistic hypothesis for why a microbiome could confer tolerance to sodium-salt, although a number of hypotheses have been suggested in the literature, such as changes in phytohormone concentrations influencing plant physiology, or indirect physiological effects on transpiration rates (Dodd & Pérez-Alfocea 2012). We selected for microbiomes conferring sodium-salt-tolerance and in parallel for microbiomes conferring aluminum-salt-tolerance because such a dual experimental design of two soil-treatments in the same experiment offered two advantages: (i) we could contrast evolving microbiomes between aluminum- versus sodium-treatments to identify candidate bacterial taxa or candidate consortia that may be important in mediating microbiome-conferred salt-tolerance to plants; and (ii) we could cross selection history with selection stress in the last Generation 9 to test for possible specificities of evolved microbiomes, as explained further below in *Crossing Evolved SOD- and ALU-Microbiomes with SOD- and ALU-Stress*.

The salt-concentration of the baseline Generation 0 (Table S3) was determined in a salt-gradient pilot experiment as that salt-concentration that caused a minimal, but just noticeable, delay in germination and a minimal growth-rate reduction. Because aluminum-sulfate delays germination and attenuates growth at far lower concentrations than sodium-sulfate, concentrations in the ALU-treatment (Table S3) were lower by several orders of magnitude than the concentrations of sodium-sulfate in the SOD-treatment. For ramping of salt-stress, pre-planned step-increments in salt-concentration between selection-cycles were likewise informed by our pilot experiments, which suggested increments for aluminum-sulfate concentrations of about two- to five-fold for the first few microbiome-generations, and less than two-fold increments for sodium-sulfate concentrations, with gradual decrease in step-increments in later microbiome-generations so as not to over-stress plants (Table S3). Because we had to prepare hydrated soil for the next selection-cycle about 1-2 weeks before the end of a given Generation, we had to decide salt-stress increments for the next selection-cycle well in advance, using information from relative growth of younger plants in the sodium-sulfate and the aluminum-sulfate treatments. Decisions on salt-increments between Generations therefore typically involved some informed guessing, to adjust salt concentrations for the next cycle such that plants in either treatment were projected to germinate and grow at about the same rate (i.e., we aimed for plants in either salt treatment to grow to comparable sizes in the same time during a selection cycle). With such projected equal growth between sodium- and aluminum-treatments, microbiomes could be harvested at the end of a selection cycle from plants of comparable sizes (typically 9-15 leaves at the time of microbiome harvesting) regardless of whether a plant was stressed with aluminum-sulfate or sodium-sulfate (i.e., sodium-treated plants were not behind in growth compared to aluminum-treated plants, or vice versa). A second pre-planned feature of our salt-ramping design was to increase salt-stress in successive selection-cycles as long as differences in effect-sizes seemed to increase between salt- and control-treatments, but to reduce the salt-stress if differences in effect-sizes diminished or disappeared, possibly because of over-stressing the plants (see above *Logic of Salt-Stress Ramping*). This seemed to happen in Generations 4 & 5 (see Figure 1 in main text), and salt-stress was therefore reduced somewhat in the subsequent four Generations 6-9 (Table S3).

For hydration of 100 pots, we mixed, in a large carboy, 12 liter double-distilled e-pure water at a 50:1-ratio with 240ml Dyna-Gro 9-7-5 (Nutrient Solutions, Richmond, CA; www.dyna-gro.com/795.htm), plus an aliquot of 1-Molar salt solution (Table S3 lists salt-aliquots in recipes for salt-nutrient mixes) to generate the specific salt-stress planned for a particular selection cycle. [To prepare 1-Molar ALU-salt stock, we dissolved 307.94g anhydrous aluminum-sulfate in 900ml e-pure water in a 1-liter bottle; to prepare 1-Molar SOD-salt stock, we dissolved 289.98g decahydrous sodium-sulfate in 900ml e-pure water in a 1-liter bottle; then filter-sterilized each salt solution to prepare sterile stock.] We used different carboys to prepare salt-nutrient mixes for the different salt treatments (SOD, ALU). The nutrient concentration in each mix (Table S3) was sufficient such that plants did not need additional fertilization during each selection-cycle of 20-30 days during Generations 0-8 when we quantified plant fitness as above-ground biomass production, and plants even had sufficient nutrients to flower and grow seed during the 68 days of Generation 9 when we quantified plant fitness as seed production. For both salt treatments, fertilizer-salt solutions had a pH = 3.75 before addition to soil, but because of the buffering capacity of PPC soil (natural pH = 5.5, see above), the hydrated soil had a pH of about 5.0-5.5 after autoclaving soils, using the pH-measurement protocol in ISO/FDIS 10390 (2005). After hydration of all pots, we immediately autoclaved all pots (to minimize the time that any live microbes in the soil could consume any nutrients), and we autoclaved in separate 1-liter flasks at the same time 800ml of each of the unused salt-nutrient solutions; these autoclaved salt-nutrient solutions were used later during planting, and as buffer (at half-concentration) to suspend microbiomes harvested from rhizospheres for microbiome-transfers (see *Planting & Microbiome-Harvest* below).

Autoclaving of Soil: After hydration of soil by carefully pouring exactly 94ml of fertilizer-salt solution into a pot, we leveled and smoothed the soil-surface in a pot with the bottom of a glass (same size as interior diameter of a pot); taped to each pot a label of autoclavable label-tape (Fisherbrand^{TM}) with a pre-written pot-number (#001-100 for pots of SOD-treatment; #101-200 for pots of ALU-treatment) to the top side of

each pot (Figure S1); then used pre-cut pieces of aluminum foil to cap the top and wrap the bottom of each pot to prevent microbial contamination during seed-stratification (see below *Planting & Stratification*). Wrapped pots were arranged vertically in large autoclave trays (67 pots per tray, 3 trays total), the trays were covered with sheets of aluminum foil, then all pots in these 3 trays were sterilized simultaneously in a large autoclave. Hydration, labeling and capping of a set of 200 pots needed typically 5-6 hours. The subsequent autoclaving procedure lasted about 10 hours overnight, starting in the evening with a first cycle of 35 minutes autoclaving (121C° temperature, 20 atm pressure) with a slow-exhaust phase lasting 90 minutes; followed by overnight exposure to high temperature in the unpressurized autoclave; followed in the morning by a second cycle of 35 minutes autoclaving with a 90-minute slow-exhaust phase. This stringent autoclaving regime was sufficient to sterilize PPC soil, because plating on PDA-medium of about 0.5g soil (n=2 SOD pots, n=2 ALU pots) taken with a sterile spatula from the interior of such autoclaved pots produced no visible microbial growth within a month of incubation of these plates at room temperature. After cooling of autoclaved pots in the foil-covered trays at room temperature for at least 16 hours, we planted seeds into the sterilized soil (one seed per pot; see below *Planting*).

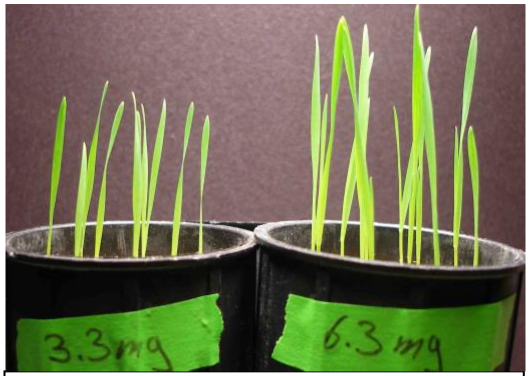

Figure S2. Pilot experiment illustrating growth variation of *B. distachyon* Bd3-1 plants growing under identical conditions from seeds weighing either 3.3mg or 6.3mg. The seed-weight range tested here includes about 90% of the 6000 seeds that we bulked before start of our microbiome-selection experiment. We used seeds of a narrow weight-window of only 0.1mg or 0.2mg for each microbiome-selection cycle (see Table S3), to help reduce within-generation and within-treatment variation in plant-phenotype (specifically here, reduce seed-weight-dependent maternal effects on plant phenotypes). Photo by UGM.

Seeds Preparation & Binning of Seeds by Weight: To have enough seeds for our 10-generation selection experiment, we first grew *Brachypodium distachyon* Bd3-1 plants under standardized light conditions (14h light, 10h dark) and room temperature in well-fertilized and well-watered greenhouse soil, harvested about 6000 seeds from these plants, then dried and stored seeds at room temperature (see above *Preparation of Brachypodium distachyon Seeds*). For our experiment, we used only long-awn seeds; that is, we discarded any short-awn seeds positioned peripherally in inflorescences (spikelet), and we discarded also any misshapen or discolored seeds. We used only long-awn seeds because these kind of seeds grow in more standardized central positions in a spikelet, because we could grasp an awn with a forceps during weighing and planting without risk of injuring a seed, and because we could plant seeds vertically into soil with only the awn protruding above the soil to reveal the exact location of a seed during later microbiome inoculation (see below *Seed Inoculation*). To weigh each seed accurately, we first removed any attached glumes to weigh only the seed with its awn. One experimenter pre-weighed each seed to bin seeds by weight to the nearest 0.1mg, then a second experimenter re-weighed all seeds in bins 4.5mg – 6.0mg again (i.e., each seed was weighed twice). To help reduce within-treatment variation in plant-phenotype (specifically here, reduce seed-weight-dependent maternal effects on plant-phenotypes, as illustrated in Figure S2), we used seeds of only a narrow weight-window for each microbiome-selection cycle. We used seeds of 5.9&5.8mg weight for the initial baseline Generation 0, then we used up seeds of bins of gradually decreasing seed-weight (5.9&5.8mg, 5.8&5.7mg, 5.6&5.5mg, …), as shown in Table S3 for each microbiome-generation.

Planting & Stratification: For planting of seeds in sterile soil, we first surface-sterilized Bd3-1 seeds in a laminar flow-hood by gently shaking the seeds for 8 minutes in 10% bleach [Chlorox®; 4ml bleach added to 36ml autoclaved e-pure water in a 50ml Falcon tube; plus 4µl Tween80-surfactant (Sigma-Aldrich, Saint Louis, MO, USA) to promote wetting of seeds], then rinsing the seeds three times to wash off bleach (three successive 1-minute gentle shaking, each in fresh 40ml e-pure autoclaved water in a 50-ml Falcon tube). In pilot tests, such surface-sterilized seeds placed on PDA-medium did not lead to bacterial or fungal growth. After rinsing, we blotted seeds on autoclaved filter paper, then air-dried the seeds in an open Petri dish in the flow-hood while preparing the flow-hood for planting inside the hood. To plant one seed into the center

of a pot, we removed the aluminum-foil lid from a pot inside the flow-hood, pushed a narrow hole into the center of the soil with a flame-sterilized fine-tipped forceps (#5 forceps), then inserted a seed into that hole such that the seed was positioned vertically in the soil and only the awn was protruding above the soil (i.e., the pointed tip of a seed was just below the soil surface). Because seeds used for a selection cycle had been binned to within 0.1mg weight (i.e., all seeds were of same size for each Generation), seeds were therefore planted at the same depth (to within about 0.5-1.0 mm identical depth), and any differences in initial germination rate (i.e., appearance of the shoot at soil surface) was unlikely due to differences in planting depth between seeds. To solidify the soil around each seed, we applied 4ml autoclaved salt-nutrient solution (same concentration that was used to hydrate soil in a given selection-cycle; Table S3) with a 5ml pipette to flush soil into the hole and completely cover each seed (excepting the awn protruding vertically above the soil surface). We covered each pot with a translucent, ethanol-sterilized lid (inverted Mini Clear Plastic Bowl 40ct; Party City, Rockaway, NJ, USA). The lids prevented entry of airborne microbes into each pot, but did not seal pots completely and permitted some gas exchange at the bottom of each lid overlapping the top of a pot. Each lid measured 5.7cm diameter x 3.8cm height, and fit snugly on each pot such that a series of 50 capped pots could be kept in a rack (D50T rack, see above) without the lids interfering with each other. We placed each rack of 50 capped pots into its own ethanol-sterilized plastic tub (Jumbo Box; Container Store, Coppell, TX), covered the tub with the tub's lid, then sealed the spaces at the side of each lid by wrapping lid & tub with a 2-meter-long strip of 10-cm-wide Parafilm to prevent entry of contaminants during subsequent cold-storage for stratification of seeds. We moved each tub into cold-storage immediately after completing the planting of 50 pots (= one full rack). For stratification, we stored the tubs with planted seeds in a 5°C cold-room for about 5 days (range 4-10 days, the duration differing slightly between Generations because of scheduling-constraints affecting planting). Planting of a set of 200 seeds (4 racks of 50 pots each) using the above methods needed typically 4.5-5.5 hours.

Preparations for Microbiome-Harvesting: To prepare salt-nutrient buffer-solution for microbiome harvesting, we used the autoclaved salt-nutrient solution that we had prepared for hydration of soil for a particular selection-cycle (see *Soil Hydration* above; Table S3), then diluted the solution to half-concentration by addition of an equal volume of autoclaved e-pure water. We decided to use for microbiome-harvesting the salt-nutrient solution at half-concentration, because we were concerned that the full-concentration may have too high osmolarity compared to the osmolarity that may exist in the soil after weeks of root- and microbiome-growth in the soil; this dilution precaution may not have been necessary, and it may be possible to harvest and propagate microbiomes even with the full-concentration of salt-nutrient buffer. Aliquots of 45ml of the sterile, half-concentration salt-nutrient buffer were added to 50ml Falcon tubes in a laminar-flow hood, and these tubes were then pre-labeled with relevant information (SOD vs ALU treatment; Generation #; date of microbiome-harvest) to save time on the actual day of microbiome-harvest. To sterilize microfilters needed for fractionation of harvested microbiomes (2µm Whatman™ filters; model Puradisc 25 GD2 Syringe Filter, 25mm diameter; Whatman PLC, United Kingdom), we wrapped filters individually in aluminum-foil, then autoclaved these in a 15min-exposure fast-exhaust cycle. On the evening before the day of microbiome-harvest, we set up a custom-made flow-hood on a bench in our Conviron growth-chamber, sterilized the inside of the hood by spraying liberally with 100% ethanol, then allowed the flow of clean air to purify the inside of the hood overnight. Our custom-made hood was constructed of a large plastic tub placed on its side, with the lid cut half so that a lid-portion affixed to the tub could shield the inside of the hood from above (like a sash on a regular flow-hood), whereas the bottom half was kept open to permit access to the inside of the hood. To generate a flow of clean air through the hood, we cut a large hole into the top of the hood (i.e., one of the original sides of the tub now resting on its side) to fit into that hole the top portion of an air purifier (model HPA104 Honeywell HEPA Allergen Remover, with HEPA filter of 0.3 microns; Honeywell International Inc., Morris Plains, NJ, USA). We operated the purifier at medium flow-setting, which generated an even flow through the hood and minimized any air-vortices that could draw impure air into the hood at high flow-setting. In a pilot test, Petri-plates with PDA-medium, exposed overnight to the flow inside our hood, revealed no visible growth within seven days of incubation of these plates at room temperature. Early on a day of a between-

generation microbiome-transfer, we moved the tubs with racks of planted, cold-stratified seeds from the cold-room into our growth-chamber, to have sufficient time for completion of all microbiome-harvests and -transfers (the total time needed on the day of microbiome harvest for completion of harvests/transfers of all lines was 8-10 hours, plus an additional 2 hours for distribution of pots in pre-determined randomized arrangements across 8 racks used to support pots in the growth-chamber). We began microbiome-harvests and -transfers immediately after moving pots with vernalized seeds into the growth chamber, so transferred microbiomes would interact with seeds at the very early stages of germination.

Phenotyping of Plants; Quantification of Above-Ground Biomass: To select the two best-growing plants from a particular selection line on the day of microbiome-harvest and -transfer, we moved all eight pots from a selection line into a separate, ethanol-sterilized rack, recorded the number of leaves of each plant, and arranged plants visually by apparent above-ground biomass into a size-ranked series (Figure S3). We chose visual sizing rather than weighing for phenotyping of plants, because visual evaluation of all eight plants in a selection-line needed only about 5-10 minutes (including recording the number of leaves for all eight plants), and because microbiomes could be harvested immediately after visually identifying a particular plant for microbiome harvest without first having to cut and weigh above-ground biomass of all plants in a selection line. We harvested rhizosphere microbiomes from only those two plants within a selection line that we visually judged to have grown the largest and second-largest above-ground biomass (Figure S3).

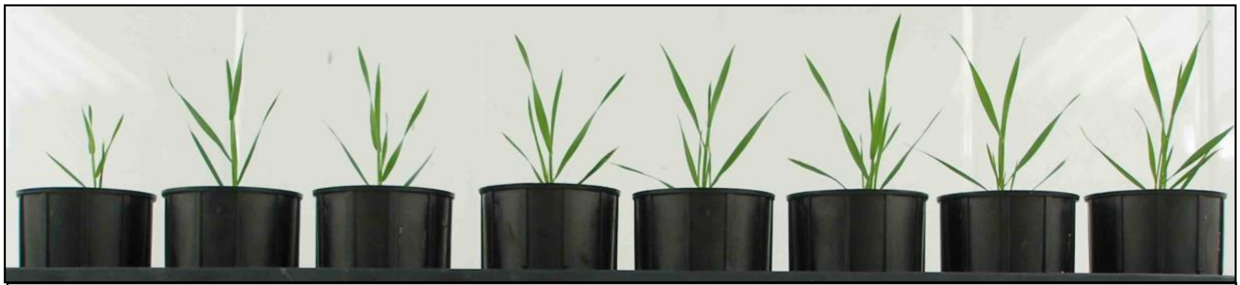

Figure S3. Plants of the same selection-line ranked by visually estimating above-ground biomass. The 8 plants were grown in 8 different racks (one plant per rack) in randomized positions in each rack, and the 8 plants were moved to a separate ethanol-sterilized rack for visual comparison immediately before microbiome harvesting from the two largest plants. See text for further details on *Phenotyping of Plants; Quantification of Above-Ground Biomass*. Photo by UGM.

To test the accuracy of our visual rankings, we later compared these rankings with dry above-ground (shoot) biomass of each plant in a selection line. To weigh shoot-biomass, we cut each plant at soil-level at the time of microbiome harvesting, stored above-ground biomass for drying in an individual paper envelope (Coin Envelope 8cm x 14cm), dried these envelopes/plants for at least two weeks at 60°C in a drying oven, then weighed dry biomass for each plant to the nearest 0.1mg. Although we judged above-ground plant-biomass visually on the day of microbiome harvesting, of the 80 lines judged during our entire experiment (5 SOD-lines + 5 ALU-lines judged each Generation, times 8 Generations; Table S1), we picked for microbiome harvest the combination of largest (#1) and second-largest (#2) plants in 56.25% of the cases; the largest (#1) and third-largest (#3) plants in 27.50%; the largest (#1) and fourth-largest (#4) plants in 6.25%; the second-largest (#2) and third-largest (#3) plants in 5.00%; the second-largest (#2) and fourth-largest (#4) plants in 5.00%; and never any lower-ranked combination. In cases where we did not identify visually the combination of #1 and #2 plants as determined later by dry weight, the slightly lighter #3 or #4 plants were typically within 0.2-4mg (0.5-9% of total dry-weight) of the two best-growing plants in the same selection-line. Moreover, because harvested microbiomes of the two chosen plants were mixed for propagation to the next microbiome-generation (see below *Microbiome Mixing*), we harvested in 100% of the cases the microbiomes from either the best-growing or second-best growing plant into the mixed microbiome that we then propagated to the next microbiome generation (i.e., a microbiome of one of the two best-growing plants was always included in the propagated microbiome mix). In sum, therefore, our method to visually judge plant size was both time-efficient (about 5-10 minutes to visually size all plants in a selection-line

and record number of leaves for each plant), and our method was also accurate to identify those plants that had grown biomass well above-average within any given selection-line (i.e., our methods were accurate to visually identify plants that were likely associated with microbiomes that conferred salt-tolerance to plants).

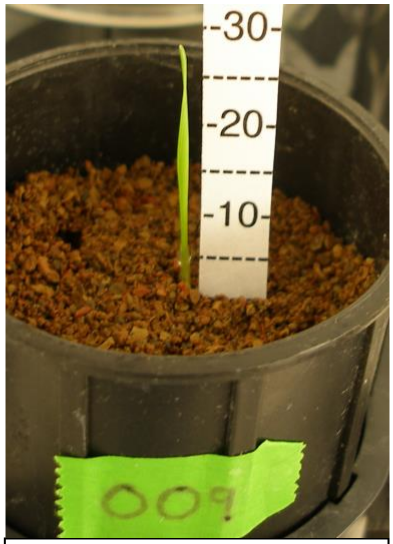

Figure S4. Measuring length of the first leaf on Day 3, using an ethanol-sterilized dry paper-strip with printed millimeter-scale. Photo by UGM.

In some cases, on the day of microbiome-harvest, more than two plants of the same selection line appeared to have the largest above-ground biomass. To decide between those plants for microbiome-harvest, we considered as a second criterion also the growth trajectory recorded from the day of germination to the day of microbiome-harvest, choosing then the plant with the best growth-trajectory. We quantified growth trajectory of plants during each generation with three methods: (i) measuring the length of the first leaf on Days 2-5; (ii) after Day 5, recording the number of leaves grown by a plant every other day up to a time when plants had grown about 10 leaves; and (iii) once plants had grown about 10 leaves, visual ranking of relative plant size (visual appearance of overall biomass) on a 10-point scale from 1-9, using the protocol below. ***Length of first leaf*:** After moving pots from the cold-room into the growth-chamber, the fastest-growing shoots became visible, as they pushed through the soil, after about 44 hours in the early, low-salt Generations, but growth rate was somewhat slower in the later, high-salt Generations when the first shoots became visible after 55-70 hours. To quantify this early growth each selection-cycle, we estimated length of the first leaf during Days 2 & 3 visually without lifting the translucent lids from pots, but measured leaf-length on Days 4 & 5 to the nearest millimeter with an ethanol-sterilized ruler (millimeter scale printed on paper strip; Figure S4) held next to the growing leaf, using a different sterile paper-ruler for each plant so as not to transfer microbes between pots. In blind, repeat evaluations, the visual sizing on Days 2 & 3 is accurate to about ±0.5mm for leaves less than 15mm tall, and accurate to about ±2mm for plants larger than 25mm. Despite the somewhat lower accuracy of the visual leaf-length estimation compared to the precise measurement with a ruler, we chose to visually size plants on Days 2 &3 because that method allowed us to leave the pots covered with the translucent lids, thus preventing any influx of microbes when lifting a lid; plants therefore interacted only with the experimentally-transferred microbiomes for the first 4 days of growth without any influx of additional microbes, thus facilitating priority effects in microbiome recruitment into the initial microbiome assembled by a plant. ***Counting leaf number*:** The fastest-growing plants showed growth of a second leaf typically late on Day 5 (in the early low-salt Generations) or on Day 6 (in the later high-salt Generations). We counted the number of leaves regularly after Day 6, typically every other day. ***Above-ground biomass estimated on a 10-point scale ranging from 0-9*:** This third method gave the most precise estimate of above-ground biomass once plant had grown more than 10 leaves, and we used this method therefore every generation to obtain a relative measure of above-ground biomass a few days before microbiome harvesting. An experimenter first looked over all plants to gain an impression of the largest plants, of the appearance of average-sized plants, and of the smallest plants, then subdivided the entire range on a subjective 0-9 point-scale, with plants of average size to be scored as 4.5 on the 0-9 point-scale. Evaluating all plants rack-by rack, the experimenter scored and recorded sizes of all 200 plants in a Generation, then blindly re-scored all plants again rack-by-rack, then calculated an average between the 1st & 2nd size-values for each plant. Comparison of the 1st & 2nd size-values for each plant showed that about 70% of the blind re-scoring were identical between 1st & 2nd size-values; and in most of the remaining 30% cases, 1st & 2nd size-values of the same plant differed by only a 1-point-value, and only in very exceptional cases (<2%) the size-values differed by 2-points on our scale. Because of this high repeatability of this scoring method, we used this method every generation to obtain estimates of the relative above-ground biomass of each plant 1-3 days before each day of microbiome harvesting.

Microbiome-Harvesting from a Rhizosphere & Microbiome Mixing: We performed all steps of microbiome harvest and microbiome transfer in a clean-air flow-hood (see above) set up on a bench inside our growth-chamber (i.e., we did not have to move microbiomes/pots of selection lines outside the growth chamber), and we sterilized hands and work-surfaces regularly with 100% ethanol to prevent contamination of samples. After choosing the two plants with the greatest above-ground biomass (see above *Phenotyping of Plants*), we cut each plant at soil level with ethanol-sterilized scissors, stored the above-ground portion in an envelope for drying, and harvested rhizosphere microbiomes immediately to minimize microbiome changes in the absence of plant-control in the rhizosphere. To extract the root-system from a pot (Deepot) with minimal contamination, we held the shoot-stub at the soil surface with ethanol-sterilized forceps, tilted the pot such that PPC-soil would gradually loosen and fall out when squeezing the plastic pot, until the root-structure could be extracted as a whole by gentle pulling at the main root with the forceps. In most cases, the entire root structure could be extracted whole, with some loss of fine roots embedded in spilled soil. Because we were interested in harvesting microbiomes that were in close physical association with a plant (i.e., we were interested in rhizoplane bacteria, plus any endophytic bacteria if they were released during root processing as a result of any root damage), we discarded any soil adhering loosely to the roots. We dislodged loosely adhering soil by knocking the root-system gently against the wall of an autoclaved aluminum-pan (e.g., Hefty EZ Foil Roaster Pan; 32cm length x 26cm width, vertical depth 11cm) such that any dislodged soil would fall into the pan without the roots contacting any discarded soil. We then cut off the top 2 cm of the root-system (i.e., roots close to the soil surface), then transferred the remaining root-system into a 50 ml Falcon tube filled with 45 ml of salt-nutrient buffer (the same buffer used also to hydrate soils of the subsequent microbiome-generation, but diluted to half-concentration to suspend harvested microbiomes; see above *Preparations for Microbiome-Harvesting*). We repeated this process with the second plant chosen for microbiome-harvest from the same selection line, and added this second root-system to the same Falcon tube as the first root-system. Combining both root-systems for microbiome-harvesting generated a so-called mixed-microbiome collected from two 'mother' rhizospheres (see *Mixed Microbiome Propagation*; and Box 3 in Mueller & Sachs 2015), which we then transferred within the same selection line to all eight 'offspring' plants/seeds of the next microbiome-generation.

Microbiome-Fractionation with Microfilters: To dislodge microbes from roots and from soil-particles adhering to roots, we turned a closed Falcon tube upside-down 50 times, then permitted soil-particles to settle in the bottom of the tube for 1 minute. A 1cm-deep sediment of PPC-soil particles typically accumulated in the bottom cone of a Falcon tube, with the roots settling on top of this sediment, and small particles and colloids remaining suspended in the salt-nutrient buffer. We aspirated 20 ml of this suspension with a sterile 20 ml syringe (external syringe diameter fitting into a 50 ml Falcon tube), then attached to the syringe's Luer-lock a 2 µm Whatman microfilter (model Puradisc 25 GD2 Syringe Filter, 25 mm diameter; Whatman PLC, United Kingdom), then filtered the aspirated suspension into an empty sterile 50 ml Falcon tube. Making sure that the exterior of the syringe did not become contaminated during this first filtering, we repeating this step with the same syringe to filter another 15-20 ml of the suspension, then mixed the combined filtrates by inverting the Falcon tube several times. The total volume of 35-40 ml filtrate was sufficient to inoculate 8 'offspring' plants/seeds each with 4 ml filtrate (total of 8 x 4 ml = 32 ml needed). In pilot tests, plating on PDA-medium 10 µL of this filtrate (2µm filter) yielded thousands of bacterial colony-forming-units (CFUs) but no fungal CFUs within 24 hours growth; whereas plating on PDA-medium 50 µL of this same filtrate that had been filtered a second time with a 0.2µm filter (VWR Sterile Syringe Filter, 0.2µm polyethersulfone membrane, 25mm diameter; Catalog #28145-501; retains even the very-small-sized bacteria, such as *Brevundimonus diminuta*) did not yield any visible microbial growth on these PDA plates kept for 7 days at room temperature. These results justified addition of a third control-treatment in Generation 9 (0.2µm filtration of suspension; *Solvent Control*) to test growth-promoting effects of root exudates, soil nutrients, and viruses that are unavoidably co-harvested with harvested bacterial microbiomes. Although a 0.2 µm filter may not eliminate ultra-small bacteria (e.g., Luef *et al* 2015; we did not use filters of smaller pore size because it was too difficult to press liquid through such filters), our control comparison between 2.0 µm-filtered and 0.2 µm-filtered bacterial microbiomes can still test whether

the bulk of the bacterial microbiome (in size range 0.2-2.0 µm) or alternatively any smaller-sized organisms (viruses, ultra-small bacteria) are responsible for conferring salt-tolerance to plants.

Inoculation of Seeds; Transfer of Microbiomes to Plants of the Next Microbiome-Generation: During planting, the 200 pots of each microbiome generation had been ordered numerically in the 4 racks used for stratification in the cold-room, so it was easy to locate in these racks a pot with a particular number that had been assigned to a specific selection-line and needed to be inoculated with a microbiome. To inoculate a seed planted in a particular pot, we moved the pot into our clean-hood in our growth chamber, opened the pot's translucent cap inside the hood (using one hand to hold the pot while opening the cap with thumb and index finger of that same hand), then used a 5 ml pipetter to transfer 4 ml of the microbiome-filtrate to the center soil in a pot where a seed had been planted before vernalization/stratification. We spread the 4 ml filtrate across an area with a radius of about 5mm around a seed, applying some of the filtrate directly onto the seed (the exact location of the seed was indicated by its awn protruding above the soil; see *Planting* above), and we spread some of the filtrate also in a circle onto the surrounding soil within 5 mm distance of a seed. To keep the filtrate well-mixed during the time needed to inoculate all 8 'offspring' soils of the same selection-line, we repeatedly mixed the filtrate in the Falcon tube with the pipette-tip before aspirating a 4 ml-aliquot to inoculate the next pot. We then taped a small tag of labeling-tape to the lid of each pot that had received an inoculum (as a check to verify later that all pots had received an inoculate, no pot/seed was accidentally skipped, and no pot/seed was accidentally inoculated twice), then we returned the pot to its appropriate position in one of the four racks. After inoculation of all 200 plants within a Generation, all pots were distributed among the 8 racks used to support plants in the growth chamber (see below *Randomization of Pot-Positions in Racks*).

Each pot was capped for the first 4 days to promote priority effects during microbiome establishment (i.e., capping prevented immigration of extrinsic microbes into the soils/microbiomes for the first 4 days; see above *Planting*), but all caps were removed on Day4 because the tallest plants (35-40mm tall on Day4) were close to reaching the cap-ceiling. We monitored growth during the first 5 days (see above *Phenotyping*) by recording length of the first leaf on Days 2-5, and recording day of appearance of the second leaf (typically on Days 6 or 7). Seeds that did not germinate or that germinated very late (i.e., no above-ground growth visible by Day 4) were extracted from pots with forceps and inspected. Most of these seeds had failed to grow both a rootlet and shoot by Day4, but some seeds had grown a rootlet but no shoot. In a typical microbiome generation, about 88-100% of the plants showed a visible shoot within the first 3 days. Germination rates were therefore good overall, and most lines had the planned 8 replicates (sometimes 7 replicates, rarely 6 replicates, if some seeds failed to germinate; see Tables S1 & S2). Germination-rates were often minimally higher in the Null-Control treatments compared to other treatments of the same soil-stress (slightly fewer non-germinating seeds in Null-Controls); and, across all plants, germination-rates were minimally higher in ALU-soil than in SOD-soil (Tables S1 & S2); we did not analyze these trends for statistical significance because differences seemed minimal, but we simply note here these general patterns that became apparent only when pooling information across all 10 Generations.

Randomization of Pot-Positions in Racks in Growth-Chamber: Deepots were supported in D50T racks (Stewe & Sons, Tangent, Oregon, USA). Each rack can hold a total of 50 pots (5 rows of 10 pots each), but to prevent contact of leaves from different plants and to reduce accidental between-pot transfer of microbes during watering (see below *Watering*), we used only 25 rack-positions (25 pots per rack, total of 8 racks, for a total number of 200 pots per selection cycle). Pots within a selection line were first assigned by blocking to a particular rack (e.g., of the 8 replicates within a selection line, one replicate was assigned to each of the 8 racks. Within each rack, however, we randomly assigned pot positions, using the *Random Sequence Generator* option at Random.Org (www.random.org/sequences/). For Generations 0-8 (growth cycles 0-8), Table S1 lists pot positions (#1-#25) from different treatments within each rack (Rack #1-8), corresponding to the following pot arrangement:

#1		#2		#3		#4		#5	
	#6		#7		#8		#9		#10
#11		#12		#13		#14		#15	
	#16		#17		#18		#19		#20
#21		#22		#23		#24		#25	

For the final Generation 9 when we added two more control-treatments (details below), we randomized 400 pot-positions by first assigning a pot to one of the 8 racks, then randomizing position within each of the 8 racks (50 pots/rack; the position-numbering of pots shown for Generation 9 in Table S2 for each rack is numbered consecutively, starting in left top corner, without leaving empty spacer-slots between pots).

The 8 racks were positioned in two groups of 4 racks each on two comparable shelves at either side of the growth chamber. Within each selection cycle, we rotated these 8 racks in clockwise rotation each day (moving one rack from right shelf to left shelf, and one rack from left to right shelf), and at the same time we also turned each rack (such that the rack-side facing the chamber wall one day faced the chamber center the next day). This rotation-turning scheme aimed to minimize possible environmental influences dependent on location of a rack on the two shelves, and to reduce any minimal differences in light-level, air-circulation, or any such uncontrolled environmental factors that may exist between different positions on the two shelves in our growth chamber. Despite our effort to minimize rack effects through daily rack-rotation and rack-turning, as well as randomization of processing order (e.g., watering, phenotyping, microbiome-harvesting), we had occasionally racks of poorer or better plant growth (e.g., Rack 7 of Generation 9 had lower average seed production compared to other racks, because many plants in that rack did not flower, or flowered late). We do not know the exact causes for occasional small rack-effects.

Starter Inoculum for Microbiomes at Beginning of the Experiment for Baseline Generation 0: We used a single microbiome-batch to inoculate all replicate pots of the initial baseline Generation 0. To prepare that inoculum, we filtered bacterial communities from a mix of roots and adhering soil taken from three principal sources: (a) root-systems with adhering soil of three local grass species (*Bromus* sp., *Andropogon* sp., *Eragrostis* sp.) collected into individual plastic bags on 3. Jan. 2015 (about 90 minutes before microbiome harvesting) at restored native habitat at Brackenridge Field Lab of the University of Texas at Austin (www.bfl.utexas.edu/); (b) root-systems with adhering soil of 40 16-day-old *B.distachyon* Bd3-1 plants grown in PPC-soil Deepots as part of a pilot experiment quantifying the effect of salt in soil on the growth rate of *B. distachyon* (see below *Salt Treatments*); and (c) old root-systems with adhering soil of 15 Bd3-1 plants grown in PPC-soil Deepots, but that had been stored in the soil/Deepots in a cold-room (6°C) for 7 months after completion of a previous low-nutrient microbiome-selection experiment. We combined roots and rhizosphere soils from these three sources in order to capture a diversity of microbes into our starter inoculum, and we included Bd3-1 rhizospheres in order to capture specific microbial taxa that may be readily recruited by *B. distachyon* into its rhizosphere microbiomes. We suspended this mix of roots and rhizosphere soil in 200 ml e-pure water, blended the mix for 30 seconds in an autoclaved Waring blender to generate a liquid slurry, allowed the solids to settle in the blender for 1 minute, then decanted the supernatant into a separate autoclaved beaker. Adding each time 200 ml e-pure water, we repeated this blending/decanting with the remaining slurry three more times to collect a total of about 600 ml supernatant. Using vacuum filtration, we pre-filtered this supernatant in a Buchner funnel through filter paper (Ahlstrom filter paper S02-007-42), eliminating larger particles suspended in the supernatant. To harvest only bacterial microbiome components (and viruses) from this pre-filtrate, we filtered the supernatant a second time in a laminar-flow hood, using a sterile 60 ml syringe fitted with a sterile 2 µm Whatman™ microfilter (Puradisc 25 GD2 Syringe Filter, 25 mm diameter; Whatman PLC, United Kingdom) to generate the bacterial mix for inoculation of replicate pots of our initial baseline Generation 0. Because the Puradisc filters became clogged after filtration of about 70-100 ml supernatant, we used 8 Puradisc filters to process about 600 ml of filtrate. We reserved 500 ml of this filtrate for inoculation of 160 randomly-assigned pots in a Bacterial-

Inoculate treatment (80 Bacterial-Inoculate with SOD soil, 80 Bacterial-Inoculate with ALU soil), and filtered the remaining 100 ml with 0.2 µm filters (VWR Sterile Syringe Filter, 0.2µm polyethersulfone membrane, 25 mm diameter; Catalog #28145-501) for inoculation of 40 pots in Null-Control treatments (20 Null-Control with SOD soil, 20 Null-Control with ALU soil). The Null-Control treatments controlled for, after elimination of bacteria, the effect of any chemicals and viruses that may have been co-harvested from rhizosphere roots and soils. Seeds in the Bacterial-Inoculum and the Null-Control treatments were inoculated following the procedure described above (see *Inoculation of Seeds*), except that each seed of Generation 0 received 2 ml inoculate, whereas each seed of subsequent Generations 1-9 received 4 ml inoculate transferred between generations. During inoculation of seeds, we mixed the stock filtrates regularly to prevent bacterial sedimentation and to insure standardized inoculation of all replicates across all treatments. We needed about 3 hours to complete the entire process from root collection to conclusion of all filtration steps, and another 2 hours to apply inoculate-aliquots of the filtrates to each of the assigned pots. We then moved all pots immediately into our growth chamber, and set out all 200 pots of Generation 0 into randomized positions in 8 racks (see above *Randomization of Pot Positions*; Tables S1 & S2).

To test for live bacteria in our 2 µm filtrate used as the Starter Inoculum, we plated on PDA-medium (2 replicate plates) 10 µL each of the 2 µm filtrate and maintained plates at room temperature; the plates showed thousands of bacterial colony-forming-units (CFUs) within 24 hours, but no fungal growth within 7 days. To test for absence of live bacteria in our 0.2 µm filtrate, we plated on PDA-medium (3 replicate plates) 50 µL each of the 0.2 µm-filtrate; these platings did not yield any visible growth on the PDA plates kept for 7 days at room temperature. These results indicate (i) a great abundance of live bacteria (and apparently no live fungi) in our initial inoculum, and (ii) elimination by the 0.2 µm filters of live bacteria that would be apparent when plating out such filtrate on PDA plates. The latter justified our use of a third control-treatment in Generation 9 (0.2 µm filtration of suspension to test growth-promoting effects of chemicals and viruses co-propagated with the harvested bacterial microbiomes; see *Solute-Control* below).

Selection of Microbiomes from Generation 0 to Inoculate Plants from Generation 1: At the start of our experiment, we did not assign microbiomes (i.e., pot numbers) from Generation 0 to specific selection lines, to permit selecting the best-growing plants from Generation 0 to contribute microbiomes to the selection-lines starting with Generation 1. We chose this particular assignment rule because random assignment to selection lines would result in some cases for a poorly-growing plant to contribute microbiomes to Generation 1, and we wanted to increase the chance of obtaining a response to microbiome selection in the fewest rounds of selection. To select plants for harvesting and propagation of rhizosphere microbiomes, we ranked, separately for plants in the SOD and ALU treatments, the plants in the Bacterial-Inoculate treatments of Generation 0 by relative size, then picked the 10 best-growing plants of each salt-treatment to contribute microbiomes to the selection-lines that we started with Generation 1 (Table S1). On Day 22 of Generation 0 (day of microbiome harvest and microbiome transfer, we first ranked plants by relative size-scores (i.e., average size-score averaged across three scores received by a plant on Days 18, 19, 20; see protocol *Above-Ground Biomass Estimated on a 10-Point Scale*), then used number of leaves recorded on Day 21 as a second criterion to differentiate between plants of equal size-score. Among the 10 best-growing plants within each of the SOD and ALU salt-treatments, we paired plants randomly to generate 5 combinations (2 plants each) for mixing of harvested microbiomes within each pair (i.e., harvested root-systems were combined from the two plants to harvest a mixed microbiome from both plants, as described above for *Microbiome Mixing*). Within each of the SOD and ALU treatments, the 5 mixed microbiomes from Generation 0 were assigned randomly to 5 SOD and 5 ALU selection lines (each with 8 ‘offspring' microbiome replicates per line) that started with Generation 1. Microbiomes were harvested and processed from chosen rhizospheres as described above. At the end of Generation 0, as well as at the end of each subsequent Generation, we cut all plants at soil level to preserve above-ground growth for later weighing of dry biomass for each plant (Tables S1 & S2; see also above *Phenotyping*).

Salt- and Control-Treatments in Generation 0-9; Sample Sizes Per Treatment: Starting with Generation 1 and continuing until the last Generation 9, we included the two aforementioned salt-treatments

(SOD and ALU soil) with *5 SOD Microbiome-Selection* Lines (8 replicates each, for a total of 40 replicates) and with 5 *ALU Microbiome-Selection* Lines (8 replicates each, for a total of 40 replicates). Also starting with Generation 1 and continuing until the last Generation 9, we included two control treatments for each of the SOD and ALU treatments, *Null-Control* (on SOD- and on ALU-soils) and *Fallow-Soil Microbiome Propagation* (on SOD- and on ALU-soils). ***Control 1, Fallow-Soil Microbiome Propagation***: For this control, we harvested microbiomes from fallow soil (from pots without a plant), then propagated the harvested microbiome to sterile fallow soil to perpetuate 'Fallow-Soil Microbiomes' in the absence of plant influences (e.g., absence of plant exudates into the soil). Fallow-soil pots were treated throughout each selection-cycle exactly like pots with plants; for example, these fallow-soil pots received the same amount of water whenever all other pots were watered. Each Fallow-Soil-Line had only one replicate pot, so a microbiome harvested from fallow-soil was propagated to a single pot of the next selection-cycle to continue a particular Fallow-Soil-Line; a portion of the same microbiome from the same pot was also transferred to pots with plants of the next cycle, to evaluate the effect of a harvested fallow-soil-microbiome on plant growth (but those microbiomes were later not propagated to subsequent Generations; i.e., these inoculations of control plants aimed at assaying the effect of un-selected fallow-soil-microbiomes on plant growth under the increasing salt stress that we increased stepwise between Generations; see above *Logic of Salt-Stress Ramping*). We chose a control of fallow-soil microbiome-propagation because this treatment resembles the kind of microbiome conditions that many plants encounter in horticulture and agriculture (soils are left fallow for some time before planting). Changes in fallow-soil microbiomes between Generations reflect ecological changes as microbe communities change over time, as well as any microbial immigration from external sources (e.g., airborne microbes raining into the soil; perhaps also unintended accidental cross-contamination between soils from different pots). We initially allocated 8 control-replicate test-plants per Fallow-Soil-Line to test the effect of each harvested fallow-soil microbiomes on plant growth (total of 5x8=40 replicates for SOD, 5x8=40 replicates for ALU), but we reduced the number of control-replicate test-plants for each of the 5 Fallow-Soil-Control replicates per line in later Generations (first reducing the number to 6 control-replicate test-plants per line in Generation 4; then reducing the number to 4 control-replicate test-plants per line in Generation 5-9), because it became clear during the first few Generations that plants receiving fallow-soil-microbiomes grew poorly under the salt stresses, far inferior to plants in the corresponding selection-lines where plants received artificially selected microbiomes (i.e., we could differentiate averages between fallow-soil and microbiome-selection lines even with the smaller number of control-replicate test-plants in the fallow-soil controls). **Control 2, *Null-Control***: For this control, plants received no experimental microbial inoculation; instead, these control plants received on the day of microbiome transfer an aliquot of the same sterile salt-nutrient buffer that we used to harvest microbiomes and then transfer to seeds of the next Generation. Because our pots were capped for the first 4 days of seed germination, Null-Control-plants grow initially under sterile conditions (before caps are lifted on Day4), but airborne microbes could enter the sterile soil and rhizospheres of Null plants from the air after Day4 once caps are lifted from pots. In pilot experiments, Null-Control plants invariably grew better during the first 10-20 days than any plant inoculated with microbiomes, possibly because Null-Control plants do not need to expend resources to mediate interactions with microbes, or because Null-Control plants do not have to compete with microbes for nutrients in the soil. Despite the microbially unusual soils of Null-Control plants, we included this control treatment because it was easy to set up (no microbiomes needed to be harvested to inoculate Null-Control soils), because Null-Control conditions were easy to standardize within Generations, and because Null-control Conditions may even be standardized between Generations if microbial immigration (i.e., rain of airborne microbes) into Null-Control soils can be assumed to be relatively constant over time. We initially allocated 10 replicates of SOD pots and 10 replicates of ALU pots to Null-Controls, but we increased the number of replicates in later Generations for the Null-Control treatments (first we increased to 20 replicates in Generation 4, then to 30 replicates in subsequent Generations) in order to improve the estimates (reduce confidence intervals) of the average growth of plants in Null-Control treatments. Tables S1 & S2 list sample sizes for all treatments for each of Generations 0-9.

Watering During Each Selection Cycle: We watered pots such that the total weight (pot plus hydrated soil) per pot remained between 200-250g and did not exceed 260g. We found in pilot experiments that a pot would be over-hydrated if the total weight reached 260-270g or more, which would result in dripping of excess water from the bottom of the pot, thus leaching nutrients and salts. Keeping pot weights below 260g at all times therefore prevented leaching of nutrients and salt. To prevent cross-contamination (microbe-exchange) between pots, we did not use bottom-hydration by immersing racks in a waterbath, but we watered pots individually, only from above, and always with autoclaved water that we dispensed with a Seripetter Dispenser (adjustable to dispense volumes of 2.5-25ml; BrandTech Scientific Inc; Essex, CT, USA) mounted on a 6-liter carboy. Because we kept pots capped during the first 4 days of plant growth (we removed caps during the afternoon of Day 4), soils remained well-hydrated during germination (little water evaporated from soil, humidity inside the cap was near 100%). We watered for the first time on Day 5 of each selection-cycle, and thereafter approximately every 2 days (sometimes also at 1-day or 3-day intervals, depending on humidity in the growth chamber and on experimenter time-constraints), but we did not pre-plan to follow a rigorous 2-day watering schedule (see Table S4). We typically watered 15-25 ml per pot depending on water loss, which depended on humidity in the growth chamber and on the size of plants (humidity was greatest during Generations 4&5 because of unusually high rainfall in spring 2015). To determine the volume to be watered on a given day, we selected six pots haphazardly from 4 racks, and weighed these on a scale (sterilizing the surface of the scale with 100% ethanol before placing a pot onto the scale). The difference between the average weight of these six pots and 255mg was the maximum quantity of water to be added to each pot. The amount to be watered could be varied to the nearest 0.5 milliliter with the carboy-mounted Seripetter Dispenser. To prepare carboys, we filled each with 6 liter of e-pure water, and autoclaved the water to ensure sterile watering. Immediately before watering, we quickly opened a carboy to add a specific volume of 1-Molar salt solution to generate a desired salt-concentration in the water (recipes listed in Table S3), mixed the contents by vigorous shaking of the carboy, mounted the ethanol-sterilized Seripetter Dispenser onto the carboy, flushed the dispenser five times to eliminate any ethanol in the dispenser, then began the watering. During the days when the Seripetter Dispenser was not used, we mounted it on a 1-Liter bottle with 100% ethanol, and kept the entire dispenser filled with ethanol to prevent growth of microbial biofilms inside the dispenser. We used different carboys dedicated to watering of SOD-salt and ALU-salt, to minimize cross-contamination of salts between treatments. In each round of watering, we first watered all pots of the SOD-treatment, then rinsed the dispenser with 100% ethanol, then watering all pots of the ALU-treatment. To minimize the chance of accidentally adding the wrong salt-water to a pot (e.g., accidentally watering ALU-soil with SOD-water, or vice versa), we labeled pots of the different salt treatments with different colors (white-label for pots ##001-100 to indicate SOD-treatment, and green-label for pots ##101-200 to indicate ALU-treatment). Table S4 summarizes the exact watering schedules, volumes of water added, and salt concentrations of the water added.

Flowering: Because we short-cycled plants in Generations 0-8 and harvested microbiomes when plants were relatively young (20-30 days old; the largest plants had typically 10-15 leaves, Table S3), only few plants bolted and developed flowers during the short-cycled Generations 0-8; these few cases of flowering were in Generations 1 & 8, whereas no plants flowered in Generation 0 and in Generations 2-7. The long light-phase (20h light, 4h dark) stimulated flowering uniformly in each Generation, but our short-cycling scheme aimed to harvest microbiomes well before plants began to flower in Generations 0-8. Because of scheduling-constraints, Generation 8 was grown for slightly longer (31 days) than earlier Generations, which could explain the flowering in some of these plants, but it is unclear why some plants began to flower in the far shorter Generation 1 (20 days). Plants in Generation 9 were grown for 68 days (see above) to permit seeds to ripen, and most of these plants produced at least some seeds (Table S1). The fact that not all plants flowered in Generation 9, and the observation that onset of flowering was delayed in the control-treatments, indicate that plants were indeed stressed by the salts, because in salt-free soils virtually all plants would have flowered.

Table S4. Ramping Salt-Stress Between and Within Microbiome-Generations. Soil in each pot was initially hydrated with 102 ml salt-solution [94 ml added to soil prior to autoclaving; 4 ml during planting; and 4 ml during microbiome inoculation in Generations 1-9 (2 ml in Generation 0); see *Planting* and *Inoculation of Seeds*]. In the baseline Generation 0, plants were watered only with unsalted water, but starting with Generation 1, we increased salt-stress *within* each Generation by watering with salted water (details in Table S3). Because we capped pots for the first 4 days to control initial microbiome assembly, we started watering each Generation on Days 5 or 6. Pots of Generations 0-3 were watered more because of low humidity (because of heating of our Greenhouse Facility in winter) and pots of Generations 4&5 were watered less because of high humidity (unusual rainfall in spring, increasing general humidity; see *Growth Chamber*). Pots were watered more in the second half of Generation 9 because plants grew large and transpired more water. Plants of Generations 0-8 were short-cycled to grow only for 20-30 days before microbiome transfer (to about 10-15 leaves for the largest plants); plants in Generation 9 were grown for 68 days to permit ripening of seeds. SOD = sodium-sulfate; ALU = aluminum-sulfate.

	Microbiome-Generation (Selection-Cycle)																			
	Gen 0		Gen 1		Gen 2		Gen 3		Gen 4		Gen 5		Gen 6		Gen 7		Gen 8		Gen 9	
**Initial Soil Hydration (see also Table S3)**	**SOD**	**ALU**	**SOD**	**ALU**	**SOD**	**ALU**	**SOD**	**ALU**	**SOD**	**ALU**	**SOD**	**ALU**	**SOD**	**ALU**	**SOD**	**ALU**	**SOD**	**ALU**	**SOD**	**ALU**
	100 ml<br>20 mM	100 ml<br>0.02 mM	102 ml<br>30 mM	102 ml<br>0.04 mM	102 ml<br>50 mM	102 ml<br>0.08 mM	102 ml<br>60 mM	102 ml<br>0.2 mM	102 ml<br>70 mM	102 ml<br>1.0 mM	102 ml<br>75 mM	102 ml<br>2.0 mM	102 ml<br>60 mM	102 ml<br>1.0 mM	102 ml<br>60 mM	102 ml<br>1.0 mM	102 ml<br>60 mM	102 ml<br>1.5 mM	102 ml<br>60 mM	102 ml<br>1.5 mM
**Watering Schedule**																				
**Day 5**	15ml<br>0 mM	15ml<br>0 mM	15ml<br>0 mM	15ml<br>0 mM	15ml<br>15 mM	15ml<br>0.16 mM	15ml<br>15 mM	15ml<br>0.20 mM	10ml<br>15 mM	10ml<br>3.0 mM	-	-	-	-	15ml<br>15 mM	15ml<br>2.0 mM	20ml<br>15 mM	20ml<br>2.0 mM	20ml<br>15 mM	20ml<br>2.0 mM
**Day 6**	23ml<br>0 mM	23ml<br>0 mM	20ml<br>0 mM	20ml<br>0 mM	25ml<br>15 mM	25ml<br>0.16 mM	-	-	-	-	5ml<br>15 mM	5ml<br>5.0 mM	15ml<br>15 mM	15ml<br>4.0 mM	-	-	-	-	-	-
**Day 7**	-	-	-	-	-	-	25ml<br>15 mM	25ml<br>0.60 mM	25ml<br>15 mM	25ml<br>3.0 mM	-	-	-	-	15ml<br>15 mM	15ml<br>2.0 mM	25ml<br>15 mM	25ml<br>2.0 mM	-	-
**Day 8**	17ml<br>0 mM	17ml<br>0 mM	20ml<br>15 mM	20ml<br>0.02 mM	25ml<br>15 mM	25ml<br>0.16 mM	-	-	-	-	-	-	-	-	-	-	-	-	20ml<br>15 mM	20ml<br>2.0 mM
**Day 9**	-	-	-	-	-	-	25ml<br>15 mM	25ml<br>0.90 mM	-	-	10ml<br>15 mM	10ml<br>10.0 mM	25ml<br>15 mM	25ml<br>4.0 mM	15ml<br>15 mM	15ml<br>2.0 mM	25ml<br>15 mM	25ml<br>2.0 mM	-	-
**Day 10**	25ml<br>0 mM	25ml<br>0 mM	20ml<br>0 mM	20ml<br>0 mM	25ml<br>15 mM	25ml<br>0.24 mM	-	-	-	-	-	-	-	-	-	-	-	-	15ml<br>15 mM	15ml<br>2.0 mM
**Day 11**	-	-	-	-	-	-	25ml<br>15 mM	25ml<br>0.90 mM	25ml<br>15 mM	25ml<br>5.0 mM	-	-	15ml<br>15 mM	15ml<br>4.0 mM	-	-	15ml<br>15 mM	15ml<br>2.0 mM	-	-
**Day 12**	25ml<br>0 mM	25ml<br>0 mM	25ml<br>15mM	25ml<br>0.02 mM	25ml<br>15 mM	25ml<br>0.32 mM	-	-	-	-	-	-	-	-	15ml<br>15 mM	15ml<br>2.0 mM	-	-	-	-
**Day 13**	-	-	-	-	25ml<br>15 mM	25ml<br>0.32 mM	25ml<br>15 mM	25ml<br>1.20 mM	-	-	20ml<br>15 mM	20ml<br>15.0 mM	25ml<br>15 mM	25ml<br>5.0 mM	-	-	15ml<br>15 mM	15ml<br>2.0 mM	20ml<br>15 mM	20ml<br>2.0 mM
**Day 14**	25ml<br>0 mM	25ml<br>0 mM	25ml<br>15mM	25ml<br>0.04 mM	-	-	-	-	-	-	-	-	-	-	20ml<br>15 mM	20ml<br>2.0 mM	-	-	15ml<br>15 mM	15ml<br>2.0 mM
**Day 15**	-	-	-	-	25ml<br>15 mM	25ml<br>0.40 mM	25ml<br>15 mM	25ml<br>1.50 mM	10ml<br>15 mM	10ml<br>5.0 mM	-	-	-	-	-	-	15ml<br>15 mM	15ml<br>2.0 mM	-	-
**Day 16**	25ml<br>0 mM	25ml<br>0 mM	25ml<br>15mM	25ml<br>0.08 mM	-	-	-	-	-	-	-	-	-	-	-	-	-	-	-	-
**Day 17**	-	-	-	-	25ml<br>15 mM	25ml<br>0.60 mM	-	-	-	-	20ml<br>15 mM	20ml<br>15.0 mM	15ml<br>15 mM	15ml<br>5.0 mM	20ml<br>15 mM	20ml<br>2.0 mM	15ml<br>15 mM	15ml<br>2.0 mM	18ml<br>15 mM	18ml<br>2.0 mM
**Day 18**	25ml<br>0 mM	25ml<br>0 mM	25ml<br>15 mM	25ml<br>0.08 mM	-	-	25ml<br>15 mM	25ml<br>1.80 mM	15ml<br>15 mM	15ml<br>5.0 mM	-	-	-	-	-	-	-	-	-	-
**Day 19**	-	-	-	-	25ml<br>15 mM	25ml<br>0.90 mM	-	-	-	-	-	-	-	-	20ml<br>15 mM	20ml<br>2.0 mM	-	-	-	-
**Day 20**	25ml<br>0 mM	25ml<br>0 mM	**Transfer to Gen2**	**Transfer to Gen2**	-	-	25ml<br>15 mM	25ml<br>1.80 mM	-	-	-	-	25ml<br>15 mM	25ml<br>3.0 mM	-	-	20ml<br>15 mM	20ml<br>2.0 mM	18ml<br>15 mM	18ml<br>2.0 mM
**Day 21**	-	-			**Transfer to Gen3**	**Transfer to Gen3**	-	-	20ml<br>15 mM	20ml<br>5.0 mM	25ml<br>15 mM	25ml<br>15.0 mM	-	-	15ml<br>15 mM	15ml<br>2.0 mM	-	-	-	-
**Day 22**	**Transfer to Gen1**	**Transfer to Gen1**					10ml<br>15 mM	10ml<br>1.80 mM	-	-	-	-	-	-	-	-	-	-	-	-
**Day 23**							**Transfer to Gen4**	**Transfer to Gen4**	-	-	-	-	-	-	20ml<br>15 mM	20ml<br>2.0 mM	25ml<br>15 mM	25ml<br>2.0 mM	20ml<br>15 mM	20ml<br>2.0 mM
**Day 24**									10ml<br>15 mM	10ml<br>5.0 mM	-	-	20ml<br>15 mM	20ml<br>3.0 mM	-	-	-	-	-	-
**Day 25**									**Transfer to Gen5**	**Transfer to Gen5**	20ml<br>15 mM	20ml<br>15.0 mM	-	-	20ml<br>15 mM	20ml<br>2.0 mM	-	-	-	-
**Day 26**											-	-	**Transfer to Gen7**	**Transfer to Gen7**	-	-	25ml<br>15 mM	25ml<br>2.0 mM	18ml<br>15 mM	18ml<br>2.0 mM
**Day 27**											-	-			20ml<br>15 mM	20ml<br>2.0 mM	-	-	-	-
**Day 28**											-	-			**Transfer to Gen8**	**Transfer to Gen8**	20ml<br>15 mM	20ml<br>2.0 mM	20ml<br>15 mM	20ml<br>2.0 mM
**Day 29**											20ml<br>15 mM	20ml<br>15.0 mM					-	-	-	-
**Day 30**											-	-					10ml<br>15 mM	10ml<br>2.0 mM	-	-
**Day 31**											10ml<br>15 mM	10ml<br>15.0 mM					**Transfer to Gen9**	**Transfer to Gen9**	20ml<br>15 mM	20ml<br>2.0 mM
**Day 32**											**Transfer to Gen6**	**Transfer to Gen6**							20ml<br>15 mM	20ml<br>2.0 mM
**Day 33**																			-	-
**Day 34**																			20ml<br>15 mM	20ml<br>2.0 mM
**Day 35**																			-	-
**Day 36**																			20ml<br>15 mM	20ml<br>2.0 mM
**Day 37**																			-	-
**Day 38**																			20ml<br>15 mM	20ml<br>2.0 mM
**Day 39**																			20ml<br>15 mM	20ml<br>2.0 mM
**Day 40**																			-	-
**Day 41**																			20ml<br>15 mM	20ml<br>2.0 mM
**Day 42**																			-	-
**Day 43**																			20ml<br>15 mM	20ml<br>2.0 mM
**Day 44**																			-	-
**Day 45**																			-	-
**Day 46**																			20ml<br>15 mM	20ml<br>2.0 mM
**Day 47**																			20ml<br>15 mM	20ml<br>2.0 mM

**Day 48**																			20ml 15 mM	20ml 2.0 mM
**Day 49**																			-	-
**Day 50**																			20ml 15 mM	20ml 2.0 mM
**Day 51**																			-	-
**Day 52**																			20ml 15 mM	20ml 2.0 mM
**Day 53**																			-	-
**Day 54**																			-	-
**Day 55**																			20ml 15 mM	20ml 2.0 mM
**Day 56**																			20ml 15 mM	20ml 2.0 mM
**Day 57**																			20ml 15 mM	20ml 2.0 mM
**Day 58**																			20ml 15 mM	20ml 2.0 mM
**Day 59**																			20ml 15 mM	20ml 2.0 mM
**Day 60**																			-	-
**Day 61**																			20ml 15 mM	20ml 2.0 mM
**Day 62**																			-	-
**Day 63**																			20ml 15 mM	20ml 2.0 mM
**Day 64**																			-	-
**Day 65**																			20ml 15 mM	20ml 2.0 mM
**Day 66**																			-	-
**Day 67**																			20ml 15 mM	20ml 2.0 mM
**Day 68**																			15ml 15 mM	15ml 2.0 mM
**Day 69**																			15ml 15 mM	15ml 2.0 mM
**Day 70**																			**End**	**End**

Visualization of Relative Plant Performance (Figure 2): Because successive generations were not grown under precisely identical conditions (e.g., we had to increase the duration of selection-cycles in later generations because plant-growth decelerated under the increasing salt-stress; we had to adjust watering schedules because of uncontrolled humidity in our growth chamber), we plot in Figure 2 (main article) plant-performance as relative above-ground biomass (rather than absolute biomass), relative to control plants of the same salt treatment. Because we had for each salt treatment (SOD, ALU) two control treatments (Fallow-Soil Control, Null Control), we calculated relative performance of plants with selected microbiomes in two ways for each salt treatment, relative to the average performance (dry above-ground biomass) of Fallow-Soil-Control plants (Figure 2a&b) and relative to average performance (dry above-ground biomass) of Null-Control plants (Figure 2c&d). To calculate average performance of Fallow-Soil-Control plants, we first averaged within each of the 5 Fallow-Soil lines, then averaged across these 5 averages. To calculate average performance of Null-Control plants, because there was only one line of Null-Control plants, average performance could be calculated directly for these plants. The calculations of relative plant-performance for Generations 0-8 appear in Columns V-AE of Table S1. In Generation 9, we allowed plants to grow for 68 days to permit flowering and ripening of seeds; plants and microbiomes of Generation 9 are therefore not comparable to those of Generations 0-8, and we analyzed data from Generation 9 therefore separately (see next; Table S2, Figures 3 & 4).

Crossing Evolved SOD- and ALU-Microbiomes with SOD- and ALU-Stress in Generation 9; Solute-Control in Generation 9: At the end of the experiment in Generation 9, we modified protocols in three important ways: (a) we grew plants for 68 days to permit flowering and ripening of seeds, because seed production seemed a more informative estimator of plant fitness than the proxy of above-ground biomass used in Generations 0-8; and (b) we doubled the total number of pots to 400 (i.e., 400 plants) to permit addition of two control treatments (in addition to Fallow-Soil-Control and Null-Control treatments already used in earlier Generations). We added these two control treatments to understand the mechanistic basis of the salt-tolerance-conferring effects of microbiomes in the SOD and ALU selection lines. The first additional control was *Solute Control* (Figure 3, main text), where we filtered out all live cells from the harvested microbiomes in the selection lines (using a 0.2µm filter; see above *Microbiome-Fractionation with Microfilters*), to test the growth-enhancing effect of viruses and plant-exuded solutes that may be co-harvested from rhizosphere soil together with the bacterial rhizosphere microbiomes propagated in the selection-lines. The second control was 2x2 *Cross-Fostering Control* (Figure 4, main text), where we crossed harvested microbiomes from the SOD and ALU selection lines with the two types of salt stress in

soil (i.e., microbiomes harvested from SOD-selection-lines were tested in both SOD-soil and in ALU-soil; microbiomes harvested from ALU-selection-lines were tested in both SOD-soil and in ALU-soil) to test specificity of the salt-tolerance-conferring effects of the microbiomes. This Cross-Fostering treatment allowed us to address the question whether the salt-tolerance-conferring effects of the *SOD-selected* microbiomes confer these effects only under SOD-stress, or also in ALU-stress; and vice versa the additional question whether the salt-tolerance-conferring effects of the *ALU-selected* microbiomes confer these effects only under SOD-stress, or also in ALU-stress. This basic cross-fostering design was inspired by the experimental methods developed by Lau & Lennon (2012), except that, in contrast to Lau & Lennon (2012), our plant-populations did not evolve because we used seeds from non-evolving stock, and that we artificially selected on microbiomes (whereas in Lau & Lennon the plants evolved under artificial selection on plants, and microbiomes were *not* propagated *differentially* as in Steps 3 & 4 of Figure 1S).

Phenotyping of Plants and Microbiome-Harvesting in the Last Generation 9: In contrast to Generations 0-8 when we used early growth of plants (above-ground biomass during first 3-4 weeks) as host phenotype to select indirectly on microbiomes, in the last Generation 9, we allowed plants to mature for 68 days (10 weeks), such that plants could flower and seeds could ripen. Because of the longer growth, some plants started to senesce towards the end of Generation 9 and some individual flower stalks of some plants started to dry (no plant dried completely by the end of Generation 9). We decided to grow plants to seed in this last Generation because we were interested in understanding how short-cycle microbiome-selection to increase above-ground biomass of young, pre-flowing plants (20-30 days old, when we harvested and transplanted microbiomes in Generations 0-8) would affect flowering and seed-set if plants were allowed to grow older (68 days). Apart from the longer duration of Generation 9 to permit flowering, a second important difference is likely the gradually increasing salt-concentration in soils of Generation 9 that were watered 34 times with salted water over 68 days (Table S4), in contrast to watering with salted water fewer times over the shorter 20-30 days in Generations 0-8 (9-12 waterings, depending on the Generation; see Table S4). At the end of Generation 9, all plants were cut at soil level, above-ground biomass was preserved for each plant in individual envelopes (for drying and later weighing of seeds and overall biomass; see above), and each root systems was extracted from its pot and placed into an autoclaved aluminum-tub for further processing. Root-systems of plants from Generations 0-8 were comparatively small (filling about 30-60% of the soil-volume in each pot), but root-systems at the end of Generation 9 were large and extended through the entire soil-volume in each pot. We shook-off most of the adhering soil from each root system of Generation 9, cut off and discarded the top-most 2cm portion with sterile scissors, then cut the remaining root-system lengthwise (top to bottom) to preserve half of the root-system in 100% ethanol (for metagenomic screens of bacterial communities), whereas we flash-froze (in liquid nitrogen) the other half of the root-system for possible later transcriptomics analyses. For some of the best-growing plants in the SOD- and ALU-selection-lines, we also preserved a representative portion of the root-system in sterile 20% glycerol (for storage at -80°C for possible later isolation of microbes). Processing all root-systems (nearly 400 plants) took considerable time over three successive days (Days 68-70 of Generation 9). Although we processed plants from 3 racks on Day 68 (Racks #3, #8, #7), 3 racks on Day 69 (Racks #6, #2, #4), and 2 racks on Day 70 (Racks #1, #5), to simplify, we label all weight-data of these plants as if collected on Day 68.

References

Aggarwal A, Ezaki B, Munjal A, Tripathi BN. 2015. Physiology and biochemistry of aluminum toxicity and tolerance in crops. Pp 35-57 in *Stress Responses in Plants* (Tripathi BN & Müller M, eds), Springer International Publishing.

Bais HP, Weir TL, Perry LG, Gilroy S, Vivanco JM. 2006. The role of root exudates in rhizosphere interactions with plants and other organisms. *Annu Rev Plant Biol* 57: 233-66. DOI: 10.1146/annurev.arplant.57.032905.105159

Bakken LR, Olsen RA. 1987. The relationship between cell size and viability of soil bacteria. *Microb Ecol* 13: 103-114. DOI: 10.1007/BF02011247

Brkljacic J, Grotewold E, Scholl R, Mockler T, Garvin DF, Vain P, Brutnell T, Sibout R, Bevan M, Budak H, Caicedo A, Gao C, Gu Y, Hazen S, Holt BF, Hong S, Jordan M, Manzaneda AJ, Mitchell-Olds T, Mochida K, Mur LAJ, Park C-M, Sedbrook J, Watt M, Zheng S, Vogel JP. 2011. *Brachypodium* as a model for the grasses: today and the future. *Plant Physiol* 157: 3–13. DOI: 10.1104/pp.111.179531

Bulgarelli D, Schlaeppi K, Spaepen S, van Themaat EVL, Schulze-Lefert P. 2013. Structure and functions of the bacterial microbiota of plants. *Annu Rev Plant Biol* 64: 807–838. DOI: 10.1146/annurev-arplant-050312-120106

Coyte KZ, Schluter J, Foster KR. 2015. The ecology of the microbiome: Networks, competition, and stability. *Science* 350: 663–666. DOI: 10.1126/science.aad2602

Delhaize E, Ryan PR. 1995. Aluminum toxicity and tolerance in plants. *Plant Physiology* 107: 315–321. DOI: 10.1104/pp.107.2.315

Des Marais DL, Juenger TE. 2016. *Brachypodium* and the abiotic environment. Pages 291-311 in *Genetics and Genomics of* Brachypodium (Vogel JP, ed). Springer International Publishing. DOI: 10.1007/7397_2015_13

Des Marais DL, Razzaque S, Hernandez KM, Garvin DF, Juenger TE. 2016. Quantitative trait loci associated with natural diversity in water-use efficiency and response to soil drying in *Brachypodium distachyon*. *Plant Science* 251: 2–11. DOI: 10.1016/j.plantsci.2016.03.010

Dodd IC1, Pérez-Alfocea F. 2012. Microbial amelioration of crop salinity stress. *J Exp Bot* 63: 3415–3428. DOI: 10.1093/jxb/ers033.

Fierer N, Ferrenberg S, Flores GE, González A, Kueneman J, Legg T, Lynch RC, McDonald D, Mihaljevic JR, O'Neill SP, Rhodes ME, Song SJ, Walters WA. 2012. From animalcules to an ecosystem: Application of ecological concepts to the human microbiome. *Annu Rev Ecol Evol Syst* 43: 137–155. DOI: 10.1146/annurev-ecolsys-110411–160307

Garland T, Rose MR. 2009. *Experimental Evolution*. University of California Press.

Garvin DF, Gu YQ, Hasterok R, Hazen SP, Jenkins G, Mockler TC, Mur LAJ, Vogel JP. 2008. Development of genetic and genomic research resources for *Brachypodium distachyon*, a new model system for grass crop research. *Crop Science* 48: S69–S84. DOI: 10.2135/cropsci2007.06.0332tpg

ISO/FDIS 10390 (2005) Soil quality – Determination of pH. International Organization for Standardization, 2005. www.iso.org/iso/catalogue_detail.htm?csnumber=40879 www.ecn.nl/docs/society/horizontal/pH_standard_for_validation.pdf

Lau JA, Lennon JT. 2012. Rapid responses of soil microorganisms improve plant fitness in novel environments. *Proc Natl Acad Sci USA* 109: 14058–14062. DOI: 10.1073/pnas.1202319109

Lodeyro AF, Carrillo N. 2015. Salt stress in higher plants: mechanisms of toxicity and defensive responses. Pp 1-33 in *Stress Responses in Plants* (Tripathi BN & Müller M, eds), Springer International Publishing.

Luef B, Frischkorn KR, Wrighton KC, Holman HY, Birarda G, Thomas BC, Singh A, Williams KH, Siegerist CE, Tringe SG, Downing KH, Comolli LR, Banfield JF. 2015. Diverse uncultivated ultra-small bacterial cells in groundwater. *Nat Commun* 6: 6372. DOI: 10.1038/ncomms7372

Mueller, U.G. Gerardo NM, Aanen DK, Six DL, Schultz TR. 2005. The evolution of agriculture in insects. *Annu Rev Ecol Evol Syst* 36: 563–595. DOI: 10.1146/annurev.ecolsys.36.102003.152626

Mueller UG, Sachs JL. 2015. Engineering microbiomes to improve plant and animal health. *Trends Microbiol* 23: 606–617. DOI: 10.1016/j.tim.2015.07.009

Panke-Buisse K, Poole AC, Goodrich JK, Ley RE, Kao-Kniffin J. 2015. Selection on soil microbiomes reveals reproducible impacts on plant function. *ISME J* 9: 980–989. DOI: 10.1038/ismej.2014.196

Priest HD, Fox SE, Rowley ER, Murray JR, Michael TP, Mockler TC. 2014. Analysis of global gene expression in *Brachypodium distachyon* reveals extensive network plasticity in response to abiotic stress. *PLoS One* 9: e87499. DOI: 10.1371/journal.pone.0087499

Scheuring I, Yu DW. 2012. How to assemble a beneficial microbiome in three easy steps. *Ecol Lett* 15: 1300–1307. DOI: 10.1111/j.1461-0248.2012.01853.x

Swenson W, Wilson DS, Elias R. 2000. Artificial ecosystem selection. *Proc Natl Acad Sci USA* 97: 9110–9114. DOI: 10.1073/pnas.150237597

Vogel JP, Garvin DF, Leong O, Hayden DM 2006. *Agrobacterium*-mediated transformation and inbred line development in the model grass *Brachypodium distachyon*. *Plant Cell, Tissue & Organ Culture* 84: 199-211. DOI: 10.1007/s11240-005-9023-9

Vogel J, Bragg J. 2009. *Brachypodium distachyon*, a new model for the Triticeae. In *Genetics and Genomics of the Triticeae*, pp. 427–449. Edited by GJ Muehlbauer & C Feuillet. New York: Springer.
