# Supplementary material for: Artificial Selection on Microbiomes To Breed Microbiomes That Confer Salt Tolerance to Plants
Source: mSystems. 2021 Nov 30;6(6):e01125-21. doi: 10.1128/mSystems.01125-21 (PMC8631316; doi:10.1128/mSystems.01125-21)
Supplement: TABLE S2 [file msystems.01125-21-st002.pdf]

Supplemental Table S2

Table S2 (Data, Generation 9)

sent to co-authors for analyses 5. Sep. 2016

Abbreviations used in this Table:

"NA" denotes empty cell (e.g., seed that failed to germinate)

"SOD" denotes sodium-sulfate stress, "ALU" denotes aluminum-sulfate stress

"Null" denotes no microbiome-inoculation of a seed, but microbiomes assemble from aerial microbial-rain

Explanation of Columns:

**Column B**	Microbiome-Generation 9 is the final test-generation after 8 rounds of microbiome-propagation from Gen0-Gen8 (see data on Gen0-Gen8 in Tables S1a&b) and then one additional 9th round of microbiome-propagation to plants of Gen9 tested in this spreadsheet
**Column C**	salt treatment of soil (& water used to water plants) <u>PRIOR TO</u> Generation 9 (i.e., selection history during Gen 0-8); this is the salt-stress under which microbiomes evolved
**Column D**	salt treatment of soil (& water used to water plants) <u>DURING</u> Generation 9; this is the salt-stress to test the evolved microbiomes in the final Generation 9;
**"**	selected-microbiomes (Pp) were tested under conditions under which they evolved (e.g., SOD-selected-microbiomes tested in SOD-soil), or under crossed condition (e.g., SOD-selected-microbiomes tested in ALU-soil, ALU-selected-microbiomes tested in SOD-soil)
**Column E**	Pp is "plant present", a microbiome was harvested from rhizosphere of Gen8 plant, filtered with a 2.0µm filter to retain only bacterial microbiomes, then propagated to seeds of Gen9;
**"**	PpFilt is a harvested selected microbiome of Gen8 (i.e., a Pp-microbiome) filtered a second time with a 0.2µm filter to eliminate also bacterial cells, then test on Gen9 seeds this 0.2µm-filtrate contaning solutes (& any viruses) co-harvested with bacterial microbiomes
**"**	Np is "no plant", a microbiome was harvested from fallow soil with no plant in pot, then propagated to seeds of Gen9 to test microbiome effects on plants
**"**	Null is "no microbiome-inoculation of seed"; plants acquire microbiomes from aerial microbial-rain
**Column F**	selection lines in Pp, PpFilt, and Np treatments; for each salt-treatment, there are 5 selection-lines for Pp (and 5 corresponding PpFilt-lines) and 5 lines for Np
**Column G**	plant number used to identify individual plants (i.e., pots); pots were randomly allocated to treatments and replicates;
**"**	Generation 9 had 400 pots total, each planted with a single seed; 20 of these seeds showed no above-ground shoot by end of Day4 and were then removed
**Column H**	rack number; for each selection-line, replicates were blocked evenly between racks; there were 8 racks total, each with 50 pots (8x50 = 400 pots total)
**Column I**	position of pot in rack; pots assigned to a particular rack were randomly assigned to the 50 pot-positions in each rack
**Column I**	total seed dry-weight in milligram (after removing glumes from seeds) on Day 68; seeds were weighed blndly with respect to plant treatment
**Column K**	total above-ground dry-weight in milligram (leaves, stems, inflorescences, seeds) on Day 68; biomass was weighed blndly with respect to plant treatment

###	Generation	Salt Treatment during selection PRIOR TO Gen9	Gen9 Salt Stress	Microbiome Treatment	Selection Line	Plant #	Rack #	Position in Rack	Total Seed Weight	Total Biomass	Line-Code used in Master Spreadsheet	Plant #	Total Seed Weight	Selection Line Average	Treatment Average	Treatment StDev	Total Biomass	Selection Line Average	Treatment Average	Treatment StDev
1	9	SOD	SOD	Pp	1	106	1	12	495.9	965.7	SodPp1xSodSoil	106	495.9				965.7			
2	9	SOD	SOD	Pp	1	196	2	8	329.6	999.5	SodPp1xSodSoil	196	329.6				999.5			
3	9	SOD	SOD	Pp	1	190	3	15	272.5	1239.4	SodPp1xSodSoil	190	272.5				1239.4			
4	9	SOD	SOD	Pp	1	82	4	5	390.9	891.1	SodPp1xSodSoil	82	390.9				891.1			
5	9	SOD	SOD	Pp	1	92	5	19	133.5	1372.6	SodPp1xSodSoil	92	133.5				1372.6			
6	9	SOD	SOD	Pp	1	57	6	28	180.2	1244.5	SodPp1xSodSoil	57	180.2				1244.5			
7	9	SOD	SOD	Pp	1	194	7	20	25.2	1111.4	SodPp1xSodSoil	194	25.2				1111.4			
8	9	SOD	SOD	Pp	1	126	8	11	377.2	839.9	SodPp1xSodSoil	126	377.2	275.6			839.9	1083.0		
9	9	SOD	SOD	Pp	2	5	1	45	342.0	748.2	SodPp2xSodSoil	5	342.0				748.2			
10	9	SOD	SOD	Pp	2	87	2	28	333.5	980.2	SodPp2xSodSoil	87	333.5				980.2			
11	9	SOD	SOD	Pp	2	88	3	32	406.2	959.5	SodPp2xSodSoil	88	406.2				959.5			
12	9	SOD	SOD	Pp	2	90	4	7	0.0	1348.5	SodPp2xSodSoil	90	0.0				1348.5			
13	9	SOD	SOD	Pp	2	155	5	15	453.7	1009.8	SodPp2xSodSoil	155	453.7				1009.8			
14	9	SOD	SOD	Pp	2	135	6	13	406.8	784.2	SodPp2xSodSoil	135	406.8				784.2			
15	9	SOD	SOD	Pp	2	7	7	32	230.8	1114.3	SodPp2xSodSoil	7	230.8				1114.3			
16	9	SOD	SOD	Pp	2	176	8	48	35.9	1163.3	SodPp2xSodSoil	176	35.9	276.1			1163.3	1013.5		
17	9	SOD	SOD	Pp	3	102	1	38	111.2	1286.6	SodPp3xSodSoil	102	111.2				1286.6			
18	9	SOD	SOD	Pp	3	123	2	50	51.3	1251.2	SodPp3xSodSoil	123	51.3				1251.2			
19	9	SOD	SOD	Pp	3	58	3	19	464.3	1047.2	SodPp3xSodSoil	58	464.3				1047.2			
20	9	SOD	SOD	Pp	3	163	4	42	447.3	881.8	SodPp3xSodSoil	163	447.3				881.8			
21	9	SOD	SOD	Pp	3	170	5	24	441.2	1224.7	SodPp3xSodSoil	170	441.2				1224.7			
22	9	SOD	SOD	Pp	3	199	6	37	23.2	1202.6	SodPp3xSodSoil	199	23.2				1202.6			
23	9	SOD	SOD	Pp	3	189	7	11	354.1	944.8	SodPp3xSodSoil	189	354.1				944.8			

Supplemental Table S2

24	9	SOD	SOD	Pp	3	33	8	38	0.0	1214.6	SodPp3xSodSoil	33	0.0	236.6			1214.6	1131.7			
25	9	SOD	SOD	Pp	4	121	1	19	11.5	1322.4	SodPp4xSodSoil	121	11.5				1322.4				
26	9	SOD	SOD	Pp	4	1	2	47	219.3	1198.9	SodPp4xSodSoil	1	219.3				1198.9				
27	9	SOD	SOD	Pp	4	149	3	4	398.2	831.6	SodPp4xSodSoil	149	398.2				831.6				
28	9	SOD	SOD	Pp	4	3	4	19	9.9	1190.4	SodPp4xSodSoil	3	9.9				1190.4				
29	9	SOD	SOD	Pp	4	180	5	2	406.0	1152.8	SodPp4xSodSoil	180	406.0				1152.8				
30	9	SOD	SOD	Pp	4	65	6	10	359.4	802.5	SodPp4xSodSoil	65	359.4				802.5				
31	9	SOD	SOD	Pp	4	73	7	39	0.0	1020.9	SodPp4xSodSoil	73	0.0				1020.9				
32	9	SOD	SOD	Pp	4	130	8	29	361.1	986.3	SodPp4xSodSoil	130	361.1	220.7			986.3	1063.2			
33	9	SOD	SOD	Pp	5	161	1	4	400.5	1169.7	SodPp5xSodSoil	161	400.5				1169.7				
34	9	SOD	SOD	Pp	5	62	2	20	315.7	742.0	SodPp5xSodSoil	62	315.7				742.0				
35	9	SOD	SOD	Pp	5	195	3	42	NA	NA	SodPp5xSodSoil	195	NA				NA				
36	9	SOD	SOD	Pp	5	166	4	49	246.3	1164.5	SodPp5xSodSoil	166	246.3				1164.5				
37	9	SOD	SOD	Pp	5	100	5	34	165.1	1220.6	SodPp5xSodSoil	100	165.1				1220.6				
38	9	SOD	SOD	Pp	5	186	6	4	388.6	974.9	SodPp5xSodSoil	186	388.6				974.9				
39	9	SOD	SOD	Pp	5	69	7	24	359.7	806.6	SodPp5xSodSoil	69	359.7				806.6				
40	9	SOD	SOD	Pp	5	70	8	23	282.3	553.5	SodPp5xSodSoil	70	282.3	308.3	263.5	34.9	553.5	947.4	1047.8	70.3	
41	9	SOD	ALU	Pp	1	289	1	29	123.6	1051.5	SodPp1xAluSoil	289	123.6				1051.5				
42	9	SOD	ALU	Pp	1	330	2	16	284.8	909.9	SodPp1xAluSoil	330	284.8				909.9				
43	9	SOD	ALU	Pp	1	265	3	3	207.9	872.2	SodPp1xAluSoil	265	207.9				872.2				
44	9	SOD	ALU	Pp	1	332	4	4	216.3	1140.6	SodPp1xAluSoil	332	216.3				1140.6				
45	9	SOD	ALU	Pp	1	300	5	6	287.5	1246.3	SodPp1xAluSoil	300	287.5				1246.3				
46	9	SOD	ALU	Pp	1	264	6	19	267.9	965.7	SodPp1xAluSoil	264	267.9				965.7				
47	9	SOD	ALU	Pp	1	393	7	41	0.0	1070.1	SodPp1xAluSoil	393	0.0				1070.1				
48	9	SOD	ALU	Pp	1	266	8	31	34.0	1022.4	SodPp1xAluSoil	266	34.0	177.8			1022.4	1034.8			
49	9	SOD	ALU	Pp	2	232	1	9	266.5	1247.9	SodPp2xAluSoil	232	266.5				1247.9				
50	9	SOD	ALU	Pp	2	348	2	14	241.4	1172.5	SodPp2xAluSoil	348	241.4				1172.5				
51	9	SOD	ALU	Pp	2	378	3	36	84.9	1190.8	SodPp2xAluSoil	378	84.9				1190.8				
52	9	SOD	ALU	Pp	2	279	4	40	0.0	1138.9	SodPp2xAluSoil	279	0.0				1138.9				
53	9	SOD	ALU	Pp	2	328	5	17	112.8	1146.9	SodPp2xAluSoil	328	112.8				1146.9				
54	9	SOD	ALU	Pp	2	205	6	32	107.5	940.7	SodPp2xAluSoil	205	107.5				940.7				
55	9	SOD	ALU	Pp	2	366	7	2	NA	NA	SodPp2xAluSoil	366	NA				NA				
56	9	SOD	ALU	Pp	2	369	8	47	260.8	925.2	SodPp2xAluSoil	369	260.8	153.4			925.2	1109.0			
57	9	SOD	ALU	Pp	3	390	1	48	190.2	1232.6	SodPp3xAluSoil	390	190.2				1232.6				
58	9	SOD	ALU	Pp	3	306	2	11	223.3	1144.2	SodPp3xAluSoil	306	223.3				1144.2				
59	9	SOD	ALU	Pp	3	259	3	40	241.8	925.0	SodPp3xAluSoil	259	241.8				925.0				
60	9	SOD	ALU	Pp	3	295	4	14	58.4	1205.7	SodPp3xAluSoil	295	58.4				1205.7				
61	9	SOD	ALU	Pp	3	271	5	47	154.6	903.6	SodPp3xAluSoil	271	154.6				903.6				
62	9	SOD	ALU	Pp	3	358	6	23	296.5	1098.0	SodPp3xAluSoil	358	296.5				1098.0				
63	9	SOD	ALU	Pp	3	304	7	43	177.0	1082.5	SodPp3xAluSoil	304	177.0				1082.5				
64	9	SOD	ALU	Pp	3	247	8	35	73.3	1207.4	SodPp3xAluSoil	247	73.3	176.9			1207.4	1099.9			
65	9	SOD	ALU	Pp	4	376	1	34	231.0	1103.7	SodPp4xAluSoil	376	231.0				1103.7				
66	9	SOD	ALU	Pp	4	308	2	44	386.1	1230.1	SodPp4xAluSoil	308	386.1				1230.1				
67	9	SOD	ALU	Pp	4	234	3	49	311.2	879.8	SodPp4xAluSoil	234	311.2				879.8				
68	9	SOD	ALU	Pp	4	335	4	18	58.7	1223.2	SodPp4xAluSoil	335	58.7				1223.2				
69	9	SOD	ALU	Pp	4	303	5	36	187.5	1022.2	SodPp4xAluSoil	303	187.5				1022.2				
70	9	SOD	ALU	Pp	4	286	6	1	41.9	1121.1	SodPp4xAluSoil	286	41.9				1121.1				
71	9	SOD	ALU	Pp	4	370	7	13	0.0	995.3	SodPp4xAluSoil	370	0.0				995.3				
72	9	SOD	ALU	Pp	4	386	8	1	220.0	916.7	SodPp4xAluSoil	386	220.0	179.6			916.7	1061.5			
73	9	SOD	ALU	Pp	5	219	1	22	112.0	1171.3	SodPp5xAluSoil	219	112.0				1171.3				
74	9	SOD	ALU	Pp	5	351	2	43	0.0	1106.5	SodPp5xAluSoil	351	0.0				1106.5				
75	9	SOD	ALU	Pp	5	365	3	29	268.1	1268.7	SodPp5xAluSoil	365	268.1				1268.7				
76	9	SOD	ALU	Pp	5	346	4	31	0.0	917.0	SodPp5xAluSoil	346	0.0				917.0				

Supplemental Table S2

77	9	SOD	ALU	Pp	5	254	5	50	85.9	1218.4	SodPp5xAluSoil	254	85.9				1218.4				
78	9	SOD	ALU	Pp	5	349	6	42	54.6	1189.1	SodPp5xAluSoil	349	54.6				1189.1				
79	9	SOD	ALU	Pp	5	311	7	16	0.0	730.7	SodPp5xAluSoil	311	0.0				730.7				
80	9	SOD	ALU	Pp	5	276	8	9	138.4	1142.3	SodPp5xAluSoil	276	138.4	82.4	154.0	41.4	1142.3	1093.0	1079.6	30.8	
81	9	SOD	SOD	PpFilt	1	187	1	36	151.0	978.2	SodPp1Filt2SodSoil	187	151.0				978.2				
82	9	SOD	SOD	PpFilt	1	35	2	1	238.1	560.2	SodPp1Filt2SodSoil	35	238.1				560.2				
83	9	SOD	SOD	PpFilt	1	4	3	28	131.6	1102.0	SodPp1Filt2SodSoil	4	131.6				1102.0				
84	9	SOD	SOD	PpFilt	1	2	4	45	NA	NA	SodPp1Filt2SodSoil	2	NA				NA				
85	9	SOD	SOD	PpFilt	1	174	5	44	0.0	1113.5	SodPp1Filt2SodSoil	174	0.0				1113.5				
86	9	SOD	SOD	PpFilt	1	28	6	14	0.0	913.9	SodPp1Filt2SodSoil	28	0.0				913.9				
87	9	SOD	SOD	PpFilt	1	133	7	23	NA	NA	SodPp1Filt2SodSoil	133	NA				NA				
88	9	SOD	SOD	PpFilt	1	63	8	36	131.8	355.2	SodPp1Filt2SodSoil	63	131.8	108.8			355.2	837.2			
89	9	SOD	SOD	PpFilt	2	9	1	47	168.1	1148.0	SodPp2Filt2SodSoil	9	168.1				1148.0				
90	9	SOD	SOD	PpFilt	2	78	2	49	127.7	1005.8	SodPp2Filt2SodSoil	78	127.7				1005.8				
91	9	SOD	SOD	PpFilt	2	76	3	16	0.0	952.9	SodPp2Filt2SodSoil	76	0.0				952.9				
92	9	SOD	SOD	PpFilt	2	177	4	20	0.0	958.1	SodPp2Filt2SodSoil	177	0.0				958.1				
93	9	SOD	SOD	PpFilt	2	101	5	35	41.5	1158.1	SodPp2Filt2SodSoil	101	41.5				1158.1				
94	9	SOD	SOD	PpFilt	2	129	6	48	9.0	1105.5	SodPp2Filt2SodSoil	129	9.0				1105.5				
95	9	SOD	SOD	PpFilt	2	162	7	29	16.9	910.9	SodPp2Filt2SodSoil	162	16.9				910.9				
96	9	SOD	SOD	PpFilt	2	83	8	10	24.1	992.2	SodPp2Filt2SodSoil	83	24.1	48.4			992.2	1028.9			
97	9	SOD	SOD	PpFilt	3	6	1	28	35.1	1141.2	SodPp3Filt2SodSoil	6	35.1				1141.2				
98	9	SOD	SOD	PpFilt	3	185	2	41	13.7	980.9	SodPp3Filt2SodSoil	185	13.7				980.9				
99	9	SOD	SOD	PpFilt	3	146	3	6	NA	NA	SodPp3Filt2SodSoil	146	NA				NA				
100	9	SOD	SOD	PpFilt	3	168	4	38	250.8	613.9	SodPp3Filt2SodSoil	168	250.8				613.9				
101	9	SOD	SOD	PpFilt	3	95	5	39	88.6	1130.5	SodPp3Filt2SodSoil	95	88.6				1130.5				
102	9	SOD	SOD	PpFilt	3	47	6	50	201.0	872.6	SodPp3Filt2SodSoil	47	201.0				872.6				
103	9	SOD	SOD	PpFilt	3	153	7	7	35.5	1096.4	SodPp3Filt2SodSoil	153	35.5				1096.4				
104	9	SOD	SOD	PpFilt	3	141	8	2	50.2	952.5	SodPp3Filt2SodSoil	141	50.2	96.4			952.5	969.7			
105	9	SOD	SOD	PpFilt	4	144	1	8	NA	NA	SodPp4Filt2SodSoil	144	NA				NA				
106	9	SOD	SOD	PpFilt	4	23	2	7	46.0	983.9	SodPp4Filt2SodSoil	23	46.0				983.9				
107	9	SOD	SOD	PpFilt	4	142	3	30	NA	NA	SodPp4Filt2SodSoil	142	NA				NA				
108	9	SOD	SOD	PpFilt	4	10	4	32	9.5	1021.2	SodPp4Filt2SodSoil	10	9.5				1021.2				
109	9	SOD	SOD	PpFilt	4	152	5	26	4.7	1128.9	SodPp4Filt2SodSoil	152	4.7				1128.9				
110	9	SOD	SOD	PpFilt	4	12	6	39	268.1	1074.5	SodPp4Filt2SodSoil	12	268.1				1074.5				
111	9	SOD	SOD	PpFilt	4	19	7	33	0.0	1010.2	SodPp4Filt2SodSoil	19	0.0				1010.2				
112	9	SOD	SOD	PpFilt	4	116	8	8	21.8	1037.9	SodPp4Filt2SodSoil	116	21.8	58.4			1037.9	1042.8			
113	9	SOD	SOD	PpFilt	5	40	1	49	0.0	1237.1	SodPp5Filt2SodSoil	40	0.0				1237.1				
114	9	SOD	SOD	PpFilt	5	84	2	45	231.1	659.6	SodPp5Filt2SodSoil	84	231.1				659.6				
115	9	SOD	SOD	PpFilt	5	108	3	33	149.0	923.5	SodPp5Filt2SodSoil	108	149.0				923.5				
116	9	SOD	SOD	PpFilt	5	26	4	39	284.9	813.8	SodPp5Filt2SodSoil	26	284.9				813.8				
117	9	SOD	SOD	PpFilt	5	132	5	13	279.5	905.4	SodPp5Filt2SodSoil	132	279.5				905.4				
118	9	SOD	SOD	PpFilt	5	72	6	44	1.7	1104.2	SodPp5Filt2SodSoil	72	1.7				1104.2				
119	9	SOD	SOD	PpFilt	5	80	7	45	8.4	1095.1	SodPp5Filt2SodSoil	80	8.4				1095.1				
120	9	SOD	SOD	PpFilt	5	172	8	42	0.0	1035.4	SodPp5Filt2SodSoil	172	0.0	119.3	86.3	31.3	1035.4	971.8	970.1	81.3	
121	9	SOD	SOD	Np	1	109	1	6	18.6	1328.0	SodNp1	109	18.6				1328.0				
122	9	SOD	SOD	Np	1	44	2	39	0.0	1053.3	SodNp1	44	0.0				1053.3				
123	9	SOD	SOD	Np	1	113	3	23	16.4	948.8	SodNp1	113	16.4				948.8				
124	9	SOD	SOD	Np	1	197	4	21	NA	NA	SodNp1	197	NA				NA				
125	9	SOD	SOD	Np	1	140	5	22	0.0	3.8	SodNp1	140	0.0				3.8				
126	9	SOD	SOD	Np	1	21	6	17	59.2	1179.3	SodNp1	21	59.2				1179.3				
127	9	SOD	SOD	Np	1	45	7	28	176.3	1063.2	SodNp1	45	176.3				1063.2				
128	9	SOD	SOD	Np	1	145	8	33	0.0	1162.8	SodNp1	145	0.0	38.6			1162.8	962.7			
129	9	SOD	SOD	Np	2	158	1	3	NA	NA	SodNp2	158	NA				NA				

Supplemental Table S2

130	9	SOD	SOD	Np	2	25	2	13	316.0	1113.6	SodNp2	25	316.0				1113.6				
131	9	SOD	SOD	Np	2	24	3	34	19.3	1181.1	SodNp2	24	19.3				1181.1				
132	9	SOD	SOD	Np	2	49	4	36	114.0	1237.8	SodNp2	49	114.0				1237.8				
133	9	SOD	SOD	Np	2	66	5	46	54.3	1310.5	SodNp2	66	54.3				1310.5				
134	9	SOD	SOD	Np	2	75	6	18	0.0	2.0	SodNp2	75	0.0				2.0				
135	9	SOD	SOD	Np	2	193	7	17	357.3	1008.4	SodNp2	193	357.3				1008.4				
136	9	SOD	SOD	Np	2	156	8	7	200.1	715.7	SodNp2	156	200.1	151.6			715.7	938.4			
137	9	SOD	SOD	Np	3	183	1	25	383.7	942.7	SodNp3	183	383.7				942.7				
138	9	SOD	SOD	Np	3	198	2	34	8.7	1332.5	SodNp3	198	8.7				1332.5				
139	9	SOD	SOD	Np	3	148	3	27	0.0	3.3	SodNp3	148	0.0				3.3				
140	9	SOD	SOD	Np	3	182	4	9	288.0	566.5	SodNp3	182	288.0				566.5				
141	9	SOD	SOD	Np	3	64	5	37	270.5	796.3	SodNp3	64	270.5				796.3				
142	9	SOD	SOD	Np	3	192	6	6	0.0	2.5	SodNp3	192	0.0				2.5				
143	9	SOD	SOD	Np	3	120	7	21	198.7	672.4	SodNp3	120	198.7				672.4				
144	9	SOD	SOD	Np	3	188	8	25	328.0	913.6	SodNp3	188	328.0	184.7			913.6	653.7			
145	9	SOD	SOD	Np	4	16	1	23	188.5	589.1	SodNp4	16	188.5				589.1				
146	9	SOD	SOD	Np	4	13	2	27	39.4	1340.0	SodNp4	13	39.4				1340.0				
147	9	SOD	SOD	Np	4	55	3	1	0.0	1017.8	SodNp4	55	0.0				1017.8				
148	9	SOD	SOD	Np	4	37	4	12	243.0	748.9	SodNp4	37	243.0				748.9				
149	9	SOD	SOD	Np	4	137	5	49	NA	NA	SodNp4	137	NA				NA				
150	9	SOD	SOD	Np	4	128	6	21	266.7	1026.0	SodNp4	128	266.7				1026.0				
151	9	SOD	SOD	Np	4	119	7	48	52.7	1158.2	SodNp4	119	52.7				1158.2				
152	9	SOD	SOD	Np	4	53	8	50	146.6	1078.2	SodNp4	53	146.6	133.8			1078.2	994.0			
153	9	SOD	SOD	Np	5	67	1	16	53.8	1272.2	SodNp5	67	53.8				1272.2				
154	9	SOD	SOD	Np	5	79	2	6	124.2	1218.8	SodNp5	79	124.2				1218.8				
155	9	SOD	SOD	Np	5	173	3	48	49.2	869.4	SodNp5	173	49.2				869.4				
156	9	SOD	SOD	Np	5	150	4	34	NA	NA	SodNp5	150	NA				NA				
157	9	SOD	SOD	Np	5	68	5	29	28.3	1193.7	SodNp5	68	28.3				1193.7				
158	9	SOD	SOD	Np	5	122	6	22	63.4	1034.4	SodNp5	122	63.4				1034.4				
159	9	SOD	SOD	Np	5	74	7	6	61.0	1313.4	SodNp5	74	61.0				1313.4				
160	9	SOD	SOD	Np	5	112	8	13	246.3	1065.1	SodNp5	112	246.3	89.5	119.6	56.8	1065.1	1138.1	937.4	176.5	
161	9	SOD	SOD	Null	NA	127	1	21	88.5	1049.5	SodNull	127	88.5				1049.5				
162	9	SOD	SOD	Null	NA	50	2	19	293.6	797.6	SodNull	50	293.6				797.6				
163	9	SOD	SOD	Null	NA	110	3	8	47.0	1125.6	SodNull	110	47.0				1125.6				
164	9	SOD	SOD	Null	NA	86	4	50	269.1	950.2	SodNull	86	269.1				950.2				
165	9	SOD	SOD	Null	NA	30	5	21	28.2	1115.3	SodNull	30	28.2				1115.3				
166	9	SOD	SOD	Null	NA	91	6	2	44.6	1095.2	SodNull	91	44.6				1095.2				
167	9	SOD	SOD	Null	NA	11	7	44	0.0	919.0	SodNull	11	0.0				919.0				
168	9	SOD	SOD	Null	NA	115	8	16	33.0	1129.5	SodNull	115	33.0				1129.5				
169	9	SOD	SOD	Null	NA	41	1	35	0.0	1151.3	SodNull	41	0.0				1151.3				
170	9	SOD	SOD	Null	NA	96	2	26	41.7	1063.4	SodNull	96	41.7				1063.4				
171	9	SOD	SOD	Null	NA	184	3	14	0.0	970.8	SodNull	184	0.0				970.8				
172	9	SOD	SOD	Null	NA	31	4	27	149.9	1037.6	SodNull	31	149.9				1037.6				
173	9	SOD	SOD	Null	NA	103	5	42	278.8	934.6	SodNull	103	278.8				934.6				
174	9	SOD	SOD	Null	NA	107	6	11	166.5	992.4	SodNull	107	166.5				992.4				
175	9	SOD	SOD	Null	NA	124	7	3	144.7	1101.5	SodNull	124	144.7				1101.5				
176	9	SOD	SOD	Null	NA	111	8	22	232.8	947.6	SodNull	111	232.8				947.6				
177	9	SOD	SOD	Null	NA	178	1	30	0.0	1115.6	SodNull	178	0.0				1115.6				
178	9	SOD	SOD	Null	NA	32	2	12	84.0	1022.7	SodNull	32	84.0				1022.7				
179	9	SOD	SOD	Null	NA	42	3	37	66.5	1176.6	SodNull	42	66.5				1176.6				
180	9	SOD	SOD	Null	NA	71	4	22	22.9	1107.1	SodNull	71	22.9				1107.1				
181	9	SOD	SOD	Null	NA	54	5	1	27.1	1046.8	SodNull	54	27.1				1046.8				
182	9	SOD	SOD	Null	NA	147	6	47	0.0	1024.5	SodNull	147	0.0				1024.5				

Supplemental Table S2

183	9	SOD	SOD	Null	NA	143	7	26	0.0	1034.6	SodNull	143	0.0				1034.6				
184	9	SOD	SOD	Null	NA	97	8	30	103.6	999.3	SodNull	97	103.6				999.3				
185	9	SOD	SOD	Null	NA	43	1	5	162.1	1032.1	SodNull	43	162.1				1032.1				
186	9	SOD	SOD	Null	NA	151	2	21	0.0	897.1	SodNull	151	0.0				897.1				
187	9	SOD	SOD	Null	NA	160	3	43	85.6	1095.1	SodNull	160	85.6				1095.1				
188	9	SOD	SOD	Null	NA	134	4	8	NA	NA	SodNull	134	NA				NA				
189	9	SOD	SOD	Null	NA	22	5	28	114.1	1047.8	SodNull	22	114.1				1047.8				
190	9	SOD	SOD	Null	NA	179	6	26	233.4	1070.1	SodNull	179	233.4				1070.1				
191	9	SOD	SOD	Null	NA	39	7	42	8.3	1074.9	SodNull	39	8.3				1074.9				
192	9	SOD	SOD	Null	NA	138	8	20	132.9	960.3	SodNull	138	132.9				960.3				
193	9	SOD	SOD	Null	NA	20	1	41	121.7	1087.0	SodNull	20	121.7				1087.0				
194	9	SOD	SOD	Null	NA	18	2	23	198.3	472.8	SodNull	18	198.3				472.8				
195	9	SOD	SOD	Null	NA	171	3	21	59.2	955.1	SodNull	171	59.2				955.1				
196	9	SOD	SOD	Null	NA	17	4	35	0.0	1062.8	SodNull	17	0.0				1062.8				
197	9	SOD	SOD	Null	NA	191	5	9	225.8	1038.1	SodNull	191	225.8				1038.1				
198	9	SOD	SOD	Null	NA	165	6	24	0.0	1084.7	SodNull	165	0.0				1084.7				
199	9	SOD	SOD	Null	NA	81	7	4	41.5	1006.9	SodNull	81	41.5				1006.9				
200	9	SOD	SOD	Null	NA	48	8	34	322.5	943.1	SodNull	48	322.5	98.2	98.2	97.1	943.1	1018.9	1018.9	119.1	
201	9	ALU	ALU	Pp	1	322	1	39	223.1	1242.5	AluPp1xAluSoil	322	223.1				1242.5				
202	9	ALU	ALU	Pp	1	297	2	18	350.1	1193.2	AluPp1xAluSoil	297	350.1				1193.2				
203	9	ALU	ALU	Pp	1	317	3	26	0.0	1069.0	AluPp1xAluSoil	317	0.0				1069.0				
204	9	ALU	ALU	Pp	1	398	4	24	272.2	1177.5	AluPp1xAluSoil	398	272.2				1177.5				
205	9	ALU	ALU	Pp	1	294	5	4	146.6	1324.4	AluPp1xAluSoil	294	146.6				1324.4				
206	9	ALU	ALU	Pp	1	241	6	31	283.3	1044.8	AluPp1xAluSoil	241	283.3				1044.8				
207	9	ALU	ALU	Pp	1	252	7	27	258.5	1040.3	AluPp1xAluSoil	252	258.5				1040.3				
208	9	ALU	ALU	Pp	1	250	8	46	263.4	1108.7	AluPp1xAluSoil	250	263.4	224.7			1108.7	1150.1			
209	9	ALU	ALU	Pp	2	212	1	50	NA	NA	AluPp2xAluSoil	212	NA				NA				
210	9	ALU	ALU	Pp	2	363	2	46	26.9	1133.0	AluPp2xAluSoil	363	26.9				1133.0				
211	9	ALU	ALU	Pp	2	272	3	9	352.6	983.6	AluPp2xAluSoil	272	352.6				983.6				
212	9	ALU	ALU	Pp	2	345	4	26	240.2	1195.2	AluPp2xAluSoil	345	240.2				1195.2				
213	9	ALU	ALU	Pp	2	261	5	10	327.7	1074.5	AluPp2xAluSoil	261	327.7				1074.5				
214	9	ALU	ALU	Pp	2	224	6	16	490.0	1075.5	AluPp2xAluSoil	224	490.0				1075.5				
215	9	ALU	ALU	Pp	2	360	7	30	302.4	946.8	AluPp2xAluSoil	360	302.4				946.8				
216	9	ALU	ALU	Pp	2	270	8	40	103.2	1089.2	AluPp2xAluSoil	270	103.2	263.3			1089.2	1071.1			
217	9	ALU	ALU	Pp	3	284	1	2	43.4	1168.6	AluPp3xAluSoil	284	43.4				1168.6				
218	9	ALU	ALU	Pp	3	269	2	17	126.4	988.1	AluPp3xAluSoil	269	126.4				988.1				
219	9	ALU	ALU	Pp	3	394	3	20	242.6	1048.9	AluPp3xAluSoil	394	242.6				1048.9				
220	9	ALU	ALU	Pp	3	395	4	28	284.6	1161.2	AluPp3xAluSoil	395	284.6				1161.2				
221	9	ALU	ALU	Pp	3	288	5	3	0.0	1132.4	AluPp3xAluSoil	288	0.0				1132.4				
222	9	ALU	ALU	Pp	3	384	6	20	25.0	1155.7	AluPp3xAluSoil	384	25.0				1155.7				
223	9	ALU	ALU	Pp	3	337	7	12	253.1	992.1	AluPp3xAluSoil	337	253.1				992.1				
224	9	ALU	ALU	Pp	3	316	8	32	122.8	1129.7	AluPp3xAluSoil	316	122.8	137.2			1129.7	1097.1			
225	9	ALU	ALU	Pp	4	290	1	7	50.9	1160.6	AluPp4xAluSoil	290	50.9				1160.6				
226	9	ALU	ALU	Pp	4	251	2	3	329.5	1071.8	AluPp4xAluSoil	251	329.5				1071.8				
227	9	ALU	ALU	Pp	4	275	3	5	406.8	1218.9	AluPp4xAluSoil	275	406.8				1218.9				
228	9	ALU	ALU	Pp	4	268	4	30	295.4	1062.5	AluPp4xAluSoil	268	295.4				1062.5				
229	9	ALU	ALU	Pp	4	201	5	11	NA	NA	AluPp4xAluSoil	201	NA				NA				
230	9	ALU	ALU	Pp	4	258	6	35	29.7	1222.3	AluPp4xAluSoil	258	29.7				1222.3				
231	9	ALU	ALU	Pp	4	218	7	35	22.1	1023.8	AluPp4xAluSoil	218	22.1				1023.8				
232	9	ALU	ALU	Pp	4	333	8	44	37.5	1142.0	AluPp4xAluSoil	333	37.5	167.4			1142.0	1128.8			
233	9	ALU	ALU	Pp	5	371	1	20	301.7	1134.4	AluPp5xAluSoil	371	301.7				1134.4				
234	9	ALU	ALU	Pp	5	326	2	4	358.9	970.3	AluPp5xAluSoil	326	358.9				970.3				
235	9	ALU	ALU	Pp	5	302	3	25	114.7	1011.2	AluPp5xAluSoil	302	114.7				1011.2				

Supplemental Table S2

236	9	ALU	ALU	Pp	5	387	4	37	230.4	1184.2	AluPp5xAluSoil	387	230.4				1184.2				
237	9	ALU	ALU	Pp	5	239	5	33	240.4	1159.8	AluPp5xAluSoil	239	240.4				1159.8				
238	9	ALU	ALU	Pp	5	206	6	34	282.2	994.9	AluPp5xAluSoil	206	282.2				994.9				
239	9	ALU	ALU	Pp	5	262	7	9	141.2	1170.4	AluPp5xAluSoil	262	141.2				1170.4				
240	9	ALU	ALU	Pp	5	204	8	28	372.2	872.5	AluPp5xAluSoil	204	372.2	255.2	209.6	55.2	872.5	1062.2	1101.9	37.4	
241	9	ALU	SOD	Pp	1	52	1	40	178.5	1309.8	AluPp1xSodSoil	52	178.5				1309.8				
242	9	ALU	SOD	Pp	1	36	2	38	NA	NA	AluPp1xSodSoil	36	NA				NA				
243	9	ALU	SOD	Pp	1	117	3	50	0.0	1143.7	AluPp1xSodSoil	117	0.0				1143.7				
244	9	ALU	SOD	Pp	1	157	4	13	436.4	1026.8	AluPp1xSodSoil	157	436.4				1026.8				
245	9	ALU	SOD	Pp	1	77	5	41	285.2	1054.8	AluPp1xSodSoil	77	285.2				1054.8				
246	9	ALU	SOD	Pp	1	98	6	30	0.0	1015.0	AluPp1xSodSoil	98	0.0				1015.0				
247	9	ALU	SOD	Pp	1	15	7	49	187.5	1074.8	AluPp1xSodSoil	15	187.5				1074.8				
248	9	ALU	SOD	Pp	1	164	8	45	242.9	639.4	AluPp1xSodSoil	164	242.9	190.1			639.4	1037.8			
249	9	ALU	SOD	Pp	2	94	1	27	0.0	1156.9	AluPp2xSodSoil	94	0.0				1156.9				
250	9	ALU	SOD	Pp	2	38	2	36	NA	NA	AluPp2xSodSoil	38	NA				NA				
251	9	ALU	SOD	Pp	2	14	3	13	302.6	943.5	AluPp2xSodSoil	14	302.6				943.5				
252	9	ALU	SOD	Pp	2	181	4	16	0.0	1214.2	AluPp2xSodSoil	181	0.0				1214.2				
253	9	ALU	SOD	Pp	2	200	5	30	49.3	1251.0	AluPp2xSodSoil	200	49.3				1251.0				
254	9	ALU	SOD	Pp	2	93	6	33	274.8	1015.8	AluPp2xSodSoil	93	274.8				1015.8				
255	9	ALU	SOD	Pp	2	34	7	25	NA	NA	AluPp2xSodSoil	34	NA				NA				
256	9	ALU	SOD	Pp	2	59	8	39	161.2	1089.1	AluPp2xSodSoil	59	161.2	131.3			1089.1	1111.8			
257	9	ALU	SOD	Pp	3	136	1	42	53.8	1292.7	AluPp3xSodSoil	136	53.8				1292.7				
258	9	ALU	SOD	Pp	3	46	2	25	0.0	1152.2	AluPp3xSodSoil	46	0.0				1152.2				
259	9	ALU	SOD	Pp	3	125	3	31	162.7	940.0	AluPp3xSodSoil	125	162.7				940.0				
260	9	ALU	SOD	Pp	3	51	4	43	369.1	1073.9	AluPp3xSodSoil	51	369.1				1073.9				
261	9	ALU	SOD	Pp	3	169	5	7	NA	NA	AluPp3xSodSoil	169	NA				NA				
262	9	ALU	SOD	Pp	3	159	6	15	367.6	949.8	AluPp3xSodSoil	159	367.6				949.8				
263	9	ALU	SOD	Pp	3	105	7	8	0.0	1111.5	AluPp3xSodSoil	105	0.0				1111.5				
264	9	ALU	SOD	Pp	3	104	8	3	222.6	1103.9	AluPp3xSodSoil	104	222.6	168.0			1103.9	1089.1			
265	9	ALU	SOD	Pp	4	61	1	14	37.1	1280.2	AluPp4xSodSoil	61	37.1				1280.2				
266	9	ALU	SOD	Pp	4	114	2	15	376.3	1009.6	AluPp4xSodSoil	114	376.3				1009.6				
267	9	ALU	SOD	Pp	4	118	3	46	291.2	660.1	AluPp4xSodSoil	118	291.2				660.1				
268	9	ALU	SOD	Pp	4	8	4	1	251.4	1162.5	AluPp4xSodSoil	8	251.4				1162.5				
269	9	ALU	SOD	Pp	4	99	5	48	230.7	462.2	AluPp4xSodSoil	99	230.7				462.2				
270	9	ALU	SOD	Pp	4	89	6	41	356.8	831.8	AluPp4xSodSoil	89	356.8				831.8				
271	9	ALU	SOD	Pp	4	154	7	50	193.6	1027.6	AluPp4xSodSoil	154	193.6				1027.6				
272	9	ALU	SOD	Pp	4	85	8	18	358.9	968.2	AluPp4xSodSoil	85	358.9	262.0			968.2	925.3			
273	9	ALU	SOD	Pp	5	175	1	33	103.6	1231.6	AluPp5xSodSoil	175	103.6				1231.6				
274	9	ALU	SOD	Pp	5	27	2	5	346.6	852.8	AluPp5xSodSoil	27	346.6				852.8				
275	9	ALU	SOD	Pp	5	29	3	38	331.6	939.4	AluPp5xSodSoil	29	331.6				939.4				
276	9	ALU	SOD	Pp	5	139	4	15	309.5	778.1	AluPp5xSodSoil	139	309.5				778.1				
277	9	ALU	SOD	Pp	5	167	5	43	349.6	1026.9	AluPp5xSodSoil	167	349.6				1026.9				
278	9	ALU	SOD	Pp	5	131	6	36	330.7	1010.9	AluPp5xSodSoil	131	330.7				1010.9				
279	9	ALU	SOD	Pp	5	60	7	15	219.4	605.7	AluPp5xSodSoil	60	219.4				605.7				
280	9	ALU	SOD	Pp	5	56	8	21	0.0	964.6	AluPp5xSodSoil	56	0.0	248.9	200.0	54.9	964.6	926.3	1018.0	88.4	
281	9	ALU	ALU	PpFilt	1	209	1	44	41.6	1111.7	AluPp1Filt2AluSoil	209	41.6				1111.7				
282	9	ALU	ALU	PpFilt	1	374	2	31	120.7	989.9	AluPp1Filt2AluSoil	374	120.7				989.9				
283	9	ALU	ALU	PpFilt	1	222	3	17	234.2	446.9	AluPp1Filt2AluSoil	222	234.2				446.9				
284	9	ALU	ALU	PpFilt	1	334	4	23	205.3	499.5	AluPp1Filt2AluSoil	334	205.3				499.5				
285	9	ALU	ALU	PpFilt	1	362	5	8	146.7	1154.7	AluPp1Filt2AluSoil	362	146.7				1154.7				
286	9	ALU	ALU	PpFilt	1	400	6	46	NA	NA	AluPp1Filt2AluSoil	400	NA				NA				
287	9	ALU	ALU	PpFilt	1	359	7	40	51.3	1037.0	AluPp1Filt2AluSoil	359	51.3				1037.0				
288	9	ALU	ALU	PpFilt	1	310	8	49	42.6	943.7	AluPp1Filt2AluSoil	310	42.6	120.3			943.7	883.3			

Supplemental Table S2

289	9	ALU	ALU	PpFilt	2	375	1	13	185.4	984.7	AluPp2Filt2AluSoil	375	185.4				984.7				
290	9	ALU	ALU	PpFilt	2	208	2	32	180.9	1083.8	AluPp2Filt2AluSoil	208	180.9				1083.8				
291	9	ALU	ALU	PpFilt	2	312	3	18	303.4	958.3	AluPp2Filt2AluSoil	312	303.4				958.3				
292	9	ALU	ALU	PpFilt	2	318	4	10	118.2	1136.2	AluPp2Filt2AluSoil	318	118.2				1136.2				
293	9	ALU	ALU	PpFilt	2	229	5	14	72.7	1168.9	AluPp2Filt2AluSoil	229	72.7				1168.9				
294	9	ALU	ALU	PpFilt	2	243	6	45	0.0	1027.4	AluPp2Filt2AluSoil	243	0.0				1027.4				
295	9	ALU	ALU	PpFilt	2	211	7	14	119.5	1071.5	AluPp2Filt2AluSoil	211	119.5				1071.5				
296	9	ALU	ALU	PpFilt	2	253	8	27	179.3	1107.9	AluPp2Filt2AluSoil	253	179.3	144.9			1107.9	1067.3			
297	9	ALU	ALU	PpFilt	3	307	1	1	75.0	1083.7	AluPp3Filt2AluSoil	307	75.0				1083.7				
298	9	ALU	ALU	PpFilt	3	327	2	24	96.2	1068.8	AluPp3Filt2AluSoil	327	96.2				1068.8				
299	9	ALU	ALU	PpFilt	3	321	3	7	239.1	661.3	AluPp3Filt2AluSoil	321	239.1				661.3				
300	9	ALU	ALU	PpFilt	3	255	4	46	90.5	1050.3	AluPp3Filt2AluSoil	255	90.5				1050.3				
301	9	ALU	ALU	PpFilt	3	256	5	45	128.1	938.7	AluPp3Filt2AluSoil	256	128.1				938.7				
302	9	ALU	ALU	PpFilt	3	329	6	12	23.8	954.6	AluPp3Filt2AluSoil	329	23.8				954.6				
303	9	ALU	ALU	PpFilt	3	305	7	22	143.7	1090.9	AluPp3Filt2AluSoil	305	143.7				1090.9				
304	9	ALU	ALU	PpFilt	3	223	8	15	198.0	1118.2	AluPp3Filt2AluSoil	223	198.0	124.3			1118.2	995.8			
305	9	ALU	ALU	PpFilt	4	356	1	46	210.9	928.7	AluPp4Filt2AluSoil	356	210.9				928.7				
306	9	ALU	ALU	PpFilt	4	338	2	29	163.0	1015.7	AluPp4Filt2AluSoil	338	163.0				1015.7				
307	9	ALU	ALU	PpFilt	4	203	3	45	208.7	908.6	AluPp4Filt2AluSoil	203	208.7				908.6				
308	9	ALU	ALU	PpFilt	4	215	4	33	121.4	1178.0	AluPp4Filt2AluSoil	215	121.4				1178.0				
309	9	ALU	ALU	PpFilt	4	299	5	27	85.2	1101.7	AluPp4Filt2AluSoil	299	85.2				1101.7				
310	9	ALU	ALU	PpFilt	4	361	6	27	248.6	1005.4	AluPp4Filt2AluSoil	361	248.6				1005.4				
311	9	ALU	ALU	PpFilt	4	396	7	31	0.0	792.2	AluPp4Filt2AluSoil	396	0.0				792.2				
312	9	ALU	ALU	PpFilt	4	331	8	17	0.0	1034.4	AluPp4Filt2AluSoil	331	0.0	129.7			1034.4	995.6			
313	9	ALU	ALU	PpFilt	5	291	1	17	278.6	1076.6	AluPp5Filt2AluSoil	291	278.6				1076.6				
314	9	ALU	ALU	PpFilt	5	277	2	48	84.3	1054.2	AluPp5Filt2AluSoil	277	84.3				1054.2				
315	9	ALU	ALU	PpFilt	5	240	3	39	240.3	483.1	AluPp5Filt2AluSoil	240	240.3				483.1				
316	9	ALU	ALU	PpFilt	5	399	4	2	40.2	1101.7	AluPp5Filt2AluSoil	399	40.2				1101.7				
317	9	ALU	ALU	PpFilt	5	382	5	16	165.7	530.9	AluPp5Filt2AluSoil	382	165.7				530.9				
318	9	ALU	ALU	PpFilt	5	238	6	40	123.9	945.7	AluPp5Filt2AluSoil	238	123.9				945.7				
319	9	ALU	ALU	PpFilt	5	237	7	36	174.2	345.2	AluPp5Filt2AluSoil	237	174.2				345.2				
320	9	ALU	ALU	PpFilt	5	350	8	41	136.2	912.0	AluPp5Filt2AluSoil	350	136.2	155.4	134.9	14.8	912.0	806.2	949.7	103.8	
321	9	ALU	ALU	Np	1	230	1	18	99.5	1058.4	AluNp1	230	99.5				1058.4				
322	9	ALU	ALU	Np	1	319	2	35	0.0	748.2	AluNp1	319	0.0				748.2				
323	9	ALU	ALU	Np	1	397	3	47	0.0	1119.6	AluNp1	397	0.0				1119.6				
324	9	ALU	ALU	Np	1	245	4	29	20.9	1470.8	AluNp1	245	20.9				1470.8				
325	9	ALU	ALU	Np	1	257	5	12	14.1	1133.0	AluNp1	257	14.1				1133.0				
326	9	ALU	ALU	Np	1	244	6	5	22.9	1201.0	AluNp1	244	22.9				1201.0				
327	9	ALU	ALU	Np	1	354	7	5	28.0	1141.7	AluNp1	354	28.0				1141.7				
328	9	ALU	ALU	Np	1	228	8	43	0.0	1110.3	AluNp1	228	0.0	23.2			1110.3	1122.9			
329	9	ALU	ALU	Np	2	217	1	24	38.5	1061.8	AluNp2	217	38.5				1061.8				
330	9	ALU	ALU	Np	2	355	2	42	14.8	1010.9	AluNp2	355	14.8				1010.9				
331	9	ALU	ALU	Np	2	383	3	10	100.3	1168.7	AluNp2	383	100.3				1168.7				
332	9	ALU	ALU	Np	2	202	4	17	NA	NA	AluNp2	202	NA				NA				
333	9	ALU	ALU	Np	2	301	5	32	222.6	775.8	AluNp2	301	222.6				775.8				
334	9	ALU	ALU	Np	2	248	6	8	55.3	1036.7	AluNp2	248	55.3				1036.7				
335	9	ALU	ALU	Np	2	267	7	38	113.2	1109.1	AluNp2	267	113.2				1109.1				
336	9	ALU	ALU	Np	2	314	8	6	273.7	846.5	AluNp2	314	273.7	116.9			846.5	1001.4			
337	9	ALU	ALU	Np	3	324	1	15	94.2	1098.5	AluNp3	324	94.2				1098.5				
338	9	ALU	ALU	Np	3	292	2	33	24.0	986.0	AluNp3	292	24.0				986.0				
339	9	ALU	ALU	Np	3	368	3	12	0.0	902.1	AluNp3	368	0.0				902.1				
340	9	ALU	ALU	Np	3	352	4	41	79.5	1093.9	AluNp3	352	79.5				1093.9				
341	9	ALU	ALU	Np	3	372	5	5	112.7	245.3	AluNp3	372	112.7				245.3				

Supplemental Table S2

342	9	ALU	ALU	Np	3	339	6	49	18.6	1143.0	AluNp3	339	18.6				1143.0				
343	9	ALU	ALU	Np	3	274	7	19	147.5	1024.5	AluNp3	274	147.5				1024.5				
344	9	ALU	ALU	Np	3	320	8	24	NA	NA	AluNp3	320	NA	68.1			NA	927.6			
345	9	ALU	ALU	Np	4	315	1	32	103.9	1044.2	AluNp4	315	103.9				1044.2				
346	9	ALU	ALU	Np	4	380	2	22	219.9	1155.3	AluNp4	380	219.9				1155.3				
347	9	ALU	ALU	Np	4	227	3	41	38.4	984.5	AluNp4	227	38.4				984.5				
348	9	ALU	ALU	Np	4	389	4	6	0.0	1158.0	AluNp4	389	0.0				1158.0				
349	9	ALU	ALU	Np	4	341	5	20	0.0	1116.3	AluNp4	341	0.0				1116.3				
350	9	ALU	ALU	Np	4	385	6	38	0.0	941.2	AluNp4	385	0.0				941.2				
351	9	ALU	ALU	Np	4	226	7	47	72.9	1056.8	AluNp4	226	72.9				1056.8				
352	9	ALU	ALU	Np	4	225	8	12	91.5	1038.9	AluNp4	225	91.5	65.8			1038.9	1061.9			
353	9	ALU	ALU	Np	5	296	1	31	0.0	1066.0	AluNp5	296	0.0				1066.0				
354	9	ALU	ALU	Np	5	379	2	10	63.6	1133.6	AluNp5	379	63.6				1133.6				
355	9	ALU	ALU	Np	5	377	3	2	219.2	811.0	AluNp5	377	219.2				811.0				
356	9	ALU	ALU	Np	5	280	4	25	102.0	1221.6	AluNp5	280	102.0				1221.6				
357	9	ALU	ALU	Np	5	336	5	23	15.9	1128.3	AluNp5	336	15.9				1128.3				
358	9	ALU	ALU	Np	5	364	6	7	218.0	1026.1	AluNp5	364	218.0				1026.1				
359	9	ALU	ALU	Np	5	367	7	1	42.6	1146.0	AluNp5	367	42.6				1146.0				
360	9	ALU	ALU	Np	5	216	8	5	0.0	1125.0	AluNp5	216	0.0	82.7	71.3	33.8	1125.0	1082.2	1039.2	76.2	
361	9	ALU	ALU	Null	NA	249	1	26	218.5	902.8	AluNull	249	218.5				902.8				
362	9	ALU	ALU	Null	NA	260	2	30	78.0	1001.4	AluNull	260	78.0				1001.4				
363	9	ALU	ALU	Null	NA	231	3	35	54.8	1133.0	AluNull	231	54.8				1133.0				
364	9	ALU	ALU	Null	NA	285	4	48	25.9	1227.3	AluNull	285	25.9				1227.3				
365	9	ALU	ALU	Null	NA	344	5	38	10.2	1058.7	AluNull	344	10.2				1058.7				
366	9	ALU	ALU	Null	NA	282	6	25	61.5	1090.5	AluNull	282	61.5				1090.5				
367	9	ALU	ALU	Null	NA	313	7	37	70.1	1101.7	AluNull	313	70.1				1101.7				
368	9	ALU	ALU	Null	NA	278	8	4	150.9	1065.7	AluNull	278	150.9				1065.7				
369	9	ALU	ALU	Null	NA	293	1	43	39.3	1095.7	AluNull	293	39.3				1095.7				
370	9	ALU	ALU	Null	NA	263	2	9	76.8	1044.7	AluNull	263	76.8				1044.7				
371	9	ALU	ALU	Null	NA	221	3	11	106.6	937.9	AluNull	221	106.6				937.9				
372	9	ALU	ALU	Null	NA	381	4	47	144.4	1084.0	AluNull	381	144.4				1084.0				
373	9	ALU	ALU	Null	NA	340	5	31	70.1	1053.9	AluNull	340	70.1				1053.9				
374	9	ALU	ALU	Null	NA	373	6	9	258.0	941.7	AluNull	373	258.0				941.7				
375	9	ALU	ALU	Null	NA	287	7	18	229.6	505.8	AluNull	287	229.6				505.8				
376	9	ALU	ALU	Null	NA	213	8	37	0.0	907.1	AluNull	213	0.0				907.1				
377	9	ALU	ALU	Null	NA	391	1	11	69.4	974.0	AluNull	391	69.4				974.0				
378	9	ALU	ALU	Null	NA	273	2	37	0.0	961.3	AluNull	273	0.0				961.3				
379	9	ALU	ALU	Null	NA	207	3	24	115.0	1041.5	AluNull	207	115.0				1041.5				
380	9	ALU	ALU	Null	NA	343	4	3	224.7	842.3	AluNull	343	224.7				842.3				
381	9	ALU	ALU	Null	NA	353	5	18	191.3	1061.1	AluNull	353	191.3				1061.1				
382	9	ALU	ALU	Null	NA	235	6	43	125.9	1071.5	AluNull	235	125.9				1071.5				
383	9	ALU	ALU	Null	NA	347	7	10	41.5	1130.1	AluNull	347	41.5				1130.1				
384	9	ALU	ALU	Null	NA	236	8	26	0.0	833.5	AluNull	236	0.0				833.5				
385	9	ALU	ALU	Null	NA	388	1	37	115.1	1099.3	AluNull	388	115.1				1099.3				
386	9	ALU	ALU	Null	NA	242	2	40	155.4	983.5	AluNull	242	155.4				983.5				
387	9	ALU	ALU	Null	NA	357	3	22	292.2	828.9	AluNull	357	292.2				828.9				
388	9	ALU	ALU	Null	NA	298	4	11	241.2	858.6	AluNull	298	241.2				858.6				
389	9	ALU	ALU	Null	NA	214	5	25	186.2	1094.2	AluNull	214	186.2				1094.2				
390	9	ALU	ALU	Null	NA	342	6	3	85.5	1046.2	AluNull	342	85.5				1046.2				
391	9	ALU	ALU	Null	NA	210	7	34	0.0	1014.0	AluNull	210	0.0				1014.0				
392	9	ALU	ALU	Null	NA	246	8	19	150.6	944.9	AluNull	246	150.6				944.9				
393	9	ALU	ALU	Null	NA	323	1	10	30.9	1092.6	AluNull	323	30.9				1092.6				
394	9	ALU	ALU	Null	NA	283	2	2	33.1	1049.8	AluNull	283	33.1				1049.8				

Supplemental Table S2

395	9	ALU	ALU	Null	NA	325	3	44	199.9	941.0	AluNull	325	199.9				941.0				
396	9	ALU	ALU	Null	NA	281	4	44	65.0	1197.0	AluNull	281	65.0				1197.0				
397	9	ALU	ALU	Null	NA	233	5	40	180.2	1047.1	AluNull	233	180.2				1047.1				
398	9	ALU	ALU	Null	NA	220	6	29	0.0	990.2	AluNull	220	0.0				990.2				
399	9	ALU	ALU	Null	NA	309	7	46	0.0	1053.2	AluNull	309	0.0				1053.2				
400	9	ALU	ALU	Null	NA	392	8	14	72.4	1108.5	AluNull	392	72.4	104.3	104.3	83.3	1108.5	1010.4	1010.4	124.9	
