# Supplementary material for: Artificial Selection on Microbiomes To Breed Microbiomes That Confer Salt Tolerance to Plants
Source: mSystems. 2021 Nov 30;6(6):e01125-21. doi: 10.1128/mSystems.01125-21 (PMC8631316; doi:10.1128/mSystems.01125-21)
Supplement: TABLE S1 [file msystems.01125-21-st001.pdf]

# Supplemental Table S1

**Table S1 (Data Generations 0-8)**

sent to co-authors for analyses 5. Sep. 2016

**Abbreviations used in this Table:** s

"NA" denotes empty cell (e.g., seed that failed to germinate)

"SOD" denotes sodium-sulfate stress, "ALU" denotes aluminum-sulfate stress

"Bact" denotes inoculation of soil&seed with the initial bacterial inoculum at the beginning of the experiment (i.e., inoculation of seeds of Generation 0)

"Null" denotes no microbiome-inoculation of a seed, but microbes can accumulate from aerial microbial rain

**Explanation of Columns:**

| | |
|---|---|
| **Column B** | selection cycle (Microbiome Generation); Generation "0" is baseline Generation prior to first round of selection; plants were not yet assigned to selection lines in Generation 0 |
| **Column C** | salt treatment of soil (and water used to water plants); SOD denotes sodium-sulfate stress, ALU denotes aluminum-sulfate stress |
| **Column D** | Pp is "plant present", a microbiome is harvested from rhizosphere and propagated to germinating seeds of next microbiome-generation; |
| " | Np is "no plant", a microbiome is harvested from fallow soil with no plant in pot, then propagated to (a) germinating seeds to test microbiome effects, and to (b) soil in pot with no seed planted in it (i.e., Fallow-Soil Microbiome Propagation) |
| " | Null is "no microbiome-inoculation of seed", plants acquire microbiomes from aerial microbial rain; number of replicates in Null-treatment increases in later Generations |
| **Column E** | selection lines in Pp and Np treatments; for each salt-treatment, there are 5 selection-lines for Pp, and 5 lines for Np |
| " | number of replicates in Pp-lines is always 8 replicates (minus a few seeds that did not germinate, or seeds that germinated very late and were then removed); |
| " | number of replicates in Np-lines is 8 replicates per line in Gen1-3, then was reduced to 6 replicates per line in Gen4, then to 4 replicates per line in subsequent Generations |
| **Column F** | plant number used to identify individual plants (i.e., pots); |
| " | each Generation had 200 pots, but 10+10 pots/soils were "parents" for the fallow-microbiome-propagation treatments, and |
| " | these fallow-microbiome "parents" are not included in this sheet and are moved to the second sheet in this Excel file |
| **Column G** | rack number; for each selection-line, replicates were blocked evenly between racks; there were 8 racks total, each with 25 pots (total 200 pots) |
| **Column H** | position of pot in rack; pots assigned to a particular rack were randomly assigned to the 25 pot-positions in each rack |
| **Column I** | dry weight (above-ground biomass in mg) of plant harvested at end of microbiome-generation; biomass was weighed blndly with respect to plant treatment |
| " | THESE ARE THE ABSOLUTE VALUES OF THE DEPENDENT VARIABLE "DRY WEIGHT" |
| " | "NA" in this column denotes that a seed did not germinate, or seed was removed because of very late germination, and therefore no DryWeight is available for this plant |
| **Column J** | "LineAverage" is the average calculated across all replicates in that line |
| **Column K** | "TreatmentAverage" is the average of the treatment (averaged across the 5 line-averages of the 5 lines in each treatment) |
| **Column L** | column to calculate "AverageDryWeight" for all observations in same SaltTreatment in same Generation |
| **Column M** | "AverageDryWeight" for all observations in same SaltTreatment in same Generation |
| **Column N** | "RelativeDryWeight" is calculated as observed DryWeight divided by "AverageDryWeight" (calculated in Column L, then copied into Column M) for all observations in same SaltTreatment in same Generation |
| " | THESE ARE THE RELATIVIZED VALUES OF THE DEPENDENT VARIABLE "DRY WEIGHT" |
| **Column O** | "Relative LineAverage" is the average of a line calculated across all relativized replicates in that line |
| **Column P** | "Relative TreatmentAverage" is the average of treatment (averaged across the 5 relativized averages of the 5 lines in each treatment) |
| **Column W** | Relative Fitness 1: Fitness (dry weight) of plant with selected microbiome, relative to average dry weight of Fallow-Soil plants |
| **Comumn AE** | Relative Fitness 2: Fitness (dry weight) of plant with selected microbiome, relative to average dry weight of Null plants |

| ### | Generation | Salt Treatment | Microbiome Treatment | Selection Line | Plant # | Rack # | Position in Rack | Dry Weight | Line Average | Treatment Average | Average Dry Weight for Salt Treatment of same Generation | Average Dry Weight for Salt Treatment of same Generation | Relative Dry Weight | Relative Line Average | Relative Treatment Average | | | | Treatment Average Ignoring Line-Nesting |
|---|---|---|---|---|---|---|---|---|---|---|---|---|---|---|---|---|---|---|---|
| | 0 | SOD | Bact | NA | 90 | 1 | 14 | 29.5 | | | | 31.9 | 0.92 | | | 0 | Bact | SOD | |
| | 0 | SOD | Bact | NA | 78 | 6 | 23 | 31.2 | | | | 31.9 | 0.98 | | | 0 | Bact | SOD | |
| | 0 | SOD | Bact | NA | 41 | 7 | 17 | 33.8 | | | | 31.9 | 1.06 | | | 0 | Bact | SOD | |
| | 0 | SOD | Bact | NA | 19 | 8 | 15 | 30.4 | | | | 31.9 | 0.95 | | | 0 | Bact | SOD | |
| | 0 | SOD | Bact | NA | 64 | 1 | 1 | 33.6 | | | | 31.9 | 1.05 | | | 0 | Bact | SOD | |
| | 0 | SOD | Bact | NA | 29 | 2 | 4 | 26.8 | | | | 31.9 | 0.84 | | | 0 | Bact | SOD | |
| | 0 | SOD | Bact | NA | 16 | 3 | 5 | NA | | | | NA | NA | | | 0 | Bact | SOD | |
| | 0 | SOD | Bact | NA | 95 | 5 | 7 | 28.3 | | | | 31.9 | 0.89 | | | 0 | Bact | SOD | |
| | 0 | SOD | Bact | NA | 69 | 2 | 10 | 30.1 | | | | 31.9 | 0.94 | | | 0 | Bact | SOD | |
| | 0 | SOD | Bact | NA | 23 | 3 | 19 | 29.7 | | | | 31.9 | 0.93 | | | 0 | Bact | SOD | |
| | 0 | SOD | Bact | NA | 50 | 4 | 7 | 35.6 | | | | 31.9 | 1.12 | | | 0 | Bact | SOD | |

# Supplemental Table S1

| Fallow-Soil-Control Relativization of Fitness Data, Averages & StDev | | | | | Null-Control Relativization of Fitness Data, Averages & StDev | | | | | | | |
|---|---|---|---|---|---|---|---|---|---|---|---|---|
| Average Dry Weight of Fallow-Soil Plant | Fitness Relative to Average Fallow-Soil Plant | Line-Averages | Averages | St.Dev. | Average Dry Weight of NullPlant | Fitness Relative to Average Null Plant | Line-Averages | Averages | St.Dev. | | | |
| NA | NA | NA | NA | NA | 32.59 | 0.91 | | | | 0 | Bact | SOD |
| NA | NA | NA | NA | NA | 32.59 | 0.96 | | | | 0 | Bact | SOD |
| NA | NA | NA | NA | NA | 32.59 | 1.04 | | | | 0 | Bact | SOD |
| NA | NA | NA | NA | NA | 32.59 | 0.93 | | | | 0 | Bact | SOD |
| NA | NA | NA | NA | NA | 32.59 | 1.03 | | | | 0 | Bact | SOD |
| NA | NA | NA | NA | NA | 32.59 | 0.82 | | | | 0 | Bact | SOD |
| NA | NA | NA | NA | NA | NA | NA | | | | 0 | Bact | SOD |
| NA | NA | NA | NA | NA | 32.59 | 0.87 | | | | 0 | Bact | SOD |
| NA | NA | NA | NA | NA | 32.59 | 0.92 | | | | 0 | Bact | SOD |
| NA | NA | NA | NA | NA | 32.59 | 0.91 | | | | 0 | Bact | SOD |
| NA | NA | NA | NA | NA | 32.59 | 1.09 | | | | 0 | Bact | SOD |

# Supplemental Table S1

| | | | | | | | | | | | | | | | | | |
|---|---|---|---|---|---|---|---|---|---|---|---|---|---|---|---|---|---|
| | 0 | SOD | Bact | NA | 82 | 2 | 18 | 32.9 | | | 31.9 | 1.03 | | 0 | Bact | SOD | |
| | 0 | SOD | Bact | NA | 2 | 5 | 25 | 29.0 | | | 31.9 | 0.91 | | 0 | Bact | SOD | |
| | 0 | SOD | Bact | NA | 35 | 8 | 19 | 28.0 | | | 31.9 | 0.88 | | 0 | Bact | SOD | |
| | 0 | SOD | Bact | NA | 53 | 2 | 5 | 38.4 | | | 31.9 | 1.20 | | 0 | Bact | SOD | |
| | 0 | SOD | Bact | NA | 93 | 3 | 23 | 27.0 | | | 31.9 | 0.85 | | 0 | Bact | SOD | |
| | 0 | SOD | Bact | NA | 62 | 6 | 3 | 32.3 | | | 31.9 | 1.01 | | 0 | Bact | SOD | |
| | 0 | SOD | Bact | NA | 71 | 1 | 16 | 29.5 | | | 31.9 | 0.92 | | 0 | Bact | SOD | |
| | 0 | SOD | Bact | NA | 18 | 3 | 20 | 25.5 | | | 31.9 | 0.80 | | 0 | Bact | SOD | |
| | 0 | SOD | Bact | NA | 1 | 4 | 19 | 32.5 | | | 31.9 | 1.02 | | 0 | Bact | SOD | |
| | 0 | SOD | Bact | NA | 24 | 5 | 5 | 32.9 | | | 31.9 | 1.03 | | 0 | Bact | SOD | |
| | 0 | SOD | Bact | NA | 12 | 6 | 9 | 31.6 | | | 31.9 | 0.99 | | 0 | Bact | SOD | |
| | 0 | SOD | Bact | NA | 88 | 3 | 14 | 34.9 | | | 31.9 | 1.09 | | 0 | Bact | SOD | |
| | 0 | SOD | Bact | NA | 31 | 5 | 9 | 33.5 | | | 31.9 | 1.05 | | 0 | Bact | SOD | |
| | 0 | SOD | Bact | NA | 32 | 6 | 14 | 36.2 | | | 31.9 | 1.13 | | 0 | Bact | SOD | |
| | 0 | SOD | Bact | NA | 48 | 8 | 4 | 35.9 | | | 31.9 | 1.13 | | 0 | Bact | SOD | |
| | 0 | SOD | Bact | NA | 61 | 3 | 9 | 31.1 | | | 31.9 | 0.97 | | 0 | Bact | SOD | |
| | 0 | SOD | Bact | NA | 89 | 4 | 11 | 26.3 | | | 31.9 | 0.82 | | 0 | Bact | SOD | |
| | 0 | SOD | Bact | NA | 17 | 8 | 11 | 26.1 | | | 31.9 | 0.82 | | 0 | Bact | SOD | |
| | 0 | SOD | Bact | NA | 37 | 5 | 22 | 35.2 | | | 31.9 | 1.10 | | 0 | Bact | SOD | |
| | 0 | SOD | Bact | NA | 22 | 8 | 22 | 35.8 | | | 31.9 | 1.12 | | 0 | Bact | SOD | |
| | 0 | SOD | Bact | NA | 99 | 1 | 4 | 34.5 | | | 31.9 | 1.08 | | 0 | Bact | SOD | |
| | 0 | SOD | Bact | NA | 45 | 6 | 19 | 30.8 | | | 31.9 | 0.97 | | 0 | Bact | SOD | |
| | 0 | SOD | Bact | NA | 38 | 3 | 12 | 33.0 | | | 31.9 | 1.03 | | 0 | Bact | SOD | |
| | 0 | SOD | Bact | NA | 25 | 4 | 6 | 33.1 | | | 31.9 | 1.04 | | 0 | Bact | SOD | |
| | 0 | SOD | Bact | NA | 15 | 5 | 18 | 29.3 | | | 31.9 | 0.92 | | 0 | Bact | SOD | |
| | 0 | SOD | Bact | NA | 14 | 2 | 11 | 33.4 | | | 31.9 | 1.05 | | 0 | Bact | SOD | |
| | 0 | SOD | Bact | NA | 26 | 5 | 8 | 34.2 | | | 31.9 | 1.07 | | 0 | Bact | SOD | |
| | 0 | SOD | Bact | NA | 79 | 8 | 12 | NA | | | NA | NA | | 0 | Bact | SOD | |
| | 0 | SOD | Bact | NA | 47 | 2 | 24 | 33.4 | | | 31.9 | 1.05 | | 0 | Bact | SOD | |
| | 0 | SOD | Bact | NA | 40 | 6 | 24 | 33.3 | | | 31.9 | 1.04 | | 0 | Bact | SOD | |
| | 0 | SOD | Bact | NA | 7 | 7 | 25 | 33.5 | | | 31.9 | 1.05 | | 0 | Bact | SOD | |
| | 0 | SOD | Bact | NA | 13 | 1 | 10 | 27.3 | | | 31.9 | 0.86 | | 0 | Bact | SOD | |
| | 0 | SOD | Bact | NA | 36 | 6 | 10 | 29.5 | | | 31.9 | 0.92 | | 0 | Bact | SOD | |
| | 0 | SOD | Bact | NA | 85 | 2 | 9 | 29.9 | | | 31.9 | 0.94 | | 0 | Bact | SOD | |
| | 0 | SOD | Bact | NA | 92 | 4 | 24 | 27.0 | | | 31.9 | 0.85 | | 0 | Bact | SOD | |
| | 0 | SOD | Bact | NA | 55 | 7 | 12 | 35.0 | | | 31.9 | 1.10 | | 0 | Bact | SOD | |
| | 0 | SOD | Bact | NA | 21 | 1 | 6 | 28.1 | | | 31.9 | 0.88 | | 0 | Bact | SOD | |
| | 0 | SOD | Bact | NA | 27 | 6 | 20 | 32.4 | | | 31.9 | 1.02 | | 0 | Bact | SOD | |
| | 0 | SOD | Bact | NA | 57 | 8 | 6 | 32.4 | | | 31.9 | 1.02 | | 0 | Bact | SOD | |
| | 0 | SOD | Bact | NA | 67 | 1 | 25 | 32.8 | | | 31.9 | 1.03 | | 0 | Bact | SOD | |
| | 0 | SOD | Bact | NA | 81 | 2 | 7 | 34.8 | | | 31.9 | 1.09 | | 0 | Bact | SOD | |
| | 0 | SOD | Bact | NA | 20 | 3 | 7 | 33.6 | | | 31.9 | 1.05 | | 0 | Bact | SOD | |
| | 0 | SOD | Bact | NA | 74 | 5 | 16 | 25.7 | | | 31.9 | 0.81 | | 0 | Bact | SOD | |
| | 0 | SOD | Bact | NA | 65 | 6 | 4 | 26.8 | | | 31.9 | 0.84 | | 0 | Bact | SOD | |
| | 0 | SOD | Bact | NA | 6 | 7 | 15 | 31.2 | | | 31.9 | 0.98 | | 0 | Bact | SOD | |
| | 0 | SOD | Bact | NA | 94 | 2 | 8 | 36.3 | | | 31.9 | 1.14 | | 0 | Bact | SOD | |
| | 0 | SOD | Bact | NA | 84 | 3 | 17 | 32.5 | | | 31.9 | 1.02 | | 0 | Bact | SOD | |
| | 0 | SOD | Bact | NA | 9 | 8 | 13 | 33.9 | | | 31.9 | 1.06 | | 0 | Bact | SOD | |
| | 0 | SOD | Bact | NA | 66 | 2 | 12 | 33.5 | | | 31.9 | 1.05 | | 0 | Bact | SOD | |
| | 0 | SOD | Bact | NA | 60 | 7 | 7 | 31.4 | | | 31.9 | 0.98 | | 0 | Bact | SOD | |
| | 0 | SOD | Bact | NA | 43 | 8 | 7 | 38.0 | | | 31.9 | 1.19 | | 0 | Bact | SOD | |
| | 0 | SOD | Bact | NA | 98 | 2 | 15 | 25.8 | | | 31.9 | 0.81 | | 0 | Bact | SOD | |
| | 0 | SOD | Bact | NA | 72 | 4 | 12 | 33.4 | | | 31.9 | 1.05 | | 0 | Bact | SOD | |

# Supplemental Table S1

| | | | | | | | | | | | | |
|---|---|---|---|---|---|---|---|---|---|---|---|---|
| NA | NA | NA | NA | NA | 32.59 | 1.01 | | | | 0 | Bact | SOD |
| NA | NA | NA | NA | NA | 32.59 | 0.89 | | | | 0 | Bact | SOD |
| NA | NA | NA | NA | NA | 32.59 | 0.86 | | | | 0 | Bact | SOD |
| NA | NA | NA | NA | NA | 32.59 | 1.18 | | | | 0 | Bact | SOD |
| NA | NA | NA | NA | NA | 32.59 | 0.83 | | | | 0 | Bact | SOD |
| NA | NA | NA | NA | NA | 32.59 | 0.99 | | | | 0 | Bact | SOD |
| NA | NA | NA | NA | NA | 32.59 | 0.91 | | | | 0 | Bact | SOD |
| NA | NA | NA | NA | NA | 32.59 | 0.78 | | | | 0 | Bact | SOD |
| NA | NA | NA | NA | NA | 32.59 | 1.00 | | | | 0 | Bact | SOD |
| NA | NA | NA | NA | NA | 32.59 | 1.01 | | | | 0 | Bact | SOD |
| NA | NA | NA | NA | NA | 32.59 | 0.97 | | | | 0 | Bact | SOD |
| NA | NA | NA | NA | NA | 32.59 | 1.07 | | | | 0 | Bact | SOD |
| NA | NA | NA | NA | NA | 32.59 | 1.03 | | | | 0 | Bact | SOD |
| NA | NA | NA | NA | NA | 32.59 | 1.11 | | | | 0 | Bact | SOD |
| NA | NA | NA | NA | NA | 32.59 | 1.10 | | | | 0 | Bact | SOD |
| NA | NA | NA | NA | NA | 32.59 | 0.95 | | | | 0 | Bact | SOD |
| NA | NA | NA | NA | NA | 32.59 | 0.81 | | | | 0 | Bact | SOD |
| NA | NA | NA | NA | NA | 32.59 | 0.80 | | | | 0 | Bact | SOD |
| NA | NA | NA | NA | NA | 32.59 | 1.08 | | | | 0 | Bact | SOD |
| NA | NA | NA | NA | NA | 32.59 | 1.10 | | | | 0 | Bact | SOD |
| NA | NA | NA | NA | NA | 32.59 | 1.06 | | | | 0 | Bact | SOD |
| NA | NA | NA | NA | NA | 32.59 | 0.95 | | | | 0 | Bact | SOD |
| NA | NA | NA | NA | NA | 32.59 | 1.01 | | | | 0 | Bact | SOD |
| NA | NA | NA | NA | NA | 32.59 | 1.02 | | | | 0 | Bact | SOD |
| NA | NA | NA | NA | NA | 32.59 | 0.90 | | | | 0 | Bact | SOD |
| NA | NA | NA | NA | NA | 32.59 | 1.02 | | | | 0 | Bact | SOD |
| NA | NA | NA | NA | NA | 32.59 | 1.05 | | | | 0 | Bact | SOD |
| NA | NA | NA | NA | NA | NA | NA | | | | 0 | Bact | SOD |
| NA | NA | NA | NA | NA | 32.59 | 1.02 | | | | 0 | Bact | SOD |
| NA | NA | NA | NA | NA | 32.59 | 1.02 | | | | 0 | Bact | SOD |
| NA | NA | NA | NA | NA | 32.59 | 1.03 | | | | 0 | Bact | SOD |
| NA | NA | NA | NA | NA | 32.59 | 0.84 | | | | 0 | Bact | SOD |
| NA | NA | NA | NA | NA | 32.59 | 0.91 | | | | 0 | Bact | SOD |
| NA | NA | NA | NA | NA | 32.59 | 0.92 | | | | 0 | Bact | SOD |
| NA | NA | NA | NA | NA | 32.59 | 0.83 | | | | 0 | Bact | SOD |
| NA | NA | NA | NA | NA | 32.59 | 1.07 | | | | 0 | Bact | SOD |
| NA | NA | NA | NA | NA | 32.59 | 0.86 | | | | 0 | Bact | SOD |
| NA | NA | NA | NA | NA | 32.59 | 0.99 | | | | 0 | Bact | SOD |
| NA | NA | NA | NA | NA | 32.59 | 0.99 | | | | 0 | Bact | SOD |
| NA | NA | NA | NA | NA | 32.59 | 1.01 | | | | 0 | Bact | SOD |
| NA | NA | NA | NA | NA | 32.59 | 1.07 | | | | 0 | Bact | SOD |
| NA | NA | NA | NA | NA | 32.59 | 1.03 | | | | 0 | Bact | SOD |
| NA | NA | NA | NA | NA | 32.59 | 0.79 | | | | 0 | Bact | SOD |
| NA | NA | NA | NA | NA | 32.59 | 0.82 | | | | 0 | Bact | SOD |
| NA | NA | NA | NA | NA | 32.59 | 0.96 | | | | 0 | Bact | SOD |
| NA | NA | NA | NA | NA | 32.59 | 1.11 | | | | 0 | Bact | SOD |
| NA | NA | NA | NA | NA | 32.59 | 1.00 | | | | 0 | Bact | SOD |
| NA | NA | NA | NA | NA | 32.59 | 1.04 | | | | 0 | Bact | SOD |
| NA | NA | NA | NA | NA | 32.59 | 1.03 | | | | 0 | Bact | SOD |
| NA | NA | NA | NA | NA | 32.59 | 0.96 | | | | 0 | Bact | SOD |
| NA | NA | NA | NA | NA | 32.59 | 1.17 | | | | 0 | Bact | SOD |
| NA | NA | NA | NA | NA | 32.59 | 0.79 | | | | 0 | Bact | SOD |
| NA | NA | NA | NA | NA | 32.59 | 1.02 | | | | 0 | Bact | SOD |

# Supplemental Table S1

| | | | | | | | | | | | | | | | | | | | |
|---|---|---|---|---|---|---|---|---|---|---|---|---|---|---|---|---|---|---|---|
| | 0 | SOD | Bact | NA | 91 | 4 | 23 | 32.2 | | | | 31.9 | 1.01 | | | 0 | Bact | SOD | |
| | 0 | SOD | Bact | NA | 49 | 5 | 19 | 30.5 | | | | 31.9 | 0.96 | | | 0 | Bact | SOD | |
| | 0 | SOD | Bact | NA | 46 | 7 | 9 | 32.8 | | | | 31.9 | 1.03 | | | 0 | Bact | SOD | |
| | 0 | SOD | Bact | NA | 44 | 1 | 21 | 26.0 | | | | 31.9 | 0.82 | | | 0 | Bact | SOD | |
| | 0 | SOD | Bact | NA | 30 | 2 | 19 | 37.2 | | | | 31.9 | 1.17 | | | 0 | Bact | SOD | |
| | 0 | SOD | Bact | NA | 34 | 4 | 2 | 34.4 | | | | 31.9 | 1.08 | | | 0 | Bact | SOD | |
| | 0 | SOD | Bact | NA | 51 | 7 | 8 | NA | | | | NA | NA | | | 0 | Bact | SOD | |
| | 0 | SOD | Bact | NA | 96 | 2 | 23 | 35.6 | | | | 31.9 | 1.12 | | | 0 | Bact | SOD | |
| | 0 | SOD | Bact | NA | 8 | 5 | 14 | 30.5 | | | | 31.9 | 0.96 | | | 0 | Bact | SOD | |
| | 0 | SOD | Bact | NA | 33 | 8 | 18 | 31.6 | | | | 31.9 | 0.99 | | | 0 | Bact | SOD | |
| | 0 | SOD | Bact | NA | 5 | 1 | 24 | 40.0 | | | | 31.9 | 1.25 | | | 0 | Bact | SOD | |
| | 0 | SOD | Bact | NA | 73 | 4 | 3 | 29.3 | | | | 31.9 | 0.92 | | | 0 | Bact | SOD | |
| | 0 | SOD | Bact | NA | 86 | 1 | 2 | 30.2 | | | | 31.9 | 0.95 | | | 0 | Bact | SOD | |
| | 0 | SOD | Bact | NA | 56 | 2 | 13 | 22.2 | | | | 31.9 | 0.70 | | | 0 | Bact | SOD | |
| | 0 | SOD | Bact | NA | 97 | 3 | 22 | 30.7 | | | | 31.9 | 0.96 | | | 0 | Bact | SOD | |
| | 0 | SOD | Bact | NA | 54 | 7 | 13 | 36.0 | 31.72 | | | 31.9 | 1.13 | 0.994 | | 0 | Bact | SOD | 31.7 |
| | 0 | SOD | Null | NA | 42 | 4 | 4 | 30.6 | | | | 31.9 | 0.96 | | | 0 | Null | SOD | |
| | 0 | SOD | Null | NA | 68 | 6 | 15 | 32.2 | | | | 31.9 | 1.01 | | | 0 | Null | SOD | |
| | 0 | SOD | Null | NA | 76 | 1 | 7 | 31.3 | | | | 31.9 | 0.98 | | | 0 | Null | SOD | |
| | 0 | SOD | Null | NA | 39 | 3 | 24 | 31.3 | | | | 31.9 | 0.98 | | | 0 | Null | SOD | |
| | 0 | SOD | Null | NA | 77 | 7 | 19 | 30.6 | | | | 31.9 | 0.96 | | | 0 | Null | SOD | |
| | 0 | SOD | Null | NA | 70 | 4 | 22 | 31.8 | | | | 31.9 | 1.00 | | | 0 | Null | SOD | |
| | 0 | SOD | Null | NA | 11 | 3 | 11 | 40.3 | | | | 31.9 | 1.26 | | | 0 | Null | SOD | |
| | 0 | SOD | Null | NA | 59 | 2 | 6 | 33.9 | | | | 31.9 | 1.06 | | | 0 | Null | SOD | |
| | 0 | SOD | Null | NA | 100 | 6 | 25 | 31.3 | | | | 31.9 | 0.98 | | | 0 | Null | SOD | |
| | 0 | SOD | Null | NA | 63 | 6 | 5 | 32.5 | | | | 31.9 | 1.02 | | | 0 | Null | SOD | |
| | 0 | SOD | Null | NA | 3 | 5 | 11 | 32.0 | | | | 31.9 | 1.00 | | | 0 | Null | SOD | |
| | 0 | SOD | Null | NA | 10 | 5 | 1 | 30.3 | | | | 31.9 | 0.95 | | | 0 | Null | SOD | |
| | 0 | SOD | Null | NA | 75 | 1 | 23 | 35.6 | | | | 31.9 | 1.12 | | | 0 | Null | SOD | |
| | 0 | SOD | Null | NA | 80 | 3 | 18 | 28.1 | | | | 31.9 | 0.88 | | | 0 | Null | SOD | |
| | 0 | SOD | Null | NA | 4 | 5 | 15 | 34.0 | | | | 31.9 | 1.07 | | | 0 | Null | SOD | |
| | 0 | SOD | Null | NA | 58 | 7 | 23 | 32.0 | | | | 31.9 | 1.00 | | | 0 | Null | SOD | |
| | 0 | SOD | Null | NA | 28 | 2 | 20 | 32.6 | | | | 31.9 | 1.02 | | | 0 | Null | SOD | |
| | 0 | SOD | Null | NA | 87 | 8 | 2 | 34.7 | | | | 31.9 | 1.09 | | | 0 | Null | SOD | |
| | 0 | SOD | Null | NA | 52 | 2 | 22 | 31.3 | | | Grand Average | 31.9 | 0.98 | | | 0 | Null | SOD | |
| | 0 | SOD | Null | NA | 83 | 6 | 21 | 35.4 | 32.59 | | 31.9 | 31.9 | 1.11 | 1.022 | | 0 | Null | SOD | 32.59 |
| | 0 | ALU | Bact | NA | 144 | 2 | 25 | 37.0 | | | | 33.1 | 1.12 | | | 0 | Bact | ALU | |
| | 0 | ALU | Bact | NA | 177 | 3 | 1 | 26.1 | | | | 33.1 | 0.79 | | | 0 | Bact | ALU | |
| | 0 | ALU | Bact | NA | 151 | 5 | 17 | 30.1 | | | | 33.1 | 0.91 | | | 0 | Bact | ALU | |
| | 0 | ALU | Bact | NA | 156 | 7 | 3 | 40.0 | | | | 33.1 | 1.21 | | | 0 | Bact | ALU | |
| | 0 | ALU | Bact | NA | 133 | 8 | 21 | 36.2 | | | | 33.1 | 1.09 | | | 0 | Bact | ALU | |
| | 0 | ALU | Bact | NA | 170 | 5 | 2 | 31.4 | | | | 33.1 | 0.95 | | | 0 | Bact | ALU | |
| | 0 | ALU | Bact | NA | 146 | 6 | 18 | NA | | | | NA | NA | | | 0 | Bact | ALU | |
| | 0 | ALU | Bact | NA | 186 | 7 | 2 | 36.0 | | | | 33.1 | 1.09 | | | 0 | Bact | ALU | |
| | 0 | ALU | Bact | NA | 169 | 8 | 10 | 38.5 | | | | 33.1 | 1.16 | | | 0 | Bact | ALU | |
| | 0 | ALU | Bact | NA | 132 | 4 | 18 | 34.7 | | | | 33.1 | 1.05 | | | 0 | Bact | ALU | |
| | 0 | ALU | Bact | NA | 116 | 6 | 2 | 33.9 | | | | 33.1 | 1.02 | | | 0 | Bact | ALU | |
| | 0 | ALU | Bact | NA | 145 | 1 | 5 | 25.6 | | | | 33.1 | 0.77 | | | 0 | Bact | ALU | |
| | 0 | ALU | Bact | NA | 111 | 4 | 9 | 31.9 | | | | 33.1 | 0.96 | | | 0 | Bact | ALU | |
| | 0 | ALU | Bact | NA | 193 | 7 | 11 | 31.6 | | | | 33.1 | 0.95 | | | 0 | Bact | ALU | |
| | 0 | ALU | Bact | NA | 149 | 2 | 1 | NA | | | | NA | NA | | | 0 | Bact | ALU | |
| | 0 | ALU | Bact | NA | 153 | 7 | 20 | 34.4 | | | | 33.1 | 1.04 | | | 0 | Bact | ALU | |
| | 0 | ALU | Bact | NA | 147 | 8 | 17 | 31.5 | | | | 33.1 | 0.95 | | | 0 | Bact | ALU | |

## Supplemental Table S1

| | | | | | | | | | | | | |
|---|---|---|---|---|---|---|---|---|---|---|---|---|
| NA | NA | NA | NA | NA | 32.59 | 0.99 | | | | 0 | Bact | SOD |
| NA | NA | NA | NA | NA | 32.59 | 0.94 | | | | 0 | Bact | SOD |
| NA | NA | NA | NA | NA | 32.59 | 1.01 | | | | 0 | Bact | SOD |
| NA | NA | NA | NA | NA | 32.59 | 0.80 | | | | 0 | Bact | SOD |
| NA | NA | NA | NA | NA | 32.59 | 1.14 | | | | 0 | Bact | SOD |
| NA | NA | NA | NA | NA | 32.59 | 1.06 | | | | 0 | Bact | SOD |
| NA | NA | NA | NA | NA | NA | NA | | | | 0 | Bact | SOD |
| NA | NA | NA | NA | NA | 32.59 | 1.09 | | | | 0 | Bact | SOD |
| NA | NA | NA | NA | NA | 32.59 | 0.94 | | | | 0 | Bact | SOD |
| NA | NA | NA | NA | NA | 32.59 | 0.97 | | | | 0 | Bact | SOD |
| NA | NA | NA | NA | NA | 32.59 | 1.23 | | | | 0 | Bact | SOD |
| NA | NA | NA | NA | NA | 32.59 | 0.90 | | | | 0 | Bact | SOD |
| NA | NA | NA | NA | NA | 32.59 | 0.93 | | | | 0 | Bact | SOD |
| NA | NA | NA | NA | NA | 32.59 | 0.68 | | Generation 0 | | 0 | Bact | SOD |
| NA | NA | NA | NA | NA | 32.59 | 0.94 | | Average | StDev | 0 | Bact | SOD |
| NA | NA | NA | NA | NA | 32.59 | 1.10 | | 0.973 | 0.107 | 0 | Bact | SOD |
| NA | NA | NA | NA | NA | 32.59 | 0.94 | | | | 0 | Null | SOD |
| NA | NA | NA | NA | NA | 32.59 | 0.99 | | | | 0 | Null | SOD |
| NA | NA | NA | NA | NA | 32.59 | 0.96 | | | | 0 | Null | SOD |
| NA | NA | NA | NA | NA | 32.59 | 0.96 | | | | 0 | Null | SOD |
| NA | NA | NA | NA | NA | 32.59 | 0.94 | | | | 0 | Null | SOD |
| NA | NA | NA | NA | NA | 32.59 | 0.98 | | | | 0 | Null | SOD |
| NA | NA | NA | NA | NA | 32.59 | 1.24 | | | | 0 | Null | SOD |
| NA | NA | NA | NA | NA | 32.59 | 1.04 | | | | 0 | Null | SOD |
| NA | NA | NA | NA | NA | 32.59 | 0.96 | | | | 0 | Null | SOD |
| NA | NA | NA | NA | NA | 32.59 | 1.00 | | | | 0 | Null | SOD |
| NA | NA | NA | NA | NA | 32.59 | 0.98 | | | | 0 | Null | SOD |
| NA | NA | NA | NA | NA | 32.59 | 0.93 | | | | 0 | Null | SOD |
| NA | NA | NA | NA | NA | 32.59 | 1.09 | | | | 0 | Null | SOD |
| NA | NA | NA | NA | NA | 32.59 | 0.86 | | | | 0 | Null | SOD |
| NA | NA | NA | NA | NA | 32.59 | 1.04 | | | | 0 | Null | SOD |
| NA | NA | NA | NA | NA | 32.59 | 0.98 | | | | 0 | Null | SOD |
| NA | NA | NA | NA | NA | 32.59 | 1.00 | | | | 0 | Null | SOD |
| NA | NA | NA | NA | NA | 32.59 | 1.06 | | | | 0 | Null | SOD |
| NA | NA | NA | NA | NA | 32.59 | 0.96 | | | | 0 | Null | SOD |
| NA | NA | NA | NA | NA | 32.59 | 1.09 | | | | 0 | Null | SOD |
| NA | NA | NA | NA | NA | 34.21 | 1.08 | | | | 0 | Bact | ALU |
| NA | NA | NA | NA | NA | 34.21 | 0.76 | | | | 0 | Bact | ALU |
| NA | NA | NA | NA | NA | 34.21 | 0.88 | | | | 0 | Bact | ALU |
| NA | NA | NA | NA | NA | 34.21 | 1.17 | | | | 0 | Bact | ALU |
| NA | NA | NA | NA | NA | 34.21 | 1.06 | | | | 0 | Bact | ALU |
| NA | NA | NA | NA | NA | 34.21 | 0.92 | | | | 0 | Bact | ALU |
| NA | NA | NA | NA | NA | NA | NA | | | | 0 | Bact | ALU |
| NA | NA | NA | NA | NA | 34.21 | 1.05 | | | | 0 | Bact | ALU |
| NA | NA | NA | NA | NA | 34.21 | 1.13 | | | | 0 | Bact | ALU |
| NA | NA | NA | NA | NA | 34.21 | 1.01 | | | | 0 | Bact | ALU |
| NA | NA | NA | NA | NA | 34.21 | 0.99 | | | | 0 | Bact | ALU |
| NA | NA | NA | NA | NA | 34.21 | 0.75 | | | | 0 | Bact | ALU |
| NA | NA | NA | NA | NA | 34.21 | 0.93 | | | | 0 | Bact | ALU |
| NA | NA | NA | NA | NA | 34.21 | 0.92 | | | | 0 | Bact | ALU |
| NA | NA | NA | NA | NA | NA | NA | | | | 0 | Bact | ALU |
| NA | NA | NA | NA | NA | 34.21 | 1.01 | | | | 0 | Bact | ALU |
| NA | NA | NA | NA | NA | 34.21 | 0.92 | | | | 0 | Bact | ALU |

# Supplemental Table S1

| | | | | | | | | | | | | | | | | | |
|---|---|---|---|---|---|---|---|---|---|---|---|---|---|---|---|---|---|
| | 0 | ALU | Bact | NA | 148 | 1 | 20 | 33.6 | | | 33.1 | 1.02 | | 0 | Bact | ALU | |
| | 0 | ALU | Bact | NA | 172 | 2 | 16 | 29.5 | | | 33.1 | 0.89 | | 0 | Bact | ALU | |
| | 0 | ALU | Bact | NA | 103 | 5 | 24 | 34.2 | | | 33.1 | 1.03 | | 0 | Bact | ALU | |
| | 0 | ALU | Bact | NA | 197 | 6 | 6 | 33.6 | | | 33.1 | 1.02 | | 0 | Bact | ALU | |
| | 0 | ALU | Bact | NA | 122 | 7 | 4 | 31.4 | | | 33.1 | 0.95 | | 0 | Bact | ALU | |
| | 0 | ALU | Bact | NA | 139 | 1 | 17 | NA | | | NA | NA | | 0 | Bact | ALU | |
| | 0 | ALU | Bact | NA | 188 | 2 | 3 | 37.5 | | | 33.1 | 1.13 | | 0 | Bact | ALU | |
| | 0 | ALU | Bact | NA | 129 | 4 | 15 | 30.4 | | | 33.1 | 0.92 | | 0 | Bact | ALU | |
| | 0 | ALU | Bact | NA | 180 | 2 | 21 | NA | | | NA | NA | | 0 | Bact | ALU | |
| | 0 | ALU | Bact | NA | 123 | 3 | 6 | 34.3 | | | 33.1 | 1.04 | | 0 | Bact | ALU | |
| | 0 | ALU | Bact | NA | 112 | 4 | 20 | 34.8 | | | 33.1 | 1.05 | | 0 | Bact | ALU | |
| | 0 | ALU | Bact | NA | 159 | 5 | 23 | 34.6 | | | 33.1 | 1.05 | | 0 | Bact | ALU | |
| | 0 | ALU | Bact | NA | 114 | 7 | 22 | 21.0 | | | 33.1 | 0.63 | | 0 | Bact | ALU | |
| | 0 | ALU | Bact | NA | 162 | 1 | 18 | 34.3 | | | 33.1 | 1.04 | | 0 | Bact | ALU | |
| | 0 | ALU | Bact | NA | 195 | 7 | 1 | 35.9 | | | 33.1 | 1.08 | | 0 | Bact | ALU | |
| | 0 | ALU | Bact | NA | 124 | 8 | 23 | 39.6 | | | 33.1 | 1.20 | | 0 | Bact | ALU | |
| | 0 | ALU | Bact | NA | 189 | 1 | 22 | 31.0 | | | 33.1 | 0.94 | | 0 | Bact | ALU | |
| | 0 | ALU | Bact | NA | 137 | 3 | 25 | 27.6 | | | 33.1 | 0.83 | | 0 | Bact | ALU | |
| | 0 | ALU | Bact | NA | 131 | 4 | 5 | 28.3 | | | 33.1 | 0.86 | | 0 | Bact | ALU | |
| | 0 | ALU | Bact | NA | 198 | 7 | 21 | 29.7 | | | 33.1 | 0.90 | | 0 | Bact | ALU | |
| | 0 | ALU | Bact | NA | 173 | 3 | 4 | 31.5 | | | 33.1 | 0.95 | | 0 | Bact | ALU | |
| | 0 | ALU | Bact | NA | 175 | 4 | 25 | 32.7 | | | 33.1 | 0.99 | | 0 | Bact | ALU | |
| | 0 | ALU | Bact | NA | 187 | 8 | 25 | 29.2 | | | 33.1 | 0.88 | | 0 | Bact | ALU | |
| | 0 | ALU | Bact | NA | 140 | 3 | 15 | 27.7 | | | 33.1 | 0.84 | | 0 | Bact | ALU | |
| | 0 | ALU | Bact | NA | 200 | 4 | 1 | 36.0 | | | 33.1 | 1.09 | | 0 | Bact | ALU | |
| | 0 | ALU | Bact | NA | 135 | 5 | 13 | 26.4 | | | 33.1 | 0.80 | | 0 | Bact | ALU | |
| | 0 | ALU | Bact | NA | 199 | 1 | 8 | 38.3 | | | 33.1 | 1.16 | | 0 | Bact | ALU | |
| | 0 | ALU | Bact | NA | 127 | 3 | 3 | 37.1 | | | 33.1 | 1.12 | | 0 | Bact | ALU | |
| | 0 | ALU | Bact | NA | 105 | 6 | 7 | 33.5 | | | 33.1 | 1.01 | | 0 | Bact | ALU | |
| | 0 | ALU | Bact | NA | 181 | 8 | 16 | 30.7 | | | 33.1 | 0.93 | | 0 | Bact | ALU | |
| | 0 | ALU | Bact | NA | 196 | 2 | 17 | 39.7 | | | 33.1 | 1.20 | | 0 | Bact | ALU | |
| | 0 | ALU | Bact | NA | 110 | 3 | 10 | 29.3 | | | 33.1 | 0.89 | | 0 | Bact | ALU | |
| | 0 | ALU | Bact | NA | 171 | 7 | 16 | 33.1 | | | 33.1 | 1.00 | | 0 | Bact | ALU | |
| | 0 | ALU | Bact | NA | 163 | 4 | 16 | 30.3 | | | 33.1 | 0.92 | | 0 | Bact | ALU | |
| | 0 | ALU | Bact | NA | 125 | 1 | 19 | 41.0 | | | 33.1 | 1.24 | | 0 | Bact | ALU | |
| | 0 | ALU | Bact | NA | 138 | 4 | 13 | 42.2 | | | 33.1 | 1.28 | | 0 | Bact | ALU | |
| | 0 | ALU | Bact | NA | 160 | 5 | 10 | 28.3 | | | 33.1 | 0.86 | | 0 | Bact | ALU | |
| | 0 | ALU | Bact | NA | 119 | 6 | 1 | 29.2 | | | 33.1 | 0.88 | | 0 | Bact | ALU | |
| | 0 | ALU | Bact | NA | 104 | 7 | 5 | 33.4 | | | 33.1 | 1.01 | | 0 | Bact | ALU | |
| | 0 | ALU | Bact | NA | 191 | 4 | 10 | 34.0 | | | 33.1 | 1.03 | | 0 | Bact | ALU | |
| | 0 | ALU | Bact | NA | 141 | 5 | 21 | 32.0 | | | 33.1 | 0.97 | | 0 | Bact | ALU | |
| | 0 | ALU | Bact | NA | 161 | 1 | 15 | NA | | | NA | NA | | 0 | Bact | ALU | |
| | 0 | ALU | Bact | NA | 126 | 5 | 6 | 28.6 | | | 33.1 | 0.86 | | 0 | Bact | ALU | |
| | 0 | ALU | Bact | NA | 107 | 6 | 16 | 32.4 | | | 33.1 | 0.98 | | 0 | Bact | ALU | |
| | 0 | ALU | Bact | NA | 178 | 7 | 14 | 34.6 | | | 33.1 | 1.05 | | 0 | Bact | ALU | |
| | 0 | ALU | Bact | NA | 101 | 8 | 1 | NA | | | NA | NA | | 0 | Bact | ALU | |
| | 0 | ALU | Bact | NA | 194 | 1 | 11 | 22.9 | | | 33.1 | 0.69 | | 0 | Bact | ALU | |
| | 0 | ALU | Bact | NA | 190 | 2 | 2 | 38.6 | | | 33.1 | 1.17 | | 0 | Bact | ALU | |
| | 0 | ALU | Bact | NA | 109 | 3 | 21 | 33.3 | | | 33.1 | 1.01 | | 0 | Bact | ALU | |
| | 0 | ALU | Bact | NA | 174 | 6 | 8 | 27.8 | | | 33.1 | 0.84 | | 0 | Bact | ALU | |
| | 0 | ALU | Bact | NA | 167 | 8 | 8 | NA | | | NA | NA | | 0 | Bact | ALU | |
| | 0 | ALU | Bact | NA | 143 | 6 | 11 | 31.6 | | | 33.1 | 0.95 | | 0 | Bact | ALU | |
| | 0 | ALU | Bact | NA | 182 | 8 | 20 | 33.6 | | | 33.1 | 1.02 | | 0 | Bact | ALU | |

## Supplemental Table S1

| | | | | | | | | | | | | |
|---|---|---|---|---|---|---|---|---|---|---|---|---|
| NA | NA | NA | NA | NA | 34.21 | 0.98 | | | | 0 | Bact | ALU |
| NA | NA | NA | NA | NA | 34.21 | 0.86 | | | | 0 | Bact | ALU |
| NA | NA | NA | NA | NA | 34.21 | 1.00 | | | | 0 | Bact | ALU |
| NA | NA | NA | NA | NA | 34.21 | 0.98 | | | | 0 | Bact | ALU |
| NA | NA | NA | NA | NA | 34.21 | 0.92 | | | | 0 | Bact | ALU |
| NA | NA | NA | NA | NA | NA | NA | | | | 0 | Bact | ALU |
| NA | NA | NA | NA | NA | 34.21 | 1.10 | | | | 0 | Bact | ALU |
| NA | NA | NA | NA | NA | 34.21 | 0.89 | | | | 0 | Bact | ALU |
| NA | NA | NA | NA | NA | NA | NA | | | | 0 | Bact | ALU |
| NA | NA | NA | NA | NA | 34.21 | 1.00 | | | | 0 | Bact | ALU |
| NA | NA | NA | NA | NA | 34.21 | 1.02 | | | | 0 | Bact | ALU |
| NA | NA | NA | NA | NA | 34.21 | 1.01 | | | | 0 | Bact | ALU |
| NA | NA | NA | NA | NA | 34.21 | 0.61 | | | | 0 | Bact | ALU |
| NA | NA | NA | NA | NA | 34.21 | 1.00 | | | | 0 | Bact | ALU |
| NA | NA | NA | NA | NA | 34.21 | 1.05 | | | | 0 | Bact | ALU |
| NA | NA | NA | NA | NA | 34.21 | 1.16 | | | | 0 | Bact | ALU |
| NA | NA | NA | NA | NA | 34.21 | 0.91 | | | | 0 | Bact | ALU |
| NA | NA | NA | NA | NA | 34.21 | 0.81 | | | | 0 | Bact | ALU |
| NA | NA | NA | NA | NA | 34.21 | 0.83 | | | | 0 | Bact | ALU |
| NA | NA | NA | NA | NA | 34.21 | 0.87 | | | | 0 | Bact | ALU |
| NA | NA | NA | NA | NA | 34.21 | 0.92 | | | | 0 | Bact | ALU |
| NA | NA | NA | NA | NA | 34.21 | 0.96 | | | | 0 | Bact | ALU |
| NA | NA | NA | NA | NA | 34.21 | 0.85 | | | | 0 | Bact | ALU |
| NA | NA | NA | NA | NA | 34.21 | 0.81 | | | | 0 | Bact | ALU |
| NA | NA | NA | NA | NA | 34.21 | 1.05 | | | | 0 | Bact | ALU |
| NA | NA | NA | NA | NA | 34.21 | 0.77 | | | | 0 | Bact | ALU |
| NA | NA | NA | NA | NA | 34.21 | 1.12 | | | | 0 | Bact | ALU |
| NA | NA | NA | NA | NA | 34.21 | 1.08 | | | | 0 | Bact | ALU |
| NA | NA | NA | NA | NA | 34.21 | 0.98 | | | | 0 | Bact | ALU |
| NA | NA | NA | NA | NA | 34.21 | 0.90 | | | | 0 | Bact | ALU |
| NA | NA | NA | NA | NA | 34.21 | 1.16 | | | | 0 | Bact | ALU |
| NA | NA | NA | NA | NA | 34.21 | 0.86 | | | | 0 | Bact | ALU |
| NA | NA | NA | NA | NA | 34.21 | 0.97 | | | | 0 | Bact | ALU |
| NA | NA | NA | NA | NA | 34.21 | 0.89 | | | | 0 | Bact | ALU |
| NA | NA | NA | NA | NA | 34.21 | 1.20 | | | | 0 | Bact | ALU |
| NA | NA | NA | NA | NA | 34.21 | 1.23 | | | | 0 | Bact | ALU |
| NA | NA | NA | NA | NA | 34.21 | 0.83 | | | | 0 | Bact | ALU |
| NA | NA | NA | NA | NA | 34.21 | 0.85 | | | | 0 | Bact | ALU |
| NA | NA | NA | NA | NA | 34.21 | 0.98 | | | | 0 | Bact | ALU |
| NA | NA | NA | NA | NA | 34.21 | 0.99 | | | | 0 | Bact | ALU |
| NA | NA | NA | NA | NA | 34.21 | 0.94 | | | | 0 | Bact | ALU |
| NA | NA | NA | NA | NA | NA | NA | | | | 0 | Bact | ALU |
| NA | NA | NA | NA | NA | 34.21 | 0.84 | | | | 0 | Bact | ALU |
| NA | NA | NA | NA | NA | 34.21 | 0.95 | | | | 0 | Bact | ALU |
| NA | NA | NA | NA | NA | 34.21 | 1.01 | | | | 0 | Bact | ALU |
| NA | NA | NA | NA | NA | NA | NA | | | | 0 | Bact | ALU |
| NA | NA | NA | NA | NA | 34.21 | 0.67 | | | | 0 | Bact | ALU |
| NA | NA | NA | NA | NA | 34.21 | 1.13 | | | | 0 | Bact | ALU |
| NA | NA | NA | NA | NA | 34.21 | 0.97 | | | | 0 | Bact | ALU |
| NA | NA | NA | NA | NA | 34.21 | 0.81 | | | | 0 | Bact | ALU |
| NA | NA | NA | NA | NA | NA | NA | | | | 0 | Bact | ALU |
| NA | NA | NA | NA | NA | 34.21 | 0.92 | | | | 0 | Bact | ALU |
| NA | NA | NA | NA | NA | 34.21 | 0.98 | | | | 0 | Bact | ALU |

# Supplemental Table S1

| | | | | | | | | | | | | | | | | | | | |
|---|---|---|---|---|---|---|---|---|---|---|---|---|---|---|---|---|---|---|---|
| | 0 | ALU | Bact | NA | 183 | 1 | 13 | 35.0 | | | | 33.1 | 1.06 | | | 0 | Bact | ALU | |
| | 0 | ALU | Bact | NA | 158 | 3 | 13 | 29.5 | | | | 33.1 | 0.89 | | | 0 | Bact | ALU | |
| | 0 | ALU | Bact | NA | 155 | 6 | 12 | 36.0 | | | | 33.1 | 1.09 | | | 0 | Bact | ALU | |
| | 0 | ALU | Bact | NA | 184 | 3 | 8 | 39.6 | | | | 33.1 | 1.20 | | | 0 | Bact | ALU | |
| | 0 | ALU | Bact | NA | 113 | 5 | 3 | 33.0 | | | | 33.1 | 1.00 | | | 0 | Bact | ALU | |
| | 0 | ALU | Bact | NA | 176 | 6 | 22 | NA | | | | NA | NA | | | 0 | Bact | ALU | |
| | 0 | ALU | Bact | NA | 166 | 7 | 24 | 29.4 | | | | 33.1 | 0.89 | | | 0 | Bact | ALU | |
| | 0 | ALU | Bact | NA | 136 | 4 | 14 | 34.4 | | | | 33.1 | 1.04 | | | 0 | Bact | ALU | |
| | 0 | ALU | Bact | NA | 150 | 5 | 20 | 34.1 | | | | 33.1 | 1.03 | | | 0 | Bact | ALU | |
| | 0 | ALU | Bact | NA | 134 | 8 | 5 | 33.6 | 32.83 | | | 33.1 | 1.02 | 0.992 | | 0 | Bact | ALU | 32.83 |
| | 0 | ALU | Null | NA | 157 | 4 | 21 | NA | | | | NA | NA | | | 0 | Null | ALU | |
| | 0 | ALU | Null | NA | 142 | 1 | 9 | 31.0 | | | | 33.1 | 0.94 | | | 0 | Null | ALU | |
| | 0 | ALU | Null | NA | 152 | 5 | 12 | NA | | | | NA | NA | | | 0 | Null | ALU | |
| | 0 | ALU | Null | NA | 120 | 8 | 3 | 40.7 | | | | 33.1 | 1.23 | | | 0 | Null | ALU | |
| | 0 | ALU | Null | NA | 154 | 1 | 12 | 35.2 | | | | 33.1 | 1.06 | | | 0 | Null | ALU | |
| | 0 | ALU | Null | NA | 128 | 2 | 14 | NA | | | | NA | NA | | | 0 | Null | ALU | |
| | 0 | ALU | Null | NA | 121 | 7 | 6 | 36.2 | | | | 33.1 | 1.09 | | | 0 | Null | ALU | |
| | 0 | ALU | Null | NA | 102 | 6 | 17 | 38.2 | | | | 33.1 | 1.15 | | | 0 | Null | ALU | |
| | 0 | ALU | Null | NA | 164 | 7 | 10 | 34.4 | | | | 33.1 | 1.04 | | | 0 | Null | ALU | |
| | 0 | ALU | Null | NA | 106 | 8 | 24 | 35.6 | | | | 33.1 | 1.08 | | | 0 | Null | ALU | |
| | 0 | ALU | Null | NA | 130 | 1 | 3 | 37.4 | | | | 33.1 | 1.13 | | | 0 | Null | ALU | |
| | 0 | ALU | Null | NA | 117 | 7 | 18 | 34.9 | | | | 33.1 | 1.05 | | | 0 | Null | ALU | |
| | 0 | ALU | Null | NA | 108 | 8 | 14 | 31.8 | | | | 33.1 | 0.96 | | | 0 | Null | ALU | |
| | 0 | ALU | Null | NA | 185 | 4 | 17 | 28.6 | | | | 33.1 | 0.86 | | | 0 | Null | ALU | |
| | 0 | ALU | Null | NA | 165 | 5 | 4 | 25.7 | | | | 33.1 | 0.78 | | | 0 | Null | ALU | |
| | 0 | ALU | Null | NA | 115 | 8 | 9 | 38.2 | | | | 33.1 | 1.15 | | | 0 | Null | ALU | |
| | 0 | ALU | Null | NA | 118 | 6 | 13 | 37.6 | | | | 33.1 | 1.14 | | | 0 | Null | ALU | |
| | 0 | ALU | Null | NA | 179 | 3 | 16 | 31.2 | | | | 33.1 | 0.94 | | | 0 | Null | ALU | |
| | 0 | ALU | Null | NA | 192 | 3 | 2 | 28.0 | | | Grand Average | 33.1 | 0.85 | | | 0 | Null | ALU | |
| | 0 | ALU | Null | NA | 168 | 4 | 8 | 36.8 | 34.21 | | 33.1 | 33.1 | 1.11 | 1.034 | | 0 | Null | ALU | 34.21 |
| 1 | 1 | SOD | Pp | 1 | 65 | 1 | 24 | 24.1 | | | | 18.98 | 1.27 | | | 1 | Pp | SOD | |
| 2 | 1 | SOD | Pp | 1 | 53 | 2 | 7 | 10.1 | | | | 18.98 | 0.53 | | | 1 | Pp | SOD | |
| 3 | 1 | SOD | Pp | 1 | 75 | 3 | 8 | 19.3 | | | | 18.98 | 1.02 | | | 1 | Pp | SOD | |
| 4 | 1 | SOD | Pp | 1 | 89 | 4 | 6 | 25.4 | | | | 18.98 | 1.34 | | | 1 | Pp | SOD | |
| 5 | 1 | SOD | Pp | 1 | 71 | 5 | 21 | NA | | | | NA | NA | | | 1 | Pp | SOD | |
| 6 | 1 | SOD | Pp | 1 | 88 | 6 | 22 | 17.8 | | | | 18.98 | 0.94 | | | 1 | Pp | SOD | |
| 7 | 1 | SOD | Pp | 1 | 70 | 7 | 24 | 25.6 | | | | 18.98 | 1.35 | | | 1 | Pp | SOD | |
| 8 | 1 | SOD | Pp | 1 | 86 | 8 | 19 | NA | 20.38 | | | NA | NA | 1.074 | | 1 | Pp | SOD | |
| 9 | 1 | SOD | Pp | 2 | 97 | 1 | 7 | 21.5 | | | | 18.98 | 1.13 | | | 1 | Pp | SOD | |
| 10 | 1 | SOD | Pp | 2 | 69 | 2 | 14 | 20.2 | | | | 18.98 | 1.06 | | | 1 | Pp | SOD | |
| 11 | 1 | SOD | Pp | 2 | 31 | 3 | 14 | 19.5 | | | | 18.98 | 1.03 | | | 1 | Pp | SOD | |
| 12 | 1 | SOD | Pp | 2 | 61 | 4 | 25 | 19.6 | | | | 18.98 | 1.03 | | | 1 | Pp | SOD | |
| 13 | 1 | SOD | Pp | 2 | 49 | 5 | 5 | 21.7 | | | | 18.98 | 1.14 | | | 1 | Pp | SOD | |
| 14 | 1 | SOD | Pp | 2 | 11 | 6 | 25 | 19.7 | | | | 18.98 | 1.04 | | | 1 | Pp | SOD | |
| 15 | 1 | SOD | Pp | 2 | 66 | 7 | 13 | 19.7 | | | | 18.98 | 1.04 | | | 1 | Pp | SOD | |
| 16 | 1 | SOD | Pp | 2 | 67 | 8 | 3 | 16.0 | 19.74 | | | 18.98 | 0.84 | 1.040 | | 1 | Pp | SOD | |
| 17 | 1 | SOD | Pp | 3 | 3 | 1 | 3 | 21.1 | | | | 18.98 | 1.11 | | | 1 | Pp | SOD | |
| 18 | 1 | SOD | Pp | 3 | 72 | 2 | 21 | 21.4 | | | | 18.98 | 1.13 | | | 1 | Pp | SOD | |
| 19 | 1 | SOD | Pp | 3 | 8 | 3 | 17 | 21.7 | | | | 18.98 | 1.14 | | | 1 | Pp | SOD | |
| 20 | 1 | SOD | Pp | 3 | 2 | 4 | 7 | 22.1 | | | | 18.98 | 1.16 | | | 1 | Pp | SOD | |
| 21 | 1 | SOD | Pp | 3 | 54 | 5 | 12 | 15.3 | | | | 18.98 | 0.81 | | | 1 | Pp | SOD | |
| 22 | 1 | SOD | Pp | 3 | 40 | 6 | 17 | 26.5 | | | | 18.98 | 1.40 | | | 1 | Pp | SOD | |
| 23 | 1 | SOD | Pp | 3 | 42 | 7 | 7 | 22.1 | | | | 18.98 | 1.16 | | | 1 | Pp | SOD | |

# Supplemental Table S1

| | | | | | | | | | | | | |
|---|---|---|---|---|---|---|---|---|---|---|---|---|
| NA | NA | NA | NA | NA | 34.21 | 1.02 | | | | 0 | Bact | ALU |
| NA | NA | NA | NA | NA | 34.21 | 0.86 | | | | 0 | Bact | ALU |
| NA | NA | NA | NA | NA | 34.21 | 1.05 | | | | 0 | Bact | ALU |
| NA | NA | NA | NA | NA | 34.21 | 1.16 | | | | 0 | Bact | ALU |
| NA | NA | NA | NA | NA | 34.21 | 0.96 | | | | 0 | Bact | ALU |
| NA | NA | NA | NA | NA | NA | NA | | | | 0 | Bact | ALU |
| NA | NA | NA | NA | NA | 34.21 | 0.86 | | | | 0 | Bact | ALU |
| NA | NA | NA | NA | NA | 34.21 | 1.01 | | Generation 0 | | 0 | Bact | ALU |
| NA | NA | NA | NA | NA | 34.21 | 1.00 | | Average | StDev | 0 | Bact | ALU |
| NA | NA | NA | NA | NA | 34.21 | 0.98 | | 0.960 | 0.121 | 0 | Bact | ALU |
| NA | NA | NA | NA | NA | NA | NA | | | | 0 | Null | ALU |
| NA | NA | NA | NA | NA | 34.21 | 0.91 | | | | 0 | Null | ALU |
| NA | NA | NA | NA | NA | NA | NA | | | | 0 | Null | ALU |
| NA | NA | NA | NA | NA | 34.21 | 1.19 | | | | 0 | Null | ALU |
| NA | NA | NA | NA | NA | 34.21 | 1.03 | | | | 0 | Null | ALU |
| NA | NA | NA | NA | NA | NA | NA | | | | 0 | Null | ALU |
| NA | NA | NA | NA | NA | 34.21 | 1.06 | | | | 0 | Null | ALU |
| NA | NA | NA | NA | NA | 34.21 | 1.12 | | | | 0 | Null | ALU |
| NA | NA | NA | NA | NA | 34.21 | 1.01 | | | | 0 | Null | ALU |
| NA | NA | NA | NA | NA | 34.21 | 1.04 | | | | 0 | Null | ALU |
| NA | NA | NA | NA | NA | 34.21 | 1.09 | | | | 0 | Null | ALU |
| NA | NA | NA | NA | NA | 34.21 | 1.02 | | | | 0 | Null | ALU |
| NA | NA | NA | NA | NA | 34.21 | 0.93 | | | | 0 | Null | ALU |
| NA | NA | NA | NA | NA | 34.21 | 0.84 | | | | 0 | Null | ALU |
| NA | NA | NA | NA | NA | 34.21 | 0.75 | | | | 0 | Null | ALU |
| NA | NA | NA | NA | NA | 34.21 | 1.12 | | | | 0 | Null | ALU |
| NA | NA | NA | NA | NA | 34.21 | 1.10 | | | | 0 | Null | ALU |
| NA | NA | NA | NA | NA | 34.21 | 0.91 | | | | 0 | Null | ALU |
| NA | NA | NA | NA | NA | 34.21 | 0.82 | | | | 0 | Null | ALU |
| NA | NA | NA | NA | NA | 34.21 | 1.08 | | | | 0 | Null | ALU |
| 16.54 | 1.46 | | | | 22.07 | 1.09 | | | | 1 | Pp | SOD |
| 16.54 | 0.61 | | | | 22.07 | 0.46 | | | | 1 | Pp | SOD |
| 16.54 | 1.17 | | | | 22.07 | 0.87 | | | | 1 | Pp | SOD |
| 16.54 | 1.54 | | | | 22.07 | 1.15 | | | | 1 | Pp | SOD |
| NA | NA | | | | NA | NA | | | | 1 | Pp | SOD |
| 16.54 | 1.08 | | | | 22.07 | 0.81 | | | | 1 | Pp | SOD |
| 16.54 | 1.55 | | | | 22.07 | 1.16 | | | | 1 | Pp | SOD |
| NA | NA | 1.232 | | | NA | NA | 0.924 | | | 1 | Pp | SOD |
| 16.54 | 1.30 | | | | 22.07 | 0.97 | | | | 1 | Pp | SOD |
| 16.54 | 1.22 | | | | 22.07 | 0.92 | | | | 1 | Pp | SOD |
| 16.54 | 1.18 | | | | 22.07 | 0.88 | | | | 1 | Pp | SOD |
| 16.54 | 1.18 | | | | 22.07 | 0.89 | | | | 1 | Pp | SOD |
| 16.54 | 1.31 | | | | 22.07 | 0.98 | | | | 1 | Pp | SOD |
| 16.54 | 1.19 | | | | 22.07 | 0.89 | | | | 1 | Pp | SOD |
| 16.54 | 1.19 | | | | 22.07 | 0.89 | | | | 1 | Pp | SOD |
| 16.54 | 0.97 | 1.193 | | | 22.07 | 0.72 | 0.894 | | | 1 | Pp | SOD |
| 16.54 | 1.28 | | | | 22.07 | 0.96 | | | | 1 | Pp | SOD |
| 16.54 | 1.29 | | | | 22.07 | 0.97 | | | | 1 | Pp | SOD |
| 16.54 | 1.31 | | | | 22.07 | 0.98 | | | | 1 | Pp | SOD |
| 16.54 | 1.34 | | | | 22.07 | 1.00 | | | | 1 | Pp | SOD |
| 16.54 | 0.92 | | | | 22.07 | 0.69 | | | | 1 | Pp | SOD |
| 16.54 | 1.60 | | | | 22.07 | 1.20 | | | | 1 | Pp | SOD |
| 16.54 | 1.34 | | | | 22.07 | 1.00 | | | | 1 | Pp | SOD |

# Supplemental Table S1

| | | | | | | | | | | | | | | | | | | | |
|---|---|---|---|---|---|---|---|---|---|---|---|---|---|---|---|---|---|---|---|
| 24 | 1 | SOD | Pp | 3 | 45 | 8 | 11 | 23.8 | 21.75 | | | 18.98 | 1.25 | 1.146 | | 1 | Pp | SOD | |
| 25 | 1 | SOD | Pp | 4 | 34 | 1 | 14 | 24.5 | | | | 18.98 | 1.29 | | | 1 | Pp | SOD | |
| 26 | 1 | SOD | Pp | 4 | 41 | 2 | 22 | 21.5 | | | | 18.98 | 1.13 | | | 1 | Pp | SOD | |
| 27 | 1 | SOD | Pp | 4 | 26 | 3 | 20 | 22.3 | | | | 18.98 | 1.18 | | | 1 | Pp | SOD | |
| 28 | 1 | SOD | Pp | 4 | 7 | 4 | 23 | NA | | | | NA | NA | | | 1 | Pp | SOD | |
| 29 | 1 | SOD | Pp | 4 | 87 | 5 | 7 | 20.0 | | | | 18.98 | 1.05 | | | 1 | Pp | SOD | |
| 30 | 1 | SOD | Pp | 4 | 33 | 6 | 1 | 19.4 | | | | 18.98 | 1.02 | | | 1 | Pp | SOD | |
| 31 | 1 | SOD | Pp | 4 | 50 | 7 | 14 | 18.6 | | | | 18.98 | 0.98 | | | 1 | Pp | SOD | |
| 32 | 1 | SOD | Pp | 4 | 51 | 8 | 5 | 22.7 | 21.29 | | | 18.98 | 1.20 | 1.122 | | 1 | Pp | SOD | |
| 33 | 1 | SOD | Pp | 5 | 56 | 1 | 15 | 22.1 | | | | 18.98 | 1.16 | | | 1 | Pp | SOD | |
| 34 | 1 | SOD | Pp | 5 | 48 | 2 | 20 | NA | | | | NA | NA | | | 1 | Pp | SOD | |
| 35 | 1 | SOD | Pp | 5 | 78 | 3 | 25 | 18.0 | | | | 18.98 | 0.95 | | | 1 | Pp | SOD | |
| 36 | 1 | SOD | Pp | 5 | 57 | 4 | 4 | 19.5 | | | | 18.98 | 1.03 | | | 1 | Pp | SOD | |
| 37 | 1 | SOD | Pp | 5 | 81 | 5 | 8 | 20.9 | | | | 18.98 | 1.10 | | | 1 | Pp | SOD | |
| 38 | 1 | SOD | Pp | 5 | 24 | 6 | 16 | 20.2 | | | | 18.98 | 1.06 | | | 1 | Pp | SOD | |
| 39 | 1 | SOD | Pp | 5 | 35 | 7 | 2 | 24.3 | | | | 18.98 | 1.28 | | | 1 | Pp | SOD | |
| 40 | 1 | SOD | Pp | 5 | 64 | 8 | 13 | NA | 20.83 | 20.80 | | NA | NA | 1.098 | 1.096 | 1 | Pp | SOD | 20.81 |
| 41 | 1 | SOD | Np | 1 | 6 | 1 | 16 | 16.7 | | | | 18.98 | 0.88 | | | 1 | Np | SOD | |
| 42 | 1 | SOD | Np | 1 | 29 | 2 | 23 | 16.4 | | | | 18.98 | 0.86 | | | 1 | Np | SOD | |
| 43 | 1 | SOD | Np | 1 | 68 | 3 | 6 | 10.7 | | | | 18.98 | 0.56 | | | 1 | Np | SOD | |
| 44 | 1 | SOD | Np | 1 | 93 | 4 | 17 | NA | | | | NA | NA | | | 1 | Np | SOD | |
| 45 | 1 | SOD | Np | 1 | 9 | 5 | 1 | 15.5 | | | | 18.98 | 0.82 | | | 1 | Np | SOD | |
| 46 | 1 | SOD | Np | 1 | 59 | 6 | 2 | 16.6 | | | | 18.98 | 0.87 | | | 1 | Np | SOD | |
| 47 | 1 | SOD | Np | 1 | 38 | 7 | 1 | 8.7 | | | | 18.98 | 0.46 | | | 1 | Np | SOD | |
| 48 | 1 | SOD | Np | 1 | 4 | 8 | 22 | 16.0 | 14.37 | | | 18.98 | 0.84 | 0.757 | | 1 | Np | SOD | |
| 49 | 1 | SOD | Np | 2 | 98 | 1 | 18 | 17.0 | | | | 18.98 | 0.90 | | | 1 | Np | SOD | |
| 50 | 1 | SOD | Np | 2 | 84 | 2 | 16 | 17.4 | | | | 18.98 | 0.92 | | | 1 | Np | SOD | |
| 51 | 1 | SOD | Np | 2 | 18 | 3 | 11 | 20.9 | | | | 18.98 | 1.10 | | | 1 | Np | SOD | |
| 52 | 1 | SOD | Np | 2 | 99 | 4 | 18 | 16.0 | | | | 18.98 | 0.84 | | | 1 | Np | SOD | |
| 53 | 1 | SOD | Np | 2 | 46 | 5 | 4 | 18.8 | | | | 18.98 | 0.99 | | | 1 | Np | SOD | |
| 54 | 1 | SOD | Np | 2 | 90 | 6 | 5 | 20.1 | | | | 18.98 | 1.06 | | | 1 | Np | SOD | |
| 55 | 1 | SOD | Np | 2 | 94 | 7 | 25 | 13.8 | | | | 18.98 | 0.73 | | | 1 | Np | SOD | |
| 56 | 1 | SOD | Np | 2 | 91 | 8 | 7 | 16.9 | 17.61 | | | 18.98 | 0.89 | 0.928 | | 1 | Np | SOD | |
| 57 | 1 | SOD | Np | 3 | 5 | 1 | 10 | 12.4 | | | | 18.98 | 0.65 | | | 1 | Np | SOD | |
| 58 | 1 | SOD | Np | 3 | 100 | 2 | 1 | 11.2 | | | | 18.98 | 0.59 | | | 1 | Np | SOD | |
| 59 | 1 | SOD | Np | 3 | 1 | 3 | 12 | 22.4 | | | | 18.98 | 1.18 | | | 1 | Np | SOD | |
| 60 | 1 | SOD | Np | 3 | 25 | 4 | 11 | 17.2 | | | | 18.98 | 0.91 | | | 1 | Np | SOD | |
| 61 | 1 | SOD | Np | 3 | 39 | 5 | 18 | 18.9 | | | | 18.98 | 1.00 | | | 1 | Np | SOD | |
| 62 | 1 | SOD | Np | 3 | 13 | 6 | 3 | 14.4 | | | | 18.98 | 0.76 | | | 1 | Np | SOD | |
| 63 | 1 | SOD | Np | 3 | 44 | 7 | 9 | 17.8 | | | | 18.98 | 0.94 | | | 1 | Np | SOD | |
| 64 | 1 | SOD | Np | 3 | 28 | 8 | 20 | 13.4 | 15.96 | | | 18.98 | 0.71 | 0.841 | | 1 | Np | SOD | |
| 65 | 1 | SOD | Np | 4 | 32 | 1 | 2 | 19.7 | | | | 18.98 | 1.04 | | | 1 | Np | SOD | |
| 66 | 1 | SOD | Np | 4 | 52 | 2 | 25 | 17.9 | | | | 18.98 | 0.94 | | | 1 | Np | SOD | |
| 67 | 1 | SOD | Np | 4 | 10 | 3 | 10 | 17.3 | | | | 18.98 | 0.91 | | | 1 | Np | SOD | |
| 68 | 1 | SOD | Np | 4 | 77 | 4 | 3 | 17.1 | | | | 18.98 | 0.90 | | | 1 | Np | SOD | |
| 69 | 1 | SOD | Np | 4 | 83 | 5 | 9 | 10.4 | | | | 18.98 | 0.55 | | | 1 | Np | SOD | |
| 70 | 1 | SOD | Np | 4 | 23 | 6 | 11 | 17.0 | | | | 18.98 | 0.90 | | | 1 | Np | SOD | |
| 71 | 1 | SOD | Np | 4 | 30 | 7 | 3 | 17.5 | | | | 18.98 | 0.92 | | | 1 | Np | SOD | |
| 72 | 1 | SOD | Np | 4 | 37 | 8 | 9 | 20.0 | 17.11 | | | 18.98 | 1.05 | 0.902 | | 1 | Np | SOD | |
| 73 | 1 | SOD | Np | 5 | 79 | 1 | 4 | 18.8 | | | | 18.98 | 0.99 | | | 1 | Np | SOD | |
| 74 | 1 | SOD | Np | 5 | 82 | 2 | 12 | 15.7 | | | | 18.98 | 0.83 | | | 1 | Np | SOD | |
| 75 | 1 | SOD | Np | 5 | 76 | 3 | 7 | 11.2 | | | | 18.98 | 0.59 | | | 1 | Np | SOD | |
| 76 | 1 | SOD | Np | 5 | 17 | 4 | 5 | 12.4 | | | | 18.98 | 0.65 | | | 1 | Np | SOD | |

# Supplemental Table S1

| | | | | | | | | | | | | |
|---|---|---|---|---|---|---|---|---|---|---|---|---|
| 16.54 | 1.44 | 1.315 | | | 22.07 | 1.08 | 0.986 | | | 1 | Pp | SOD |
| 16.54 | 1.48 | | | | 22.07 | 1.11 | | | | 1 | Pp | SOD |
| 16.54 | 1.30 | | | | 22.07 | 0.97 | | | | 1 | Pp | SOD |
| 16.54 | 1.35 | | | | 22.07 | 1.01 | | | | 1 | Pp | SOD |
| NA | NA | | | | NA | NA | | | | 1 | Pp | SOD |
| 16.54 | 1.21 | | | | 22.07 | 0.91 | | | | 1 | Pp | SOD |
| 16.54 | 1.17 | | | | 22.07 | 0.88 | | | | 1 | Pp | SOD |
| 16.54 | 1.12 | | | | 22.07 | 0.84 | | | | 1 | Pp | SOD |
| 16.54 | 1.37 | 1.287 | | | 22.07 | 1.03 | 0.964 | | | 1 | Pp | SOD |
| 16.54 | 1.34 | | | | 22.07 | 1.00 | | | | 1 | Pp | SOD |
| NA | NA | | | | NA | NA | | | | 1 | Pp | SOD |
| 16.54 | 1.09 | | | | 22.07 | 0.82 | | | | 1 | Pp | SOD |
| 16.54 | 1.18 | | | | 22.07 | 0.88 | | | | 1 | Pp | SOD |
| 16.54 | 1.26 | | | | 22.07 | 0.95 | | | | 1 | Pp | SOD |
| 16.54 | 1.22 | | Generation 1 | | 22.07 | 0.92 | | Generation 1 | | 1 | Pp | SOD |
| 16.54 | 1.47 | | Average | StDev | 22.07 | 1.10 | | Average | StDev | 1 | Pp | SOD |
| NA | NA | 1.259 | 1.257 | 0.047 | NA | NA | 0.944 | 0.942 | 0.035 | 1 | Pp | SOD |
| | | | | | | | | | | 1 | Np | SOD |
| | | | | | | | | | | 1 | Np | SOD |
| | | | | | | | | | | 1 | Np | SOD |
| | | | | | | | | | | 1 | Np | SOD |
| | | | | | | | | | | 1 | Np | SOD |
| | | | | | | | | | | 1 | Np | SOD |
| | | | | | | | | | | 1 | Np | SOD |
| | | | | | | | | | | 1 | Np | SOD |
| | | | | | | | | | | 1 | Np | SOD |
| | | | | | | | | | | 1 | Np | SOD |
| | | | | | | | | | | 1 | Np | SOD |
| | | | | | | | | | | 1 | Np | SOD |
| | | | | | | | | | | 1 | Np | SOD |
| | | | | | | | | | | 1 | Np | SOD |
| | | | | | | | | | | 1 | Np | SOD |
| | | | | | | | | | | 1 | Np | SOD |
| | | | | | | | | | | 1 | Np | SOD |
| | | | | | | | | | | 1 | Np | SOD |
| | | | | | | | | | | 1 | Np | SOD |
| | | | | | | | | | | 1 | Np | SOD |
| | | | | | | | | | | 1 | Np | SOD |
| | | | | | | | | | | 1 | Np | SOD |
| | | | | | | | | | | 1 | Np | SOD |
| | | | | | | | | | | 1 | Np | SOD |
| | | | | | | | | | | 1 | Np | SOD |
| | | | | | | | | | | 1 | Np | SOD |
| | | | | | | | | | | 1 | Np | SOD |
| | | | | | | | | | | 1 | Np | SOD |
| | | | | | | | | | | 1 | Np | SOD |
| | | | | | | | | | | 1 | Np | SOD |
| | | | | | | | | | | 1 | Np | SOD |
| | | | | | | | | | | 1 | Np | SOD |
| | | | | | | | | | | 1 | Np | SOD |
| | | | | | | | | | | 1 | Np | SOD |
| | | | | | | | | | | 1 | Np | SOD |
| | | | | | | | | | | 1 | Np | SOD |

# Supplemental Table S1

| | | | | | | | | | | | | | | | | | | | |
|---|---|---|---|---|---|---|---|---|---|---|---|---|---|---|---|---|---|---|---|
| 77 | 1 | SOD | Np | 5 | 62 | 5 | 19 | 23.2 | | | | 18.98 | 1.22 | | | 1 | Np | SOD | |
| 78 | 1 | SOD | Np | 5 | 58 | 6 | 13 | 21.7 | | | | 18.98 | 1.14 | | | 1 | Np | SOD | |
| 79 | 1 | SOD | Np | 5 | 55 | 7 | 23 | 19.5 | | | | 18.98 | 1.03 | | | 1 | Np | SOD | |
| 80 | 1 | SOD | Np | 5 | 12 | 8 | 2 | 16.5 | 17.38 | 16.49 | | 18.98 | 0.87 | 0.916 | 0.869 | 1 | Np | SOD | 16.54 |
| 91 | 1 | SOD | Null | NA | 96 | 1 | 21 | 21.6 | | | | 18.98 | 1.14 | | | 1 | Null | SOD | |
| 92 | 1 | SOD | Null | NA | 22 | 2 | 15 | 24.5 | | | | 18.98 | 1.29 | | | 1 | Null | SOD | |
| 93 | 1 | SOD | Null | NA | 95 | 3 | 4 | 22.9 | | | | 18.98 | 1.21 | | | 1 | Null | SOD | |
| 94 | 1 | SOD | Null | NA | 85 | 4 | 12 | 20.8 | | | | 18.98 | 1.10 | | | 1 | Null | SOD | |
| 95 | 1 | SOD | Null | NA | 15 | 5 | 17 | 18.6 | | | | 18.98 | 0.98 | | | 1 | Null | SOD | |
| 96 | 1 | SOD | Null | NA | 16 | 6 | 19 | 24.6 | | | | 18.98 | 1.30 | | | 1 | Null | SOD | |
| 97 | 1 | SOD | Null | NA | 73 | 7 | 11 | 17.3 | | | | 18.98 | 0.91 | | | 1 | Null | SOD | |
| 98 | 1 | SOD | Null | NA | 60 | 8 | 12 | 24.6 | | | | 18.98 | 1.30 | | | 1 | Null | SOD | |
| 99 | 1 | SOD | Null | NA | 74 | 3 | 16 | 25.2 | | | Grand Average | 18.98 | 1.33 | | | 1 | Null | SOD | |
| 100 | 1 | SOD | Null | NA | 14 | 8 | 23 | 20.6 | 22.07 | | 18.98 | 18.98 | 1.09 | 1.163 | | 1 | Null | SOD | 22.07 |
| 101 | 1 | ALU | Pp | 1 | 183 | 1 | 6 | 9.0 | | | | 20.16 | 0.45 | | | 1 | Pp | ALU | |
| 102 | 1 | ALU | Pp | 1 | 190 | 2 | 17 | 19.5 | | | | 20.16 | 0.97 | | | 1 | Pp | ALU | |
| 103 | 1 | ALU | Pp | 1 | 139 | 3 | 18 | 24.6 | | | | 20.16 | 1.22 | | | 1 | Pp | ALU | |
| 104 | 1 | ALU | Pp | 1 | 149 | 4 | 24 | 28.1 | | | | 20.16 | 1.39 | | | 1 | Pp | ALU | |
| 105 | 1 | ALU | Pp | 1 | 152 | 5 | 10 | 21.6 | | | | 20.16 | 1.07 | | | 1 | Pp | ALU | |
| 106 | 1 | ALU | Pp | 1 | 146 | 6 | 4 | 18.6 | | | | 20.16 | 0.92 | | | 1 | Pp | ALU | |
| 107 | 1 | ALU | Pp | 1 | 192 | 7 | 22 | NA | | | | NA | NA | | | 1 | Pp | ALU | |
| 108 | 1 | ALU | Pp | 1 | 188 | 8 | 24 | 25.1 | 20.93 | | | 20.16 | 1.24 | 1.038 | | 1 | Pp | ALU | |
| 109 | 1 | ALU | Pp | 2 | 131 | 1 | 12 | 24.7 | | | | 20.16 | 1.23 | | | 1 | Pp | ALU | |
| 110 | 1 | ALU | Pp | 2 | 128 | 2 | 6 | 27.7 | | | | 20.16 | 1.37 | | | 1 | Pp | ALU | |
| 111 | 1 | ALU | Pp | 2 | 122 | 3 | 19 | 20.6 | | | | 20.16 | 1.02 | | | 1 | Pp | ALU | |
| 112 | 1 | ALU | Pp | 2 | 159 | 4 | 22 | 19.7 | | | | 20.16 | 0.98 | | | 1 | Pp | ALU | |
| 113 | 1 | ALU | Pp | 2 | 196 | 5 | 20 | NA | | | | NA | NA | | | 1 | Pp | ALU | |
| 114 | 1 | ALU | Pp | 2 | 123 | 6 | 9 | NA | | | | NA | NA | | | 1 | Pp | ALU | |
| 115 | 1 | ALU | Pp | 2 | 127 | 7 | 5 | 22.3 | | | | 20.16 | 1.11 | | | 1 | Pp | ALU | |
| 116 | 1 | ALU | Pp | 2 | 138 | 8 | 15 | 19.3 | 22.38 | | | 20.16 | 0.96 | 1.110 | | 1 | Pp | ALU | |
| 117 | 1 | ALU | Pp | 3 | 160 | 1 | 23 | 11.2 | | | | 20.16 | 0.56 | | | 1 | Pp | ALU | |
| 118 | 1 | ALU | Pp | 3 | 187 | 2 | 4 | 14.3 | | | | 20.16 | 0.71 | | | 1 | Pp | ALU | |
| 119 | 1 | ALU | Pp | 3 | 162 | 3 | 22 | 25.7 | | | | 20.16 | 1.27 | | | 1 | Pp | ALU | |
| 120 | 1 | ALU | Pp | 3 | 110 | 4 | 15 | 27.5 | | | | 20.16 | 1.36 | | | 1 | Pp | ALU | |
| 121 | 1 | ALU | Pp | 3 | 125 | 5 | 11 | 22.1 | | | | 20.16 | 1.10 | | | 1 | Pp | ALU | |
| 122 | 1 | ALU | Pp | 3 | 191 | 6 | 21 | 23.8 | | | | 20.16 | 1.18 | | | 1 | Pp | ALU | |
| 123 | 1 | ALU | Pp | 3 | 165 | 7 | 4 | 21.0 | | | | 20.16 | 1.04 | | | 1 | Pp | ALU | |
| 124 | 1 | ALU | Pp | 3 | 151 | 8 | 8 | 25.4 | 21.38 | | | 20.16 | 1.26 | 1.060 | | 1 | Pp | ALU | |
| 125 | 1 | ALU | Pp | 4 | 120 | 1 | 19 | 24.0 | | | | 20.16 | 1.19 | | | 1 | Pp | ALU | |
| 126 | 1 | ALU | Pp | 4 | 129 | 2 | 9 | 25.2 | | | | 20.16 | 1.25 | | | 1 | Pp | ALU | |
| 127 | 1 | ALU | Pp | 4 | 168 | 3 | 2 | 23.5 | | | | 20.16 | 1.17 | | | 1 | Pp | ALU | |
| 128 | 1 | ALU | Pp | 4 | 142 | 4 | 10 | 17.1 | | | | 20.16 | 0.85 | | | 1 | Pp | ALU | |
| 129 | 1 | ALU | Pp | 4 | 137 | 5 | 24 | 19.6 | | | | 20.16 | 0.97 | | | 1 | Pp | ALU | |
| 130 | 1 | ALU | Pp | 4 | 181 | 6 | 8 | NA | | | | NA | NA | | | 1 | Pp | ALU | |
| 131 | 1 | ALU | Pp | 4 | 166 | 7 | 19 | 23.8 | | | | 20.16 | 1.18 | | | 1 | Pp | ALU | |
| 132 | 1 | ALU | Pp | 4 | 194 | 8 | 18 | 26.0 | 22.74 | | | 20.16 | 1.29 | 1.128 | | 1 | Pp | ALU | |
| 133 | 1 | ALU | Pp | 5 | 150 | 1 | 22 | 25.9 | | | | 20.16 | 1.28 | | | 1 | Pp | ALU | |
| 134 | 1 | ALU | Pp | 5 | 174 | 2 | 13 | 24.4 | | | | 20.16 | 1.21 | | | 1 | Pp | ALU | |
| 135 | 1 | ALU | Pp | 5 | 133 | 3 | 5 | 22.0 | | | | 20.16 | 1.09 | | | 1 | Pp | ALU | |
| 136 | 1 | ALU | Pp | 5 | 154 | 4 | 19 | 23.4 | | | | 20.16 | 1.16 | | | 1 | Pp | ALU | |
| 137 | 1 | ALU | Pp | 5 | 126 | 5 | 14 | 17.1 | | | | 20.16 | 0.85 | | | 1 | Pp | ALU | |
| 138 | 1 | ALU | Pp | 5 | 161 | 6 | 7 | NA | | | | NA | NA | | | 1 | Pp | ALU | |
| 139 | 1 | ALU | Pp | 5 | 167 | 7 | 6 | 21.0 | | | | 20.16 | 1.04 | | | 1 | Pp | ALU | |

# Supplemental Table S1

| | | | | | | | | | | | | |
|---|---|---|---|---|---|---|---|---|---|---|---|---|
| | | | | | | | | | | 1 | Np | SOD |
| | | | | | | | | | | 1 | Np | SOD |
| | | | | | | | | | | 1 | Np | SOD |
| | | | | | | | | | | 1 | Np | SOD |
| | | | | | | | | | | 1 | Null | SOD |
| | | | | | | | | | | 1 | Null | SOD |
| | | | | | | | | | | 1 | Null | SOD |
| | | | | | | | | | | 1 | Null | SOD |
| | | | | | | | | | | 1 | Null | SOD |
| | | | | | | | | | | 1 | Null | SOD |
| | | | | | | | | | | 1 | Null | SOD |
| | | | | | | | | | | 1 | Null | SOD |
| | | | | | | | | | | 1 | Null | SOD |
| | | | | | | | | | | 1 | Null | SOD |
| 17.81 | 0.51 | | | | 22.32 | 0.40 | | | | 1 | Pp | ALU |
| 17.81 | 1.10 | | | | 22.32 | 0.87 | | | | 1 | Pp | ALU |
| 17.81 | 1.38 | | | | 22.32 | 1.10 | | | | 1 | Pp | ALU |
| 17.81 | 1.58 | | | | 22.32 | 1.26 | | | | 1 | Pp | ALU |
| 17.81 | 1.21 | | | | 22.32 | 0.97 | | | | 1 | Pp | ALU |
| 17.81 | 1.04 | | | | 22.32 | 0.83 | | | | 1 | Pp | ALU |
| NA | NA | | | | NA | NA | | | | 1 | Pp | ALU |
| 17.81 | 1.41 | 1.18 | | | 22.32 | 1.12 | 0.938 | | | 1 | Pp | ALU |
| 17.81 | 1.39 | | | | 22.32 | 1.11 | | | | 1 | Pp | ALU |
| 17.81 | 1.56 | | | | 22.32 | 1.24 | | | | 1 | Pp | ALU |
| 17.81 | 1.16 | | | | 22.32 | 0.92 | | | | 1 | Pp | ALU |
| 17.81 | 1.11 | | | | 22.32 | 0.88 | | | | 1 | Pp | ALU |
| NA | NA | | | | NA | NA | | | | 1 | Pp | ALU |
| NA | NA | | | | NA | NA | | | | 1 | Pp | ALU |
| 17.81 | 1.25 | | | | 22.32 | 1.00 | | | | 1 | Pp | ALU |
| 17.81 | 1.08 | 1.26 | | | 22.32 | 0.86 | 1.003 | | | 1 | Pp | ALU |
| 17.81 | 0.63 | | | | 22.32 | 0.50 | | | | 1 | Pp | ALU |
| 17.81 | 0.80 | | | | 22.32 | 0.64 | | | | 1 | Pp | ALU |
| 17.81 | 1.44 | | | | 22.32 | 1.15 | | | | 1 | Pp | ALU |
| 17.81 | 1.54 | | | | 22.32 | 1.23 | | | | 1 | Pp | ALU |
| 17.81 | 1.24 | | | | 22.32 | 0.99 | | | | 1 | Pp | ALU |
| 17.81 | 1.34 | | | | 22.32 | 1.07 | | | | 1 | Pp | ALU |
| 17.81 | 1.18 | | | | 22.32 | 0.94 | | | | 1 | Pp | ALU |
| 17.81 | 1.43 | 1.20 | | | 22.32 | 1.14 | 0.958 | | | 1 | Pp | ALU |
| 17.81 | 1.35 | | | | 22.32 | 1.08 | | | | 1 | Pp | ALU |
| 17.81 | 1.42 | | | | 22.32 | 1.13 | | | | 1 | Pp | ALU |
| 17.81 | 1.32 | | | | 22.32 | 1.05 | | | | 1 | Pp | ALU |
| 17.81 | 0.96 | | | | 22.32 | 0.77 | | | | 1 | Pp | ALU |
| 17.81 | 1.10 | | | | 22.32 | 0.88 | | | | 1 | Pp | ALU |
| NA | NA | | | | NA | NA | | | | 1 | Pp | ALU |
| 17.81 | 1.34 | | | | 22.32 | 1.07 | | | | 1 | Pp | ALU |
| 17.81 | 1.46 | 1.28 | | | 22.32 | 1.16 | 1.019 | | | 1 | Pp | ALU |
| 17.81 | 1.45 | | | | 22.32 | 1.16 | | | | 1 | Pp | ALU |
| 17.81 | 1.37 | | | | 22.32 | 1.09 | | | | 1 | Pp | ALU |
| 17.81 | 1.24 | | | | 22.32 | 0.99 | | | | 1 | Pp | ALU |
| 17.81 | 1.31 | | | | 22.32 | 1.05 | | | | 1 | Pp | ALU |
| 17.81 | 0.96 | | | | 22.32 | 0.77 | | | | 1 | Pp | ALU |
| NA | NA | | Generation 1 | | NA | NA | | Generation 1 | | 1 | Pp | ALU |
| 17.81 | 1.18 | | Average | StDev | 22.32 | 0.94 | | Average | StDev | 1 | Pp | ALU |

# Supplemental Table S1

| | | | | | | | | | | | | | | | | | | | |
|---|---|---|---|---|---|---|---|---|---|---|---|---|---|---|---|---|---|---|---|
| 140 | 1 | ALU | Pp | 5 | 147 | 8 | 25 | 26.4 | 22.89 | 22.06 | | 20.16 | 1.31 | 1.135 | 1.094 | 1 | Pp | ALU | 22.03 |
| 141 | 1 | ALU | Np | 1 | 169 | 1 | 17 | 18.1 | | | | 20.16 | 0.90 | | | 1 | Np | ALU | |
| 142 | 1 | ALU | Np | 1 | 105 | 2 | 3 | 15.4 | | | | 20.16 | 0.76 | | | 1 | Np | ALU | |
| 143 | 1 | ALU | Np | 1 | 113 | 3 | 3 | NA | | | | NA | NA | | | 1 | Np | ALU | |
| 144 | 1 | ALU | Np | 1 | 104 | 4 | 14 | 19.5 | | | | 20.16 | 0.97 | | | 1 | Np | ALU | |
| 145 | 1 | ALU | Np | 1 | 134 | 5 | 2 | 18.6 | | | | 20.16 | 0.92 | | | 1 | Np | ALU | |
| 146 | 1 | ALU | Np | 1 | 182 | 6 | 20 | 16.2 | | | | 20.16 | 0.80 | | | 1 | Np | ALU | |
| 147 | 1 | ALU | Np | 1 | 144 | 7 | 15 | 21.6 | | | | 20.16 | 1.07 | | | 1 | Np | ALU | |
| 148 | 1 | ALU | Np | 1 | 141 | 8 | 16 | 7.0 | 16.63 | | | 20.16 | 0.35 | 0.825 | | 1 | Np | ALU | |
| 149 | 1 | ALU | Np | 2 | 115 | 1 | 9 | 19.6 | | | | 20.16 | 0.97 | | | 1 | Np | ALU | |
| 150 | 1 | ALU | Np | 2 | 163 | 2 | 11 | 20.5 | | | | 20.16 | 1.02 | | | 1 | Np | ALU | |
| 151 | 1 | ALU | Np | 2 | 106 | 3 | 15 | 17.1 | | | | 20.16 | 0.85 | | | 1 | Np | ALU | |
| 152 | 1 | ALU | Np | 2 | 177 | 4 | 1 | 20.0 | | | | 20.16 | 0.99 | | | 1 | Np | ALU | |
| 153 | 1 | ALU | Np | 2 | 197 | 5 | 15 | 14.5 | | | | 20.16 | 0.72 | | | 1 | Np | ALU | |
| 154 | 1 | ALU | Np | 2 | 136 | 6 | 14 | 18.3 | | | | 20.16 | 0.91 | | | 1 | Np | ALU | |
| 155 | 1 | ALU | Np | 2 | 155 | 7 | 12 | 18.6 | | | | 20.16 | 0.92 | | | 1 | Np | ALU | |
| 156 | 1 | ALU | Np | 2 | 170 | 8 | 21 | 22.2 | 18.85 | | | 20.16 | 1.10 | 0.935 | | 1 | Np | ALU | |
| 157 | 1 | ALU | Np | 3 | 112 | 1 | 13 | NA | | | | NA | NA | | | 1 | Np | ALU | |
| 158 | 1 | ALU | Np | 3 | 200 | 2 | 10 | 20.4 | | | | 20.16 | 1.01 | | | 1 | Np | ALU | |
| 159 | 1 | ALU | Np | 3 | 101 | 3 | 21 | 19.1 | | | | 20.16 | 0.95 | | | 1 | Np | ALU | |
| 160 | 1 | ALU | Np | 3 | 178 | 4 | 21 | 19.5 | | | | 20.16 | 0.97 | | | 1 | Np | ALU | |
| 161 | 1 | ALU | Np | 3 | 102 | 5 | 6 | 18.7 | | | | 20.16 | 0.93 | | | 1 | Np | ALU | |
| 162 | 1 | ALU | Np | 3 | 140 | 6 | 23 | 17.3 | | | | 20.16 | 0.86 | | | 1 | Np | ALU | |
| 163 | 1 | ALU | Np | 3 | 153 | 7 | 8 | 19.1 | | | | 20.16 | 0.95 | | | 1 | Np | ALU | |
| 164 | 1 | ALU | Np | 3 | 158 | 8 | 4 | 20.2 | 19.19 | | | 20.16 | 1.00 | 0.952 | | 1 | Np | ALU | |
| 165 | 1 | ALU | Np | 4 | 184 | 1 | 25 | 16.1 | | | | 20.16 | 0.80 | | | 1 | Np | ALU | |
| 166 | 1 | ALU | Np | 4 | 107 | 2 | 2 | 19.1 | | | | 20.16 | 0.95 | | | 1 | Np | ALU | |
| 167 | 1 | ALU | Np | 4 | 109 | 3 | 24 | 14.0 | | | | 20.16 | 0.69 | | | 1 | Np | ALU | |
| 168 | 1 | ALU | Np | 4 | 180 | 4 | 2 | 17.9 | | | | 20.16 | 0.89 | | | 1 | Np | ALU | |
| 169 | 1 | ALU | Np | 4 | 118 | 5 | 25 | 12.2 | | | | 20.16 | 0.61 | | | 1 | Np | ALU | |
| 170 | 1 | ALU | Np | 4 | 114 | 6 | 12 | 17.1 | | | | 20.16 | 0.85 | | | 1 | Np | ALU | |
| 171 | 1 | ALU | Np | 4 | 124 | 7 | 17 | 21.0 | | | | 20.16 | 1.04 | | | 1 | Np | ALU | |
| 172 | 1 | ALU | Np | 4 | 185 | 8 | 17 | 20.7 | 17.26 | | | 20.16 | 1.03 | 0.856 | | 1 | Np | ALU | |
| 173 | 1 | ALU | Np | 5 | 189 | 1 | 8 | NA | | | | NA | NA | | | 1 | Np | ALU | |
| 174 | 1 | ALU | Np | 5 | 148 | 2 | 5 | 15.9 | | | | 20.16 | 0.79 | | | 1 | Np | ALU | |
| 175 | 1 | ALU | Np | 5 | 176 | 3 | 9 | 15.9 | | | | 20.16 | 0.79 | | | 1 | Np | ALU | |
| 176 | 1 | ALU | Np | 5 | 117 | 4 | 16 | 14.9 | | | | 20.16 | 0.74 | | | 1 | Np | ALU | |
| 177 | 1 | ALU | Np | 5 | 199 | 5 | 13 | 13.5 | | | | 20.16 | 0.67 | | | 1 | Np | ALU | |
| 178 | 1 | ALU | Np | 5 | 111 | 6 | 10 | 22.1 | | | | 20.16 | 1.10 | | | 1 | Np | ALU | |
| 179 | 1 | ALU | Np | 5 | 172 | 7 | 16 | 18.8 | | | | 20.16 | 0.93 | | | 1 | Np | ALU | |
| 180 | 1 | ALU | Np | 5 | 143 | 8 | 6 | 18.1 | 17.03 | 17.79 | | 20.16 | 0.90 | 0.845 | 0.882 | 1 | Np | ALU | 17.81 |
| 191 | 1 | ALU | Null | NA | 119 | 1 | 5 | 21.1 | | | | 20.16 | 1.05 | | | 1 | Null | ALU | |
| 192 | 1 | ALU | Null | NA | 145 | 2 | 18 | 27.9 | | | | 20.16 | 1.38 | | | 1 | Null | ALU | |
| 193 | 1 | ALU | Null | NA | 130 | 3 | 23 | 22.2 | | | | 20.16 | 1.10 | | | 1 | Null | ALU | |
| 194 | 1 | ALU | Null | NA | 108 | 4 | 20 | 18.1 | | | | 20.16 | 0.90 | | | 1 | Null | ALU | |
| 195 | 1 | ALU | Null | NA | 156 | 5 | 22 | 21.6 | | | | 20.16 | 1.07 | | | 1 | Null | ALU | |
| 196 | 1 | ALU | Null | NA | 195 | 6 | 6 | 20.5 | | | | 20.16 | 1.02 | | | 1 | Null | ALU | |
| 197 | 1 | ALU | Null | NA | 164 | 7 | 21 | 20.2 | | | | 20.16 | 1.00 | | | 1 | Null | ALU | |
| 198 | 1 | ALU | Null | NA | 121 | 8 | 10 | 23.0 | | | | 20.16 | 1.14 | | | 1 | Null | ALU | |
| 199 | 1 | ALU | Null | NA | 186 | 2 | 19 | 26.0 | | | Grand Average | 20.16 | 1.29 | | | 1 | Null | ALU | |
| 200 | 1 | ALU | Null | NA | 103 | 5 | 23 | 22.6 | 22.32 | | 20.16 | 20.16 | 1.12 | 1.107 | | 1 | Null | ALU | 22.32 |
| 1 | 2 | SOD | Pp | 1 | 79 | 1 | 24 | 27.5 | | | | 19.25 | 1.43 | | | 2 | Pp | SOD | |
| 2 | 2 | SOD | Pp | 1 | 100 | 2 | 22 | 23.2 | | | | 19.25 | 1.21 | | | 2 | Pp | SOD | |

# Supplemental Table S1

| | | | | | | | | | | | | |
|---|---|---|---|---|---|---|---|---|---|---|---|---|
| 17.81 | 1.48 | 1.29 | 1.239 | 0.049 | 22.32 | 1.18 | 1.025 | 0.988 | 0.039 | 1 | Pp | ALU |
| | | | | | | | | | | 1 | Np | ALU |
| | | | | | | | | | | 1 | Np | ALU |
| | | | | | | | | | | 1 | Np | ALU |
| | | | | | | | | | | 1 | Np | ALU |
| | | | | | | | | | | 1 | Np | ALU |
| | | | | | | | | | | 1 | Np | ALU |
| | | | | | | | | | | 1 | Np | ALU |
| | | | | | | | | | | 1 | Np | ALU |
| | | | | | | | | | | 1 | Np | ALU |
| | | | | | | | | | | 1 | Np | ALU |
| | | | | | | | | | | 1 | Np | ALU |
| | | | | | | | | | | 1 | Np | ALU |
| | | | | | | | | | | 1 | Np | ALU |
| | | | | | | | | | | 1 | Np | ALU |
| | | | | | | | | | | 1 | Np | ALU |
| | | | | | | | | | | 1 | Np | ALU |
| | | | | | | | | | | 1 | Np | ALU |
| | | | | | | | | | | 1 | Np | ALU |
| | | | | | | | | | | 1 | Np | ALU |
| | | | | | | | | | | 1 | Np | ALU |
| | | | | | | | | | | 1 | Np | ALU |
| | | | | | | | | | | 1 | Np | ALU |
| | | | | | | | | | | 1 | Np | ALU |
| | | | | | | | | | | 1 | Np | ALU |
| | | | | | | | | | | 1 | Np | ALU |
| | | | | | | | | | | 1 | Np | ALU |
| | | | | | | | | | | 1 | Np | ALU |
| | | | | | | | | | | 1 | Np | ALU |
| | | | | | | | | | | 1 | Np | ALU |
| | | | | | | | | | | 1 | Np | ALU |
| | | | | | | | | | | 1 | Np | ALU |
| | | | | | | | | | | 1 | Np | ALU |
| | | | | | | | | | | 1 | Np | ALU |
| | | | | | | | | | | 1 | Np | ALU |
| | | | | | | | | | | 1 | Np | ALU |
| | | | | | | | | | | 1 | Np | ALU |
| | | | | | | | | | | 1 | Np | ALU |
| | | | | | | | | | | 1 | Np | ALU |
| | | | | | | | | | | 1 | Np | ALU |
| | | | | | | | | | | 1 | Np | ALU |
| | | | | | | | | | | 1 | Null | ALU |
| | | | | | | | | | | 1 | Null | ALU |
| | | | | | | | | | | 1 | Null | ALU |
| | | | | | | | | | | 1 | Null | ALU |
| | | | | | | | | | | 1 | Null | ALU |
| | | | | | | | | | | 1 | Null | ALU |
| | | | | | | | | | | 1 | Null | ALU |
| | | | | | | | | | | 1 | Null | ALU |
| | | | | | | | | | | 1 | Null | ALU |
| | | | | | | | | | | 1 | Null | ALU |
| 12.37 | 2.22 | | | | 22.60 | 1.22 | | | | 2 | Pp | SOD |
| 12.37 | 1.88 | | | | 22.60 | 1.03 | | | | 2 | Pp | SOD |

# Supplemental Table S1

| | | | | | | | | | | | | | | | | | | | |
|---|---|---|---|---|---|---|---|---|---|---|---|---|---|---|---|---|---|---|---|
| 3 | 2 | SOD | Pp | 1 | 26 | 3 | 17 | NA | | | | NA | NA | | | 2 | Pp | SOD | |
| 4 | 2 | SOD | Pp | 1 | 6 | 4 | 10 | 23.3 | | | | 19.25 | 1.21 | | | 2 | Pp | SOD | |
| 5 | 2 | SOD | Pp | 1 | 29 | 5 | 16 | 22.1 | | | | 19.25 | 1.15 | | | 2 | Pp | SOD | |
| 6 | 2 | SOD | Pp | 1 | 98 | 6 | 7 | 25.5 | | | | 19.25 | 1.32 | | | 2 | Pp | SOD | |
| 7 | 2 | SOD | Pp | 1 | 46 | 7 | 21 | 23.9 | | | | 19.25 | 1.24 | | | 2 | Pp | SOD | |
| 8 | 2 | SOD | Pp | 1 | 5 | 8 | 3 | 27.2 | 24.67 | | | 19.25 | 1.41 | 1.282 | | 2 | Pp | SOD | |
| 9 | 2 | SOD | Pp | 2 | 96 | 1 | 20 | 25.5 | | | | 19.25 | 1.32 | | | 2 | Pp | SOD | |
| 10 | 2 | SOD | Pp | 2 | 22 | 2 | 11 | 21.5 | | | | 19.25 | 1.12 | | | 2 | Pp | SOD | |
| 11 | 2 | SOD | Pp | 2 | 14 | 3 | 19 | 27.6 | | | | 19.25 | 1.43 | | | 2 | Pp | SOD | |
| 12 | 2 | SOD | Pp | 2 | 49 | 4 | 11 | NA | | | | NA | NA | | | 2 | Pp | SOD | |
| 13 | 2 | SOD | Pp | 2 | 38 | 5 | 2 | 25.7 | | | | 19.25 | 1.34 | | | 2 | Pp | SOD | |
| 14 | 2 | SOD | Pp | 2 | 78 | 6 | 3 | 26.7 | | | | 19.25 | 1.39 | | | 2 | Pp | SOD | |
| 15 | 2 | SOD | Pp | 2 | 30 | 7 | 20 | 21.9 | | | | 19.25 | 1.14 | | | 2 | Pp | SOD | |
| 16 | 2 | SOD | Pp | 2 | 11 | 8 | 19 | 23.3 | 24.60 | | | 19.25 | 1.21 | 1.278 | | 2 | Pp | SOD | |
| 17 | 2 | SOD | Pp | 3 | 54 | 1 | 1 | 26.5 | | | | 19.25 | 1.38 | | | 2 | Pp | SOD | |
| 18 | 2 | SOD | Pp | 3 | 13 | 2 | 10 | NA | | | | NA | NA | | | 2 | Pp | SOD | |
| 19 | 2 | SOD | Pp | 3 | 80 | 3 | 8 | 29.1 | | | | 19.25 | 1.51 | | | 2 | Pp | SOD | |
| 20 | 2 | SOD | Pp | 3 | 92 | 4 | 4 | 25.7 | | | | 19.25 | 1.34 | | | 2 | Pp | SOD | |
| 21 | 2 | SOD | Pp | 3 | 39 | 5 | 5 | 27.5 | | | | 19.25 | 1.43 | | | 2 | Pp | SOD | |
| 22 | 2 | SOD | Pp | 3 | 88 | 6 | 21 | 25.3 | | | | 19.25 | 1.31 | | | 2 | Pp | SOD | |
| 23 | 2 | SOD | Pp | 3 | 18 | 7 | 4 | 26.3 | | | | 19.25 | 1.37 | | | 2 | Pp | SOD | |
| 24 | 2 | SOD | Pp | 3 | 9 | 8 | 7 | 25.0 | 26.49 | | | 19.25 | 1.30 | 1.376 | | 2 | Pp | SOD | |
| 25 | 2 | SOD | Pp | 4 | 21 | 1 | 10 | 25.9 | | | | 19.25 | 1.35 | | | 2 | Pp | SOD | |
| 26 | 2 | SOD | Pp | 4 | 60 | 2 | 25 | 30.5 | | | | 19.25 | 1.58 | | | 2 | Pp | SOD | |
| 27 | 2 | SOD | Pp | 4 | 47 | 3 | 2 | 25.4 | | | | 19.25 | 1.32 | | | 2 | Pp | SOD | |
| 28 | 2 | SOD | Pp | 4 | 43 | 4 | 8 | 28.9 | | | | 19.25 | 1.50 | | | 2 | Pp | SOD | |
| 29 | 2 | SOD | Pp | 4 | 12 | 5 | 7 | 21.7 | | | | 19.25 | 1.13 | | | 2 | Pp | SOD | |
| 30 | 2 | SOD | Pp | 4 | 42 | 6 | 16 | 23.3 | | | | 19.25 | 1.21 | | | 2 | Pp | SOD | |
| 31 | 2 | SOD | Pp | 4 | 99 | 7 | 23 | 25.7 | | | | 19.25 | 1.34 | | | 2 | Pp | SOD | |
| 32 | 2 | SOD | Pp | 4 | 25 | 8 | 11 | 25.7 | 25.89 | | | 19.25 | 1.34 | 1.345 | | 2 | Pp | SOD | |
| 33 | 2 | SOD | Pp | 5 | 69 | 1 | 15 | 25.1 | | | | 19.25 | 1.30 | | | 2 | Pp | SOD | |
| 34 | 2 | SOD | Pp | 5 | 95 | 2 | 6 | 19.8 | | | | 19.25 | 1.03 | | | 2 | Pp | SOD | |
| 35 | 2 | SOD | Pp | 5 | 36 | 3 | 15 | 20.5 | | | | 19.25 | 1.07 | | | 2 | Pp | SOD | |
| 36 | 2 | SOD | Pp | 5 | 66 | 4 | 3 | 23.2 | | | | 19.25 | 1.21 | | | 2 | Pp | SOD | |
| 37 | 2 | SOD | Pp | 5 | 89 | 5 | 23 | 24.6 | | | | 19.25 | 1.28 | | | 2 | Pp | SOD | |
| 38 | 2 | SOD | Pp | 5 | 97 | 6 | 17 | 22.2 | | | | 19.25 | 1.15 | | | 2 | Pp | SOD | |
| 39 | 2 | SOD | Pp | 5 | 59 | 7 | 2 | 25.5 | | | | 19.25 | 1.32 | | | 2 | Pp | SOD | |
| 40 | 2 | SOD | Pp | 5 | 63 | 8 | 20 | 21.0 | 22.74 | 24.88 | | 19.25 | 1.09 | 1.181 | 1.292 | 2 | Pp | SOD | 24.85 |
| 41 | 2 | SOD | Np | 1 | 81 | 1 | 18 | 17.6 | | | | 19.25 | 0.91 | | | 2 | Np | SOD | |
| 42 | 2 | SOD | Np | 1 | 61 | 2 | 18 | NA | | | | NA | NA | | | 2 | Np | SOD | |
| 43 | 2 | SOD | Np | 1 | 76 | 3 | 21 | 0.8 | | | | 19.25 | 0.04 | | | 2 | Np | SOD | |
| 44 | 2 | SOD | Np | 1 | 48 | 4 | 16 | 15.9 | | | | 19.25 | 0.83 | | | 2 | Np | SOD | |
| 45 | 2 | SOD | Np | 1 | 57 | 5 | 14 | 16.8 | | | | 19.25 | 0.87 | | | 2 | Np | SOD | |
| 46 | 2 | SOD | Np | 1 | 24 | 6 | 15 | 14.9 | | | | 19.25 | 0.77 | | | 2 | Np | SOD | |
| 47 | 2 | SOD | Np | 1 | 90 | 7 | 11 | 18.4 | | | | 19.25 | 0.96 | | | 2 | Np | SOD | |
| 48 | 2 | SOD | Np | 1 | 34 | 8 | 22 | 1.4 | 12.26 | | | 19.25 | 0.07 | 0.637 | | 2 | Np | SOD | |
| 49 | 2 | SOD | Np | 2 | 94 | 1 | 8 | 1.1 | | | | 19.25 | 0.06 | | | 2 | Np | SOD | |
| 50 | 2 | SOD | Np | 2 | 56 | 2 | 23 | 17.3 | | | | 19.25 | 0.90 | | | 2 | Np | SOD | |
| 51 | 2 | SOD | Np | 2 | 31 | 3 | 24 | 21.6 | | | | 19.25 | 1.12 | | | 2 | Np | SOD | |
| 52 | 2 | SOD | Np | 2 | 87 | 4 | 17 | 19.3 | | | | 19.25 | 1.00 | | | 2 | Np | SOD | |
| 53 | 2 | SOD | Np | 2 | 20 | 5 | 15 | 3.0 | | | | 19.25 | 0.16 | | | 2 | Np | SOD | |
| 54 | 2 | SOD | Np | 2 | 23 | 6 | 14 | 0.5 | | | | 19.25 | 0.03 | | | 2 | Np | SOD | |
| 55 | 2 | SOD | Np | 2 | 41 | 7 | 5 | 19.2 | | | | 19.25 | 1.00 | | | 2 | Np | SOD | |

# Supplemental Table S1

| | | | | | | | | | | | | |
|---|---|---|---|---|---|---|---|---|---|---|---|---|
| NA | NA | | | | NA | NA | | | | 2 | Pp | SOD |
| 12.37 | 1.88 | | | | 22.60 | 1.03 | | | | 2 | Pp | SOD |
| 12.37 | 1.79 | | | | 22.60 | 0.98 | | | | 2 | Pp | SOD |
| 12.37 | 2.06 | | | | 22.60 | 1.13 | | | | 2 | Pp | SOD |
| 12.37 | 1.93 | | | | 22.60 | 1.06 | | | | 2 | Pp | SOD |
| 12.37 | 2.20 | 1.99 | | | 22.60 | 1.20 | 1.092 | | | 2 | Pp | SOD |
| 12.37 | 2.06 | | | | 22.60 | 1.13 | | | | 2 | Pp | SOD |
| 12.37 | 1.74 | | | | 22.60 | 0.95 | | | | 2 | Pp | SOD |
| 12.37 | 2.23 | | | | 22.60 | 1.22 | | | | 2 | Pp | SOD |
| NA | NA | | | | NA | NA | | | | 2 | Pp | SOD |
| 12.37 | 2.08 | | | | 22.60 | 1.14 | | | | 2 | Pp | SOD |
| 12.37 | 2.16 | | | | 22.60 | 1.18 | | | | 2 | Pp | SOD |
| 12.37 | 1.77 | | | | 22.60 | 0.97 | | | | 2 | Pp | SOD |
| 12.37 | 1.88 | 1.99 | | | 22.60 | 1.03 | 1.088 | | | 2 | Pp | SOD |
| 12.37 | 2.14 | | | | 22.60 | 1.17 | | | | 2 | Pp | SOD |
| NA | NA | | | | NA | NA | | | | 2 | Pp | SOD |
| 12.37 | 2.35 | | | | 22.60 | 1.29 | | | | 2 | Pp | SOD |
| 12.37 | 2.08 | | | | 22.60 | 1.14 | | | | 2 | Pp | SOD |
| 12.37 | 2.22 | | | | 22.60 | 1.22 | | | | 2 | Pp | SOD |
| 12.37 | 2.05 | | | | 22.60 | 1.12 | | | | 2 | Pp | SOD |
| 12.37 | 2.13 | | | | 22.60 | 1.16 | | | | 2 | Pp | SOD |
| 12.37 | 2.02 | 2.14 | | | 22.60 | 1.11 | 1.172 | | | 2 | Pp | SOD |
| 12.37 | 2.09 | | | | 22.60 | 1.15 | | | | 2 | Pp | SOD |
| 12.37 | 2.47 | | | | 22.60 | 1.35 | | | | 2 | Pp | SOD |
| 12.37 | 2.05 | | | | 22.60 | 1.12 | | | | 2 | Pp | SOD |
| 12.37 | 2.34 | | | | 22.60 | 1.28 | | | | 2 | Pp | SOD |
| 12.37 | 1.75 | | | | 22.60 | 0.96 | | | | 2 | Pp | SOD |
| 12.37 | 1.88 | | | | 22.60 | 1.03 | | | | 2 | Pp | SOD |
| 12.37 | 2.08 | | | | 22.60 | 1.14 | | | | 2 | Pp | SOD |
| 12.37 | 2.08 | 2.09 | | | 22.60 | 1.14 | 1.145 | | | 2 | Pp | SOD |
| 12.37 | 2.03 | | | | 22.60 | 1.11 | | | | 2 | Pp | SOD |
| 12.37 | 1.60 | | | | 22.60 | 0.88 | | | | 2 | Pp | SOD |
| 12.37 | 1.66 | | | | 22.60 | 0.91 | | | | 2 | Pp | SOD |
| 12.37 | 1.88 | | | | 22.60 | 1.03 | | | | 2 | Pp | SOD |
| 12.37 | 1.99 | | | | 22.60 | 1.09 | | | | 2 | Pp | SOD |
| 12.37 | 1.79 | | Generation 2 | | 22.60 | 0.98 | | Generation 2 | | 2 | Pp | SOD |
| 12.37 | 2.06 | | Average | StDev | 22.60 | 1.13 | | Average | StDev | 2 | Pp | SOD |
| 12.37 | 1.70 | 1.84 | 2.011 | 0.116 | 22.60 | 0.93 | 1.006 | 1.101 | 0.064 | 2 | Pp | SOD |
| | | | | | | | | | | 2 | Np | SOD |
| | | | | | | | | | | 2 | Np | SOD |
| | | | | | | | | | | 2 | Np | SOD |
| | | | | | | | | | | 2 | Np | SOD |
| | | | | | | | | | | 2 | Np | SOD |
| | | | | | | | | | | 2 | Np | SOD |
| | | | | | | | | | | 2 | Np | SOD |
| | | | | | | | | | | 2 | Np | SOD |
| | | | | | | | | | | 2 | Np | SOD |
| | | | | | | | | | | 2 | Np | SOD |
| | | | | | | | | | | 2 | Np | SOD |
| | | | | | | | | | | 2 | Np | SOD |
| | | | | | | | | | | 2 | Np | SOD |
| | | | | | | | | | | 2 | Np | SOD |
| | | | | | | | | | | 2 | Np | SOD |

# Supplemental Table S1

| | | | | | | | | | | | | | | | | | | | |
|---|---|---|---|---|---|---|---|---|---|---|---|---|---|---|---|---|---|---|---|
| 56 | 2 | SOD | Np | 2 | 72 | 8 | 2 | 9.4 | 11.43 | | | 19.25 | 0.49 | 0.594 | | 2 | Np | SOD | |
| 57 | 2 | SOD | Np | 3 | 28 | 1 | 4 | 22.2 | | | | 19.25 | 1.15 | | | 2 | Np | SOD | |
| 58 | 2 | SOD | Np | 3 | 73 | 2 | 8 | 17.3 | | | | 19.25 | 0.90 | | | 2 | Np | SOD | |
| 59 | 2 | SOD | Np | 3 | 17 | 3 | 1 | 3.0 | | | | 19.25 | 0.16 | | | 2 | Np | SOD | |
| 60 | 2 | SOD | Np | 3 | 82 | 4 | 1 | NA | | | | NA | NA | | | 2 | Np | SOD | |
| 61 | 2 | SOD | Np | 3 | 15 | 5 | 25 | NA | | | | NA | NA | | | 2 | Np | SOD | |
| 62 | 2 | SOD | Np | 3 | 93 | 6 | 5 | 19.2 | | | | 19.25 | 1.00 | | | 2 | Np | SOD | |
| 63 | 2 | SOD | Np | 3 | 8 | 7 | 19 | NA | | | | NA | NA | | | 2 | Np | SOD | |
| 64 | 2 | SOD | Np | 3 | 1 | 8 | 16 | 15.8 | 15.50 | | | 19.25 | 0.82 | 0.805 | | 2 | Np | SOD | |
| 65 | 2 | SOD | Np | 4 | 75 | 1 | 21 | 11.6 | | | | 19.25 | 0.60 | | | 2 | Np | SOD | |
| 66 | 2 | SOD | Np | 4 | 86 | 2 | 15 | 15.2 | | | | 19.25 | 0.79 | | | 2 | Np | SOD | |
| 67 | 2 | SOD | Np | 4 | 37 | 3 | 13 | 18.4 | | | | 19.25 | 0.96 | | | 2 | Np | SOD | |
| 68 | 2 | SOD | Np | 4 | 53 | 4 | 12 | 21.1 | | | | 19.25 | 1.10 | | | 2 | Np | SOD | |
| 69 | 2 | SOD | Np | 4 | 74 | 5 | 13 | 15.8 | | | | 19.25 | 0.82 | | | 2 | Np | SOD | |
| 70 | 2 | SOD | Np | 4 | 2 | 6 | 12 | 3.0 | | | | 19.25 | 0.16 | | | 2 | Np | SOD | |
| 71 | 2 | SOD | Np | 4 | 40 | 7 | 6 | 0.8 | | | | 19.25 | 0.04 | | | 2 | Np | SOD | |
| 72 | 2 | SOD | Np | 4 | 4 | 8 | 14 | 10.5 | 12.05 | | | 19.25 | 0.55 | 0.626 | | 2 | Np | SOD | |
| 73 | 2 | SOD | Np | 5 | 83 | 1 | 13 | 14.7 | | | | 19.25 | 0.76 | | | 2 | Np | SOD | |
| 74 | 2 | SOD | Np | 5 | 64 | 2 | 7 | 16.2 | | | | 19.25 | 0.84 | | | 2 | Np | SOD | |
| 75 | 2 | SOD | Np | 5 | 62 | 3 | 3 | 0.6 | | | | 19.25 | 0.03 | | | 2 | Np | SOD | |
| 76 | 2 | SOD | Np | 5 | 33 | 4 | 14 | NA | | | | NA | NA | | | 2 | Np | SOD | |
| 77 | 2 | SOD | Np | 5 | 10 | 5 | 20 | 16.3 | | | | 19.25 | 0.85 | | | 2 | Np | SOD | |
| 78 | 2 | SOD | Np | 5 | 71 | 6 | 1 | 3.7 | | | | 19.25 | 0.19 | | | 2 | Np | SOD | |
| 79 | 2 | SOD | Np | 5 | 45 | 7 | 15 | 17.6 | | | | 19.25 | 0.91 | | | 2 | Np | SOD | |
| 80 | 2 | SOD | Np | 5 | 91 | 8 | 5 | 12.8 | 11.70 | 12.59 | | 19.25 | 0.67 | 0.608 | 0.654 | 2 | Np | SOD | 12.37 |
| 91 | 2 | SOD | Null | NA | 77 | 1 | 7 | 27.8 | | | | 19.25 | 1.44 | | | 2 | Null | SOD | |
| 92 | 2 | SOD | Null | NA | 51 | 2 | 16 | 23.6 | | | | 19.25 | 1.23 | | | 2 | Null | SOD | |
| 93 | 2 | SOD | Null | NA | 68 | 3 | 5 | 23.0 | | | | 19.25 | 1.19 | | | 2 | Null | SOD | |
| 94 | 2 | SOD | Null | NA | 16 | 4 | 25 | 22.8 | | | | 19.25 | 1.18 | | | 2 | Null | SOD | |
| 95 | 2 | SOD | Null | NA | 84 | 5 | 1 | 17.7 | | | | 19.25 | 0.92 | | | 2 | Null | SOD | |
| 96 | 2 | SOD | Null | NA | 65 | 6 | 8 | 23.5 | | | | 19.25 | 1.22 | | | 2 | Null | SOD | |
| 97 | 2 | SOD | Null | NA | 50 | 7 | 14 | 22.7 | | | | 19.25 | 1.18 | | | 2 | Null | SOD | |
| 98 | 2 | SOD | Null | NA | 7 | 8 | 8 | 23.4 | | | | 19.25 | 1.22 | | | 2 | Null | SOD | |
| 99 | 2 | SOD | Null | NA | 32 | 3 | 22 | 22.0 | | | Grand Average | 19.25 | 1.14 | | | 2 | Null | SOD | |
| 100 | 2 | SOD | Null | NA | 70 | 6 | 20 | 19.5 | 22.60 | | 19.25 | 19.25 | 1.01 | 1.174 | | 2 | Null | SOD | 22.60 |
| 101 | 2 | ALU | Pp | 1 | 143 | 1 | 19 | 28.2 | | | | 25.64 | 1.10 | | | 2 | Pp | ALU | |
| 102 | 2 | ALU | Pp | 1 | 117 | 2 | 12 | NA | | | | NA | NA | | | 2 | Pp | ALU | |
| 103 | 2 | ALU | Pp | 1 | 125 | 3 | 16 | 21.4 | | | | 25.64 | 0.83 | | | 2 | Pp | ALU | |
| 104 | 2 | ALU | Pp | 1 | 103 | 4 | 13 | 22.6 | | | | 25.64 | 0.88 | | | 2 | Pp | ALU | |
| 105 | 2 | ALU | Pp | 1 | 174 | 5 | 8 | 26.7 | | | | 25.64 | 1.04 | | | 2 | Pp | ALU | |
| 106 | 2 | ALU | Pp | 1 | 190 | 6 | 10 | 25.5 | | | | 25.64 | 0.99 | | | 2 | Pp | ALU | |
| 107 | 2 | ALU | Pp | 1 | 196 | 7 | 18 | 33.8 | | | | 25.64 | 1.32 | | | 2 | Pp | ALU | |
| 108 | 2 | ALU | Pp | 1 | 105 | 8 | 25 | 28.8 | 26.71 | | | 25.64 | 1.12 | 1.042 | | 2 | Pp | ALU | |
| 109 | 2 | ALU | Pp | 2 | 186 | 1 | 23 | 31.3 | | | | 25.64 | 1.22 | | | 2 | Pp | ALU | |
| 110 | 2 | ALU | Pp | 2 | 162 | 2 | 5 | 15.2 | | | | 25.64 | 0.59 | | | 2 | Pp | ALU | |
| 111 | 2 | ALU | Pp | 2 | 185 | 3 | 20 | 33.1 | | | | 25.64 | 1.29 | | | 2 | Pp | ALU | |
| 112 | 2 | ALU | Pp | 2 | 126 | 4 | 9 | 28.6 | | | | 25.64 | 1.12 | | | 2 | Pp | ALU | |
| 113 | 2 | ALU | Pp | 2 | 179 | 5 | 10 | 28.6 | | | | 25.64 | 1.12 | | | 2 | Pp | ALU | |
| 114 | 2 | ALU | Pp | 2 | 176 | 6 | 6 | 32.1 | | | | 25.64 | 1.25 | | | 2 | Pp | ALU | |
| 115 | 2 | ALU | Pp | 2 | 107 | 7 | 1 | 28.9 | | | | 25.64 | 1.13 | | | 2 | Pp | ALU | |
| 116 | 2 | ALU | Pp | 2 | 131 | 8 | 10 | 29.9 | 28.46 | | | 25.64 | 1.17 | 1.110 | | 2 | Pp | ALU | |
| 117 | 2 | ALU | Pp | 3 | 121 | 1 | 25 | 29.6 | | | | 25.64 | 1.15 | | | 2 | Pp | ALU | |
| 118 | 2 | ALU | Pp | 3 | 130 | 2 | 20 | 20.7 | | | | 25.64 | 0.81 | | | 2 | Pp | ALU | |

# Supplemental Table S1

| | | | | | | | | | | | | |
|---|---|---|---|---|---|---|---|---|---|---|---|---|
| | | | | | | | | | | 2 | Np | SOD |
| | | | | | | | | | | 2 | Np | SOD |
| | | | | | | | | | | 2 | Np | SOD |
| | | | | | | | | | | 2 | Np | SOD |
| | | | | | | | | | | 2 | Np | SOD |
| | | | | | | | | | | 2 | Np | SOD |
| | | | | | | | | | | 2 | Np | SOD |
| | | | | | | | | | | 2 | Np | SOD |
| | | | | | | | | | | 2 | Np | SOD |
| | | | | | | | | | | 2 | Np | SOD |
| | | | | | | | | | | 2 | Np | SOD |
| | | | | | | | | | | 2 | Np | SOD |
| | | | | | | | | | | 2 | Np | SOD |
| | | | | | | | | | | 2 | Np | SOD |
| | | | | | | | | | | 2 | Np | SOD |
| | | | | | | | | | | 2 | Np | SOD |
| | | | | | | | | | | 2 | Np | SOD |
| | | | | | | | | | | 2 | Np | SOD |
| | | | | | | | | | | 2 | Np | SOD |
| | | | | | | | | | | 2 | Np | SOD |
| | | | | | | | | | | 2 | Np | SOD |
| | | | | | | | | | | 2 | Np | SOD |
| | | | | | | | | | | 2 | Np | SOD |
| | | | | | | | | | | 2 | Np | SOD |
| | | | | | | | | | | 2 | Np | SOD |
| | | | | | | | | | | 2 | Null | SOD |
| | | | | | | | | | | 2 | Null | SOD |
| | | | | | | | | | | 2 | Null | SOD |
| | | | | | | | | | | 2 | Null | SOD |
| | | | | | | | | | | 2 | Null | SOD |
| | | | | | | | | | | 2 | Null | SOD |
| | | | | | | | | | | 2 | Null | SOD |
| | | | | | | | | | | 2 | Null | SOD |
| | | | | | | | | | | 2 | Null | SOD |
| | | | | | | | | | | 2 | Null | SOD |
| 22.13 | 1.27 | | | | 25.58 | 1.10 | | | | 2 | Pp | ALU |
| NA | NA | | | | NA | NA | | | | 2 | Pp | ALU |
| 22.13 | 0.97 | | | | 25.58 | 0.84 | | | | 2 | Pp | ALU |
| 22.13 | 1.02 | | | | 25.58 | 0.88 | | | | 2 | Pp | ALU |
| 22.13 | 1.21 | | | | 25.58 | 1.04 | | | | 2 | Pp | ALU |
| 22.13 | 1.15 | | | | 25.58 | 1.00 | | | | 2 | Pp | ALU |
| 22.13 | 1.53 | | | | 25.58 | 1.32 | | | | 2 | Pp | ALU |
| 22.13 | 1.30 | 1.21 | | | 25.58 | 1.13 | 1.044 | | | 2 | Pp | ALU |
| 22.13 | 1.41 | | | | 25.58 | 1.22 | | | | 2 | Pp | ALU |
| 22.13 | 0.69 | | | | 25.58 | 0.59 | | | | 2 | Pp | ALU |
| 22.13 | 1.50 | | | | 25.58 | 1.29 | | | | 2 | Pp | ALU |
| 22.13 | 1.29 | | | | 25.58 | 1.12 | | | | 2 | Pp | ALU |
| 22.13 | 1.29 | | | | 25.58 | 1.12 | | | | 2 | Pp | ALU |
| 22.13 | 1.45 | | | | 25.58 | 1.25 | | | | 2 | Pp | ALU |
| 22.13 | 1.31 | | | | 25.58 | 1.13 | | | | 2 | Pp | ALU |
| 22.13 | 1.35 | 1.29 | | | 25.58 | 1.17 | 1.113 | | | 2 | Pp | ALU |
| 22.13 | 1.34 | | | | 25.58 | 1.16 | | | | 2 | Pp | ALU |
| 22.13 | 0.94 | | | | 25.58 | 0.81 | | | | 2 | Pp | ALU |

# Supplemental Table S1

| | | | | | | | | | | | | | | | | | | | |
|---|---|---|---|---|---|---|---|---|---|---|---|---|---|---|---|---|---|---|---|
| 119 | 2 | ALU | Pp | 3 | 110 | 3 | 14 | 30.3 | | | | 25.64 | 1.18 | | | 2 | Pp | ALU | |
| 120 | 2 | ALU | Pp | 3 | 128 | 4 | 20 | 32.6 | | | | 25.64 | 1.27 | | | 2 | Pp | ALU | |
| 121 | 2 | ALU | Pp | 3 | 173 | 5 | 17 | 32.4 | | | | 25.64 | 1.26 | | | 2 | Pp | ALU | |
| 122 | 2 | ALU | Pp | 3 | 153 | 6 | 19 | 31.0 | | | | 25.64 | 1.21 | | | 2 | Pp | ALU | |
| 123 | 2 | ALU | Pp | 3 | 194 | 7 | 9 | 28.9 | | | | 25.64 | 1.13 | | | 2 | Pp | ALU | |
| 124 | 2 | ALU | Pp | 3 | 184 | 8 | 21 | 24.5 | 28.75 | | | 25.64 | 0.96 | 1.121 | | 2 | Pp | ALU | |
| 125 | 2 | ALU | Pp | 4 | 109 | 1 | 12 | 30.1 | | | | 25.64 | 1.17 | | | 2 | Pp | ALU | |
| 126 | 2 | ALU | Pp | 4 | 104 | 2 | 9 | 26.3 | | | | 25.64 | 1.03 | | | 2 | Pp | ALU | |
| 127 | 2 | ALU | Pp | 4 | 139 | 3 | 4 | 34.2 | | | | 25.64 | 1.33 | | | 2 | Pp | ALU | |
| 128 | 2 | ALU | Pp | 4 | 175 | 4 | 6 | 36.3 | | | | 25.64 | 1.42 | | | 2 | Pp | ALU | |
| 129 | 2 | ALU | Pp | 4 | 116 | 5 | 24 | 38.6 | | | | 25.64 | 1.51 | | | 2 | Pp | ALU | |
| 130 | 2 | ALU | Pp | 4 | 182 | 6 | 22 | 41.1 | | | | 25.64 | 1.60 | | | 2 | Pp | ALU | |
| 131 | 2 | ALU | Pp | 4 | 192 | 7 | 24 | 33.9 | | | | 25.64 | 1.32 | | | 2 | Pp | ALU | |
| 132 | 2 | ALU | Pp | 4 | 198 | 8 | 17 | 25.2 | 33.21 | | | 25.64 | 0.98 | 1.295 | | 2 | Pp | ALU | |
| 133 | 2 | ALU | Pp | 5 | 137 | 1 | 16 | 31.5 | | | | 25.64 | 1.23 | | | 2 | Pp | ALU | |
| 134 | 2 | ALU | Pp | 5 | 200 | 2 | 4 | 28.1 | | | | 25.64 | 1.10 | | | 2 | Pp | ALU | |
| 135 | 2 | ALU | Pp | 5 | 132 | 3 | 6 | 27.4 | | | | 25.64 | 1.07 | | | 2 | Pp | ALU | |
| 136 | 2 | ALU | Pp | 5 | 141 | 4 | 19 | 30.6 | | | | 25.64 | 1.19 | | | 2 | Pp | ALU | |
| 137 | 2 | ALU | Pp | 5 | 177 | 5 | 6 | 30.7 | | | | 25.64 | 1.20 | | | 2 | Pp | ALU | |
| 138 | 2 | ALU | Pp | 5 | 102 | 6 | 11 | 26.8 | | | | 25.64 | 1.05 | | | 2 | Pp | ALU | |
| 139 | 2 | ALU | Pp | 5 | 154 | 7 | 25 | 24.7 | | | | 25.64 | 0.96 | | | 2 | Pp | ALU | |
| 140 | 2 | ALU | Pp | 5 | 161 | 8 | 1 | 27.4 | 28.40 | 29.11 | | 25.64 | 1.07 | 1.107 | 1.135 | 2 | Pp | ALU | 29.17 |
| 141 | 2 | ALU | Np | 1 | 188 | 1 | 2 | 26.7 | | | | 25.64 | 1.04 | | | 2 | Np | ALU | |
| 142 | 2 | ALU | Np | 1 | 101 | 2 | 21 | 11.0 | | | | 25.64 | 0.43 | | | 2 | Np | ALU | |
| 143 | 2 | ALU | Np | 1 | 181 | 3 | 9 | 21.0 | | | | 25.64 | 0.82 | | | 2 | Np | ALU | |
| 144 | 2 | ALU | Np | 1 | 140 | 4 | 18 | 25.9 | | | | 25.64 | 1.01 | | | 2 | Np | ALU | |
| 145 | 2 | ALU | Np | 1 | 197 | 5 | 12 | 20.6 | | | | 25.64 | 0.80 | | | 2 | Np | ALU | |
| 146 | 2 | ALU | Np | 1 | 155 | 6 | 18 | 24.4 | | | | 25.64 | 0.95 | | | 2 | Np | ALU | |
| 147 | 2 | ALU | Np | 1 | 157 | 7 | 7 | 23.7 | | | | 25.64 | 0.92 | | | 2 | Np | ALU | |
| 148 | 2 | ALU | Np | 1 | 166 | 8 | 13 | 32.4 | 23.21 | | | 25.64 | 1.26 | 0.905 | | 2 | Np | ALU | |
| 149 | 2 | ALU | Np | 2 | 170 | 1 | 11 | 26.1 | | | | 25.64 | 1.02 | | | 2 | Np | ALU | |
| 150 | 2 | ALU | Np | 2 | 147 | 2 | 1 | 19.6 | | | | 25.64 | 0.76 | | | 2 | Np | ALU | |
| 151 | 2 | ALU | Np | 2 | 150 | 3 | 11 | 20.6 | | | | 25.64 | 0.80 | | | 2 | Np | ALU | |
| 152 | 2 | ALU | Np | 2 | 165 | 4 | 21 | 22.0 | | | | 25.64 | 0.86 | | | 2 | Np | ALU | |
| 153 | 2 | ALU | Np | 2 | 171 | 5 | 19 | 28.2 | | | | 25.64 | 1.10 | | | 2 | Np | ALU | |
| 154 | 2 | ALU | Np | 2 | 191 | 6 | 25 | 22.9 | | | | 25.64 | 0.89 | | | 2 | Np | ALU | |
| 155 | 2 | ALU | Np | 2 | 112 | 7 | 13 | 25.7 | | | | 25.64 | 1.00 | | | 2 | Np | ALU | |
| 156 | 2 | ALU | Np | 2 | 114 | 8 | 4 | 22.2 | 23.41 | | | 25.64 | 0.87 | 0.913 | | 2 | Np | ALU | |
| 157 | 2 | ALU | Np | 3 | 187 | 1 | 9 | 27.6 | | | | 25.64 | 1.08 | | | 2 | Np | ALU | |
| 158 | 2 | ALU | Np | 3 | 118 | 2 | 13 | 27.0 | | | | 25.64 | 1.05 | | | 2 | Np | ALU | |
| 159 | 2 | ALU | Np | 3 | 146 | 3 | 23 | 25.3 | | | | 25.64 | 0.99 | | | 2 | Np | ALU | |
| 160 | 2 | ALU | Np | 3 | 136 | 4 | 7 | 26.3 | | | | 25.64 | 1.03 | | | 2 | Np | ALU | |
| 161 | 2 | ALU | Np | 3 | 124 | 5 | 4 | 24.0 | | | | 25.64 | 0.94 | | | 2 | Np | ALU | |
| 162 | 2 | ALU | Np | 3 | 163 | 6 | 23 | 26.4 | | | | 25.64 | 1.03 | | | 2 | Np | ALU | |
| 163 | 2 | ALU | Np | 3 | 142 | 7 | 10 | 25.8 | | | | 25.64 | 1.01 | | | 2 | Np | ALU | |
| 164 | 2 | ALU | Np | 3 | 180 | 8 | 6 | 2.1 | 23.06 | | | 25.64 | 0.08 | 0.899 | | 2 | Np | ALU | |
| 165 | 2 | ALU | Np | 4 | 183 | 1 | 22 | 33.0 | | | | 25.64 | 1.29 | | | 2 | Np | ALU | |
| 166 | 2 | ALU | Np | 4 | 134 | 2 | 17 | 15.7 | | | | 25.64 | 0.61 | | | 2 | Np | ALU | |
| 167 | 2 | ALU | Np | 4 | 156 | 3 | 12 | 27.0 | | | | 25.64 | 1.05 | | | 2 | Np | ALU | |
| 168 | 2 | ALU | Np | 4 | 122 | 4 | 2 | 29.5 | | | | 25.64 | 1.15 | | | 2 | Np | ALU | |
| 169 | 2 | ALU | Np | 4 | 199 | 5 | 22 | 23.0 | | | | 25.64 | 0.90 | | | 2 | Np | ALU | |
| 170 | 2 | ALU | Np | 4 | 111 | 6 | 13 | 0.6 | | | | 25.64 | 0.02 | | | 2 | Np | ALU | |
| 171 | 2 | ALU | Np | 4 | 113 | 7 | 3 | 11.1 | | | | 25.64 | 0.43 | | | 2 | Np | ALU | |

# Supplemental Table S1

| | | | | | | | | | | | | |
|---|---|---|---|---|---|---|---|---|---|---|---|---|
| 22.13 | 1.37 | | | | 25.58 | 1.18 | | | | 2 | Pp | ALU |
| 22.13 | 1.47 | | | | 25.58 | 1.27 | | | | 2 | Pp | ALU |
| 22.13 | 1.46 | | | | 25.58 | 1.27 | | | | 2 | Pp | ALU |
| 22.13 | 1.40 | | | | 25.58 | 1.21 | | | | 2 | Pp | ALU |
| 22.13 | 1.31 | | | | 25.58 | 1.13 | | | | 2 | Pp | ALU |
| 22.13 | 1.11 | 1.30 | | | 25.58 | 0.96 | 1.124 | | | 2 | Pp | ALU |
| 22.13 | 1.36 | | | | 25.58 | 1.18 | | | | 2 | Pp | ALU |
| 22.13 | 1.19 | | | | 25.58 | 1.03 | | | | 2 | Pp | ALU |
| 22.13 | 1.55 | | | | 25.58 | 1.34 | | | | 2 | Pp | ALU |
| 22.13 | 1.64 | | | | 25.58 | 1.42 | | | | 2 | Pp | ALU |
| 22.13 | 1.74 | | | | 25.58 | 1.51 | | | | 2 | Pp | ALU |
| 22.13 | 1.86 | | | | 25.58 | 1.61 | | | | 2 | Pp | ALU |
| 22.13 | 1.53 | | | | 25.58 | 1.33 | | | | 2 | Pp | ALU |
| 22.13 | 1.14 | 1.50 | | | 25.58 | 0.99 | 1.298 | | | 2 | Pp | ALU |
| 22.13 | 1.42 | | | | 25.58 | 1.23 | | | | 2 | Pp | ALU |
| 22.13 | 1.27 | | | | 25.58 | 1.10 | | | | 2 | Pp | ALU |
| 22.13 | 1.24 | | | | 25.58 | 1.07 | | | | 2 | Pp | ALU |
| 22.13 | 1.38 | | | | 25.58 | 1.20 | | | | 2 | Pp | ALU |
| 22.13 | 1.39 | | | | 25.58 | 1.20 | | | | 2 | Pp | ALU |
| 22.13 | 1.21 | | Generation 2 | | 25.58 | 1.05 | | Generation 2 | | 2 | Pp | ALU |
| 22.13 | 1.12 | | Average | StDev | 25.58 | 0.97 | | Average | StDev | 2 | Pp | ALU |
| 22.13 | 1.24 | 1.28 | 1.315 | 0.110 | 25.58 | 1.07 | 1.110 | 1.138 | 0.095 | 2 | Pp | ALU |
| | | | | | | | | | | 2 | Np | ALU |
| | | | | | | | | | | 2 | Np | ALU |
| | | | | | | | | | | 2 | Np | ALU |
| | | | | | | | | | | 2 | Np | ALU |
| | | | | | | | | | | 2 | Np | ALU |
| | | | | | | | | | | 2 | Np | ALU |
| | | | | | | | | | | 2 | Np | ALU |
| | | | | | | | | | | 2 | Np | ALU |
| | | | | | | | | | | 2 | Np | ALU |
| | | | | | | | | | | 2 | Np | ALU |
| | | | | | | | | | | 2 | Np | ALU |
| | | | | | | | | | | 2 | Np | ALU |
| | | | | | | | | | | 2 | Np | ALU |
| | | | | | | | | | | 2 | Np | ALU |
| | | | | | | | | | | 2 | Np | ALU |
| | | | | | | | | | | 2 | Np | ALU |
| | | | | | | | | | | 2 | Np | ALU |
| | | | | | | | | | | 2 | Np | ALU |
| | | | | | | | | | | 2 | Np | ALU |
| | | | | | | | | | | 2 | Np | ALU |
| | | | | | | | | | | 2 | Np | ALU |
| | | | | | | | | | | 2 | Np | ALU |
| | | | | | | | | | | 2 | Np | ALU |
| | | | | | | | | | | 2 | Np | ALU |
| | | | | | | | | | | 2 | Np | ALU |
| | | | | | | | | | | 2 | Np | ALU |
| | | | | | | | | | | 2 | Np | ALU |
| | | | | | | | | | | 2 | Np | ALU |
| | | | | | | | | | | 2 | Np | ALU |
| | | | | | | | | | | 2 | Np | ALU |
| | | | | | | | | | | 2 | Np | ALU |

# Supplemental Table S1

| | | | | | | | | | | | | | | | | | | | |
|---|---|---|---|---|---|---|---|---|---|---|---|---|---|---|---|---|---|---|---|
| 172 | 2 | ALU | Np | 4 | 106 | 8 | 23 | 6.6 | 18.31 | | | 25.64 | 0.26 | 0.714 | | 2 | Np | ALU | |
| 173 | 2 | ALU | Np | 5 | 129 | 1 | 6 | 26.2 | | | | 25.64 | 1.02 | | | 2 | Np | ALU | |
| 174 | 2 | ALU | Np | 5 | 148 | 2 | 24 | 25.8 | | | | 25.64 | 1.01 | | | 2 | Np | ALU | |
| 175 | 2 | ALU | Np | 5 | 149 | 3 | 10 | NA | | | | NA | NA | | | 2 | Np | ALU | |
| 176 | 2 | ALU | Np | 5 | 168 | 4 | 22 | 32.7 | | | | 25.64 | 1.28 | | | 2 | Np | ALU | |
| 177 | 2 | ALU | Np | 5 | 127 | 5 | 9 | 12.4 | | | | 25.64 | 0.48 | | | 2 | Np | ALU | |
| 178 | 2 | ALU | Np | 5 | 123 | 6 | 4 | 24.9 | | | | 25.64 | 0.97 | | | 2 | Np | ALU | |
| 179 | 2 | ALU | Np | 5 | 160 | 7 | 17 | 9.5 | | | | 25.64 | 0.37 | | | 2 | Np | ALU | |
| 180 | 2 | ALU | Np | 5 | 120 | 8 | 18 | 27.7 | 22.74 | 22.15 | | 25.64 | 1.08 | 0.887 | 0.864 | 2 | Np | ALU | 22.13 |
| 191 | 2 | ALU | Null | NA | 145 | 1 | 3 | 26.8 | | | | 25.64 | 1.05 | | | 2 | Null | ALU | |
| 192 | 2 | ALU | Null | NA | 164 | 2 | 19 | NA | | | | NA | NA | | | 2 | Null | ALU | |
| 193 | 2 | ALU | Null | NA | 151 | 3 | 25 | 23.6 | | | | 25.64 | 0.92 | | | 2 | Null | ALU | |
| 194 | 2 | ALU | Null | NA | 108 | 4 | 5 | 23.9 | | | | 25.64 | 0.93 | | | 2 | Null | ALU | |
| 195 | 2 | ALU | Null | NA | 158 | 5 | 11 | 23.4 | | | | 25.64 | 0.91 | | | 2 | Null | ALU | |
| 196 | 2 | ALU | Null | NA | 152 | 6 | 9 | 25.6 | | | | 25.64 | 1.00 | | | 2 | Null | ALU | |
| 197 | 2 | ALU | Null | NA | 178 | 7 | 22 | 23.2 | | | | 25.64 | 0.90 | | | 2 | Null | ALU | |
| 198 | 2 | ALU | Null | NA | 133 | 8 | 24 | 25.6 | | | | 25.64 | 1.00 | | | 2 | Null | ALU | |
| 199 | 2 | ALU | Null | NA | 172 | 4 | 15 | 29.0 | | | Grand Average | 25.64 | 1.13 | | | 2 | Null | ALU | |
| 200 | 2 | ALU | Null | NA | 195 | 7 | 8 | 29.1 | 25.58 | | 25.64 | 25.64 | 1.13 | 0.997 | | 2 | Null | ALU | 25.58 |
| 1 | 3 | SOD | Pp | 1 | 62 | 1 | 2 | NA | | | | NA | NA | | | 3 | Pp | SOD | |
| 2 | 3 | SOD | Pp | 1 | 43 | 2 | 5 | 33.4 | | | | 24.66 | 1.35 | | | 3 | Pp | SOD | |
| 3 | 3 | SOD | Pp | 1 | 27 | 3 | 9 | 33.8 | | | | 24.66 | 1.37 | | | 3 | Pp | SOD | |
| 4 | 3 | SOD | Pp | 1 | 44 | 4 | 13 | 41.3 | | | | 24.66 | 1.67 | | | 3 | Pp | SOD | |
| 5 | 3 | SOD | Pp | 1 | 75 | 5 | 10 | 31.7 | | | | 24.66 | 1.29 | | | 3 | Pp | SOD | |
| 6 | 3 | SOD | Pp | 1 | 19 | 6 | 20 | 31.2 | | | | 24.66 | 1.27 | | | 3 | Pp | SOD | |
| 7 | 3 | SOD | Pp | 1 | 50 | 7 | 16 | NA | | | | NA | NA | | | 3 | Pp | SOD | |
| 8 | 3 | SOD | Pp | 1 | 96 | 8 | 2 | 31.0 | 33.73 | | | 24.66 | 1.26 | 1.368 | | 3 | Pp | SOD | |
| 9 | 3 | SOD | Pp | 2 | 76 | 1 | 3 | 37.6 | | | | 24.66 | 1.52 | | | 3 | Pp | SOD | |
| 10 | 3 | SOD | Pp | 2 | 54 | 2 | 4 | 35.2 | | | | 24.66 | 1.43 | | | 3 | Pp | SOD | |
| 11 | 3 | SOD | Pp | 2 | 81 | 3 | 5 | 40.2 | | | | 24.66 | 1.63 | | | 3 | Pp | SOD | |
| 12 | 3 | SOD | Pp | 2 | 36 | 4 | 25 | 37.3 | | | | 24.66 | 1.51 | | | 3 | Pp | SOD | |
| 13 | 3 | SOD | Pp | 2 | 87 | 5 | 21 | 32.7 | | | | 24.66 | 1.33 | | | 3 | Pp | SOD | |
| 14 | 3 | SOD | Pp | 2 | 39 | 6 | 16 | 37.3 | | | | 24.66 | 1.51 | | | 3 | Pp | SOD | |
| 15 | 3 | SOD | Pp | 2 | 24 | 7 | 18 | 36.7 | | | | 24.66 | 1.49 | | | 3 | Pp | SOD | |
| 16 | 3 | SOD | Pp | 2 | 51 | 8 | 10 | 31.3 | 36.04 | | | 24.66 | 1.27 | 1.461 | | 3 | Pp | SOD | |
| 17 | 3 | SOD | Pp | 3 | 64 | 1 | 14 | 37.8 | | | | 24.66 | 1.53 | | | 3 | Pp | SOD | |
| 18 | 3 | SOD | Pp | 3 | 94 | 2 | 21 | 34.0 | | | | 24.66 | 1.38 | | | 3 | Pp | SOD | |
| 19 | 3 | SOD | Pp | 3 | 90 | 3 | 24 | 31.5 | | | | 24.66 | 1.28 | | | 3 | Pp | SOD | |
| 20 | 3 | SOD | Pp | 3 | 58 | 4 | 2 | 30.3 | | | | 24.66 | 1.23 | | | 3 | Pp | SOD | |
| 21 | 3 | SOD | Pp | 3 | 42 | 5 | 6 | 26.5 | | | | 24.66 | 1.07 | | | 3 | Pp | SOD | |
| 22 | 3 | SOD | Pp | 3 | 88 | 6 | 23 | 32.9 | | | | 24.66 | 1.33 | | | 3 | Pp | SOD | |
| 23 | 3 | SOD | Pp | 3 | 66 | 7 | 2 | 32.8 | | | | 24.66 | 1.33 | | | 3 | Pp | SOD | |
| 24 | 3 | SOD | Pp | 3 | 17 | 8 | 20 | 27.0 | 31.60 | | | 24.66 | 1.09 | 1.282 | | 3 | Pp | SOD | |
| 25 | 3 | SOD | Pp | 4 | 16 | 1 | 24 | 33.3 | | | | 24.66 | 1.35 | | | 3 | Pp | SOD | |
| 26 | 3 | SOD | Pp | 4 | 69 | 2 | 10 | 35.8 | | | | 24.66 | 1.45 | | | 3 | Pp | SOD | |
| 27 | 3 | SOD | Pp | 4 | 57 | 3 | 21 | 32.1 | | | | 24.66 | 1.30 | | | 3 | Pp | SOD | |
| 28 | 3 | SOD | Pp | 4 | 91 | 4 | 16 | 32.8 | | | | 24.66 | 1.33 | | | 3 | Pp | SOD | |
| 29 | 3 | SOD | Pp | 4 | 72 | 5 | 3 | 31.4 | | | | 24.66 | 1.27 | | | 3 | Pp | SOD | |
| 30 | 3 | SOD | Pp | 4 | 30 | 6 | 10 | 31.2 | | | | 24.66 | 1.27 | | | 3 | Pp | SOD | |
| 31 | 3 | SOD | Pp | 4 | 55 | 7 | 22 | 37.6 | | | | 24.66 | 1.52 | | | 3 | Pp | SOD | |
| 32 | 3 | SOD | Pp | 4 | 53 | 8 | 1 | 35.5 | 33.71 | | | 24.66 | 1.44 | 1.367 | | 3 | Pp | SOD | |
| 33 | 3 | SOD | Pp | 5 | 82 | 1 | 13 | NA | | | | NA | NA | | | 3 | Pp | SOD | |
| 34 | 3 | SOD | Pp | 5 | 59 | 2 | 18 | 42.6 | | | | 24.66 | 1.73 | | | 3 | Pp | SOD | |

# Supplemental Table S1

| | | | | | | | | | | | | |
|---|---|---|---|---|---|---|---|---|---|---|---|---|
| | | | | | | | | | | 2 | Np | ALU |
| | | | | | | | | | | 2 | Np | ALU |
| | | | | | | | | | | 2 | Np | ALU |
| | | | | | | | | | | 2 | Np | ALU |
| | | | | | | | | | | 2 | Np | ALU |
| | | | | | | | | | | 2 | Np | ALU |
| | | | | | | | | | | 2 | Np | ALU |
| | | | | | | | | | | 2 | Np | ALU |
| | | | | | | | | | | 2 | Np | ALU |
| | | | | | | | | | | 2 | Null | ALU |
| | | | | | | | | | | 2 | Null | ALU |
| | | | | | | | | | | 2 | Null | ALU |
| | | | | | | | | | | 2 | Null | ALU |
| | | | | | | | | | | 2 | Null | ALU |
| | | | | | | | | | | 2 | Null | ALU |
| | | | | | | | | | | 2 | Null | ALU |
| | | | | | | | | | | 2 | Null | ALU |
| | | | | | | | | | | 2 | Null | ALU |
| | | | | | | | | | | 2 | Null | ALU |
| NA | NA | | | | NA | NA | | | | 3 | Pp | SOD |
| 14.15 | 2.36 | | | | 31.92 | 1.05 | | | | 3 | Pp | SOD |
| 14.15 | 2.39 | | | | 31.92 | 1.06 | | | | 3 | Pp | SOD |
| 14.15 | 2.92 | | | | 31.92 | 1.29 | | | | 3 | Pp | SOD |
| 14.15 | 2.24 | | | | 31.92 | 0.99 | | | | 3 | Pp | SOD |
| 14.15 | 2.21 | | | | 31.92 | 0.98 | | | | 3 | Pp | SOD |
| NA | NA | | | | NA | NA | | | | 3 | Pp | SOD |
| 14.15 | 2.19 | 2.38 | | | 31.92 | 0.97 | 1.057 | | | 3 | Pp | SOD |
| 14.15 | 2.66 | | | | 31.92 | 1.18 | | | | 3 | Pp | SOD |
| 14.15 | 2.49 | | | | 31.92 | 1.10 | | | | 3 | Pp | SOD |
| 14.15 | 2.84 | | | | 31.92 | 1.26 | | | | 3 | Pp | SOD |
| 14.15 | 2.64 | | | | 31.92 | 1.17 | | | | 3 | Pp | SOD |
| 14.15 | 2.31 | | | | 31.92 | 1.02 | | | | 3 | Pp | SOD |
| 14.15 | 2.64 | | | | 31.92 | 1.17 | | | | 3 | Pp | SOD |
| 14.15 | 2.59 | | | | 31.92 | 1.15 | | | | 3 | Pp | SOD |
| 14.15 | 2.21 | 2.55 | | | 31.92 | 0.98 | 1.129 | | | 3 | Pp | SOD |
| 14.15 | 2.67 | | | | 31.92 | 1.18 | | | | 3 | Pp | SOD |
| 14.15 | 2.40 | | | | 31.92 | 1.07 | | | | 3 | Pp | SOD |
| 14.15 | 2.23 | | | | 31.92 | 0.99 | | | | 3 | Pp | SOD |
| 14.15 | 2.14 | | | | 31.92 | 0.95 | | | | 3 | Pp | SOD |
| 14.15 | 1.87 | | | | 31.92 | 0.83 | | | | 3 | Pp | SOD |
| 14.15 | 2.33 | | | | 31.92 | 1.03 | | | | 3 | Pp | SOD |
| 14.15 | 2.32 | | | | 31.92 | 1.03 | | | | 3 | Pp | SOD |
| 14.15 | 1.91 | 2.23 | | | 31.92 | 0.85 | 0.990 | | | 3 | Pp | SOD |
| 14.15 | 2.35 | | | | 31.92 | 1.04 | | | | 3 | Pp | SOD |
| 14.15 | 2.53 | | | | 31.92 | 1.12 | | | | 3 | Pp | SOD |
| 14.15 | 2.27 | | | | 31.92 | 1.01 | | | | 3 | Pp | SOD |
| 14.15 | 2.32 | | | | 31.92 | 1.03 | | | | 3 | Pp | SOD |
| 14.15 | 2.22 | | | | 31.92 | 0.98 | | | | 3 | Pp | SOD |
| 14.15 | 2.21 | | | | 31.92 | 0.98 | | | | 3 | Pp | SOD |
| 14.15 | 2.66 | | | | 31.92 | 1.18 | | | | 3 | Pp | SOD |
| 14.15 | 2.51 | 2.38 | | | 31.92 | 1.11 | 1.056 | | | 3 | Pp | SOD |
| NA | NA | | | | NA | NA | | | | 3 | Pp | SOD |
| 14.15 | 3.01 | | | | 31.92 | 1.33 | | | | 3 | Pp | SOD |

## Supplemental Table S1

| | | | | | | | | | | | | | | | | | | | |
|---|---|---|---|---|---|---|---|---|---|---|---|---|---|---|---|---|---|---|---|
| 35 | 3 | SOD | Pp | 5 | 14 | 3 | 13 | 31.5 | | | | 24.66 | 1.28 | | | 3 | Pp | SOD | |
| 36 | 3 | SOD | Pp | 5 | 34 | 4 | 23 | 35.0 | | | | 24.66 | 1.42 | | | 3 | Pp | SOD | |
| 37 | 3 | SOD | Pp | 5 | 79 | 5 | 18 | 25.7 | | | | 24.66 | 1.04 | | | 3 | Pp | SOD | |
| 38 | 3 | SOD | Pp | 5 | 40 | 6 | 1 | 30.0 | | | | 24.66 | 1.22 | | | 3 | Pp | SOD | |
| 39 | 3 | SOD | Pp | 5 | 29 | 7 | 3 | NA | | | | NA | NA | | | 3 | Pp | SOD | |
| 40 | 3 | SOD | Pp | 5 | 20 | 8 | 18 | 36.5 | 33.55 | 33.73 | | 24.66 | 1.48 | 1.361 | 1.368 | 3 | Pp | SOD | 33.74 |
| 41 | 3 | SOD | Np | 1 | 100 | 1 | 23 | NA | | | | NA | NA | | | 3 | Np | SOD | |
| 42 | 3 | SOD | Np | 1 | 67 | 2 | 1 | 0.2 | | | | 24.66 | 0.01 | | | 3 | Np | SOD | |
| 43 | 3 | SOD | Np | 1 | 28 | 3 | 7 | 1.4 | | | | 24.66 | 0.06 | | | 3 | Np | SOD | |
| 44 | 3 | SOD | Np | 1 | 70 | 4 | 24 | 6.3 | | | | 24.66 | 0.26 | | | 3 | Np | SOD | |
| 45 | 3 | SOD | Np | 1 | 8 | 5 | 16 | 26.7 | | | | 24.66 | 1.08 | | | 3 | Np | SOD | |
| 46 | 3 | SOD | Np | 1 | 2 | 6 | 14 | 27.8 | | | | 24.66 | 1.13 | | | 3 | Np | SOD | |
| 47 | 3 | SOD | Np | 1 | 93 | 7 | 4 | 0.4 | | | | 24.66 | 0.02 | | | 3 | Np | SOD | |
| 48 | 3 | SOD | Np | 1 | 13 | 8 | 19 | 29.7 | 13.21 | | | 24.66 | 1.20 | 0.536 | | 3 | Np | SOD | |
| 49 | 3 | SOD | Np | 2 | 92 | 1 | 15 | 0.2 | | | | 24.66 | 0.01 | | | 3 | Np | SOD | |
| 50 | 3 | SOD | Np | 2 | 23 | 2 | 15 | 0.6 | | | | 24.66 | 0.02 | | | 3 | Np | SOD | |
| 51 | 3 | SOD | Np | 2 | 41 | 3 | 23 | 33.0 | | | | 24.66 | 1.34 | | | 3 | Np | SOD | |
| 52 | 3 | SOD | Np | 2 | 22 | 4 | 15 | 13.5 | | | | 24.66 | 0.55 | | | 3 | Np | SOD | |
| 53 | 3 | SOD | Np | 2 | 83 | 5 | 11 | 0.3 | | | | 24.66 | 0.01 | | | 3 | Np | SOD | |
| 54 | 3 | SOD | Np | 2 | 4 | 6 | 11 | 20.7 | | | | 24.66 | 0.84 | | | 3 | Np | SOD | |
| 55 | 3 | SOD | Np | 2 | 6 | 7 | 5 | 18.8 | | | | 24.66 | 0.76 | | | 3 | Np | SOD | |
| 56 | 3 | SOD | Np | 2 | 12 | 8 | 13 | 22.1 | 13.65 | | | 24.66 | 0.90 | 0.554 | | 3 | Np | SOD | |
| 57 | 3 | SOD | Np | 3 | 32 | 1 | 1 | 28.9 | | | | 24.66 | 1.17 | | | 3 | Np | SOD | |
| 58 | 3 | SOD | Np | 3 | 85 | 2 | 16 | 20.9 | | | | 24.66 | 0.85 | | | 3 | Np | SOD | |
| 59 | 3 | SOD | Np | 3 | 7 | 3 | 22 | 13.0 | | | | 24.66 | 0.53 | | | 3 | Np | SOD | |
| 60 | 3 | SOD | Np | 3 | 1 | 4 | 18 | 0.9 | | | | 24.66 | 0.04 | | | 3 | Np | SOD | |
| 61 | 3 | SOD | Np | 3 | 73 | 5 | 20 | 0.2 | | | | 24.66 | 0.01 | | | 3 | Np | SOD | |
| 62 | 3 | SOD | Np | 3 | 5 | 6 | 4 | 1.3 | | | | 24.66 | 0.05 | | | 3 | Np | SOD | |
| 63 | 3 | SOD | Np | 3 | 15 | 7 | 23 | 0.4 | | | | 24.66 | 0.02 | | | 3 | Np | SOD | |
| 64 | 3 | SOD | Np | 3 | 46 | 8 | 5 | 27.5 | 11.64 | | | 24.66 | 1.12 | 0.472 | | 3 | Np | SOD | |
| 65 | 3 | SOD | Np | 4 | 78 | 1 | 5 | 0.7 | | | | 24.66 | 0.03 | | | 3 | Np | SOD | |
| 66 | 3 | SOD | Np | 4 | 97 | 2 | 6 | 34.1 | | | | 24.66 | 1.38 | | | 3 | Np | SOD | |
| 67 | 3 | SOD | Np | 4 | 47 | 3 | 3 | 1.0 | | | | 24.66 | 0.04 | | | 3 | Np | SOD | |
| 68 | 3 | SOD | Np | 4 | 80 | 4 | 20 | 0.8 | | | | 24.66 | 0.03 | | | 3 | Np | SOD | |
| 69 | 3 | SOD | Np | 4 | 26 | 5 | 15 | 28.9 | | | | 24.66 | 1.17 | | | 3 | Np | SOD | |
| 70 | 3 | SOD | Np | 4 | 25 | 6 | 9 | 4.8 | | | | 24.66 | 0.19 | | | 3 | Np | SOD | |
| 71 | 3 | SOD | Np | 4 | 68 | 7 | 24 | 30.2 | | | | 24.66 | 1.22 | | | 3 | Np | SOD | |
| 72 | 3 | SOD | Np | 4 | 65 | 8 | 11 | NA | 14.36 | | | NA | NA | 0.582 | | 3 | Np | SOD | |
| 73 | 3 | SOD | Np | 5 | 74 | 1 | 20 | 27.2 | | | | 24.66 | 1.10 | | | 3 | Np | SOD | |
| 74 | 3 | SOD | Np | 5 | 11 | 2 | 9 | 29.2 | | | | 24.66 | 1.18 | | | 3 | Np | SOD | |
| 75 | 3 | SOD | Np | 5 | 21 | 3 | 19 | 30.8 | | | | 24.66 | 1.25 | | | 3 | Np | SOD | |
| 76 | 3 | SOD | Np | 5 | 98 | 4 | 9 | 0.9 | | | | 24.66 | 0.04 | | | 3 | Np | SOD | |
| 77 | 3 | SOD | Np | 5 | 33 | 5 | 25 | 31.0 | | | | 24.66 | 1.26 | | | 3 | Np | SOD | |
| 78 | 3 | SOD | Np | 5 | 84 | 6 | 17 | 0.5 | | | | 24.66 | 0.02 | | | 3 | Np | SOD | |
| 79 | 3 | SOD | Np | 5 | 52 | 7 | 17 | 0.2 | | | | 24.66 | 0.01 | | | 3 | Np | SOD | |
| 80 | 3 | SOD | Np | 5 | 10 | 8 | 24 | 22.5 | 17.79 | 14.13 | | 24.66 | 0.91 | 0.721 | 0.573 | 3 | Np | SOD | 14.15 |
| 91 | 3 | SOD | Null | NA | 49 | 1 | 19 | 32.6 | | | | 24.66 | 1.32 | | | 3 | Null | SOD | |
| 92 | 3 | SOD | Null | NA | 60 | 2 | 7 | 40.0 | | | | 24.66 | 1.62 | | | 3 | Null | SOD | |
| 93 | 3 | SOD | Null | NA | 86 | 3 | 17 | 37.2 | | | | 24.66 | 1.51 | | | 3 | Null | SOD | |
| 94 | 3 | SOD | Null | NA | 89 | 4 | 7 | 28.8 | | | | 24.66 | 1.17 | | | 3 | Null | SOD | |
| 95 | 3 | SOD | Null | NA | 95 | 5 | 2 | 30.7 | | | | 24.66 | 1.25 | | | 3 | Null | SOD | |
| 96 | 3 | SOD | Null | NA | 77 | 6 | 15 | 31.2 | | | | 24.66 | 1.27 | | | 3 | Null | SOD | |
| 97 | 3 | SOD | Null | NA | 45 | 7 | 19 | 25.5 | | | | 24.66 | 1.03 | | | 3 | Null | SOD | |

# Supplemental Table S1

| | | | | | | | | | | | | |
|---|---|---|---|---|---|---|---|---|---|---|---|---|
| 14.15 | 2.23 | | | | 31.92 | 0.99 | | | | 3 | Pp | SOD |
| 14.15 | 2.47 | | | | 31.92 | 1.10 | | | | 3 | Pp | SOD |
| 14.15 | 1.82 | | | | 31.92 | 0.81 | | | | 3 | Pp | SOD |
| 14.15 | 2.12 | | Generation 3 | | 31.92 | 0.94 | | Generation 3 | | 3 | Pp | SOD |
| NA | NA | | Average | StDev | NA | NA | | Average | StDev | 3 | Pp | SOD |
| 14.15 | 2.58 | 2.37 | 2.384 | 0.111 | 31.92 | 1.14 | 1.051 | 1.057 | 0.049 | 3 | Pp | SOD |
| | | | | | | | | | | 3 | Np | SOD |
| | | | | | | | | | | 3 | Np | SOD |
| | | | | | | | | | | 3 | Np | SOD |
| | | | | | | | | | | 3 | Np | SOD |
| | | | | | | | | | | 3 | Np | SOD |
| | | | | | | | | | | 3 | Np | SOD |
| | | | | | | | | | | 3 | Np | SOD |
| | | | | | | | | | | 3 | Np | SOD |
| | | | | | | | | | | 3 | Np | SOD |
| | | | | | | | | | | 3 | Np | SOD |
| | | | | | | | | | | 3 | Np | SOD |
| | | | | | | | | | | 3 | Np | SOD |
| | | | | | | | | | | 3 | Np | SOD |
| | | | | | | | | | | 3 | Np | SOD |
| | | | | | | | | | | 3 | Np | SOD |
| | | | | | | | | | | 3 | Np | SOD |
| | | | | | | | | | | 3 | Np | SOD |
| | | | | | | | | | | 3 | Np | SOD |
| | | | | | | | | | | 3 | Np | SOD |
| | | | | | | | | | | 3 | Np | SOD |
| | | | | | | | | | | 3 | Np | SOD |
| | | | | | | | | | | 3 | Np | SOD |
| | | | | | | | | | | 3 | Np | SOD |
| | | | | | | | | | | 3 | Np | SOD |
| | | | | | | | | | | 3 | Np | SOD |
| | | | | | | | | | | 3 | Np | SOD |
| | | | | | | | | | | 3 | Np | SOD |
| | | | | | | | | | | 3 | Np | SOD |
| | | | | | | | | | | 3 | Np | SOD |
| | | | | | | | | | | 3 | Np | SOD |
| | | | | | | | | | | 3 | Np | SOD |
| | | | | | | | | | | 3 | Np | SOD |
| | | | | | | | | | | 3 | Np | SOD |
| | | | | | | | | | | 3 | Np | SOD |
| | | | | | | | | | | 3 | Np | SOD |
| | | | | | | | | | | 3 | Np | SOD |
| | | | | | | | | | | 3 | Np | SOD |
| | | | | | | | | | | 3 | Np | SOD |
| | | | | | | | | | | 3 | Np | SOD |
| | | | | | | | | | | 3 | Np | SOD |
| | | | | | | | | | | 3 | Null | SOD |
| | | | | | | | | | | 3 | Null | SOD |
| | | | | | | | | | | 3 | Null | SOD |
| | | | | | | | | | | 3 | Null | SOD |
| | | | | | | | | | | 3 | Null | SOD |
| | | | | | | | | | | 3 | Null | SOD |
| | | | | | | | | | | 3 | Null | SOD |

# Supplemental Table S1

| | | | | | | | | | | | | | | | | | | | |
|---|---|---|---|---|---|---|---|---|---|---|---|---|---|---|---|---|---|---|---|
| 98 | 3 | SOD | Null | NA | 56 | 8 | 4 | 32.5 | | | | 24.66 | 1.32 | | | 3 | Null | SOD | |
| 99 | 3 | SOD | Null | NA | 38 | 1 | 12 | 32.9 | | | Grand Average | 24.66 | 1.33 | | | 3 | Null | SOD | |
| 100 | 3 | SOD | Null | NA | 35 | 7 | 1 | 27.8 | 31.92 | | 24.66 | 24.66 | 1.13 | 1.294 | | 3 | Null | SOD | 31.92 |
| 101 | 3 | ALU | Pp | 1 | 133 | 1 | 22 | 50.1 | | | | 36.81 | 1.36 | | | 3 | Pp | ALU | |
| 102 | 3 | ALU | Pp | 1 | 108 | 2 | 22 | 42.2 | | | | 36.81 | 1.15 | | | 3 | Pp | ALU | |
| 103 | 3 | ALU | Pp | 1 | 143 | 3 | 20 | 45.0 | | | | 36.81 | 1.22 | | | 3 | Pp | ALU | |
| 104 | 3 | ALU | Pp | 1 | 122 | 4 | 6 | 48.3 | | | | 36.81 | 1.31 | | | 3 | Pp | ALU | |
| 105 | 3 | ALU | Pp | 1 | 132 | 5 | 8 | 50.0 | | | | 36.81 | 1.36 | | | 3 | Pp | ALU | |
| 106 | 3 | ALU | Pp | 1 | 158 | 6 | 7 | 44.4 | | | | 36.81 | 1.21 | | | 3 | Pp | ALU | |
| 107 | 3 | ALU | Pp | 1 | 172 | 7 | 20 | 51.3 | | | | 36.81 | 1.39 | | | 3 | Pp | ALU | |
| 108 | 3 | ALU | Pp | 1 | 181 | 8 | 21 | 50.5 | 47.73 | | | 36.81 | 1.37 | 1.296 | | 3 | Pp | ALU | |
| 109 | 3 | ALU | Pp | 2 | 170 | 1 | 9 | 47.2 | | | | 36.81 | 1.28 | | | 3 | Pp | ALU | |
| 110 | 3 | ALU | Pp | 2 | 144 | 2 | 8 | 54.6 | | | | 36.81 | 1.48 | | | 3 | Pp | ALU | |
| 111 | 3 | ALU | Pp | 2 | 120 | 3 | 15 | 46.2 | | | | 36.81 | 1.25 | | | 3 | Pp | ALU | |
| 112 | 3 | ALU | Pp | 2 | 116 | 4 | 12 | 49.1 | | | | 36.81 | 1.33 | | | 3 | Pp | ALU | |
| 113 | 3 | ALU | Pp | 2 | 167 | 5 | 19 | 49.1 | | | | 36.81 | 1.33 | | | 3 | Pp | ALU | |
| 114 | 3 | ALU | Pp | 2 | 150 | 6 | 19 | 45.1 | | | | 36.81 | 1.23 | | | 3 | Pp | ALU | |
| 115 | 3 | ALU | Pp | 2 | 105 | 7 | 9 | 35.1 | | | | 36.81 | 0.95 | | | 3 | Pp | ALU | |
| 116 | 3 | ALU | Pp | 2 | 168 | 8 | 3 | 46.6 | 46.63 | | | 36.81 | 1.27 | 1.267 | | 3 | Pp | ALU | |
| 117 | 3 | ALU | Pp | 3 | 163 | 1 | 17 | 29.1 | | | | 36.81 | 0.79 | | | 3 | Pp | ALU | |
| 118 | 3 | ALU | Pp | 3 | 184 | 2 | 14 | 40.9 | | | | 36.81 | 1.11 | | | 3 | Pp | ALU | |
| 119 | 3 | ALU | Pp | 3 | 110 | 3 | 2 | 52.2 | | | | 36.81 | 1.42 | | | 3 | Pp | ALU | |
| 120 | 3 | ALU | Pp | 3 | 175 | 4 | 3 | 39.8 | | | | 36.81 | 1.08 | | | 3 | Pp | ALU | |
| 121 | 3 | ALU | Pp | 3 | 199 | 5 | 23 | 36.9 | | | | 36.81 | 1.00 | | | 3 | Pp | ALU | |
| 122 | 3 | ALU | Pp | 3 | 180 | 6 | 6 | 40.5 | | | | 36.81 | 1.10 | | | 3 | Pp | ALU | |
| 123 | 3 | ALU | Pp | 3 | 142 | 7 | 6 | 35.2 | | | | 36.81 | 0.96 | | | 3 | Pp | ALU | |
| 124 | 3 | ALU | Pp | 3 | 126 | 8 | 16 | 45.7 | 40.04 | | | 36.81 | 1.24 | 1.088 | | 3 | Pp | ALU | |
| 125 | 3 | ALU | Pp | 4 | 178 | 1 | 11 | 39.7 | | | | 36.81 | 1.08 | | | 3 | Pp | ALU | |
| 126 | 3 | ALU | Pp | 4 | 140 | 2 | 17 | 47.6 | | | | 36.81 | 1.29 | | | 3 | Pp | ALU | |
| 127 | 3 | ALU | Pp | 4 | 164 | 3 | 25 | 46.6 | | | | 36.81 | 1.27 | | | 3 | Pp | ALU | |
| 128 | 3 | ALU | Pp | 4 | 129 | 4 | 21 | 44.7 | | | | 36.81 | 1.21 | | | 3 | Pp | ALU | |
| 129 | 3 | ALU | Pp | 4 | 191 | 5 | 1 | 38.4 | | | | 36.81 | 1.04 | | | 3 | Pp | ALU | |
| 130 | 3 | ALU | Pp | 4 | 174 | 6 | 25 | 40.2 | | | | 36.81 | 1.09 | | | 3 | Pp | ALU | |
| 131 | 3 | ALU | Pp | 4 | 165 | 7 | 21 | 40.8 | | | | 36.81 | 1.11 | | | 3 | Pp | ALU | |
| 132 | 3 | ALU | Pp | 4 | 187 | 8 | 7 | 43.3 | 42.66 | | | 36.81 | 1.18 | 1.159 | | 3 | Pp | ALU | |
| 133 | 3 | ALU | Pp | 5 | 188 | 1 | 4 | 55.0 | | | | 36.81 | 1.49 | | | 3 | Pp | ALU | |
| 134 | 3 | ALU | Pp | 5 | 145 | 2 | 2 | 24.0 | | | | 36.81 | 0.65 | | | 3 | Pp | ALU | |
| 135 | 3 | ALU | Pp | 5 | 119 | 3 | 18 | 52.0 | | | | 36.81 | 1.41 | | | 3 | Pp | ALU | |
| 136 | 3 | ALU | Pp | 5 | 183 | 4 | 11 | 40.8 | | | | 36.81 | 1.11 | | | 3 | Pp | ALU | |
| 137 | 3 | ALU | Pp | 5 | 179 | 5 | 14 | 48.6 | | | | 36.81 | 1.32 | | | 3 | Pp | ALU | |
| 138 | 3 | ALU | Pp | 5 | 200 | 6 | 22 | 42.3 | | | | 36.81 | 1.15 | | | 3 | Pp | ALU | |
| 139 | 3 | ALU | Pp | 5 | 148 | 7 | 10 | 42.6 | | | | 36.81 | 1.16 | | | 3 | Pp | ALU | |
| 140 | 3 | ALU | Pp | 5 | 130 | 8 | 15 | 32.0 | 42.16 | 43.84 | | 36.81 | 0.87 | 1.145 | 1.191 | 3 | Pp | ALU | 43.84 |
| 141 | 3 | ALU | Np | 1 | 198 | 1 | 16 | 27.0 | | | | 36.81 | 0.73 | | | 3 | Np | ALU | |
| 142 | 3 | ALU | Np | 1 | 134 | 2 | 25 | 41.1 | | | | 36.81 | 1.12 | | | 3 | Np | ALU | |
| 143 | 3 | ALU | Np | 1 | 111 | 3 | 10 | 26.5 | | | | 36.81 | 0.72 | | | 3 | Np | ALU | |
| 144 | 3 | ALU | Np | 1 | 173 | 4 | 8 | 32.0 | | | | 36.81 | 0.87 | | | 3 | Np | ALU | |
| 145 | 3 | ALU | Np | 1 | 124 | 5 | 24 | 34.4 | | | | 36.81 | 0.93 | | | 3 | Np | ALU | |
| 146 | 3 | ALU | Np | 1 | 152 | 6 | 24 | 1.2 | | | | 36.81 | 0.03 | | | 3 | Np | ALU | |
| 147 | 3 | ALU | Np | 1 | 141 | 7 | 15 | 0.9 | | | | 36.81 | 0.02 | | | 3 | Np | ALU | |
| 148 | 3 | ALU | Np | 1 | 194 | 8 | 22 | 0.6 | 20.46 | | | 36.81 | 0.02 | 0.556 | | 3 | Np | ALU | |
| 149 | 3 | ALU | Np | 2 | 192 | 1 | 25 | 30.6 | | | | 36.81 | 0.83 | | | 3 | Np | ALU | |
| 150 | 3 | ALU | Np | 2 | 154 | 2 | 19 | 35.4 | | | | 36.81 | 0.96 | | | 3 | Np | ALU | |

# Supplemental Table S1

| | | | | | | | | | | | | |
|---|---|---|---|---|---|---|---|---|---|---|---|---|
| | | | | | | | | | | 3 | Null | SOD |
| | | | | | | | | | | 3 | Null | SOD |
| | | | | | | | | | | 3 | Null | SOD |
| 28.69 | 1.75 | | | | 41.29 | 1.21 | | | | 3 | Pp | ALU |
| 28.69 | 1.47 | | | | 41.29 | 1.02 | | | | 3 | Pp | ALU |
| 28.69 | 1.57 | | | | 41.29 | 1.09 | | | | 3 | Pp | ALU |
| 28.69 | 1.68 | | | | 41.29 | 1.17 | | | | 3 | Pp | ALU |
| 28.69 | 1.74 | | | | 41.29 | 1.21 | | | | 3 | Pp | ALU |
| 28.69 | 1.55 | | | | 41.29 | 1.08 | | | | 3 | Pp | ALU |
| 28.69 | 1.79 | | | | 41.29 | 1.24 | | | | 3 | Pp | ALU |
| 28.69 | 1.76 | 1.66 | | | 41.29 | 1.22 | 1.156 | | | 3 | Pp | ALU |
| 28.69 | 1.65 | | | | 41.29 | 1.14 | | | | 3 | Pp | ALU |
| 28.69 | 1.90 | | | | 41.29 | 1.32 | | | | 3 | Pp | ALU |
| 28.69 | 1.61 | | | | 41.29 | 1.12 | | | | 3 | Pp | ALU |
| 28.69 | 1.71 | | | | 41.29 | 1.19 | | | | 3 | Pp | ALU |
| 28.69 | 1.71 | | | | 41.29 | 1.19 | | | | 3 | Pp | ALU |
| 28.69 | 1.57 | | | | 41.29 | 1.09 | | | | 3 | Pp | ALU |
| 28.69 | 1.22 | | | | 41.29 | 0.85 | | | | 3 | Pp | ALU |
| 28.69 | 1.62 | 1.63 | | | 41.29 | 1.13 | 1.129 | | | 3 | Pp | ALU |
| 28.69 | 1.01 | | | | 41.29 | 0.70 | | | | 3 | Pp | ALU |
| 28.69 | 1.43 | | | | 41.29 | 0.99 | | | | 3 | Pp | ALU |
| 28.69 | 1.82 | | | | 41.29 | 1.26 | | | | 3 | Pp | ALU |
| 28.69 | 1.39 | | | | 41.29 | 0.96 | | | | 3 | Pp | ALU |
| 28.69 | 1.29 | | | | 41.29 | 0.89 | | | | 3 | Pp | ALU |
| 28.69 | 1.41 | | | | 41.29 | 0.98 | | | | 3 | Pp | ALU |
| 28.69 | 1.23 | | | | 41.29 | 0.85 | | | | 3 | Pp | ALU |
| 28.69 | 1.59 | 1.40 | | | 41.29 | 1.11 | 0.970 | | | 3 | Pp | ALU |
| 28.69 | 1.38 | | | | 41.29 | 0.96 | | | | 3 | Pp | ALU |
| 28.69 | 1.66 | | | | 41.29 | 1.15 | | | | 3 | Pp | ALU |
| 28.69 | 1.62 | | | | 41.29 | 1.13 | | | | 3 | Pp | ALU |
| 28.69 | 1.56 | | | | 41.29 | 1.08 | | | | 3 | Pp | ALU |
| 28.69 | 1.34 | | | | 41.29 | 0.93 | | | | 3 | Pp | ALU |
| 28.69 | 1.40 | | | | 41.29 | 0.97 | | | | 3 | Pp | ALU |
| 28.69 | 1.42 | | | | 41.29 | 0.99 | | | | 3 | Pp | ALU |
| 28.69 | 1.51 | 1.49 | | | 41.29 | 1.05 | 1.033 | | | 3 | Pp | ALU |
| 28.69 | 1.92 | | | | 41.29 | 1.33 | | | | 3 | Pp | ALU |
| 28.69 | 0.84 | | | | 41.29 | 0.58 | | | | 3 | Pp | ALU |
| 28.69 | 1.81 | | | | 41.29 | 1.26 | | | | 3 | Pp | ALU |
| 28.69 | 1.42 | | | | 41.29 | 0.99 | | | | 3 | Pp | ALU |
| 28.69 | 1.69 | | | | 41.29 | 1.18 | | | | 3 | Pp | ALU |
| 28.69 | 1.47 | | Generation 3 | | 41.29 | 1.02 | | Generation 3 | | 3 | Pp | ALU |
| 28.69 | 1.48 | | Average | StDev | 41.29 | 1.03 | | Average | StDev | 3 | Pp | ALU |
| 28.69 | 1.12 | 1.47 | 1.528 | 0.112 | 41.29 | 0.78 | 1.021 | 1.062 | 0.078 | 3 | Pp | ALU |
| | | | | | | | | | | 3 | Np | ALU |
| | | | | | | | | | | 3 | Np | ALU |
| | | | | | | | | | | 3 | Np | ALU |
| | | | | | | | | | | 3 | Np | ALU |
| | | | | | | | | | | 3 | Np | ALU |
| | | | | | | | | | | 3 | Np | ALU |
| | | | | | | | | | | 3 | Np | ALU |
| | | | | | | | | | | 3 | Np | ALU |
| | | | | | | | | | | 3 | Np | ALU |
| | | | | | | | | | | 3 | Np | ALU |

# Supplemental Table S1

| | | | | | | | | | | | | | | | | | | | |
|---|---|---|---|---|---|---|---|---|---|---|---|---|---|---|---|---|---|---|---|
| 151 | 3 | ALU | Np | 2 | 137 | 3 | 8 | NA | | | | NA | NA | | | 3 | Np | ALU | |
| 152 | 3 | ALU | Np | 2 | 197 | 4 | 17 | 41.8 | | | | 36.81 | 1.14 | | | 3 | Np | ALU | |
| 153 | 3 | ALU | Np | 2 | 117 | 5 | 9 | 27.3 | | | | 36.81 | 0.74 | | | 3 | Np | ALU | |
| 154 | 3 | ALU | Np | 2 | 135 | 6 | 18 | 31.1 | | | | 36.81 | 0.84 | | | 3 | Np | ALU | |
| 155 | 3 | ALU | Np | 2 | 131 | 7 | 13 | 43.3 | | | | 36.81 | 1.18 | | | 3 | Np | ALU | |
| 156 | 3 | ALU | Np | 2 | 182 | 8 | 17 | 41.0 | 35.79 | | | 36.81 | 1.11 | 0.972 | | 3 | Np | ALU | |
| 157 | 3 | ALU | Np | 3 | 190 | 1 | 21 | 9.4 | | | | 36.81 | 0.26 | | | 3 | Np | ALU | |
| 158 | 3 | ALU | Np | 3 | 138 | 2 | 13 | 37.5 | | | | 36.81 | 1.02 | | | 3 | Np | ALU | |
| 159 | 3 | ALU | Np | 3 | 196 | 3 | 16 | 46.4 | | | | 36.81 | 1.26 | | | 3 | Np | ALU | |
| 160 | 3 | ALU | Np | 3 | 101 | 4 | 1 | 42.6 | | | | 36.81 | 1.16 | | | 3 | Np | ALU | |
| 161 | 3 | ALU | Np | 3 | 153 | 5 | 22 | 42.0 | | | | 36.81 | 1.14 | | | 3 | Np | ALU | |
| 162 | 3 | ALU | Np | 3 | 149 | 6 | 12 | 41.8 | | | | 36.81 | 1.14 | | | 3 | Np | ALU | |
| 163 | 3 | ALU | Np | 3 | 161 | 7 | 11 | 32.8 | | | | 36.81 | 0.89 | | | 3 | Np | ALU | |
| 164 | 3 | ALU | Np | 3 | 193 | 8 | 8 | 0.2 | 31.59 | | | 36.81 | 0.01 | 0.858 | | 3 | Np | ALU | |
| 165 | 3 | ALU | Np | 4 | 195 | 1 | 10 | 36.5 | | | | 36.81 | 0.99 | | | 3 | Np | ALU | |
| 166 | 3 | ALU | Np | 4 | 106 | 2 | 3 | 8.3 | | | | 36.81 | 0.23 | | | 3 | Np | ALU | |
| 167 | 3 | ALU | Np | 4 | 159 | 3 | 12 | 19.3 | | | | 36.81 | 0.52 | | | 3 | Np | ALU | |
| 168 | 3 | ALU | Np | 4 | 169 | 4 | 14 | 41.7 | | | | 36.81 | 1.13 | | | 3 | Np | ALU | |
| 169 | 3 | ALU | Np | 4 | 171 | 5 | 17 | 37.9 | | | | 36.81 | 1.03 | | | 3 | Np | ALU | |
| 170 | 3 | ALU | Np | 4 | 109 | 6 | 2 | 27.4 | | | | 36.81 | 0.74 | | | 3 | Np | ALU | |
| 171 | 3 | ALU | Np | 4 | 123 | 7 | 7 | 32.0 | | | | 36.81 | 0.87 | | | 3 | Np | ALU | |
| 172 | 3 | ALU | Np | 4 | 118 | 8 | 12 | 31.6 | 29.34 | | | 36.81 | 0.86 | 0.797 | | 3 | Np | ALU | |
| 173 | 3 | ALU | Np | 5 | 128 | 1 | 7 | 40.5 | | | | 36.81 | 1.10 | | | 3 | Np | ALU | |
| 174 | 3 | ALU | Np | 5 | 136 | 2 | 20 | 38.1 | | | | 36.81 | 1.03 | | | 3 | Np | ALU | |
| 175 | 3 | ALU | Np | 5 | 185 | 3 | 1 | 0.3 | | | | 36.81 | 0.01 | | | 3 | Np | ALU | |
| 176 | 3 | ALU | Np | 5 | 127 | 4 | 4 | 32.7 | | | | 36.81 | 0.89 | | | 3 | Np | ALU | |
| 177 | 3 | ALU | Np | 5 | 151 | 5 | 13 | 0.7 | | | | 36.81 | 0.02 | | | 3 | Np | ALU | |
| 178 | 3 | ALU | Np | 5 | 177 | 6 | 21 | 29.7 | | | | 36.81 | 0.81 | | | 3 | Np | ALU | |
| 179 | 3 | ALU | Np | 5 | 189 | 7 | 12 | 40.5 | | | | 36.81 | 1.10 | | | 3 | Np | ALU | |
| 180 | 3 | ALU | Np | 5 | 102 | 8 | 14 | 34.7 | 27.15 | 28.86 | | 36.81 | 0.94 | 0.737 | 0.784 | 3 | Np | ALU | 28.69 |
| 191 | 3 | ALU | Null | NA | 147 | 1 | 18 | 47.2 | | | | 36.81 | 1.28 | | | 3 | Null | ALU | |
| 192 | 3 | ALU | Null | NA | 186 | 2 | 12 | NA | | | | NA | NA | | | 3 | Null | ALU | |
| 193 | 3 | ALU | Null | NA | 176 | 3 | 4 | NA | | | | NA | NA | | | 3 | Null | ALU | |
| 194 | 3 | ALU | Null | NA | 162 | 4 | 10 | 39.7 | | | | 36.81 | 1.08 | | | 3 | Null | ALU | |
| 195 | 3 | ALU | Null | NA | 146 | 5 | 7 | 41.4 | | | | 36.81 | 1.12 | | | 3 | Null | ALU | |
| 196 | 3 | ALU | Null | NA | 107 | 6 | 8 | 36.4 | | | | 36.81 | 0.99 | | | 3 | Null | ALU | |
| 197 | 3 | ALU | Null | NA | 103 | 7 | 25 | 45.1 | | | | 36.81 | 1.23 | | | 3 | Null | ALU | |
| 198 | 3 | ALU | Null | NA | 157 | 8 | 9 | 31.5 | | | | 36.81 | 0.86 | | | 3 | Null | ALU | |
| 199 | 3 | ALU | Null | NA | 113 | 2 | 23 | 41.8 | | | Grand Average | 36.81 | 1.14 | | | 3 | Null | ALU | |
| 200 | 3 | ALU | Null | NA | 166 | 6 | 3 | 47.2 | 41.29 | | 36.81 | 36.81 | 1.28 | 1.122 | | 3 | Null | ALU | 41.29 |
| 1 | 4 | SOD | Pp | 1 | 2 | 1 | 21 | 16.7 | | | | 16.14 | 1.03 | | | 4 | Pp | SOD | |
| 2 | 4 | SOD | Pp | 1 | 21 | 2 | 19 | 20.7 | | | | 16.14 | 1.28 | | | 4 | Pp | SOD | |
| 3 | 4 | SOD | Pp | 1 | 27 | 3 | 25 | 20.8 | | | | 16.14 | 1.29 | | | 4 | Pp | SOD | |
| 4 | 4 | SOD | Pp | 1 | 77 | 4 | 14 | 20.7 | | | | 16.14 | 1.28 | | | 4 | Pp | SOD | |
| 5 | 4 | SOD | Pp | 1 | 82 | 5 | 20 | 13.3 | | | | 16.14 | 0.82 | | | 4 | Pp | SOD | |
| 6 | 4 | SOD | Pp | 1 | 61 | 6 | 13 | 22.5 | | | | 16.14 | 1.39 | | | 4 | Pp | SOD | |
| 7 | 4 | SOD | Pp | 1 | 76 | 7 | 8 | 21.6 | | | | 16.14 | 1.34 | | | 4 | Pp | SOD | |
| 8 | 4 | SOD | Pp | 1 | 58 | 8 | 5 | 22.2 | 19.81 | | | 16.14 | 1.38 | 1.228 | | 4 | Pp | SOD | |
| 9 | 4 | SOD | Pp | 2 | 92 | 1 | 6 | 16.0 | | | | 16.14 | 0.99 | | | 4 | Pp | SOD | |
| 10 | 4 | SOD | Pp | 2 | 72 | 2 | 5 | 15.9 | | | | 16.14 | 0.99 | | | 4 | Pp | SOD | |
| 11 | 4 | SOD | Pp | 2 | 33 | 3 | 1 | 16.8 | | | | 16.14 | 1.04 | | | 4 | Pp | SOD | |
| 12 | 4 | SOD | Pp | 2 | 38 | 4 | 2 | 20.3 | | | | 16.14 | 1.26 | | | 4 | Pp | SOD | |
| 13 | 4 | SOD | Pp | 2 | 54 | 5 | 23 | 18.3 | | | | 16.14 | 1.13 | | | 4 | Pp | SOD | |

# Supplemental Table S1

| | | | | | | | | | | | | |
|---|---|---|---|---|---|---|---|---|---|---|---|---|
| | | | | | | | | | | 3 | Np | ALU |
| | | | | | | | | | | 3 | Np | ALU |
| | | | | | | | | | | 3 | Np | ALU |
| | | | | | | | | | | 3 | Np | ALU |
| | | | | | | | | | | 3 | Np | ALU |
| | | | | | | | | | | 3 | Np | ALU |
| | | | | | | | | | | 3 | Np | ALU |
| | | | | | | | | | | 3 | Np | ALU |
| | | | | | | | | | | 3 | Np | ALU |
| | | | | | | | | | | 3 | Np | ALU |
| | | | | | | | | | | 3 | Np | ALU |
| | | | | | | | | | | 3 | Np | ALU |
| | | | | | | | | | | 3 | Np | ALU |
| | | | | | | | | | | 3 | Np | ALU |
| | | | | | | | | | | 3 | Np | ALU |
| | | | | | | | | | | 3 | Np | ALU |
| | | | | | | | | | | 3 | Np | ALU |
| | | | | | | | | | | 3 | Np | ALU |
| | | | | | | | | | | 3 | Np | ALU |
| | | | | | | | | | | 3 | Np | ALU |
| | | | | | | | | | | 3 | Np | ALU |
| | | | | | | | | | | 3 | Np | ALU |
| | | | | | | | | | | 3 | Np | ALU |
| | | | | | | | | | | 3 | Np | ALU |
| | | | | | | | | | | 3 | Np | ALU |
| | | | | | | | | | | 3 | Np | ALU |
| | | | | | | | | | | 3 | Np | ALU |
| | | | | | | | | | | 3 | Np | ALU |
| | | | | | | | | | | 3 | Np | ALU |
| | | | | | | | | | | 3 | Np | ALU |
| | | | | | | | | | | 3 | Null | ALU |
| | | | | | | | | | | 3 | Null | ALU |
| | | | | | | | | | | 3 | Null | ALU |
| | | | | | | | | | | 3 | Null | ALU |
| | | | | | | | | | | 3 | Null | ALU |
| | | | | | | | | | | 3 | Null | ALU |
| | | | | | | | | | | 3 | Null | ALU |
| | | | | | | | | | | 3 | Null | ALU |
| | | | | | | | | | | 3 | Null | ALU |
| | | | | | | | | | | 3 | Null | ALU |
| 9.40 | 1.78 | | | | 19.46 | 0.86 | | | | 4 | Pp | SOD |
| 9.40 | 2.20 | | | | 19.46 | 1.06 | | | | 4 | Pp | SOD |
| 9.40 | 2.21 | | | | 19.46 | 1.07 | | | | 4 | Pp | SOD |
| 9.40 | 2.20 | | | | 19.46 | 1.06 | | | | 4 | Pp | SOD |
| 9.40 | 1.41 | | | | 19.46 | 0.68 | | | | 4 | Pp | SOD |
| 9.40 | 2.39 | | | | 19.46 | 1.16 | | | | 4 | Pp | SOD |
| 9.40 | 2.30 | | | | 19.46 | 1.11 | | | | 4 | Pp | SOD |
| 9.40 | 2.36 | 2.11 | | | 19.46 | 1.14 | 1.018 | | | 4 | Pp | SOD |
| 9.40 | 1.70 | | | | 19.46 | 0.82 | | | | 4 | Pp | SOD |
| 9.40 | 1.69 | | | | 19.46 | 0.82 | | | | 4 | Pp | SOD |
| 9.40 | 1.79 | | | | 19.46 | 0.86 | | | | 4 | Pp | SOD |
| 9.40 | 2.16 | | | | 19.46 | 1.04 | | | | 4 | Pp | SOD |
| 9.40 | 1.95 | | | | 19.46 | 0.94 | | | | 4 | Pp | SOD |

# Supplemental Table S1

| | | | | | | | | | | | | | | | | | | | |
|---|---|---|---|---|---|---|---|---|---|---|---|---|---|---|---|---|---|---|---|
| 14 | 4 | SOD | Pp | 2 | 5 | 6 | 24 | 16.2 | | | | 16.14 | 1.00 | | | 4 | Pp | SOD | |
| 15 | 4 | SOD | Pp | 2 | 80 | 7 | 16 | 18.9 | | | | 16.14 | 1.17 | | | 4 | Pp | SOD | |
| 16 | 4 | SOD | Pp | 2 | 89 | 8 | 14 | 19.7 | 17.76 | | | 16.14 | 1.22 | 1.101 | | 4 | Pp | SOD | |
| 17 | 4 | SOD | Pp | 3 | 32 | 1 | 18 | 19.4 | | | | 16.14 | 1.20 | | | 4 | Pp | SOD | |
| 18 | 4 | SOD | Pp | 3 | 67 | 2 | 4 | 22.6 | | | | 16.14 | 1.40 | | | 4 | Pp | SOD | |
| 19 | 4 | SOD | Pp | 3 | 26 | 3 | 7 | 21.9 | | | | 16.14 | 1.36 | | | 4 | Pp | SOD | |
| 20 | 4 | SOD | Pp | 3 | 87 | 4 | 9 | 19.5 | | | | 16.14 | 1.21 | | | 4 | Pp | SOD | |
| 21 | 4 | SOD | Pp | 3 | 41 | 5 | 9 | 19.7 | | | | 16.14 | 1.22 | | | 4 | Pp | SOD | |
| 22 | 4 | SOD | Pp | 3 | 35 | 6 | 16 | 19.3 | | | | 16.14 | 1.20 | | | 4 | Pp | SOD | |
| 23 | 4 | SOD | Pp | 3 | 85 | 7 | 17 | 22.4 | | | | 16.14 | 1.39 | | | 4 | Pp | SOD | |
| 24 | 4 | SOD | Pp | 3 | 84 | 8 | 15 | 20.6 | 20.68 | | | 16.14 | 1.28 | 1.281 | | 4 | Pp | SOD | |
| 25 | 4 | SOD | Pp | 4 | 23 | 1 | 2 | 19.2 | | | | 16.14 | 1.19 | | | 4 | Pp | SOD | |
| 26 | 4 | SOD | Pp | 4 | 16 | 2 | 17 | 19.0 | | | | 16.14 | 1.18 | | | 4 | Pp | SOD | |
| 27 | 4 | SOD | Pp | 4 | 95 | 3 | 15 | 18.6 | | | | 16.14 | 1.15 | | | 4 | Pp | SOD | |
| 28 | 4 | SOD | Pp | 4 | 36 | 4 | 4 | 19.1 | | | | 16.14 | 1.18 | | | 4 | Pp | SOD | |
| 29 | 4 | SOD | Pp | 4 | 24 | 5 | 19 | 19.6 | | | | 16.14 | 1.21 | | | 4 | Pp | SOD | |
| 30 | 4 | SOD | Pp | 4 | 60 | 6 | 17 | 16.2 | | | | 16.14 | 1.00 | | | 4 | Pp | SOD | |
| 31 | 4 | SOD | Pp | 4 | 96 | 7 | 12 | NA | | | | NA | NA | | | 4 | Pp | SOD | |
| 32 | 4 | SOD | Pp | 4 | 28 | 8 | 8 | 19.2 | 18.70 | | | 16.14 | 1.19 | 1.159 | | 4 | Pp | SOD | |
| 33 | 4 | SOD | Pp | 5 | 6 | 1 | 11 | 18.2 | | | | 16.14 | 1.13 | | | 4 | Pp | SOD | |
| 34 | 4 | SOD | Pp | 5 | 12 | 2 | 18 | 19.8 | | | | 16.14 | 1.23 | | | 4 | Pp | SOD | |
| 35 | 4 | SOD | Pp | 5 | 98 | 3 | 23 | 21.4 | | | | 16.14 | 1.33 | | | 4 | Pp | SOD | |
| 36 | 4 | SOD | Pp | 5 | 46 | 4 | 5 | 18.1 | | | | 16.14 | 1.12 | | | 4 | Pp | SOD | |
| 37 | 4 | SOD | Pp | 5 | 86 | 5 | 2 | 22.2 | | | | 16.14 | 1.38 | | | 4 | Pp | SOD | |
| 38 | 4 | SOD | Pp | 5 | 19 | 6 | 8 | 23.2 | | | | 16.14 | 1.44 | | | 4 | Pp | SOD | |
| 39 | 4 | SOD | Pp | 5 | 9 | 7 | 13 | 25.6 | | | | 16.14 | 1.59 | | | 4 | Pp | SOD | |
| 40 | 4 | SOD | Pp | 5 | 42 | 8 | 4 | 23.0 | 21.44 | 19.68 | | 16.14 | 1.43 | 1.328 | 1.219 | 4 | Pp | SOD | 19.70 |
| 41 | 4 | SOD | Np | 1 | 71 | 3 | 13 | 0.4 | | | | 16.14 | 0.02 | | | 4 | Np | SOD | |
| 42 | 4 | SOD | Np | 1 | 39 | 4 | 22 | 13.8 | | | | 16.14 | 0.86 | | | 4 | Np | SOD | |
| 43 | 4 | SOD | Np | 1 | 31 | 5 | 14 | 15.9 | | | | 16.14 | 0.99 | | | 4 | Np | SOD | |
| 44 | 4 | SOD | Np | 1 | 55 | 6 | 4 | 0.5 | | | | 16.14 | 0.03 | | | 4 | Np | SOD | |
| 45 | 4 | SOD | Np | 1 | 34 | 7 | 19 | 0.8 | | | | 16.14 | 0.05 | | | 4 | Np | SOD | |
| 46 | 4 | SOD | Np | 1 | 90 | 8 | 9 | 12.0 | 7.23 | | | 16.14 | 0.74 | 0.448 | | 4 | Np | SOD | |
| 47 | 4 | SOD | Np | 2 | 15 | 1 | 7 | 14.4 | | | | 16.14 | 0.89 | | | 4 | Np | SOD | |
| 48 | 4 | SOD | Np | 2 | 37 | 2 | 14 | 12.3 | | | | 16.14 | 0.76 | | | 4 | Np | SOD | |
| 49 | 4 | SOD | Np | 2 | 52 | 5 | 11 | 16.8 | | | | 16.14 | 1.04 | | | 4 | Np | SOD | |
| 50 | 4 | SOD | Np | 2 | 45 | 6 | 2 | 2.0 | | | | 16.14 | 0.12 | | | 4 | Np | SOD | |
| 51 | 4 | SOD | Np | 2 | 69 | 7 | 20 | 13.3 | | | | 16.14 | 0.82 | | | 4 | Np | SOD | |
| 52 | 4 | SOD | Np | 2 | 49 | 8 | 10 | 13.1 | 11.98 | | | 16.14 | 0.81 | 0.743 | | 4 | Np | SOD | |
| 53 | 4 | SOD | Np | 3 | 3 | 1 | 10 | 12.8 | | | | 16.14 | 0.79 | | | 4 | Np | SOD | |
| 54 | 4 | SOD | Np | 3 | 40 | 2 | 2 | 12.2 | | | | 16.14 | 0.76 | | | 4 | Np | SOD | |
| 55 | 4 | SOD | Np | 3 | 59 | 3 | 6 | 8.7 | | | | 16.14 | 0.54 | | | 4 | Np | SOD | |
| 56 | 4 | SOD | Np | 3 | 74 | 4 | 21 | 0.8 | | | | 16.14 | 0.05 | | | 4 | Np | SOD | |
| 57 | 4 | SOD | Np | 3 | 50 | 7 | 18 | 0.4 | | | | 16.14 | 0.02 | | | 4 | Np | SOD | |
| 58 | 4 | SOD | Np | 3 | 51 | 8 | 2 | 20.7 | 9.27 | | | 16.14 | 1.28 | 0.574 | | 4 | Np | SOD | |
| 59 | 4 | SOD | Np | 4 | 18 | 1 | 16 | 0.6 | | | | 16.14 | 0.04 | | | 4 | Np | SOD | |
| 60 | 4 | SOD | Np | 4 | 79 | 2 | 21 | 11.4 | | | | 16.14 | 0.71 | | | 4 | Np | SOD | |
| 61 | 4 | SOD | Np | 4 | 47 | 3 | 24 | 17.7 | | | | 16.14 | 1.10 | | | 4 | Np | SOD | |
| 62 | 4 | SOD | Np | 4 | 29 | 4 | 8 | 20.1 | | | | 16.14 | 1.25 | | | 4 | Np | SOD | |
| 63 | 4 | SOD | Np | 4 | 94 | 5 | 3 | 24.1 | | | | 16.14 | 1.49 | | | 4 | Np | SOD | |
| 64 | 4 | SOD | Np | 4 | 20 | 6 | 11 | 2.5 | 12.73 | | | 16.14 | 0.15 | 0.789 | | 4 | Np | SOD | |
| 65 | 4 | SOD | Np | 5 | 81 | 1 | 25 | NA | | | | NA | NA | | | 4 | Np | SOD | |
| 66 | 4 | SOD | Np | 5 | 93 | 3 | 21 | 7.4 | | | | 16.14 | 0.46 | | | 4 | Np | SOD | |

# Supplemental Table S1

| | | | | | | | | | | | | |
|---|---|---|---|---|---|---|---|---|---|---|---|---|
| 9.40 | 1.72 | | | | 19.46 | 0.83 | | | | 4 | Pp | SOD |
| 9.40 | 2.01 | | | | 19.46 | 0.97 | | | | 4 | Pp | SOD |
| 9.40 | 2.10 | 1.89 | | | 19.46 | 1.01 | 0.913 | | | 4 | Pp | SOD |
| 9.40 | 2.06 | | | | 19.46 | 1.00 | | | | 4 | Pp | SOD |
| 9.40 | 2.40 | | | | 19.46 | 1.16 | | | | 4 | Pp | SOD |
| 9.40 | 2.33 | | | | 19.46 | 1.13 | | | | 4 | Pp | SOD |
| 9.40 | 2.07 | | | | 19.46 | 1.00 | | | | 4 | Pp | SOD |
| 9.40 | 2.10 | | | | 19.46 | 1.01 | | | | 4 | Pp | SOD |
| 9.40 | 2.05 | | | | 19.46 | 0.99 | | | | 4 | Pp | SOD |
| 9.40 | 2.38 | | | | 19.46 | 1.15 | | | | 4 | Pp | SOD |
| 9.40 | 2.19 | 2.20 | | | 19.46 | 1.06 | 1.063 | | | 4 | Pp | SOD |
| 9.40 | 2.04 | | | | 19.46 | 0.99 | | | | 4 | Pp | SOD |
| 9.40 | 2.02 | | | | 19.46 | 0.98 | | | | 4 | Pp | SOD |
| 9.40 | 1.98 | | | | 19.46 | 0.96 | | | | 4 | Pp | SOD |
| 9.40 | 2.03 | | | | 19.46 | 0.98 | | | | 4 | Pp | SOD |
| 9.40 | 2.09 | | | | 19.46 | 1.01 | | | | 4 | Pp | SOD |
| 9.40 | 1.72 | | | | 19.46 | 0.83 | | | | 4 | Pp | SOD |
| NA | NA | | | | NA | NA | | | | 4 | Pp | SOD |
| 9.40 | 2.04 | 1.99 | | | 19.46 | 0.99 | 0.961 | | | 4 | Pp | SOD |
| 9.40 | 1.94 | | | | 19.46 | 0.94 | | | | 4 | Pp | SOD |
| 9.40 | 2.11 | | | | 19.46 | 1.02 | | | | 4 | Pp | SOD |
| 9.40 | 2.28 | | | | 19.46 | 1.10 | | | | 4 | Pp | SOD |
| 9.40 | 1.93 | | | | 19.46 | 0.93 | | | | 4 | Pp | SOD |
| 9.40 | 2.36 | | | | 19.46 | 1.14 | | | | 4 | Pp | SOD |
| 9.40 | 2.47 | | Generation 4 | | 19.46 | 1.19 | | Generation 4 | | 4 | Pp | SOD |
| 9.40 | 2.72 | | Average | StDev | 19.46 | 1.32 | | Average | StDev | 4 | Pp | SOD |
| 9.40 | 2.45 | 2.28 | 2.093 | 0.157 | 19.46 | 1.18 | 1.102 | 1.011 | 0.076 | 4 | Pp | SOD |
| | | | | | | | | | | 4 | Np | SOD |
| | | | | | | | | | | 4 | Np | SOD |
| | | | | | | | | | | 4 | Np | SOD |
| | | | | | | | | | | 4 | Np | SOD |
| | | | | | | | | | | 4 | Np | SOD |
| | | | | | | | | | | 4 | Np | SOD |
| | | | | | | | | | | 4 | Np | SOD |
| | | | | | | | | | | 4 | Np | SOD |
| | | | | | | | | | | 4 | Np | SOD |
| | | | | | | | | | | 4 | Np | SOD |
| | | | | | | | | | | 4 | Np | SOD |
| | | | | | | | | | | 4 | Np | SOD |
| | | | | | | | | | | 4 | Np | SOD |
| | | | | | | | | | | 4 | Np | SOD |
| | | | | | | | | | | 4 | Np | SOD |
| | | | | | | | | | | 4 | Np | SOD |
| | | | | | | | | | | 4 | Np | SOD |
| | | | | | | | | | | 4 | Np | SOD |
| | | | | | | | | | | 4 | Np | SOD |
| | | | | | | | | | | 4 | Np | SOD |
| | | | | | | | | | | 4 | Np | SOD |
| | | | | | | | | | | 4 | Np | SOD |
| | | | | | | | | | | 4 | Np | SOD |
| | | | | | | | | | | 4 | Np | SOD |
| | | | | | | | | | | 4 | Np | SOD |
| | | | | | | | | | | 4 | Np | SOD |

# Supplemental Table S1

| | | | | | | | | | | | | | | | | | | | |
|---|---|---|---|---|---|---|---|---|---|---|---|---|---|---|---|---|---|---|---|
| 67 | 4 | SOD | Np | 5 | 4 | 4 | 15 | 0.7 | | | | 16.14 | 0.04 | | | 4 | Np | SOD | |
| 68 | 4 | SOD | Np | 5 | 91 | 5 | 22 | 0.4 | | | | 16.14 | 0.02 | | | 4 | Np | SOD | |
| 69 | 4 | SOD | Np | 5 | 44 | 6 | 5 | 1.0 | | | | 16.14 | 0.06 | | | 4 | Np | SOD | |
| 70 | 4 | SOD | Np | 5 | 88 | 7 | 2 | 15.8 | 5.06 | 9.26 | | 16.14 | 0.98 | 0.314 | 0.573 | 4 | Np | SOD | 9.40 |
| 81 | 4 | SOD | Null | NA | 1 | 1 | 14 | 18.1 | | | | 16.14 | 1.12 | | | 4 | Null | SOD | |
| 82 | 4 | SOD | Null | NA | 63 | 2 | 12 | NA | | | | NA | NA | | | 4 | Null | SOD | |
| 83 | 4 | SOD | Null | NA | 73 | 3 | 16 | 18.7 | | | | 16.14 | 1.16 | | | 4 | Null | SOD | |
| 84 | 4 | SOD | Null | NA | 70 | 4 | 7 | 21.6 | | | | 16.14 | 1.34 | | | 4 | Null | SOD | |
| 85 | 4 | SOD | Null | NA | 100 | 5 | 7 | NA | | | | NA | NA | | | 4 | Null | SOD | |
| 86 | 4 | SOD | Null | NA | 53 | 6 | 6 | NA | | | | NA | NA | | | 4 | Null | SOD | |
| 87 | 4 | SOD | Null | NA | 57 | 7 | 21 | 16.3 | | | | 16.14 | 1.01 | | | 4 | Null | SOD | |
| 88 | 4 | SOD | Null | NA | 43 | 8 | 7 | 17.3 | | | | 16.14 | 1.07 | | | 4 | Null | SOD | |
| 89 | 4 | SOD | Null | NA | 56 | 1 | 24 | 17.0 | | | | 16.14 | 1.05 | | | 4 | Null | SOD | |
| 90 | 4 | SOD | Null | NA | 10 | 2 | 16 | 20.4 | | | | 16.14 | 1.26 | | | 4 | Null | SOD | |
| 91 | 4 | SOD | Null | NA | 62 | 3 | 4 | 24.8 | | | | 16.14 | 1.54 | | | 4 | Null | SOD | |
| 92 | 4 | SOD | Null | NA | 8 | 4 | 1 | 17.2 | | | | 16.14 | 1.07 | | | 4 | Null | SOD | |
| 93 | 4 | SOD | Null | NA | 75 | 5 | 10 | 15.3 | | | | 16.14 | 0.95 | | | 4 | Null | SOD | |
| 94 | 4 | SOD | Null | NA | 22 | 6 | 14 | 23.0 | | | | 16.14 | 1.43 | | | 4 | Null | SOD | |
| 95 | 4 | SOD | Null | NA | 17 | 7 | 24 | 19.9 | | | | 16.14 | 1.23 | | | 4 | Null | SOD | |
| 96 | 4 | SOD | Null | NA | 66 | 8 | 18 | 24.4 | | | | 16.14 | 1.51 | | | 4 | Null | SOD | |
| 97 | 4 | SOD | Null | NA | 25 | 1 | 3 | 21.2 | | | | 16.14 | 1.31 | | | 4 | Null | SOD | |
| 98 | 4 | SOD | Null | NA | 48 | 3 | 10 | 17.9 | | | | 16.14 | 1.11 | | | 4 | Null | SOD | |
| 99 | 4 | SOD | Null | NA | 65 | 5 | 21 | 15.9 | | | Grand Average | 16.14 | 0.99 | | | 4 | Null | SOD | |
| 100 | 4 | SOD | Null | NA | 11 | 7 | 5 | 21.8 | 19.46 | | 16.14 | 16.14 | 1.35 | 1.206 | | 4 | Null | SOD | 19.46 |
| 101 | 4 | ALU | Pp | 1 | 120 | 1 | 15 | NA | | | | NA | NA | | | 4 | Pp | ALU | |
| 102 | 4 | ALU | Pp | 1 | 126 | 2 | 11 | 29.3 | | | | 22.10 | 1.33 | | | 4 | Pp | ALU | |
| 103 | 4 | ALU | Pp | 1 | 198 | 3 | 8 | 29.6 | | | | 22.10 | 1.34 | | | 4 | Pp | ALU | |
| 104 | 4 | ALU | Pp | 1 | 169 | 4 | 17 | 26.9 | | | | 22.10 | 1.22 | | | 4 | Pp | ALU | |
| 105 | 4 | ALU | Pp | 1 | 130 | 5 | 15 | 23.8 | | | | 22.10 | 1.08 | | | 4 | Pp | ALU | |
| 106 | 4 | ALU | Pp | 1 | 195 | 6 | 19 | 32.6 | | | | 22.10 | 1.47 | | | 4 | Pp | ALU | |
| 107 | 4 | ALU | Pp | 1 | 165 | 7 | 6 | 31.0 | | | | 22.10 | 1.40 | | | 4 | Pp | ALU | |
| 108 | 4 | ALU | Pp | 1 | 105 | 8 | 24 | 31.3 | 29.21 | | | 22.10 | 1.42 | 1.322 | | 4 | Pp | ALU | |
| 109 | 4 | ALU | Pp | 2 | 175 | 1 | 9 | 26.4 | | | | 22.10 | 1.19 | | | 4 | Pp | ALU | |
| 110 | 4 | ALU | Pp | 2 | 184 | 2 | 8 | 24.2 | | | | 22.10 | 1.09 | | | 4 | Pp | ALU | |
| 111 | 4 | ALU | Pp | 2 | 147 | 3 | 20 | 26.1 | | | | 22.10 | 1.18 | | | 4 | Pp | ALU | |
| 112 | 4 | ALU | Pp | 2 | 167 | 4 | 23 | 26.0 | | | | 22.10 | 1.18 | | | 4 | Pp | ALU | |
| 113 | 4 | ALU | Pp | 2 | 103 | 5 | 6 | 33.2 | | | | 22.10 | 1.50 | | | 4 | Pp | ALU | |
| 114 | 4 | ALU | Pp | 2 | 172 | 6 | 10 | 24.1 | | | | 22.10 | 1.09 | | | 4 | Pp | ALU | |
| 115 | 4 | ALU | Pp | 2 | 196 | 7 | 14 | 30.0 | | | | 22.10 | 1.36 | | | 4 | Pp | ALU | |
| 116 | 4 | ALU | Pp | 2 | 152 | 8 | 21 | 24.2 | 26.78 | | | 22.10 | 1.09 | 1.211 | | 4 | Pp | ALU | |
| 117 | 4 | ALU | Pp | 3 | 161 | 1 | 17 | 26.7 | | | | 22.10 | 1.21 | | | 4 | Pp | ALU | |
| 118 | 4 | ALU | Pp | 3 | 104 | 2 | 25 | 27.2 | | | | 22.10 | 1.23 | | | 4 | Pp | ALU | |
| 119 | 4 | ALU | Pp | 3 | 157 | 3 | 18 | 26.3 | | | | 22.10 | 1.19 | | | 4 | Pp | ALU | |
| 120 | 4 | ALU | Pp | 3 | 199 | 4 | 12 | 28.6 | | | | 22.10 | 1.29 | | | 4 | Pp | ALU | |
| 121 | 4 | ALU | Pp | 3 | 189 | 5 | 16 | 26.9 | | | | 22.10 | 1.22 | | | 4 | Pp | ALU | |
| 122 | 4 | ALU | Pp | 3 | 148 | 6 | 12 | 32.1 | | | | 22.10 | 1.45 | | | 4 | Pp | ALU | |
| 123 | 4 | ALU | Pp | 3 | 155 | 7 | 9 | 30.4 | | | | 22.10 | 1.38 | | | 4 | Pp | ALU | |
| 124 | 4 | ALU | Pp | 3 | 150 | 8 | 6 | 24.9 | 27.89 | | | 22.10 | 1.13 | 1.262 | | 4 | Pp | ALU | |
| 125 | 4 | ALU | Pp | 4 | 197 | 1 | 8 | 32.7 | | | | 22.10 | 1.48 | | | 4 | Pp | ALU | |
| 126 | 4 | ALU | Pp | 4 | 160 | 2 | 20 | 22.2 | | | | 22.10 | 1.00 | | | 4 | Pp | ALU | |
| 127 | 4 | ALU | Pp | 4 | 176 | 3 | 12 | 20.5 | | | | 22.10 | 0.93 | | | 4 | Pp | ALU | |
| 128 | 4 | ALU | Pp | 4 | 131 | 4 | 25 | 18.1 | | | | 22.10 | 0.82 | | | 4 | Pp | ALU | |
| 129 | 4 | ALU | Pp | 4 | 200 | 5 | 17 | 27.4 | | | | 22.10 | 1.24 | | | 4 | Pp | ALU | |

# Supplemental Table S1

| | | | | | | | | | | | | |
|---|---|---|---|---|---|---|---|---|---|---|---|---|
| | | | | | | | | | | 4 | Np | SOD |
| | | | | | | | | | | 4 | Np | SOD |
| | | | | | | | | | | 4 | Np | SOD |
| | | | | | | | | | | 4 | Np | SOD |
| | | | | | | | | | | 4 | Null | SOD |
| | | | | | | | | | | 4 | Null | SOD |
| | | | | | | | | | | 4 | Null | SOD |
| | | | | | | | | | | 4 | Null | SOD |
| | | | | | | | | | | 4 | Null | SOD |
| | | | | | | | | | | 4 | Null | SOD |
| | | | | | | | | | | 4 | Null | SOD |
| | | | | | | | | | | 4 | Null | SOD |
| | | | | | | | | | | 4 | Null | SOD |
| | | | | | | | | | | 4 | Null | SOD |
| | | | | | | | | | | 4 | Null | SOD |
| | | | | | | | | | | 4 | Null | SOD |
| | | | | | | | | | | 4 | Null | SOD |
| | | | | | | | | | | 4 | Null | SOD |
| | | | | | | | | | | 4 | Null | SOD |
| | | | | | | | | | | 4 | Null | SOD |
| | | | | | | | | | | 4 | Null | SOD |
| | | | | | | | | | | 4 | Null | SOD |
| | | | | | | | | | | 4 | Null | SOD |
| | | | | | | | | | | 4 | Null | SOD |
| NA | NA | | | | NA | NA | | | | 4 | Pp | ALU |
| 14.44 | 2.03 | | | | 23.84 | 1.23 | | | | 4 | Pp | ALU |
| 14.44 | 2.05 | | | | 23.84 | 1.24 | | | | 4 | Pp | ALU |
| 14.44 | 1.86 | | | | 23.84 | 1.13 | | | | 4 | Pp | ALU |
| 14.44 | 1.65 | | | | 23.84 | 1.00 | | | | 4 | Pp | ALU |
| 14.44 | 2.26 | | | | 23.84 | 1.37 | | | | 4 | Pp | ALU |
| 14.44 | 2.15 | | | | 23.84 | 1.30 | | | | 4 | Pp | ALU |
| 14.44 | 2.17 | 2.02 | | | 23.84 | 1.31 | 1.225 | | | 4 | Pp | ALU |
| 14.44 | 1.83 | | | | 23.84 | 1.11 | | | | 4 | Pp | ALU |
| 14.44 | 1.68 | | | | 23.84 | 1.02 | | | | 4 | Pp | ALU |
| 14.44 | 1.81 | | | | 23.84 | 1.09 | | | | 4 | Pp | ALU |
| 14.44 | 1.80 | | | | 23.84 | 1.09 | | | | 4 | Pp | ALU |
| 14.44 | 2.30 | | | | 23.84 | 1.39 | | | | 4 | Pp | ALU |
| 14.44 | 1.67 | | | | 23.84 | 1.01 | | | | 4 | Pp | ALU |
| 14.44 | 2.08 | | | | 23.84 | 1.26 | | | | 4 | Pp | ALU |
| 14.44 | 1.68 | 1.85 | | | 23.84 | 1.02 | 1.123 | | | 4 | Pp | ALU |
| 14.44 | 1.85 | | | | 23.84 | 1.12 | | | | 4 | Pp | ALU |
| 14.44 | 1.88 | | | | 23.84 | 1.14 | | | | 4 | Pp | ALU |
| 14.44 | 1.82 | | | | 23.84 | 1.10 | | | | 4 | Pp | ALU |
| 14.44 | 1.98 | | | | 23.84 | 1.20 | | | | 4 | Pp | ALU |
| 14.44 | 1.86 | | | | 23.84 | 1.13 | | | | 4 | Pp | ALU |
| 14.44 | 2.22 | | | | 23.84 | 1.35 | | | | 4 | Pp | ALU |
| 14.44 | 2.11 | | | | 23.84 | 1.28 | | | | 4 | Pp | ALU |
| 14.44 | 1.72 | 1.93 | | | 23.84 | 1.04 | 1.170 | | | 4 | Pp | ALU |
| 14.44 | 2.26 | | | | 23.84 | 1.37 | | | | 4 | Pp | ALU |
| 14.44 | 1.54 | | | | 23.84 | 0.93 | | | | 4 | Pp | ALU |
| 14.44 | 1.42 | | | | 23.84 | 0.86 | | | | 4 | Pp | ALU |
| 14.44 | 1.25 | | | | 23.84 | 0.76 | | | | 4 | Pp | ALU |
| 14.44 | 1.90 | | | | 23.84 | 1.15 | | | | 4 | Pp | ALU |

# Supplemental Table S1

| | | | | | | | | | | | | | | | | | | | |
|---|---|---|---|---|---|---|---|---|---|---|---|---|---|---|---|---|---|---|---|
| 130 | 4 | ALU | Pp | 4 | 171 | 6 | 7 | 28.6 | | | | 22.10 | 1.29 | | | 4 | Pp | ALU | |
| 131 | 4 | ALU | Pp | 4 | 180 | 7 | 3 | 29.5 | | | | 22.10 | 1.33 | | | 4 | Pp | ALU | |
| 132 | 4 | ALU | Pp | 4 | 115 | 8 | 19 | 29.2 | 26.03 | | | 22.10 | 1.32 | 1.177 | | 4 | Pp | ALU | |
| 133 | 4 | ALU | Pp | 5 | 159 | 1 | 12 | 25.8 | | | | 22.10 | 1.17 | | | 4 | Pp | ALU | |
| 134 | 4 | ALU | Pp | 5 | 121 | 2 | 7 | 27.2 | | | | 22.10 | 1.23 | | | 4 | Pp | ALU | |
| 135 | 4 | ALU | Pp | 5 | 111 | 3 | 5 | 17.0 | | | | 22.10 | 0.77 | | | 4 | Pp | ALU | |
| 136 | 4 | ALU | Pp | 5 | 154 | 4 | 10 | 27.6 | | | | 22.10 | 1.25 | | | 4 | Pp | ALU | |
| 137 | 4 | ALU | Pp | 5 | 108 | 5 | 4 | 27.8 | | | | 22.10 | 1.26 | | | 4 | Pp | ALU | |
| 138 | 4 | ALU | Pp | 5 | 101 | 6 | 18 | 23.0 | | | | 22.10 | 1.04 | | | 4 | Pp | ALU | |
| 139 | 4 | ALU | Pp | 5 | 177 | 7 | 7 | 31.6 | | | | 22.10 | 1.43 | | | 4 | Pp | ALU | |
| 140 | 4 | ALU | Pp | 5 | 168 | 8 | 17 | 27.3 | 25.91 | 27.16 | | 22.10 | 1.24 | 1.172 | 1.229 | 4 | Pp | ALU | 27.11 |
| 141 | 4 | ALU | Np | 1 | 173 | 3 | 9 | 15.2 | | | | 22.10 | 0.69 | | | 4 | Np | ALU | |
| 142 | 4 | ALU | Np | 1 | 149 | 4 | 20 | 7.6 | | | | 22.10 | 0.34 | | | 4 | Np | ALU | |
| 143 | 4 | ALU | Np | 1 | 128 | 5 | 13 | 20.0 | | | | 22.10 | 0.90 | | | 4 | Np | ALU | |
| 144 | 4 | ALU | Np | 1 | 106 | 6 | 25 | 18.4 | | | | 22.10 | 0.83 | | | 4 | Np | ALU | |
| 145 | 4 | ALU | Np | 1 | 143 | 7 | 25 | 16.5 | | | | 22.10 | 0.75 | | | 4 | Np | ALU | |
| 146 | 4 | ALU | Np | 1 | 107 | 8 | 22 | 19.2 | 16.15 | | | 22.10 | 0.87 | 0.731 | | 4 | Np | ALU | |
| 147 | 4 | ALU | Np | 2 | 185 | 1 | 1 | 5.7 | | | | 22.10 | 0.26 | | | 4 | Np | ALU | |
| 148 | 4 | ALU | Np | 2 | 145 | 2 | 3 | 3.5 | | | | 22.10 | 0.16 | | | 4 | Np | ALU | |
| 149 | 4 | ALU | Np | 2 | 129 | 5 | 12 | 11.1 | | | | 22.10 | 0.50 | | | 4 | Np | ALU | |
| 150 | 4 | ALU | Np | 2 | 164 | 6 | 21 | 13.5 | | | | 22.10 | 0.61 | | | 4 | Np | ALU | |
| 151 | 4 | ALU | Np | 2 | 139 | 7 | 4 | 10.7 | | | | 22.10 | 0.48 | | | 4 | Np | ALU | |
| 152 | 4 | ALU | Np | 2 | 190 | 8 | 13 | 8.6 | 8.85 | | | 22.10 | 0.39 | 0.400 | | 4 | Np | ALU | |
| 153 | 4 | ALU | Np | 3 | 109 | 1 | 22 | 12.7 | | | | 22.10 | 0.57 | | | 4 | Np | ALU | |
| 154 | 4 | ALU | Np | 3 | 134 | 2 | 24 | 14.6 | | | | 22.10 | 0.66 | | | 4 | Np | ALU | |
| 155 | 4 | ALU | Np | 3 | 133 | 3 | 3 | 16.7 | | | | 22.10 | 0.76 | | | 4 | Np | ALU | |
| 156 | 4 | ALU | Np | 3 | 188 | 4 | 6 | 1.4 | | | | 22.10 | 0.06 | | | 4 | Np | ALU | |
| 157 | 4 | ALU | Np | 3 | 183 | 7 | 10 | 13.0 | | | | 22.10 | 0.59 | | | 4 | Np | ALU | |
| 158 | 4 | ALU | Np | 3 | 124 | 8 | 25 | 12.4 | 11.80 | | | 22.10 | 0.56 | 0.534 | | 4 | Np | ALU | |
| 159 | 4 | ALU | Np | 4 | 127 | 1 | 19 | 19.5 | | | | 22.10 | 0.88 | | | 4 | Np | ALU | |
| 160 | 4 | ALU | Np | 4 | 114 | 2 | 1 | 19.0 | | | | 22.10 | 0.86 | | | 4 | Np | ALU | |
| 161 | 4 | ALU | Np | 4 | 122 | 3 | 19 | 21.0 | | | | 22.10 | 0.95 | | | 4 | Np | ALU | |
| 162 | 4 | ALU | Np | 4 | 102 | 4 | 13 | 17.4 | | | | 22.10 | 0.79 | | | 4 | Np | ALU | |
| 163 | 4 | ALU | Np | 4 | 141 | 5 | 8 | 20.2 | | | | 22.10 | 0.91 | | | 4 | Np | ALU | |
| 164 | 4 | ALU | Np | 4 | 156 | 6 | 23 | 8.9 | 17.67 | | | 22.10 | 0.40 | 0.799 | | 4 | Np | ALU | |
| 165 | 4 | ALU | Np | 5 | 151 | 2 | 23 | 17.3 | | | | 22.10 | 0.78 | | | 4 | Np | ALU | |
| 166 | 4 | ALU | Np | 5 | 163 | 3 | 11 | 18.3 | | | | 22.10 | 0.83 | | | 4 | Np | ALU | |
| 167 | 4 | ALU | Np | 5 | 140 | 4 | 16 | 17.6 | | | | 22.10 | 0.80 | | | 4 | Np | ALU | |
| 168 | 4 | ALU | Np | 5 | 174 | 6 | 22 | 17.8 | | | | 22.10 | 0.81 | | | 4 | Np | ALU | |
| 169 | 4 | ALU | Np | 5 | 178 | 7 | 1 | 13.0 | | | | 22.10 | 0.59 | | | 4 | Np | ALU | |
| 170 | 4 | ALU | Np | 5 | 144 | 8 | 23 | 22.4 | 17.73 | 14.4 | | 22.10 | 1.01 | 0.802 | 0.653 | 4 | Np | ALU | 14.44 |
| 181 | 4 | ALU | Null | NA | 110 | 1 | 13 | 26.0 | | | | 22.10 | 1.18 | | | 4 | Null | ALU | |
| 182 | 4 | ALU | Null | NA | 119 | 2 | 13 | 22.5 | | | | 22.10 | 1.02 | | | 4 | Null | ALU | |
| 183 | 4 | ALU | Null | NA | 153 | 3 | 2 | 22.6 | | | | 22.10 | 1.02 | | | 4 | Null | ALU | |
| 184 | 4 | ALU | Null | NA | 191 | 4 | 11 | 20.9 | | | | 22.10 | 0.95 | | | 4 | Null | ALU | |
| 185 | 4 | ALU | Null | NA | 136 | 5 | 5 | 22.8 | | | | 22.10 | 1.03 | | | 4 | Null | ALU | |
| 186 | 4 | ALU | Null | NA | 123 | 6 | 20 | 25.5 | | | | 22.10 | 1.15 | | | 4 | Null | ALU | |
| 187 | 4 | ALU | Null | NA | 117 | 7 | 23 | 25.8 | | | | 22.10 | 1.17 | | | 4 | Null | ALU | |
| 188 | 4 | ALU | Null | NA | 116 | 8 | 3 | 27.7 | | | | 22.10 | 1.25 | | | 4 | Null | ALU | |
| 189 | 4 | ALU | Null | NA | 118 | 1 | 4 | 9.0 | | | | 22.10 | 0.41 | | | 4 | Null | ALU | |
| 190 | 4 | ALU | Null | NA | 166 | 2 | 6 | 19.3 | | | | 22.10 | 0.87 | | | 4 | Null | ALU | |
| 191 | 4 | ALU | Null | NA | 142 | 3 | 14 | 23.0 | | | | 22.10 | 1.04 | | | 4 | Null | ALU | |
| 192 | 4 | ALU | Null | NA | 137 | 4 | 3 | 23.0 | | | | 22.10 | 1.04 | | | 4 | Null | ALU | |

## Supplemental Table S1

| | | | | | | | | | | | | |
|---|---|---|---|---|---|---|---|---|---|---|---|---|
| 14.44 | 1.98 | | | | 23.84 | 1.20 | | | | 4 | Pp | ALU |
| 14.44 | 2.04 | | | | 23.84 | 1.24 | | | | 4 | Pp | ALU |
| 14.44 | 2.02 | 1.80 | | | 23.84 | 1.22 | 1.092 | | | 4 | Pp | ALU |
| 14.44 | 1.79 | | | | 23.84 | 1.08 | | | | 4 | Pp | ALU |
| 14.44 | 1.88 | | | | 23.84 | 1.14 | | | | 4 | Pp | ALU |
| 14.44 | 1.18 | | | | 23.84 | 0.71 | | | | 4 | Pp | ALU |
| 14.44 | 1.91 | | | | 23.84 | 1.16 | | | | 4 | Pp | ALU |
| 14.44 | 1.93 | | | | 23.84 | 1.17 | | | | 4 | Pp | ALU |
| 14.44 | 1.59 | | Generation 4 | | 23.84 | 0.96 | | Generation 4 | | 4 | Pp | ALU |
| 14.44 | 2.19 | | Average | StDev | 23.84 | 1.33 | | Average | StDev | 4 | Pp | ALU |
| 14.44 | 1.89 | 1.79 | 1.881 | 0.096 | 23.84 | 1.15 | 1.087 | 1.139 | 0.058 | 4 | Pp | ALU |
| | | | | | | | | | | 4 | Np | ALU |
| | | | | | | | | | | 4 | Np | ALU |
| | | | | | | | | | | 4 | Np | ALU |
| | | | | | | | | | | 4 | Np | ALU |
| | | | | | | | | | | 4 | Np | ALU |
| | | | | | | | | | | 4 | Np | ALU |
| | | | | | | | | | | 4 | Np | ALU |
| | | | | | | | | | | 4 | Np | ALU |
| | | | | | | | | | | 4 | Np | ALU |
| | | | | | | | | | | 4 | Np | ALU |
| | | | | | | | | | | 4 | Np | ALU |
| | | | | | | | | | | 4 | Np | ALU |
| | | | | | | | | | | 4 | Np | ALU |
| | | | | | | | | | | 4 | Np | ALU |
| | | | | | | | | | | 4 | Np | ALU |
| | | | | | | | | | | 4 | Np | ALU |
| | | | | | | | | | | 4 | Np | ALU |
| | | | | | | | | | | 4 | Np | ALU |
| | | | | | | | | | | 4 | Np | ALU |
| | | | | | | | | | | 4 | Np | ALU |
| | | | | | | | | | | 4 | Np | ALU |
| | | | | | | | | | | 4 | Np | ALU |
| | | | | | | | | | | 4 | Np | ALU |
| | | | | | | | | | | 4 | Np | ALU |
| | | | | | | | | | | 4 | Np | ALU |
| | | | | | | | | | | 4 | Np | ALU |
| | | | | | | | | | | 4 | Np | ALU |
| | | | | | | | | | | 4 | Np | ALU |
| | | | | | | | | | | 4 | Np | ALU |
| | | | | | | | | | | 4 | Np | ALU |
| | | | | | | | | | | 4 | Null | ALU |
| | | | | | | | | | | 4 | Null | ALU |
| | | | | | | | | | | 4 | Null | ALU |
| | | | | | | | | | | 4 | Null | ALU |
| | | | | | | | | | | 4 | Null | ALU |
| | | | | | | | | | | 4 | Null | ALU |
| | | | | | | | | | | 4 | Null | ALU |
| | | | | | | | | | | 4 | Null | ALU |
| | | | | | | | | | | 4 | Null | ALU |
| | | | | | | | | | | 4 | Null | ALU |
| | | | | | | | | | | 4 | Null | ALU |
| | | | | | | | | | | 4 | Null | ALU |

## Supplemental Table S1

| | | | | | | | | | | | | | | | | | | | |
|---|---|---|---|---|---|---|---|---|---|---|---|---|---|---|---|---|---|---|---|
| 193 | 4 | ALU | Null | NA | 146 | 5 | 18 | 25.0 | | | | 22.10 | 1.13 | | | 4 | Null | ALU | |
| 194 | 4 | ALU | Null | NA | 181 | 6 | 15 | 24.9 | | | | 22.10 | 1.13 | | | 4 | Null | ALU | |
| 195 | 4 | ALU | Null | NA | 193 | 7 | 22 | 25.3 | | | | 22.10 | 1.14 | | | 4 | Null | ALU | |
| 196 | 4 | ALU | Null | NA | 194 | 8 | 20 | 25.6 | | | | 22.10 | 1.16 | | | 4 | Null | ALU | |
| 197 | 4 | ALU | Null | NA | 170 | 2 | 22 | 28.2 | | | | 22.10 | 1.28 | | | 4 | Null | ALU | |
| 198 | 4 | ALU | Null | NA | 162 | 4 | 18 | 24.4 | | | | 22.10 | 1.10 | | | 4 | Null | ALU | |
| 199 | 4 | ALU | Null | NA | 138 | 6 | 9 | 25.8 | | | Grand Average | 22.10 | 1.17 | | | 4 | Null | ALU | |
| 200 | 4 | ALU | Null | NA | 132 | 8 | 1 | 29.5 | 23.84 | | 22.1 | 22.10 | 1.33 | 1.079 | | 4 | Null | ALU | 23.84 |
| 1 | 5 | SOD | Pp | 1 | 21 | 1 | 13 | 35.5 | | | | 31.72 | 1.12 | | | 5 | Pp | SOD | |
| 2 | 5 | SOD | Pp | 1 | 96 | 2 | 3 | 36.1 | | | | 31.72 | 1.14 | | | 5 | Pp | SOD | |
| 3 | 5 | SOD | Pp | 1 | 57 | 3 | 23 | 40.1 | | | | 31.72 | 1.26 | | | 5 | Pp | SOD | |
| 4 | 5 | SOD | Pp | 1 | 58 | 4 | 6 | 27.8 | | | | 31.72 | 0.88 | | | 5 | Pp | SOD | |
| 5 | 5 | SOD | Pp | 1 | 48 | 5 | 11 | 37.2 | | | | 31.72 | 1.17 | | | 5 | Pp | SOD | |
| 6 | 5 | SOD | Pp | 1 | 69 | 6 | 19 | 30.0 | | | | 31.72 | 0.95 | | | 5 | Pp | SOD | |
| 7 | 5 | SOD | Pp | 1 | 85 | 7 | 10 | 27.7 | | | | 31.72 | 0.87 | | | 5 | Pp | SOD | |
| 8 | 5 | SOD | Pp | 1 | 79 | 8 | 19 | 35.4 | 33.73 | | | 31.72 | 1.12 | 1.063 | | 5 | Pp | SOD | |
| 9 | 5 | SOD | Pp | 2 | 50 | 1 | 14 | 33.8 | | | | 31.72 | 1.07 | | | 5 | Pp | SOD | |
| 10 | 5 | SOD | Pp | 2 | 12 | 2 | 2 | 37.0 | | | | 31.72 | 1.17 | | | 5 | Pp | SOD | |
| 11 | 5 | SOD | Pp | 2 | 60 | 3 | 20 | 37.0 | | | | 31.72 | 1.17 | | | 5 | Pp | SOD | |
| 12 | 5 | SOD | Pp | 2 | 52 | 4 | 22 | 30.9 | | | | 31.72 | 0.97 | | | 5 | Pp | SOD | |
| 13 | 5 | SOD | Pp | 2 | 87 | 5 | 23 | 47.4 | | | | 31.72 | 1.49 | | | 5 | Pp | SOD | |
| 14 | 5 | SOD | Pp | 2 | 8 | 6 | 12 | 37.6 | | | | 31.72 | 1.19 | | | 5 | Pp | SOD | |
| 15 | 5 | SOD | Pp | 2 | 95 | 7 | 8 | 39.9 | | | | 31.72 | 1.26 | | | 5 | Pp | SOD | |
| 16 | 5 | SOD | Pp | 2 | 77 | 8 | 14 | NA | 37.66 | | | NA | NA | 1.187 | | 5 | Pp | SOD | |
| 17 | 5 | SOD | Pp | 3 | 39 | 1 | 18 | 36.4 | | | | 31.72 | 1.15 | | | 5 | Pp | SOD | |
| 18 | 5 | SOD | Pp | 3 | 92 | 2 | 16 | 36.6 | | | | 31.72 | 1.15 | | | 5 | Pp | SOD | |
| 19 | 5 | SOD | Pp | 3 | 78 | 3 | 17 | 35.9 | | | | 31.72 | 1.13 | | | 5 | Pp | SOD | |
| 20 | 5 | SOD | Pp | 3 | 9 | 4 | 1 | 28.4 | | | | 31.72 | 0.90 | | | 5 | Pp | SOD | |
| 21 | 5 | SOD | Pp | 3 | 63 | 5 | 14 | 42.0 | | | | 31.72 | 1.32 | | | 5 | Pp | SOD | |
| 22 | 5 | SOD | Pp | 3 | 15 | 6 | 23 | 28.7 | | | | 31.72 | 0.90 | | | 5 | Pp | SOD | |
| 23 | 5 | SOD | Pp | 3 | 18 | 7 | 2 | 36.2 | | | | 31.72 | 1.14 | | | 5 | Pp | SOD | |
| 24 | 5 | SOD | Pp | 3 | 13 | 8 | 11 | 34.5 | 34.84 | | | 31.72 | 1.09 | 1.098 | | 5 | Pp | SOD | |
| 25 | 5 | SOD | Pp | 4 | 4 | 1 | 7 | 40.7 | | | | 31.72 | 1.28 | | | 5 | Pp | SOD | |
| 26 | 5 | SOD | Pp | 4 | 36 | 2 | 7 | 30.3 | | | | 31.72 | 0.96 | | | 5 | Pp | SOD | |
| 27 | 5 | SOD | Pp | 4 | 49 | 3 | 11 | 28.0 | | | | 31.72 | 0.88 | | | 5 | Pp | SOD | |
| 28 | 5 | SOD | Pp | 4 | 6 | 4 | 9 | 31.9 | | | | 31.72 | 1.01 | | | 5 | Pp | SOD | |
| 29 | 5 | SOD | Pp | 4 | 68 | 5 | 17 | 43.6 | | | | 31.72 | 1.37 | | | 5 | Pp | SOD | |
| 30 | 5 | SOD | Pp | 4 | 43 | 6 | 22 | 34.4 | | | | 31.72 | 1.08 | | | 5 | Pp | SOD | |
| 31 | 5 | SOD | Pp | 4 | 83 | 7 | 7 | 34.7 | | | | 31.72 | 1.09 | | | 5 | Pp | SOD | |
| 32 | 5 | SOD | Pp | 4 | 65 | 8 | 8 | NA | 34.80 | | | NA | NA | 1.097 | | 5 | Pp | SOD | |
| 33 | 5 | SOD | Pp | 5 | 70 | 1 | 9 | 34.8 | | | | 31.72 | 1.10 | | | 5 | Pp | SOD | |
| 34 | 5 | SOD | Pp | 5 | 73 | 2 | 10 | 38.4 | | | | 31.72 | 1.21 | | | 5 | Pp | SOD | |
| 35 | 5 | SOD | Pp | 5 | 93 | 3 | 5 | 28.3 | | | | 31.72 | 0.89 | | | 5 | Pp | SOD | |
| 36 | 5 | SOD | Pp | 5 | 82 | 4 | 2 | 29.7 | | | | 31.72 | 0.94 | | | 5 | Pp | SOD | |
| 37 | 5 | SOD | Pp | 5 | 38 | 5 | 7 | 43.3 | | | | 31.72 | 1.36 | | | 5 | Pp | SOD | |
| 38 | 5 | SOD | Pp | 5 | 46 | 6 | 11 | 33.2 | | | | 31.72 | 1.05 | | | 5 | Pp | SOD | |
| 39 | 5 | SOD | Pp | 5 | 67 | 7 | 16 | 36.5 | | | | 31.72 | 1.15 | | | 5 | Pp | SOD | |
| 40 | 5 | SOD | Pp | 5 | 45 | 8 | 6 | 33.1 | 34.66 | 35.14 | | 31.72 | 1.04 | 1.093 | 1.108 | 5 | Pp | SOD | 35.08 |
| 41 | 5 | SOD | Np | 1 | 99 | 1 | 24 | 31.7 | | | | 31.72 | 1.00 | | | 5 | Np | SOD | |
| 42 | 5 | SOD | Np | 1 | 37 | 2 | 22 | 0.9 | | | | 31.72 | 0.03 | | | 5 | Np | SOD | |
| 43 | 5 | SOD | Np | 1 | 7 | 5 | 21 | 34.5 | | | | 31.72 | 1.09 | | | 5 | Np | SOD | |
| 44 | 5 | SOD | Np | 1 | 55 | 6 | 2 | 36.7 | 25.95 | | | 31.72 | 1.16 | 0.818 | | 5 | Np | SOD | |
| 45 | 5 | SOD | Np | 2 | 47 | 3 | 14 | 23.6 | | | | 31.72 | 0.74 | | | 5 | Np | SOD | |

## Supplemental Table S1

| | | | | | | | | | | | | |
|---|---|---|---|---|---|---|---|---|---|---|---|---|
| | | | | | | | | | | 4 | Null | ALU |
| | | | | | | | | | | 4 | Null | ALU |
| | | | | | | | | | | 4 | Null | ALU |
| | | | | | | | | | | 4 | Null | ALU |
| | | | | | | | | | | 4 | Null | ALU |
| | | | | | | | | | | 4 | Null | ALU |
| | | | | | | | | | | 4 | Null | ALU |
| | | | | | | | | | | 4 | Null | ALU |
| 24.24 | 1.46 | | | | 32.28 | 1.10 | | | | 5 | Pp | SOD |
| 24.24 | 1.49 | | | | 32.28 | 1.12 | | | | 5 | Pp | SOD |
| 24.24 | 1.65 | | | | 32.28 | 1.24 | | | | 5 | Pp | SOD |
| 24.24 | 1.15 | | | | 32.28 | 0.86 | | | | 5 | Pp | SOD |
| 24.24 | 1.53 | | | | 32.28 | 1.15 | | | | 5 | Pp | SOD |
| 24.24 | 1.24 | | | | 32.28 | 0.93 | | | | 5 | Pp | SOD |
| 24.24 | 1.14 | | | | 32.28 | 0.86 | | | | 5 | Pp | SOD |
| 24.24 | 1.46 | 1.39 | | | 32.28 | 1.10 | 1.045 | | | 5 | Pp | SOD |
| 24.24 | 1.39 | | | | 32.28 | 1.05 | | | | 5 | Pp | SOD |
| 24.24 | 1.53 | | | | 32.28 | 1.15 | | | | 5 | Pp | SOD |
| 24.24 | 1.53 | | | | 32.28 | 1.15 | | | | 5 | Pp | SOD |
| 24.24 | 1.27 | | | | 32.28 | 0.96 | | | | 5 | Pp | SOD |
| 24.24 | 1.96 | | | | 32.28 | 1.47 | | | | 5 | Pp | SOD |
| 24.24 | 1.55 | | | | 32.28 | 1.16 | | | | 5 | Pp | SOD |
| 24.24 | 1.65 | | | | 32.28 | 1.24 | | | | 5 | Pp | SOD |
| NA | NA | 1.55 | | | NA | NA | 1.167 | | | 5 | Pp | SOD |
| 24.24 | 1.50 | | | | 32.28 | 1.13 | | | | 5 | Pp | SOD |
| 24.24 | 1.51 | | | | 32.28 | 1.13 | | | | 5 | Pp | SOD |
| 24.24 | 1.48 | | | | 32.28 | 1.11 | | | | 5 | Pp | SOD |
| 24.24 | 1.17 | | | | 32.28 | 0.88 | | | | 5 | Pp | SOD |
| 24.24 | 1.73 | | | | 32.28 | 1.30 | | | | 5 | Pp | SOD |
| 24.24 | 1.18 | | | | 32.28 | 0.89 | | | | 5 | Pp | SOD |
| 24.24 | 1.49 | | | | 32.28 | 1.12 | | | | 5 | Pp | SOD |
| 24.24 | 1.42 | 1.44 | | | 32.28 | 1.07 | 1.079 | | | 5 | Pp | SOD |
| 24.24 | 1.68 | | | | 32.28 | 1.26 | | | | 5 | Pp | SOD |
| 24.24 | 1.25 | | | | 32.28 | 0.94 | | | | 5 | Pp | SOD |
| 24.24 | 1.16 | | | | 32.28 | 0.87 | | | | 5 | Pp | SOD |
| 24.24 | 1.32 | | | | 32.28 | 0.99 | | | | 5 | Pp | SOD |
| 24.24 | 1.80 | | | | 32.28 | 1.35 | | | | 5 | Pp | SOD |
| 24.24 | 1.42 | | | | 32.28 | 1.07 | | | | 5 | Pp | SOD |
| 24.24 | 1.43 | | | | 32.28 | 1.07 | | | | 5 | Pp | SOD |
| NA | NA | 1.44 | | | NA | NA | 1.078 | | | 5 | Pp | SOD |
| 24.24 | 1.44 | | | | 32.28 | 1.08 | | | | 5 | Pp | SOD |
| 24.24 | 1.58 | | | | 32.28 | 1.19 | | | | 5 | Pp | SOD |
| 24.24 | 1.17 | | | | 32.28 | 0.88 | | | | 5 | Pp | SOD |
| 24.24 | 1.23 | | | | 32.28 | 0.92 | | | | 5 | Pp | SOD |
| 24.24 | 1.79 | | | | 32.28 | 1.34 | | | | 5 | Pp | SOD |
| 24.24 | 1.37 | | Generation 5 | | 32.28 | 1.03 | | Generation 5 | | 5 | Pp | SOD |
| 24.24 | 1.51 | | Average | StDev | 32.28 | 1.13 | | Average | StDev | 5 | Pp | SOD |
| 24.24 | 1.37 | 1.43 | 1.449 | 0.061 | 32.28 | 1.03 | 1.074 | 1.088 | 0.046 | 5 | Pp | SOD |
| | | | | | | | | | | 5 | Np | SOD |
| | | | | | | | | | | 5 | Np | SOD |
| | | | | | | | | | | 5 | Np | SOD |
| | | | | | | | | | | 5 | Np | SOD |
| | | | | | | | | | | 5 | Np | SOD |

# Supplemental Table S1

| | | | | | | | | | | | | | | | | | | | |
|---|---|---|---|---|---|---|---|---|---|---|---|---|---|---|---|---|---|---|---|
| 46 | 5 | SOD | Np | 2 | 22 | 4 | 12 | 39.1 | | | | 31.72 | 1.23 | | | 5 | Np | SOD | |
| 47 | 5 | SOD | Np | 2 | 75 | 7 | 21 | 38.6 | | | | 31.72 | 1.22 | | | 5 | Np | SOD | |
| 48 | 5 | SOD | Np | 2 | 44 | 8 | 4 | 1.3 | 25.65 | | | 31.72 | 0.04 | 0.809 | | 5 | Np | SOD | |
| 49 | 5 | SOD | Np | 3 | 35 | 1 | 10 | 19.9 | | | | 31.72 | 0.63 | | | 5 | Np | SOD | |
| 50 | 5 | SOD | Np | 3 | 33 | 2 | 1 | 5.3 | | | | 31.72 | 0.17 | | | 5 | Np | SOD | |
| 51 | 5 | SOD | Np | 3 | 3 | 5 | 6 | NA | | | | NA | NA | | | 5 | Np | SOD | |
| 52 | 5 | SOD | Np | 3 | 62 | 6 | 21 | 20.8 | 15.33 | | | 31.72 | 0.66 | 0.483 | | 5 | Np | SOD | |
| 53 | 5 | SOD | Np | 4 | 31 | 3 | 13 | 0.6 | | | | 31.72 | 0.02 | | | 5 | Np | SOD | |
| 54 | 5 | SOD | Np | 4 | 40 | 4 | 5 | 36.2 | | | | 31.72 | 1.14 | | | 5 | Np | SOD | |
| 55 | 5 | SOD | Np | 4 | 94 | 7 | 5 | 21.5 | | | | 31.72 | 0.68 | | | 5 | Np | SOD | |
| 56 | 5 | SOD | Np | 4 | 41 | 8 | 9 | 0.9 | 14.80 | | | 31.72 | 0.03 | 0.467 | | 5 | Np | SOD | |
| 57 | 5 | SOD | Np | 5 | 23 | 1 | 23 | 41.5 | | | | 31.72 | 1.31 | | | 5 | Np | SOD | |
| 58 | 5 | SOD | Np | 5 | 56 | 3 | 16 | 33.1 | | | | 31.72 | 1.04 | | | 5 | Np | SOD | |
| 59 | 5 | SOD | Np | 5 | 81 | 5 | 1 | 30.5 | | | | 31.72 | 0.96 | | | 5 | Np | SOD | |
| 60 | 5 | SOD | Np | 5 | 59 | 7 | 13 | 43.9 | 37.25 | 23.80 | | 31.72 | 1.38 | 1.174 | 0.750 | 5 | Np | SOD | 24.24 |
| 71 | 5 | SOD | Null | NA | 89 | 1 | 15 | 38.1 | | | | 31.72 | 1.20 | | | 5 | Null | SOD | |
| 72 | 5 | SOD | Null | NA | 14 | 2 | 5 | 27.0 | | | | 31.72 | 0.85 | | | 5 | Null | SOD | |
| 73 | 5 | SOD | Null | NA | 16 | 3 | 19 | 28.0 | | | | 31.72 | 0.88 | | | 5 | Null | SOD | |
| 74 | 5 | SOD | Null | NA | 5 | 4 | 23 | 30.6 | | | | 31.72 | 0.96 | | | 5 | Null | SOD | |
| 75 | 5 | SOD | Null | NA | 90 | 5 | 22 | 32.4 | | | | 31.72 | 1.02 | | | 5 | Null | SOD | |
| 76 | 5 | SOD | Null | NA | 2 | 6 | 7 | 33.2 | | | | 31.72 | 1.05 | | | 5 | Null | SOD | |
| 77 | 5 | SOD | Null | NA | 53 | 7 | 14 | 31.2 | | | | 31.72 | 0.98 | | | 5 | Null | SOD | |
| 78 | 5 | SOD | Null | NA | 27 | 8 | 21 | 19.5 | | | | 31.72 | 0.61 | | | 5 | Null | SOD | |
| 79 | 5 | SOD | Null | NA | 76 | 1 | 20 | 38.7 | | | | 31.72 | 1.22 | | | 5 | Null | SOD | |
| 80 | 5 | SOD | Null | NA | 91 | 2 | 21 | 32.1 | | | | 31.72 | 1.01 | | | 5 | Null | SOD | |
| 81 | 5 | SOD | Null | NA | 17 | 3 | 8 | 29.6 | | | | 31.72 | 0.93 | | | 5 | Null | SOD | |
| 82 | 5 | SOD | Null | NA | 72 | 4 | 18 | 35.2 | | | | 31.72 | 1.11 | | | 5 | Null | SOD | |
| 83 | 5 | SOD | Null | NA | 98 | 5 | 19 | 30.9 | | | | 31.72 | 0.97 | | | 5 | Null | SOD | |
| 84 | 5 | SOD | Null | NA | 11 | 6 | 20 | 31.3 | | | | 31.72 | 0.99 | | | 5 | Null | SOD | |
| 85 | 5 | SOD | Null | NA | 42 | 7 | 23 | NA | | | | NA | NA | | | 5 | Null | SOD | |
| 86 | 5 | SOD | Null | NA | 28 | 8 | 5 | 17.1 | | | | 31.72 | 0.54 | | | 5 | Null | SOD | |
| 87 | 5 | SOD | Null | NA | 10 | 1 | 3 | 37.1 | | | | 31.72 | 1.17 | | | 5 | Null | SOD | |
| 88 | 5 | SOD | Null | NA | 51 | 2 | 15 | 33.5 | | | | 31.72 | 1.06 | | | 5 | Null | SOD | |
| 89 | 5 | SOD | Null | NA | 25 | 3 | 2 | 33.5 | | | | 31.72 | 1.06 | | | 5 | Null | SOD | |
| 90 | 5 | SOD | Null | NA | 19 | 4 | 15 | NA | | | | NA | NA | | | 5 | Null | SOD | |
| 91 | 5 | SOD | Null | NA | 100 | 5 | 15 | 34.6 | | | | 31.72 | 1.09 | | | 5 | Null | SOD | |
| 92 | 5 | SOD | Null | NA | 64 | 6 | 14 | NA | | | | NA | NA | | | 5 | Null | SOD | |
| 93 | 5 | SOD | Null | NA | 54 | 7 | 25 | NA | | | | NA | NA | | | 5 | Null | SOD | |
| 94 | 5 | SOD | Null | NA | 61 | 8 | 7 | 40.5 | | | | 31.72 | 1.28 | | | 5 | Null | SOD | |
| 95 | 5 | SOD | Null | NA | 74 | 1 | 25 | 34.7 | | | | 31.72 | 1.09 | | | 5 | Null | SOD | |
| 96 | 5 | SOD | Null | NA | 30 | 3 | 6 | 27.0 | | | | 31.72 | 0.85 | | | 5 | Null | SOD | |
| 97 | 5 | SOD | Null | NA | 24 | 4 | 17 | 36.2 | | | | 31.72 | 1.14 | | | 5 | Null | SOD | |
| 98 | 5 | SOD | Null | NA | 66 | 5 | 8 | 35.5 | | | | 31.72 | 1.12 | | | 5 | Null | SOD | |
| 99 | 5 | SOD | Null | NA | 71 | 6 | 9 | 38.3 | | | Grand Average | 31.72 | 1.21 | | | 5 | Null | SOD | |
| 100 | 5 | SOD | Null | NA | 26 | 8 | 18 | 33.5 | 32.28 | | 31.72 | 31.72 | 1.06 | 1.018 | | 5 | Null | SOD | 32.28 |
| 101 | 5 | ALU | Pp | 1 | 165 | 1 | 22 | 57.8 | | | | 38.27 | 1.51 | | | 5 | Pp | ALU | |
| 102 | 5 | ALU | Pp | 1 | 136 | 2 | 12 | 42.4 | | | | 38.27 | 1.11 | | | 5 | Pp | ALU | |
| 103 | 5 | ALU | Pp | 1 | 131 | 3 | 22 | 50.5 | | | | 38.27 | 1.32 | | | 5 | Pp | ALU | |
| 104 | 5 | ALU | Pp | 1 | 103 | 4 | 4 | 48.2 | | | | 38.27 | 1.26 | | | 5 | Pp | ALU | |
| 105 | 5 | ALU | Pp | 1 | 104 | 5 | 9 | 50.8 | | | | 38.27 | 1.33 | | | 5 | Pp | ALU | |
| 106 | 5 | ALU | Pp | 1 | 132 | 6 | 8 | 49.4 | | | | 38.27 | 1.29 | | | 5 | Pp | ALU | |
| 107 | 5 | ALU | Pp | 1 | 195 | 7 | 19 | 36.3 | | | | 38.27 | 0.95 | | | 5 | Pp | ALU | |
| 108 | 5 | ALU | Pp | 1 | 145 | 8 | 12 | 53.5 | 48.61 | | | 38.27 | 1.40 | 1.270 | | 5 | Pp | ALU | |

## Supplemental Table S1

| | | | | | | | | | | | | |
|---|---|---|---|---|---|---|---|---|---|---|---|---|
| | | | | | | | | | | 5 | Np | SOD |
| | | | | | | | | | | 5 | Np | SOD |
| | | | | | | | | | | 5 | Np | SOD |
| | | | | | | | | | | 5 | Np | SOD |
| | | | | | | | | | | 5 | Np | SOD |
| | | | | | | | | | | 5 | Np | SOD |
| | | | | | | | | | | 5 | Np | SOD |
| | | | | | | | | | | 5 | Np | SOD |
| | | | | | | | | | | 5 | Np | SOD |
| | | | | | | | | | | 5 | Np | SOD |
| | | | | | | | | | | 5 | Np | SOD |
| | | | | | | | | | | 5 | Np | SOD |
| | | | | | | | | | | 5 | Np | SOD |
| | | | | | | | | | | 5 | Np | SOD |
| | | | | | | | | | | 5 | Np | SOD |
| | | | | | | | | | | 5 | Null | SOD |
| | | | | | | | | | | 5 | Null | SOD |
| | | | | | | | | | | 5 | Null | SOD |
| | | | | | | | | | | 5 | Null | SOD |
| | | | | | | | | | | 5 | Null | SOD |
| | | | | | | | | | | 5 | Null | SOD |
| | | | | | | | | | | 5 | Null | SOD |
| | | | | | | | | | | 5 | Null | SOD |
| | | | | | | | | | | 5 | Null | SOD |
| | | | | | | | | | | 5 | Null | SOD |
| | | | | | | | | | | 5 | Null | SOD |
| | | | | | | | | | | 5 | Null | SOD |
| | | | | | | | | | | 5 | Null | SOD |
| | | | | | | | | | | 5 | Null | SOD |
| | | | | | | | | | | 5 | Null | SOD |
| | | | | | | | | | | 5 | Null | SOD |
| | | | | | | | | | | 5 | Null | SOD |
| | | | | | | | | | | 5 | Null | SOD |
| | | | | | | | | | | 5 | Null | SOD |
| | | | | | | | | | | 5 | Null | SOD |
| | | | | | | | | | | 5 | Null | SOD |
| | | | | | | | | | | 5 | Null | SOD |
| | | | | | | | | | | 5 | Null | SOD |
| | | | | | | | | | | 5 | Null | SOD |
| | | | | | | | | | | 5 | Null | SOD |
| | | | | | | | | | | 5 | Null | SOD |
| | | | | | | | | | | 5 | Null | SOD |
| | | | | | | | | | | 5 | Null | SOD |
| | | | | | | | | | | 5 | Null | SOD |
| | | | | | | | | | | 5 | Null | SOD |
| 20.84 | 2.77 | | | | 41.39 | 1.40 | | | | 5 | Pp | ALU |
| 20.84 | 2.03 | | | | 41.39 | 1.02 | | | | 5 | Pp | ALU |
| 20.84 | 2.42 | | | | 41.39 | 1.22 | | | | 5 | Pp | ALU |
| 20.84 | 2.31 | | | | 41.39 | 1.16 | | | | 5 | Pp | ALU |
| 20.84 | 2.44 | | | | 41.39 | 1.23 | | | | 5 | Pp | ALU |
| 20.84 | 2.37 | | | | 41.39 | 1.19 | | | | 5 | Pp | ALU |
| 20.84 | 1.74 | | | | 41.39 | 0.88 | | | | 5 | Pp | ALU |
| 20.84 | 2.57 | 2.33 | | | 41.39 | 1.29 | 1.175 | | | 5 | Pp | ALU |

# Supplemental Table S1

| | | | | | | | | | | | | | | | | | | | |
|---|---|---|---|---|---|---|---|---|---|---|---|---|---|---|---|---|---|---|---|
| 109 | 5 | ALU | Pp | 2 | 130 | 1 | 19 | 38.2 | | | | 38.27 | 1.00 | | | 5 | Pp | ALU | |
| 110 | 5 | ALU | Pp | 2 | 169 | 2 | 13 | 49.7 | | | | 38.27 | 1.30 | | | 5 | Pp | ALU | |
| 111 | 5 | ALU | Pp | 2 | 178 | 3 | 21 | 36.2 | | | | 38.27 | 0.95 | | | 5 | Pp | ALU | |
| 112 | 5 | ALU | Pp | 2 | 150 | 4 | 20 | 38.9 | | | | 38.27 | 1.02 | | | 5 | Pp | ALU | |
| 113 | 5 | ALU | Pp | 2 | 120 | 5 | 24 | NA | | | | NA | NA | | | 5 | Pp | ALU | |
| 114 | 5 | ALU | Pp | 2 | 113 | 6 | 18 | 36.9 | | | | 38.27 | 0.96 | | | 5 | Pp | ALU | |
| 115 | 5 | ALU | Pp | 2 | 164 | 7 | 18 | 42.3 | | | | 38.27 | 1.11 | | | 5 | Pp | ALU | |
| 116 | 5 | ALU | Pp | 2 | 134 | 8 | 25 | 51.3 | 41.93 | | | 38.27 | 1.34 | 1.096 | | 5 | Pp | ALU | |
| 117 | 5 | ALU | Pp | 3 | 199 | 1 | 4 | 49.6 | | | | 38.27 | 1.30 | | | 5 | Pp | ALU | |
| 118 | 5 | ALU | Pp | 3 | 156 | 2 | 25 | 36.1 | | | | 38.27 | 0.94 | | | 5 | Pp | ALU | |
| 119 | 5 | ALU | Pp | 3 | 175 | 3 | 7 | 43.3 | | | | 38.27 | 1.13 | | | 5 | Pp | ALU | |
| 120 | 5 | ALU | Pp | 3 | 196 | 4 | 19 | 53.4 | | | | 38.27 | 1.40 | | | 5 | Pp | ALU | |
| 121 | 5 | ALU | Pp | 3 | 144 | 5 | 16 | 40.0 | | | | 38.27 | 1.05 | | | 5 | Pp | ALU | |
| 122 | 5 | ALU | Pp | 3 | 149 | 6 | 3 | 36.6 | | | | 38.27 | 0.96 | | | 5 | Pp | ALU | |
| 123 | 5 | ALU | Pp | 3 | 186 | 7 | 3 | 42.4 | | | | 38.27 | 1.11 | | | 5 | Pp | ALU | |
| 124 | 5 | ALU | Pp | 3 | 121 | 8 | 3 | 39.2 | 42.58 | | | 38.27 | 1.02 | 1.113 | | 5 | Pp | ALU | |
| 125 | 5 | ALU | Pp | 4 | 187 | 1 | 17 | 64.6 | | | | 38.27 | 1.69 | | | 5 | Pp | ALU | |
| 126 | 5 | ALU | Pp | 4 | 176 | 2 | 14 | 46.5 | | | | 38.27 | 1.22 | | | 5 | Pp | ALU | |
| 127 | 5 | ALU | Pp | 4 | 133 | 3 | 25 | 38.5 | | | | 38.27 | 1.01 | | | 5 | Pp | ALU | |
| 128 | 5 | ALU | Pp | 4 | 114 | 4 | 21 | 44.7 | | | | 38.27 | 1.17 | | | 5 | Pp | ALU | |
| 129 | 5 | ALU | Pp | 4 | 142 | 5 | 3 | 47.5 | | | | 38.27 | 1.24 | | | 5 | Pp | ALU | |
| 130 | 5 | ALU | Pp | 4 | 127 | 6 | 5 | 43.4 | | | | 38.27 | 1.13 | | | 5 | Pp | ALU | |
| 131 | 5 | ALU | Pp | 4 | 168 | 7 | 1 | 12.5 | | | | 38.27 | 0.33 | | | 5 | Pp | ALU | |
| 132 | 5 | ALU | Pp | 4 | 111 | 8 | 17 | 47.8 | 43.19 | | | 38.27 | 1.25 | 1.129 | | 5 | Pp | ALU | |
| 133 | 5 | ALU | Pp | 5 | 129 | 1 | 5 | 49.8 | | | | 38.27 | 1.30 | | | 5 | Pp | ALU | |
| 134 | 5 | ALU | Pp | 5 | 146 | 2 | 19 | 48.4 | | | | 38.27 | 1.26 | | | 5 | Pp | ALU | |
| 135 | 5 | ALU | Pp | 5 | 137 | 3 | 4 | 42.8 | | | | 38.27 | 1.12 | | | 5 | Pp | ALU | |
| 136 | 5 | ALU | Pp | 5 | 135 | 4 | 8 | 38.3 | | | | 38.27 | 1.00 | | | 5 | Pp | ALU | |
| 137 | 5 | ALU | Pp | 5 | 107 | 5 | 4 | 52.5 | | | | 38.27 | 1.37 | | | 5 | Pp | ALU | |
| 138 | 5 | ALU | Pp | 5 | 192 | 6 | 17 | 41.3 | | | | 38.27 | 1.08 | | | 5 | Pp | ALU | |
| 139 | 5 | ALU | Pp | 5 | 172 | 7 | 9 | 41.6 | | | | 38.27 | 1.09 | | | 5 | Pp | ALU | |
| 140 | 5 | ALU | Pp | 5 | 154 | 8 | 2 | 35.5 | 43.78 | 44.02 | | 38.27 | 0.93 | 1.144 | 1.150 | 5 | Pp | ALU | 44.07 |
| 141 | 5 | ALU | Np | 1 | 182 | 1 | 21 | 35.4 | | | | 38.27 | 0.93 | | | 5 | Np | ALU | |
| 142 | 5 | ALU | Np | 1 | 118 | 2 | 4 | 1.9 | | | | 38.27 | 0.05 | | | 5 | Np | ALU | |
| 143 | 5 | ALU | Np | 1 | 143 | 5 | 20 | 37.4 | | | | 38.27 | 0.98 | | | 5 | Np | ALU | |
| 144 | 5 | ALU | Np | 1 | 112 | 6 | 10 | 25.8 | 25.13 | | | 38.27 | 0.67 | 0.657 | | 5 | Np | ALU | |
| 145 | 5 | ALU | Np | 2 | 101 | 3 | 15 | 0.5 | | | | 38.27 | 0.01 | | | 5 | Np | ALU | |
| 146 | 5 | ALU | Np | 2 | 160 | 4 | 7 | 0.9 | | | | 38.27 | 0.02 | | | 5 | Np | ALU | |
| 147 | 5 | ALU | Np | 2 | 181 | 7 | 20 | 5.8 | | | | 38.27 | 0.15 | | | 5 | Np | ALU | |
| 148 | 5 | ALU | Np | 2 | 198 | 8 | 22 | 40.2 | 11.85 | | | 38.27 | 1.05 | 0.310 | | 5 | Np | ALU | |
| 149 | 5 | ALU | Np | 3 | 177 | 1 | 16 | 37.3 | | | | 38.27 | 0.97 | | | 5 | Np | ALU | |
| 150 | 5 | ALU | Np | 3 | 110 | 2 | 9 | 25.3 | | | | 38.27 | 0.66 | | | 5 | Np | ALU | |
| 151 | 5 | ALU | Np | 3 | 125 | 5 | 5 | 40.0 | | | | 38.27 | 1.05 | | | 5 | Np | ALU | |
| 152 | 5 | ALU | Np | 3 | 166 | 6 | 25 | 36.7 | 34.83 | | | 38.27 | 0.96 | 0.910 | | 5 | Np | ALU | |
| 153 | 5 | ALU | Np | 4 | 102 | 3 | 1 | NA | | | | NA | NA | | | 5 | Np | ALU | |
| 154 | 5 | ALU | Np | 4 | 159 | 4 | 25 | 0.3 | | | | 38.27 | 0.01 | | | 5 | Np | ALU | |
| 155 | 5 | ALU | Np | 4 | 188 | 7 | 15 | 1.6 | | | | 38.27 | 0.04 | | | 5 | Np | ALU | |
| 156 | 5 | ALU | Np | 4 | 139 | 8 | 16 | NA | 0.95 | | | NA | NA | 0.025 | | 5 | Np | ALU | |
| 157 | 5 | ALU | Np | 5 | 190 | 2 | 24 | 0.6 | | | | 38.27 | 0.02 | | | 5 | Np | ALU | |
| 158 | 5 | ALU | Np | 5 | 171 | 4 | 24 | 34.2 | | | | 38.27 | 0.89 | | | 5 | Np | ALU | |
| 159 | 5 | ALU | Np | 5 | 153 | 6 | 13 | 50.7 | | | | 38.27 | 1.32 | | | 5 | Np | ALU | |
| 160 | 5 | ALU | Np | 5 | 161 | 8 | 23 | 0.6 | 21.53 | 18.86 | | 38.27 | 0.02 | 0.562 | 0.493 | 5 | Np | ALU | 20.84 |
| 171 | 5 | ALU | Null | NA | 141 | 1 | 2 | 38.6 | | | | 38.27 | 1.01 | | | 5 | Null | ALU | |

# Supplemental Table S1

| | | | | | | | | | | | | |
|---|---|---|---|---|---|---|---|---|---|---|---|---|
| 20.84 | 1.83 | | | | 41.39 | 0.92 | | | | 5 | Pp | ALU |
| 20.84 | 2.38 | | | | 41.39 | 1.20 | | | | 5 | Pp | ALU |
| 20.84 | 1.74 | | | | 41.39 | 0.87 | | | | 5 | Pp | ALU |
| 20.84 | 1.87 | | | | 41.39 | 0.94 | | | | 5 | Pp | ALU |
| NA | NA | | | | NA | NA | | | | 5 | Pp | ALU |
| 20.84 | 1.77 | | | | 41.39 | 0.89 | | | | 5 | Pp | ALU |
| 20.84 | 2.03 | | | | 41.39 | 1.02 | | | | 5 | Pp | ALU |
| 20.84 | 2.46 | 2.01 | | | 41.39 | 1.24 | 1.013 | | | 5 | Pp | ALU |
| 20.84 | 2.38 | | | | 41.39 | 1.20 | | | | 5 | Pp | ALU |
| 20.84 | 1.73 | | | | 41.39 | 0.87 | | | | 5 | Pp | ALU |
| 20.84 | 2.08 | | | | 41.39 | 1.05 | | | | 5 | Pp | ALU |
| 20.84 | 2.56 | | | | 41.39 | 1.29 | | | | 5 | Pp | ALU |
| 20.84 | 1.92 | | | | 41.39 | 0.97 | | | | 5 | Pp | ALU |
| 20.84 | 1.76 | | | | 41.39 | 0.88 | | | | 5 | Pp | ALU |
| 20.84 | 2.03 | | | | 41.39 | 1.02 | | | | 5 | Pp | ALU |
| 20.84 | 1.88 | 2.04 | | | 41.39 | 0.95 | 1.029 | | | 5 | Pp | ALU |
| 20.84 | 3.10 | | | | 41.39 | 1.56 | | | | 5 | Pp | ALU |
| 20.84 | 2.23 | | | | 41.39 | 1.12 | | | | 5 | Pp | ALU |
| 20.84 | 1.85 | | | | 41.39 | 0.93 | | | | 5 | Pp | ALU |
| 20.84 | 2.14 | | | | 41.39 | 1.08 | | | | 5 | Pp | ALU |
| 20.84 | 2.28 | | | | 41.39 | 1.15 | | | | 5 | Pp | ALU |
| 20.84 | 2.08 | | | | 41.39 | 1.05 | | | | 5 | Pp | ALU |
| 20.84 | 0.60 | | | | 41.39 | 0.30 | | | | 5 | Pp | ALU |
| 20.84 | 2.29 | 2.07 | | | 41.39 | 1.15 | 1.044 | | | 5 | Pp | ALU |
| 20.84 | 2.39 | | | | 41.39 | 1.20 | | | | 5 | Pp | ALU |
| 20.84 | 2.32 | | | | 41.39 | 1.17 | | | | 5 | Pp | ALU |
| 20.84 | 2.05 | | | | 41.39 | 1.03 | | | | 5 | Pp | ALU |
| 20.84 | 1.84 | | | | 41.39 | 0.93 | | | | 5 | Pp | ALU |
| 20.84 | 2.52 | | | | 41.39 | 1.27 | | | | 5 | Pp | ALU |
| 20.84 | 1.98 | | Generation 5 | | 41.39 | 1.00 | | Generation 5 | | 5 | Pp | ALU |
| 20.84 | 2.00 | | Average | StDev | 41.39 | 1.01 | | Average | StDev | 5 | Pp | ALU |
| 20.84 | 1.70 | 2.10 | 2.11 | 0.13 | 41.39 | 0.86 | 1.058 | 1.064 | 0.064 | 5 | Pp | ALU |
| | | | | | | | | | | 5 | Np | ALU |
| | | | | | | | | | | 5 | Np | ALU |
| | | | | | | | | | | 5 | Np | ALU |
| | | | | | | | | | | 5 | Np | ALU |
| | | | | | | | | | | 5 | Np | ALU |
| | | | | | | | | | | 5 | Np | ALU |
| | | | | | | | | | | 5 | Np | ALU |
| | | | | | | | | | | 5 | Np | ALU |
| | | | | | | | | | | 5 | Np | ALU |
| | | | | | | | | | | 5 | Np | ALU |
| | | | | | | | | | | 5 | Np | ALU |
| | | | | | | | | | | 5 | Np | ALU |
| | | | | | | | | | | 5 | Np | ALU |
| | | | | | | | | | | 5 | Np | ALU |
| | | | | | | | | | | 5 | Np | ALU |
| | | | | | | | | | | 5 | Np | ALU |
| | | | | | | | | | | 5 | Np | ALU |
| | | | | | | | | | | 5 | Np | ALU |
| | | | | | | | | | | 5 | Np | ALU |
| | | | | | | | | | | 5 | Np | ALU |
| | | | | | | | | | | 5 | Null | ALU |

# Supplemental Table S1

| | | | | | | | | | | | | | | | | | | | |
|---|---|---|---|---|---|---|---|---|---|---|---|---|---|---|---|---|---|---|---|
| 172 | 5 | ALU | Null | NA | 117 | 2 | 23 | 41.3 | | | | 38.27 | 1.08 | | | 5 | Null | ALU | |
| 173 | 5 | ALU | Null | NA | 108 | 3 | 24 | 41.0 | | | | 38.27 | 1.07 | | | 5 | Null | ALU | |
| 174 | 5 | ALU | Null | NA | 191 | 4 | 10 | 37.7 | | | | 38.27 | 0.99 | | | 5 | Null | ALU | |
| 175 | 5 | ALU | Null | NA | 162 | 5 | 10 | 47.8 | | | | 38.27 | 1.25 | | | 5 | Null | ALU | |
| 176 | 5 | ALU | Null | NA | 170 | 6 | 1 | 40.7 | | | | 38.27 | 1.06 | | | 5 | Null | ALU | |
| 177 | 5 | ALU | Null | NA | 185 | 7 | 11 | 36.4 | | | | 38.27 | 0.95 | | | 5 | Null | ALU | |
| 178 | 5 | ALU | Null | NA | 155 | 8 | 20 | NA | | | | NA | NA | | | 5 | Null | ALU | |
| 179 | 5 | ALU | Null | NA | 105 | 1 | 11 | 54.9 | | | | 38.27 | 1.43 | | | 5 | Null | ALU | |
| 180 | 5 | ALU | Null | NA | 189 | 2 | 20 | 38.1 | | | | 38.27 | 1.00 | | | 5 | Null | ALU | |
| 181 | 5 | ALU | Null | NA | 123 | 3 | 12 | 44.3 | | | | 38.27 | 1.16 | | | 5 | Null | ALU | |
| 182 | 5 | ALU | Null | NA | 106 | 4 | 16 | 33.2 | | | | 38.27 | 0.87 | | | 5 | Null | ALU | |
| 183 | 5 | ALU | Null | NA | 148 | 5 | 18 | 44.7 | | | | 38.27 | 1.17 | | | 5 | Null | ALU | |
| 184 | 5 | ALU | Null | NA | 194 | 6 | 24 | 46.3 | | | | 38.27 | 1.21 | | | 5 | Null | ALU | |
| 185 | 5 | ALU | Null | NA | 180 | 7 | 17 | 46.4 | | | | 38.27 | 1.21 | | | 5 | Null | ALU | |
| 186 | 5 | ALU | Null | NA | 119 | 8 | 15 | 43.7 | | | | 38.27 | 1.14 | | | 5 | Null | ALU | |
| 187 | 5 | ALU | Null | NA | 157 | 1 | 12 | 44.5 | | | | 38.27 | 1.16 | | | 5 | Null | ALU | |
| 188 | 5 | ALU | Null | NA | 124 | 2 | 11 | 35.6 | | | | 38.27 | 0.93 | | | 5 | Null | ALU | |
| 189 | 5 | ALU | Null | NA | 138 | 3 | 18 | 37.6 | | | | 38.27 | 0.98 | | | 5 | Null | ALU | |
| 190 | 5 | ALU | Null | NA | 140 | 4 | 14 | 40.3 | | | | 38.27 | 1.05 | | | 5 | Null | ALU | |
| 191 | 5 | ALU | Null | NA | 109 | 5 | 2 | 42.0 | | | | 38.27 | 1.10 | | | 5 | Null | ALU | |
| 192 | 5 | ALU | Null | NA | 200 | 6 | 4 | 46.8 | | | | 38.27 | 1.22 | | | 5 | Null | ALU | |
| 193 | 5 | ALU | Null | NA | 167 | 7 | 22 | 40.4 | | | | 38.27 | 1.06 | | | 5 | Null | ALU | |
| 194 | 5 | ALU | Null | NA | 173 | 8 | 13 | 36.9 | | | | 38.27 | 0.96 | | | 5 | Null | ALU | |
| 195 | 5 | ALU | Null | NA | 158 | 2 | 6 | 40.8 | | | | 38.27 | 1.07 | | | 5 | Null | ALU | |
| 196 | 5 | ALU | Null | NA | 122 | 3 | 9 | 43.3 | | | | 38.27 | 1.13 | | | 5 | Null | ALU | |
| 197 | 5 | ALU | Null | NA | 152 | 4 | 3 | 43.6 | | | | 38.27 | 1.14 | | | 5 | Null | ALU | |
| 198 | 5 | ALU | Null | NA | 184 | 5 | 25 | NA | | | | NA | NA | | | 5 | Null | ALU | |
| 199 | 5 | ALU | Null | NA | 163 | 7 | 6 | 39.9 | | | Grand Average | 38.27 | 1.04 | | | 5 | Null | ALU | |
| 200 | 5 | ALU | Null | NA | 126 | 8 | 1 | 32.0 | 41.39 | | 38.27 | 38.27 | 0.84 | 1.081 | | 5 | Null | ALU | 41.39 |
| 1 | 6 | SOD | Pp | 1 | 4 | 1 | 25 | NA | | | | NA | NA | | | 6 | Pp | SOD | |
| 2 | 6 | SOD | Pp | 1 | 71 | 2 | 4 | NA | | | | NA | NA | | | 6 | Pp | SOD | |
| 3 | 6 | SOD | Pp | 1 | 58 | 3 | 16 | 34.8 | | | | 30.05 | 1.16 | | | 6 | Pp | SOD | |
| 4 | 6 | SOD | Pp | 1 | 8 | 4 | 24 | 34.2 | | | | 30.05 | 1.14 | | | 6 | Pp | SOD | |
| 5 | 6 | SOD | Pp | 1 | 12 | 5 | 23 | 30.7 | | | | 30.05 | 1.02 | | | 6 | Pp | SOD | |
| 6 | 6 | SOD | Pp | 1 | 13 | 6 | 16 | 25.1 | | | | 30.05 | 0.84 | | | 6 | Pp | SOD | |
| 7 | 6 | SOD | Pp | 1 | 68 | 7 | 7 | 40.4 | | | | 30.05 | 1.34 | | | 6 | Pp | SOD | |
| 8 | 6 | SOD | Pp | 1 | 83 | 8 | 17 | 35.3 | 33.42 | | | 30.05 | 1.17 | 1.112 | | 6 | Pp | SOD | |
| 9 | 6 | SOD | Pp | 2 | 37 | 1 | 7 | 39.5 | | | | 30.05 | 1.31 | | | 6 | Pp | SOD | |
| 10 | 6 | SOD | Pp | 2 | 67 | 2 | 10 | 29.7 | | | | 30.05 | 0.99 | | | 6 | Pp | SOD | |
| 11 | 6 | SOD | Pp | 2 | 54 | 3 | 22 | 21.4 | | | | 30.05 | 0.71 | | | 6 | Pp | SOD | |
| 12 | 6 | SOD | Pp | 2 | 28 | 4 | 21 | 34.6 | | | | 30.05 | 1.15 | | | 6 | Pp | SOD | |
| 13 | 6 | SOD | Pp | 2 | 66 | 5 | 16 | 30.9 | | | | 30.05 | 1.03 | | | 6 | Pp | SOD | |
| 14 | 6 | SOD | Pp | 2 | 94 | 6 | 20 | 35.1 | | | | 30.05 | 1.17 | | | 6 | Pp | SOD | |
| 15 | 6 | SOD | Pp | 2 | 89 | 7 | 4 | 27.6 | | | | 30.05 | 0.92 | | | 6 | Pp | SOD | |
| 16 | 6 | SOD | Pp | 2 | 75 | 8 | 24 | 20.6 | 29.93 | | | 30.05 | 0.69 | 0.996 | | 6 | Pp | SOD | |
| 17 | 6 | SOD | Pp | 3 | 48 | 1 | 22 | 36.2 | | | | 30.05 | 1.20 | | | 6 | Pp | SOD | |
| 18 | 6 | SOD | Pp | 3 | 42 | 2 | 7 | 35.3 | | | | 30.05 | 1.17 | | | 6 | Pp | SOD | |
| 19 | 6 | SOD | Pp | 3 | 23 | 3 | 13 | 37.8 | | | | 30.05 | 1.26 | | | 6 | Pp | SOD | |
| 20 | 6 | SOD | Pp | 3 | 73 | 4 | 19 | 41.3 | | | | 30.05 | 1.37 | | | 6 | Pp | SOD | |
| 21 | 6 | SOD | Pp | 3 | 95 | 5 | 15 | NA | | | | NA | NA | | | 6 | Pp | SOD | |
| 22 | 6 | SOD | Pp | 3 | 51 | 6 | 1 | 38.8 | | | | 30.05 | 1.29 | | | 6 | Pp | SOD | |
| 23 | 6 | SOD | Pp | 3 | 27 | 7 | 8 | 35.3 | | | | 30.05 | 1.17 | | | 6 | Pp | SOD | |
| 24 | 6 | SOD | Pp | 3 | 59 | 8 | 10 | 37.4 | 37.44 | | | 30.05 | 1.24 | 1.246 | | 6 | Pp | SOD | |

## Supplemental Table S1

| | | | | | | | | | | | | |
|---|---|---|---|---|---|---|---|---|---|---|---|---|
| | | | | | | | | | | 5 | Null | ALU |
| | | | | | | | | | | 5 | Null | ALU |
| | | | | | | | | | | 5 | Null | ALU |
| | | | | | | | | | | 5 | Null | ALU |
| | | | | | | | | | | 5 | Null | ALU |
| | | | | | | | | | | 5 | Null | ALU |
| | | | | | | | | | | 5 | Null | ALU |
| | | | | | | | | | | 5 | Null | ALU |
| | | | | | | | | | | 5 | Null | ALU |
| | | | | | | | | | | 5 | Null | ALU |
| | | | | | | | | | | 5 | Null | ALU |
| | | | | | | | | | | 5 | Null | ALU |
| | | | | | | | | | | 5 | Null | ALU |
| | | | | | | | | | | 5 | Null | ALU |
| | | | | | | | | | | 5 | Null | ALU |
| | | | | | | | | | | 5 | Null | ALU |
| | | | | | | | | | | 5 | Null | ALU |
| | | | | | | | | | | 5 | Null | ALU |
| | | | | | | | | | | 5 | Null | ALU |
| | | | | | | | | | | 5 | Null | ALU |
| | | | | | | | | | | 5 | Null | ALU |
| | | | | | | | | | | 5 | Null | ALU |
| | | | | | | | | | | 5 | Null | ALU |
| | | | | | | | | | | 5 | Null | ALU |
| | | | | | | | | | | 5 | Null | ALU |
| | | | | | | | | | | 5 | Null | ALU |
| | | | | | | | | | | 5 | Null | ALU |
| | | | | | | | | | | 5 | Null | ALU |
| | | | | | | | | | | 5 | Null | ALU |
| NA | NA | | | | NA | NA | | | | 6 | Pp | SOD |
| NA | NA | | | | NA | NA | | | | 6 | Pp | SOD |
| 19.09 | 1.82 | | | | 31.46 | 1.11 | | | | 6 | Pp | SOD |
| 19.09 | 1.79 | | | | 31.46 | 1.09 | | | | 6 | Pp | SOD |
| 19.09 | 1.61 | | | | 31.46 | 0.98 | | | | 6 | Pp | SOD |
| 19.09 | 1.31 | | | | 31.46 | 0.80 | | | | 6 | Pp | SOD |
| 19.09 | 2.12 | | | | 31.46 | 1.28 | | | | 6 | Pp | SOD |
| 19.09 | 1.85 | 1.75 | | | 31.46 | 1.12 | 1.062 | | | 6 | Pp | SOD |
| 19.09 | 2.07 | | | | 31.46 | 1.26 | | | | 6 | Pp | SOD |
| 19.09 | 1.56 | | | | 31.46 | 0.94 | | | | 6 | Pp | SOD |
| 19.09 | 1.12 | | | | 31.46 | 0.68 | | | | 6 | Pp | SOD |
| 19.09 | 1.81 | | | | 31.46 | 1.10 | | | | 6 | Pp | SOD |
| 19.09 | 1.62 | | | | 31.46 | 0.98 | | | | 6 | Pp | SOD |
| 19.09 | 1.84 | | | | 31.46 | 1.12 | | | | 6 | Pp | SOD |
| 19.09 | 1.45 | | | | 31.46 | 0.88 | | | | 6 | Pp | SOD |
| 19.09 | 1.08 | 1.57 | | | 31.46 | 0.65 | 0.951 | | | 6 | Pp | SOD |
| 19.09 | 1.90 | | | | 31.46 | 1.15 | | | | 6 | Pp | SOD |
| 19.09 | 1.85 | | | | 31.46 | 1.12 | | | | 6 | Pp | SOD |
| 19.09 | 1.98 | | | | 31.46 | 1.20 | | | | 6 | Pp | SOD |
| 19.09 | 2.16 | | | | 31.46 | 1.31 | | | | 6 | Pp | SOD |
| NA | NA | | | | NA | NA | | | | 6 | Pp | SOD |
| 19.09 | 2.03 | | | | 31.46 | 1.23 | | | | 6 | Pp | SOD |
| 19.09 | 1.85 | | | | 31.46 | 1.12 | | | | 6 | Pp | SOD |
| 19.09 | 1.96 | 1.96 | | | 31.46 | 1.19 | 1.190 | | | 6 | Pp | SOD |

## Supplemental Table S1

| | | | | | | | | | | | | | | | | | | | |
|---|---|---|---|---|---|---|---|---|---|---|---|---|---|---|---|---|---|---|---|
| 25 | 6 | SOD | Pp | 4 | 38 | 1 | 17 | 41.3 | | | | 30.05 | 1.37 | | | 6 | Pp | SOD | |
| 26 | 6 | SOD | Pp | 4 | 35 | 2 | 25 | 32.1 | | | | 30.05 | 1.07 | | | 6 | Pp | SOD | |
| 27 | 6 | SOD | Pp | 4 | 16 | 3 | 17 | 38.9 | | | | 30.05 | 1.29 | | | 6 | Pp | SOD | |
| 28 | 6 | SOD | Pp | 4 | 46 | 4 | 13 | 29.3 | | | | 30.05 | 0.98 | | | 6 | Pp | SOD | |
| 29 | 6 | SOD | Pp | 4 | 97 | 5 | 11 | 26.5 | | | | 30.05 | 0.88 | | | 6 | Pp | SOD | |
| 30 | 6 | SOD | Pp | 4 | 52 | 6 | 22 | 33.1 | | | | 30.05 | 1.10 | | | 6 | Pp | SOD | |
| 31 | 6 | SOD | Pp | 4 | 91 | 7 | 23 | 38.0 | | | | 30.05 | 1.26 | | | 6 | Pp | SOD | |
| 32 | 6 | SOD | Pp | 4 | 7 | 8 | 2 | 37.0 | 34.53 | | | 30.05 | 1.23 | 1.149 | | 6 | Pp | SOD | |
| 33 | 6 | SOD | Pp | 5 | 14 | 1 | 3 | 44.5 | | | | 30.05 | 1.48 | | | 6 | Pp | SOD | |
| 34 | 6 | SOD | Pp | 5 | 86 | 2 | 12 | 36.3 | | | | 30.05 | 1.21 | | | 6 | Pp | SOD | |
| 35 | 6 | SOD | Pp | 5 | 53 | 3 | 5 | 29.8 | | | | 30.05 | 0.99 | | | 6 | Pp | SOD | |
| 36 | 6 | SOD | Pp | 5 | 85 | 4 | 10 | 33.3 | | | | 30.05 | 1.11 | | | 6 | Pp | SOD | |
| 37 | 6 | SOD | Pp | 5 | 50 | 5 | 2 | 40.8 | | | | 30.05 | 1.36 | | | 6 | Pp | SOD | |
| 38 | 6 | SOD | Pp | 5 | 79 | 6 | 4 | 33.2 | | | | 30.05 | 1.10 | | | 6 | Pp | SOD | |
| 39 | 6 | SOD | Pp | 5 | 1 | 7 | 12 | NA | | | | NA | NA | | | 6 | Pp | SOD | |
| 40 | 6 | SOD | Pp | 5 | 19 | 8 | 13 | 41.8 | 37.10 | 34.48 | | 30.05 | 1.39 | 1.235 | 1.148 | 6 | Pp | SOD | 34.39 |
| 41 | 6 | SOD | Np | 1 | 64 | 1 | 6 | NA | | | | NA | NA | | | 6 | Np | SOD | |
| 42 | 6 | SOD | Np | 1 | 41 | 2 | 11 | 0.6 | | | | 30.05 | 0.02 | | | 6 | Np | SOD | |
| 43 | 6 | SOD | Np | 1 | 98 | 5 | 17 | 29.5 | | | | 30.05 | 0.98 | | | 6 | Np | SOD | |
| 44 | 6 | SOD | Np | 1 | 72 | 6 | 17 | 8.1 | 12.73 | | | 30.05 | 0.27 | 0.424 | | 6 | Np | SOD | |
| 45 | 6 | SOD | Np | 2 | 57 | 3 | 6 | 5.5 | | | | 30.05 | 0.18 | | | 6 | Np | SOD | |
| 46 | 6 | SOD | Np | 2 | 62 | 4 | 6 | 23.3 | | | | 30.05 | 0.78 | | | 6 | Np | SOD | |
| 47 | 6 | SOD | Np | 2 | 80 | 7 | 11 | 0.8 | | | | 30.05 | 0.03 | | | 6 | Np | SOD | |
| 48 | 6 | SOD | Np | 2 | 31 | 8 | 5 | 28.5 | 14.53 | | | 30.05 | 0.95 | 0.483 | | 6 | Np | SOD | |
| 49 | 6 | SOD | Np | 3 | 56 | 1 | 8 | 26.9 | | | | 30.05 | 0.90 | | | 6 | Np | SOD | |
| 50 | 6 | SOD | Np | 3 | 44 | 2 | 19 | 30.1 | | | | 30.05 | 1.00 | | | 6 | Np | SOD | |
| 51 | 6 | SOD | Np | 3 | 70 | 5 | 5 | 34.1 | | | | 30.05 | 1.13 | | | 6 | Np | SOD | |
| 52 | 6 | SOD | Np | 3 | 34 | 6 | 7 | 39.7 | 32.70 | | | 30.05 | 1.32 | 1.088 | | 6 | Np | SOD | |
| 53 | 6 | SOD | Np | 4 | 78 | 3 | 2 | 17.0 | | | | 30.05 | 0.57 | | | 6 | Np | SOD | |
| 54 | 6 | SOD | Np | 4 | 61 | 4 | 5 | 19.8 | | | | 30.05 | 0.66 | | | 6 | Np | SOD | |
| 55 | 6 | SOD | Np | 4 | 93 | 7 | 25 | 36.9 | | | | 30.05 | 1.23 | | | 6 | Np | SOD | |
| 56 | 6 | SOD | Np | 4 | 74 | 8 | 8 | 1.0 | 18.68 | | | 30.05 | 0.03 | 0.622 | | 6 | Np | SOD | |
| 57 | 6 | SOD | Np | 5 | 43 | 1 | 23 | 19.1 | | | | 30.05 | 0.64 | | | 6 | Np | SOD | |
| 58 | 6 | SOD | Np | 5 | 92 | 3 | 25 | 22.4 | | | | 30.05 | 0.75 | | | 6 | Np | SOD | |
| 59 | 6 | SOD | Np | 5 | 9 | 5 | 18 | NA | | | | NA | NA | | | 6 | Np | SOD | |
| 60 | 6 | SOD | Np | 5 | 63 | 7 | 10 | 0.4 | 13.97 | 18.52 | | 30.05 | 0.01 | 0.465 | 0.616 | 6 | Np | SOD | 19.09 |
| 71 | 6 | SOD | Null | NA | 2 | 1 | 12 | 23.6 | | | | 30.05 | 0.79 | | | 6 | Null | SOD | |
| 72 | 6 | SOD | Null | NA | 90 | 2 | 24 | 31.9 | | | | 30.05 | 1.06 | | | 6 | Null | SOD | |
| 73 | 6 | SOD | Null | NA | 45 | 3 | 24 | 34.2 | | | | 30.05 | 1.14 | | | 6 | Null | SOD | |
| 74 | 6 | SOD | Null | NA | 39 | 4 | 16 | 36.0 | | | | 30.05 | 1.20 | | | 6 | Null | SOD | |
| 75 | 6 | SOD | Null | NA | 82 | 5 | 4 | 33.7 | | | | 30.05 | 1.12 | | | 6 | Null | SOD | |
| 76 | 6 | SOD | Null | NA | 69 | 6 | 23 | 36.4 | | | | 30.05 | 1.21 | | | 6 | Null | SOD | |
| 77 | 6 | SOD | Null | NA | 76 | 7 | 22 | 31.1 | | | | 30.05 | 1.04 | | | 6 | Null | SOD | |
| 78 | 6 | SOD | Null | NA | 77 | 8 | 6 | 22.6 | | | | 30.05 | 0.75 | | | 6 | Null | SOD | |
| 79 | 6 | SOD | Null | NA | 30 | 1 | 1 | 32.4 | | | | 30.05 | 1.08 | | | 6 | Null | SOD | |
| 80 | 6 | SOD | Null | NA | 29 | 2 | 22 | 23.3 | | | | 30.05 | 0.78 | | | 6 | Null | SOD | |
| 81 | 6 | SOD | Null | NA | 20 | 3 | 14 | 31.9 | | | | 30.05 | 1.06 | | | 6 | Null | SOD | |
| 82 | 6 | SOD | Null | NA | 65 | 4 | 7 | 32.2 | | | | 30.05 | 1.07 | | | 6 | Null | SOD | |
| 83 | 6 | SOD | Null | NA | 21 | 5 | 12 | 33.6 | | | | 30.05 | 1.12 | | | 6 | Null | SOD | |
| 84 | 6 | SOD | Null | NA | 18 | 6 | 25 | 22.6 | | | | 30.05 | 0.75 | | | 6 | Null | SOD | |
| 85 | 6 | SOD | Null | NA | 40 | 7 | 2 | 35.8 | | | | 30.05 | 1.19 | | | 6 | Null | SOD | |
| 86 | 6 | SOD | Null | NA | 100 | 8 | 4 | 30.4 | | | | 30.05 | 1.01 | | | 6 | Null | SOD | |
| 87 | 6 | SOD | Null | NA | 15 | 1 | 9 | 32.6 | | | | 30.05 | 1.08 | | | 6 | Null | SOD | |

# Supplemental Table S1

| | | | | | | | | | | | | |
|---|---|---|---|---|---|---|---|---|---|---|---|---|
| 19.09 | 2.16 | | | | 31.46 | 1.31 | | | | 6 | Pp | SOD |
| 19.09 | 1.68 | | | | 31.46 | 1.02 | | | | 6 | Pp | SOD |
| 19.09 | 2.04 | | | | 31.46 | 1.24 | | | | 6 | Pp | SOD |
| 19.09 | 1.53 | | | | 31.46 | 0.93 | | | | 6 | Pp | SOD |
| 19.09 | 1.39 | | | | 31.46 | 0.84 | | | | 6 | Pp | SOD |
| 19.09 | 1.73 | | | | 31.46 | 1.05 | | | | 6 | Pp | SOD |
| 19.09 | 1.99 | | | | 31.46 | 1.21 | | | | 6 | Pp | SOD |
| 19.09 | 1.94 | 1.81 | | | 31.46 | 1.18 | 1.097 | | | 6 | Pp | SOD |
| 19.09 | 2.33 | | | | 31.46 | 1.41 | | | | 6 | Pp | SOD |
| 19.09 | 1.90 | | | | 31.46 | 1.15 | | | | 6 | Pp | SOD |
| 19.09 | 1.56 | | | | 31.46 | 0.95 | | | | 6 | Pp | SOD |
| 19.09 | 1.74 | | | | 31.46 | 1.06 | | | | 6 | Pp | SOD |
| 19.09 | 2.14 | | | | 31.46 | 1.30 | | | | 6 | Pp | SOD |
| 19.09 | 1.74 | | Generation 6 | | 31.46 | 1.06 | | Generation 6 | | 6 | Pp | SOD |
| NA | NA | | Average | StDev | NA | NA | | Average | StDev | 6 | Pp | SOD |
| 19.09 | 2.19 | 1.94 | 1.81 | 0.16 | 31.46 | 1.33 | 1.179 | 1.096 | 0.097 | 6 | Pp | SOD |
| | | | | | | | | | | 6 | Np | SOD |
| | | | | | | | | | | 6 | Np | SOD |
| | | | | | | | | | | 6 | Np | SOD |
| | | | | | | | | | | 6 | Np | SOD |
| | | | | | | | | | | 6 | Np | SOD |
| | | | | | | | | | | 6 | Np | SOD |
| | | | | | | | | | | 6 | Np | SOD |
| | | | | | | | | | | 6 | Np | SOD |
| | | | | | | | | | | 6 | Np | SOD |
| | | | | | | | | | | 6 | Np | SOD |
| | | | | | | | | | | 6 | Np | SOD |
| | | | | | | | | | | 6 | Np | SOD |
| | | | | | | | | | | 6 | Np | SOD |
| | | | | | | | | | | 6 | Np | SOD |
| | | | | | | | | | | 6 | Np | SOD |
| | | | | | | | | | | 6 | Np | SOD |
| | | | | | | | | | | 6 | Np | SOD |
| | | | | | | | | | | 6 | Np | SOD |
| | | | | | | | | | | 6 | Np | SOD |
| | | | | | | | | | | 6 | Np | SOD |
| | | | | | | | | | | 6 | Null | SOD |
| | | | | | | | | | | 6 | Null | SOD |
| | | | | | | | | | | 6 | Null | SOD |
| | | | | | | | | | | 6 | Null | SOD |
| | | | | | | | | | | 6 | Null | SOD |
| | | | | | | | | | | 6 | Null | SOD |
| | | | | | | | | | | 6 | Null | SOD |
| | | | | | | | | | | 6 | Null | SOD |
| | | | | | | | | | | 6 | Null | SOD |
| | | | | | | | | | | 6 | Null | SOD |
| | | | | | | | | | | 6 | Null | SOD |
| | | | | | | | | | | 6 | Null | SOD |
| | | | | | | | | | | 6 | Null | SOD |
| | | | | | | | | | | 6 | Null | SOD |
| | | | | | | | | | | 6 | Null | SOD |
| | | | | | | | | | | 6 | Null | SOD |
| | | | | | | | | | | 6 | Null | SOD |

# Supplemental Table S1

| | | | | | | | | | | | | | | | | | | | |
|---|---|---|---|---|---|---|---|---|---|---|---|---|---|---|---|---|---|---|---|
| 88 | 6 | SOD | Null | NA | 6 | 2 | 13 | 32.2 | | | | 30.05 | 1.07 | | | 6 | Null | SOD | |
| 89 | 6 | SOD | Null | NA | 84 | 3 | 12 | 35.8 | | | | 30.05 | 1.19 | | | 6 | Null | SOD | |
| 90 | 6 | SOD | Null | NA | 49 | 4 | 1 | NA | | | | NA | NA | | | 6 | Null | SOD | |
| 91 | 6 | SOD | Null | NA | 33 | 5 | 3 | 32.9 | | | | 30.05 | 1.09 | | | 6 | Null | SOD | |
| 92 | 6 | SOD | Null | NA | 55 | 6 | 14 | 36.2 | | | | 30.05 | 1.20 | | | 6 | Null | SOD | |
| 93 | 6 | SOD | Null | NA | 25 | 7 | 16 | 29.2 | | | | 30.05 | 0.97 | | | 6 | Null | SOD | |
| 94 | 6 | SOD | Null | NA | 24 | 8 | 20 | 33.2 | | | | 30.05 | 1.10 | | | 6 | Null | SOD | |
| 95 | 6 | SOD | Null | NA | 81 | 1 | 15 | 34.8 | | | | 30.05 | 1.16 | | | 6 | Null | SOD | |
| 96 | 6 | SOD | Null | NA | 3 | 3 | 10 | 31.0 | | | | 30.05 | 1.03 | | | 6 | Null | SOD | |
| 97 | 6 | SOD | Null | NA | 5 | 4 | 8 | 30.5 | | | | 30.05 | 1.02 | | | 6 | Null | SOD | |
| 98 | 6 | SOD | Null | NA | 17 | 5 | 14 | 29.8 | | | | 30.05 | 0.99 | | | 6 | Null | SOD | |
| 99 | 6 | SOD | Null | NA | 60 | 6 | 10 | 35.4 | | | Grand Average | 30.05 | 1.18 | | | 6 | Null | SOD | |
| 100 | 6 | SOD | Null | NA | 96 | 8 | 11 | 27.0 | 31.46 | | 30.05 | 30.05 | 0.90 | 1.047 | | 6 | Null | SOD | 31.46 |
| 101 | 6 | ALU | Pp | 1 | 116 | 1 | 10 | 38.9 | | | | 39.96 | 0.97 | | | 6 | Pp | ALU | |
| 102 | 6 | ALU | Pp | 1 | 122 | 2 | 1 | 40.2 | | | | 39.96 | 1.01 | | | 6 | Pp | ALU | |
| 103 | 6 | ALU | Pp | 1 | 102 | 3 | 7 | 40.5 | | | | 39.96 | 1.01 | | | 6 | Pp | ALU | |
| 104 | 6 | ALU | Pp | 1 | 196 | 4 | 2 | 34.3 | | | | 39.96 | 0.86 | | | 6 | Pp | ALU | |
| 105 | 6 | ALU | Pp | 1 | 188 | 5 | 10 | 43.5 | | | | 39.96 | 1.09 | | | 6 | Pp | ALU | |
| 106 | 6 | ALU | Pp | 1 | 192 | 6 | 3 | 38.2 | | | | 39.96 | 0.96 | | | 6 | Pp | ALU | |
| 107 | 6 | ALU | Pp | 1 | 131 | 7 | 6 | NA | | | | NA | NA | | | 6 | Pp | ALU | |
| 108 | 6 | ALU | Pp | 1 | 158 | 8 | 3 | 47.4 | 40.43 | | | 39.96 | 1.19 | 1.012 | | 6 | Pp | ALU | |
| 109 | 6 | ALU | Pp | 2 | 178 | 1 | 19 | 46.3 | | | | 39.96 | 1.16 | | | 6 | Pp | ALU | |
| 110 | 6 | ALU | Pp | 2 | 139 | 2 | 23 | 47.3 | | | | 39.96 | 1.18 | | | 6 | Pp | ALU | |
| 111 | 6 | ALU | Pp | 2 | 172 | 3 | 4 | 48.9 | | | | 39.96 | 1.22 | | | 6 | Pp | ALU | |
| 112 | 6 | ALU | Pp | 2 | 169 | 4 | 9 | 44.4 | | | | 39.96 | 1.11 | | | 6 | Pp | ALU | |
| 113 | 6 | ALU | Pp | 2 | 150 | 5 | 9 | 37.2 | | | | 39.96 | 0.93 | | | 6 | Pp | ALU | |
| 114 | 6 | ALU | Pp | 2 | 177 | 6 | 8 | NA | | | | NA | NA | | | 6 | Pp | ALU | |
| 115 | 6 | ALU | Pp | 2 | 160 | 7 | 14 | 40.5 | | | | 39.96 | 1.01 | | | 6 | Pp | ALU | |
| 116 | 6 | ALU | Pp | 2 | 127 | 8 | 15 | 45.0 | 44.23 | | | 39.96 | 1.13 | 1.107 | | 6 | Pp | ALU | |
| 117 | 6 | ALU | Pp | 3 | 140 | 1 | 20 | 49.3 | | | | 39.96 | 1.23 | | | 6 | Pp | ALU | |
| 118 | 6 | ALU | Pp | 3 | 117 | 2 | 2 | 45.5 | | | | 39.96 | 1.14 | | | 6 | Pp | ALU | |
| 119 | 6 | ALU | Pp | 3 | 137 | 3 | 1 | 38.0 | | | | 39.96 | 0.95 | | | 6 | Pp | ALU | |
| 120 | 6 | ALU | Pp | 3 | 121 | 4 | 3 | 42.5 | | | | 39.96 | 1.06 | | | 6 | Pp | ALU | |
| 121 | 6 | ALU | Pp | 3 | 171 | 5 | 1 | 38.9 | | | | 39.96 | 0.97 | | | 6 | Pp | ALU | |
| 122 | 6 | ALU | Pp | 3 | 106 | 6 | 15 | 48.5 | | | | 39.96 | 1.21 | | | 6 | Pp | ALU | |
| 123 | 6 | ALU | Pp | 3 | 168 | 7 | 19 | 40.5 | | | | 39.96 | 1.01 | | | 6 | Pp | ALU | |
| 124 | 6 | ALU | Pp | 3 | 157 | 8 | 18 | 35.5 | 42.34 | | | 39.96 | 0.89 | 1.060 | | 6 | Pp | ALU | |
| 125 | 6 | ALU | Pp | 4 | 163 | 1 | 14 | 51.8 | | | | 39.96 | 1.30 | | | 6 | Pp | ALU | |
| 126 | 6 | ALU | Pp | 4 | 175 | 2 | 14 | NA | | | | NA | NA | | | 6 | Pp | ALU | |
| 127 | 6 | ALU | Pp | 4 | 200 | 3 | 18 | 41.2 | | | | 39.96 | 1.03 | | | 6 | Pp | ALU | |
| 128 | 6 | ALU | Pp | 4 | 134 | 4 | 14 | 47.6 | | | | 39.96 | 1.19 | | | 6 | Pp | ALU | |
| 129 | 6 | ALU | Pp | 4 | 147 | 5 | 20 | 41.0 | | | | 39.96 | 1.03 | | | 6 | Pp | ALU | |
| 130 | 6 | ALU | Pp | 4 | 189 | 6 | 9 | 53.2 | | | | 39.96 | 1.33 | | | 6 | Pp | ALU | |
| 131 | 6 | ALU | Pp | 4 | 191 | 7 | 20 | 47.0 | | | | 39.96 | 1.18 | | | 6 | Pp | ALU | |
| 132 | 6 | ALU | Pp | 4 | 104 | 8 | 9 | 43.6 | 46.49 | | | 39.96 | 1.09 | 1.163 | | 6 | Pp | ALU | |
| 133 | 6 | ALU | Pp | 5 | 186 | 1 | 18 | 38.1 | | | | 39.96 | 0.95 | | | 6 | Pp | ALU | |
| 134 | 6 | ALU | Pp | 5 | 133 | 2 | 3 | 46.6 | | | | 39.96 | 1.17 | | | 6 | Pp | ALU | |
| 135 | 6 | ALU | Pp | 5 | 197 | 3 | 19 | NA | | | | NA | NA | | | 6 | Pp | ALU | |
| 136 | 6 | ALU | Pp | 5 | 105 | 4 | 18 | 48.1 | | | | 39.96 | 1.20 | | | 6 | Pp | ALU | |
| 137 | 6 | ALU | Pp | 5 | 180 | 5 | 25 | 42.8 | | | | 39.96 | 1.07 | | | 6 | Pp | ALU | |
| 138 | 6 | ALU | Pp | 5 | 179 | 6 | 18 | 46.8 | | | | 39.96 | 1.17 | | | 6 | Pp | ALU | |
| 139 | 6 | ALU | Pp | 5 | 136 | 7 | 17 | 56.4 | | | | 39.96 | 1.41 | | | 6 | Pp | ALU | |
| 140 | 6 | ALU | Pp | 5 | 149 | 8 | 12 | 36.1 | 44.99 | 43.69 | | 39.96 | 0.90 | 1.126 | 1.093 | 6 | Pp | ALU | 43.66 |

# Supplemental Table S1

| | | | | | | | | | | | | |
|---|---|---|---|---|---|---|---|---|---|---|---|---|
| | | | | | | | | | | 6 | Null | SOD |
| | | | | | | | | | | 6 | Null | SOD |
| | | | | | | | | | | 6 | Null | SOD |
| | | | | | | | | | | 6 | Null | SOD |
| | | | | | | | | | | 6 | Null | SOD |
| | | | | | | | | | | 6 | Null | SOD |
| | | | | | | | | | | 6 | Null | SOD |
| | | | | | | | | | | 6 | Null | SOD |
| | | | | | | | | | | 6 | Null | SOD |
| | | | | | | | | | | 6 | Null | SOD |
| | | | | | | | | | | 6 | Null | SOD |
| | | | | | | | | | | 6 | Null | SOD |
| | | | | | | | | | | 6 | Null | SOD |
| 34.35 | 1.13 | | | | 39.05 | 1.00 | | | | 6 | Pp | ALU |
| 34.35 | 1.17 | | | | 39.05 | 1.03 | | | | 6 | Pp | ALU |
| 34.35 | 1.18 | | | | 39.05 | 1.04 | | | | 6 | Pp | ALU |
| 34.35 | 1.00 | | | | 39.05 | 0.88 | | | | 6 | Pp | ALU |
| 34.35 | 1.27 | | | | 39.05 | 1.11 | | | | 6 | Pp | ALU |
| 34.35 | 1.11 | | | | 39.05 | 0.98 | | | | 6 | Pp | ALU |
| NA | NA | | | | NA | NA | | | | 6 | Pp | ALU |
| 34.35 | 1.38 | 1.18 | | | 39.05 | 1.21 | 1.035 | | | 6 | Pp | ALU |
| 34.35 | 1.35 | | | | 39.05 | 1.19 | | | | 6 | Pp | ALU |
| 34.35 | 1.38 | | | | 39.05 | 1.21 | | | | 6 | Pp | ALU |
| 34.35 | 1.42 | | | | 39.05 | 1.25 | | | | 6 | Pp | ALU |
| 34.35 | 1.29 | | | | 39.05 | 1.14 | | | | 6 | Pp | ALU |
| 34.35 | 1.08 | | | | 39.05 | 0.95 | | | | 6 | Pp | ALU |
| NA | NA | | | | NA | NA | | | | 6 | Pp | ALU |
| 34.35 | 1.18 | | | | 39.05 | 1.04 | | | | 6 | Pp | ALU |
| 34.35 | 1.31 | 1.29 | | | 39.05 | 1.15 | 1.133 | | | 6 | Pp | ALU |
| 34.35 | 1.44 | | | | 39.05 | 1.26 | | | | 6 | Pp | ALU |
| 34.35 | 1.32 | | | | 39.05 | 1.17 | | | | 6 | Pp | ALU |
| 34.35 | 1.11 | | | | 39.05 | 0.97 | | | | 6 | Pp | ALU |
| 34.35 | 1.24 | | | | 39.05 | 1.09 | | | | 6 | Pp | ALU |
| 34.35 | 1.13 | | | | 39.05 | 1.00 | | | | 6 | Pp | ALU |
| 34.35 | 1.41 | | | | 39.05 | 1.24 | | | | 6 | Pp | ALU |
| 34.35 | 1.18 | | | | 39.05 | 1.04 | | | | 6 | Pp | ALU |
| 34.35 | 1.03 | 1.23 | | | 39.05 | 0.91 | 1.084 | | | 6 | Pp | ALU |
| 34.35 | 1.51 | | | | 39.05 | 1.33 | | | | 6 | Pp | ALU |
| NA | NA | | | | NA | NA | | | | 6 | Pp | ALU |
| 34.35 | 1.20 | | | | 39.05 | 1.06 | | | | 6 | Pp | ALU |
| 34.35 | 1.39 | | | | 39.05 | 1.22 | | | | 6 | Pp | ALU |
| 34.35 | 1.19 | | | | 39.05 | 1.05 | | | | 6 | Pp | ALU |
| 34.35 | 1.55 | | | | 39.05 | 1.36 | | | | 6 | Pp | ALU |
| 34.35 | 1.37 | | | | 39.05 | 1.20 | | | | 6 | Pp | ALU |
| 34.35 | 1.27 | 1.35 | | | 39.05 | 1.12 | 1.190 | | | 6 | Pp | ALU |
| 34.35 | 1.11 | | | | 39.05 | 0.98 | | | | 6 | Pp | ALU |
| 34.35 | 1.36 | | | | 39.05 | 1.19 | | | | 6 | Pp | ALU |
| NA | NA | | | | NA | NA | | | | 6 | Pp | ALU |
| 34.35 | 1.40 | | | | 39.05 | 1.23 | | | | 6 | Pp | ALU |
| 34.35 | 1.25 | | | | 39.05 | 1.10 | | | | 6 | Pp | ALU |
| 34.35 | 1.36 | | Generation 6 | | 39.05 | 1.20 | | Generation 6 | | 6 | Pp | ALU |
| 34.35 | 1.64 | | Average | StDev | 39.05 | 1.44 | | Average | StDev | 6 | Pp | ALU |
| 34.35 | 1.05 | 1.31 | 1.27 | 0.07 | 39.05 | 0.92 | 1.152 | 1.119 | 0.060 | 6 | Pp | ALU |

# Supplemental Table S1

| | | | | | | | | | | | | | | | | | | | |
|---|---|---|---|---|---|---|---|---|---|---|---|---|---|---|---|---|---|---|---|
| 141 | 6 | ALU | Np | 1 | 156 | 1 | 24 | 35.6 | | | | 39.96 | 0.89 | | | 6 | Np | ALU | |
| 142 | 6 | ALU | Np | 1 | 132 | 2 | 8 | 47.3 | | | | 39.96 | 1.18 | | | 6 | Np | ALU | |
| 143 | 6 | ALU | Np | 1 | 125 | 5 | 19 | 42.3 | | | | 39.96 | 1.06 | | | 6 | Np | ALU | |
| 144 | 6 | ALU | Np | 1 | 144 | 6 | 5 | 29.5 | 38.68 | | | 39.96 | 0.74 | 0.968 | | 6 | Np | ALU | |
| 145 | 6 | ALU | Np | 2 | 130 | 3 | 3 | 37.4 | | | | 39.96 | 0.94 | | | 6 | Np | ALU | |
| 146 | 6 | ALU | Np | 2 | 193 | 4 | 4 | 32.2 | | | | 39.96 | 0.81 | | | 6 | Np | ALU | |
| 147 | 6 | ALU | Np | 2 | 173 | 7 | 15 | 35.9 | | | | 39.96 | 0.90 | | | 6 | Np | ALU | |
| 148 | 6 | ALU | Np | 2 | 141 | 8 | 14 | 33.9 | 34.85 | | | 39.96 | 0.85 | 0.872 | | 6 | Np | ALU | |
| 149 | 6 | ALU | Np | 3 | 114 | 1 | 11 | 33.3 | | | | 39.96 | 0.83 | | | 6 | Np | ALU | |
| 150 | 6 | ALU | Np | 3 | 164 | 2 | 17 | 32.4 | | | | 39.96 | 0.81 | | | 6 | Np | ALU | |
| 151 | 6 | ALU | Np | 3 | 108 | 5 | 13 | 31.7 | | | | 39.96 | 0.79 | | | 6 | Np | ALU | |
| 152 | 6 | ALU | Np | 3 | 113 | 6 | 6 | 26.4 | 30.95 | | | 39.96 | 0.66 | 0.775 | | 6 | Np | ALU | |
| 153 | 6 | ALU | Np | 4 | 146 | 3 | 21 | 37.8 | | | | 39.96 | 0.95 | | | 6 | Np | ALU | |
| 154 | 6 | ALU | Np | 4 | 112 | 4 | 22 | NA | | | | NA | NA | | | 6 | Np | ALU | |
| 155 | 6 | ALU | Np | 4 | 165 | 7 | 24 | 36.6 | | | | 39.96 | 0.92 | | | 6 | Np | ALU | |
| 156 | 6 | ALU | Np | 4 | 194 | 8 | 25 | 35.7 | 36.70 | | | 39.96 | 0.89 | 0.918 | | 6 | Np | ALU | |
| 157 | 6 | ALU | Np | 5 | 190 | 2 | 16 | 31.3 | | | | 39.96 | 0.78 | | | 6 | Np | ALU | |
| 158 | 6 | ALU | Np | 5 | 142 | 4 | 17 | 35.6 | | | | 39.96 | 0.89 | | | 6 | Np | ALU | |
| 159 | 6 | ALU | Np | 5 | 124 | 6 | 11 | 30.5 | | | | 39.96 | 0.76 | | | 6 | Np | ALU | |
| 160 | 6 | ALU | Np | 5 | 159 | 8 | 23 | 27.2 | 31.15 | 34.47 | | 39.96 | 0.68 | 0.780 | 0.862 | 6 | Np | ALU | 34.35 |
| 171 | 6 | ALU | Null | NA | 199 | 1 | 21 | 35.3 | | | | 39.96 | 0.88 | | | 6 | Null | ALU | |
| 172 | 6 | ALU | Null | NA | 176 | 2 | 6 | 35.2 | | | | 39.96 | 0.88 | | | 6 | Null | ALU | |
| 173 | 6 | ALU | Null | NA | 185 | 3 | 9 | 38.9 | | | | 39.96 | 0.97 | | | 6 | Null | ALU | |
| 174 | 6 | ALU | Null | NA | 145 | 4 | 23 | 37.8 | | | | 39.96 | 0.95 | | | 6 | Null | ALU | |
| 175 | 6 | ALU | Null | NA | 184 | 5 | 22 | 38.0 | | | | 39.96 | 0.95 | | | 6 | Null | ALU | |
| 176 | 6 | ALU | Null | NA | 135 | 6 | 21 | 42.1 | | | | 39.96 | 1.05 | | | 6 | Null | ALU | |
| 177 | 6 | ALU | Null | NA | 128 | 7 | 13 | 47.0 | | | | 39.96 | 1.18 | | | 6 | Null | ALU | |
| 178 | 6 | ALU | Null | NA | 119 | 8 | 22 | 36.0 | | | | 39.96 | 0.90 | | | 6 | Null | ALU | |
| 179 | 6 | ALU | Null | NA | 198 | 1 | 4 | 27.6 | | | | 39.96 | 0.69 | | | 6 | Null | ALU | |
| 180 | 6 | ALU | Null | NA | 138 | 2 | 18 | 37.4 | | | | 39.96 | 0.94 | | | 6 | Null | ALU | |
| 181 | 6 | ALU | Null | NA | 107 | 3 | 15 | 40.4 | | | | 39.96 | 1.01 | | | 6 | Null | ALU | |
| 182 | 6 | ALU | Null | NA | 111 | 4 | 25 | 34.5 | | | | 39.96 | 0.86 | | | 6 | Null | ALU | |
| 183 | 6 | ALU | Null | NA | 151 | 5 | 7 | 39.7 | | | | 39.96 | 0.99 | | | 6 | Null | ALU | |
| 184 | 6 | ALU | Null | NA | 162 | 6 | 19 | NA | | | | NA | NA | | | 6 | Null | ALU | |
| 185 | 6 | ALU | Null | NA | 152 | 7 | 18 | 43.6 | | | | 39.96 | 1.09 | | | 6 | Null | ALU | |
| 186 | 6 | ALU | Null | NA | 109 | 8 | 16 | 41.4 | | | | 39.96 | 1.04 | | | 6 | Null | ALU | |
| 187 | 6 | ALU | Null | NA | 174 | 1 | 2 | 37.0 | | | | 39.96 | 0.93 | | | 6 | Null | ALU | |
| 188 | 6 | ALU | Null | NA | 181 | 2 | 15 | 46.2 | | | | 39.96 | 1.16 | | | 6 | Null | ALU | |
| 189 | 6 | ALU | Null | NA | 120 | 3 | 8 | 42.2 | | | | 39.96 | 1.06 | | | 6 | Null | ALU | |
| 190 | 6 | ALU | Null | NA | 143 | 4 | 20 | 40.7 | | | | 39.96 | 1.02 | | | 6 | Null | ALU | |
| 191 | 6 | ALU | Null | NA | 103 | 5 | 21 | 36.2 | | | | 39.96 | 0.91 | | | 6 | Null | ALU | |
| 192 | 6 | ALU | Null | NA | 129 | 6 | 24 | 41.7 | | | | 39.96 | 1.04 | | | 6 | Null | ALU | |
| 193 | 6 | ALU | Null | NA | 154 | 7 | 9 | 38.0 | | | | 39.96 | 0.95 | | | 6 | Null | ALU | |
| 194 | 6 | ALU | Null | NA | 115 | 8 | 1 | 39.4 | | | | 39.96 | 0.99 | | | 6 | Null | ALU | |
| 195 | 6 | ALU | Null | NA | 123 | 2 | 21 | 37.1 | | | | 39.96 | 0.93 | | | 6 | Null | ALU | |
| 196 | 6 | ALU | Null | NA | 126 | 3 | 23 | 41.8 | | | | 39.96 | 1.05 | | | 6 | Null | ALU | |
| 197 | 6 | ALU | Null | NA | 182 | 4 | 12 | 41.4 | | | | 39.96 | 1.04 | | | 6 | Null | ALU | |
| 198 | 6 | ALU | Null | NA | 167 | 5 | 24 | 38.2 | | | | 39.96 | 0.96 | | | 6 | Null | ALU | |
| 199 | 6 | ALU | Null | NA | 110 | 7 | 3 | 40.9 | | | Grand Average | 39.96 | 1.02 | | | 6 | Null | ALU | |
| 200 | 6 | ALU | Null | NA | 148 | 8 | 21 | 36.7 | 39.05 | | 39.96 | 39.96 | 0.92 | 0.977 | | 6 | Null | ALU | 39.05 |
| 1 | 7 | SOD | Pp | 1 | 12 | 1 | 17 | 60.9 | | | | 55.01 | 1.11 | | | 7 | Pp | SOD | |
| 2 | 7 | SOD | Pp | 1 | 100 | 2 | 13 | 65.0 | | | | 55.01 | 1.18 | | | 7 | Pp | SOD | |
| 3 | 7 | SOD | Pp | 1 | 93 | 3 | 8 | 56.0 | | | | 55.01 | 1.02 | | | 7 | Pp | SOD | |

# Supplemental Table S1

| | | | | | | | | | | | | |
|---|---|---|---|---|---|---|---|---|---|---|---|---|
| | | | | | | | | | | 6 | Np | ALU |
| | | | | | | | | | | 6 | Np | ALU |
| | | | | | | | | | | 6 | Np | ALU |
| | | | | | | | | | | 6 | Np | ALU |
| | | | | | | | | | | 6 | Np | ALU |
| | | | | | | | | | | 6 | Np | ALU |
| | | | | | | | | | | 6 | Np | ALU |
| | | | | | | | | | | 6 | Np | ALU |
| | | | | | | | | | | 6 | Np | ALU |
| | | | | | | | | | | 6 | Np | ALU |
| | | | | | | | | | | 6 | Np | ALU |
| | | | | | | | | | | 6 | Np | ALU |
| | | | | | | | | | | 6 | Np | ALU |
| | | | | | | | | | | 6 | Np | ALU |
| | | | | | | | | | | 6 | Np | ALU |
| | | | | | | | | | | 6 | Np | ALU |
| | | | | | | | | | | 6 | Np | ALU |
| | | | | | | | | | | 6 | Np | ALU |
| | | | | | | | | | | 6 | Np | ALU |
| | | | | | | | | | | 6 | Np | ALU |
| | | | | | | | | | | 6 | Null | ALU |
| | | | | | | | | | | 6 | Null | ALU |
| | | | | | | | | | | 6 | Null | ALU |
| | | | | | | | | | | 6 | Null | ALU |
| | | | | | | | | | | 6 | Null | ALU |
| | | | | | | | | | | 6 | Null | ALU |
| | | | | | | | | | | 6 | Null | ALU |
| | | | | | | | | | | 6 | Null | ALU |
| | | | | | | | | | | 6 | Null | ALU |
| | | | | | | | | | | 6 | Null | ALU |
| | | | | | | | | | | 6 | Null | ALU |
| | | | | | | | | | | 6 | Null | ALU |
| | | | | | | | | | | 6 | Null | ALU |
| | | | | | | | | | | 6 | Null | ALU |
| | | | | | | | | | | 6 | Null | ALU |
| | | | | | | | | | | 6 | Null | ALU |
| | | | | | | | | | | 6 | Null | ALU |
| | | | | | | | | | | 6 | Null | ALU |
| | | | | | | | | | | 6 | Null | ALU |
| | | | | | | | | | | 6 | Null | ALU |
| | | | | | | | | | | 6 | Null | ALU |
| | | | | | | | | | | 6 | Null | ALU |
| | | | | | | | | | | 6 | Null | ALU |
| | | | | | | | | | | 6 | Null | ALU |
| | | | | | | | | | | 6 | Null | ALU |
| | | | | | | | | | | 6 | Null | ALU |
| | | | | | | | | | | 6 | Null | ALU |
| | | | | | | | | | | 6 | Null | ALU |
| | | | | | | | | | | 6 | Null | ALU |
| | | | | | | | | | | 6 | Null | ALU |
| 36.69 | 1.66 | | | | 58.75 | 1.04 | | | | 7 | Pp | SOD |
| 36.69 | 1.77 | | | | 58.75 | 1.11 | | | | 7 | Pp | SOD |
| 36.69 | 1.53 | | | | 58.75 | 0.95 | | | | 7 | Pp | SOD |

# Supplemental Table S1

| | | | | | | | | | | | | | | | | | | | |
|---|---|---|---|---|---|---|---|---|---|---|---|---|---|---|---|---|---|---|---|
| 4 | 7 | SOD | Pp | 1 | 11 | 4 | 3 | 67.4 | | | | 55.01 | 1.23 | | | 7 | Pp | SOD | |
| 5 | 7 | SOD | Pp | 1 | 5 | 5 | 11 | 65.0 | | | | 55.01 | 1.18 | | | 7 | Pp | SOD | |
| 6 | 7 | SOD | Pp | 1 | 85 | 6 | 18 | 66.3 | | | | 55.01 | 1.21 | | | 7 | Pp | SOD | |
| 7 | 7 | SOD | Pp | 1 | 49 | 7 | 24 | 76.0 | | | | 55.01 | 1.38 | | | 7 | Pp | SOD | |
| 8 | 7 | SOD | Pp | 1 | 16 | 8 | 23 | 70.5 | 65.89 | | | 55.01 | 1.28 | 1.198 | | 7 | Pp | SOD | |
| 9 | 7 | SOD | Pp | 2 | 69 | 1 | 14 | 68.3 | | | | 55.01 | 1.24 | | | 7 | Pp | SOD | |
| 10 | 7 | SOD | Pp | 2 | 6 | 2 | 3 | 67.5 | | | | 55.01 | 1.23 | | | 7 | Pp | SOD | |
| 11 | 7 | SOD | Pp | 2 | 36 | 3 | 22 | 60.6 | | | | 55.01 | 1.10 | | | 7 | Pp | SOD | |
| 12 | 7 | SOD | Pp | 2 | 60 | 4 | 12 | 56.1 | | | | 55.01 | 1.02 | | | 7 | Pp | SOD | |
| 13 | 7 | SOD | Pp | 2 | 97 | 5 | 20 | 60.5 | | | | 55.01 | 1.10 | | | 7 | Pp | SOD | |
| 14 | 7 | SOD | Pp | 2 | 14 | 6 | 21 | NA | | | | NA | NA | | | 7 | Pp | SOD | |
| 15 | 7 | SOD | Pp | 2 | 63 | 7 | 7 | 72.0 | | | | 55.01 | 1.31 | | | 7 | Pp | SOD | |
| 16 | 7 | SOD | Pp | 2 | 9 | 8 | 12 | 73.2 | 65.46 | | | 55.01 | 1.33 | 1.190 | | 7 | Pp | SOD | |
| 17 | 7 | SOD | Pp | 3 | 55 | 1 | 11 | 44.0 | | | | 55.01 | 0.80 | | | 7 | Pp | SOD | |
| 18 | 7 | SOD | Pp | 3 | 89 | 2 | 25 | 64.1 | | | | 55.01 | 1.17 | | | 7 | Pp | SOD | |
| 19 | 7 | SOD | Pp | 3 | 8 | 3 | 21 | 67.2 | | | | 55.01 | 1.22 | | | 7 | Pp | SOD | |
| 20 | 7 | SOD | Pp | 3 | 45 | 4 | 24 | 56.6 | | | | 55.01 | 1.03 | | | 7 | Pp | SOD | |
| 21 | 7 | SOD | Pp | 3 | 87 | 5 | 21 | 50.3 | | | | 55.01 | 0.91 | | | 7 | Pp | SOD | |
| 22 | 7 | SOD | Pp | 3 | 7 | 6 | 22 | NA | | | | NA | NA | | | 7 | Pp | SOD | |
| 23 | 7 | SOD | Pp | 3 | 46 | 7 | 13 | 58.2 | | | | 55.01 | 1.06 | | | 7 | Pp | SOD | |
| 24 | 7 | SOD | Pp | 3 | 42 | 8 | 24 | 46.0 | 55.20 | | | 55.01 | 0.84 | 1.003 | | 7 | Pp | SOD | |
| 25 | 7 | SOD | Pp | 4 | 53 | 1 | 16 | 61.6 | | | | 55.01 | 1.12 | | | 7 | Pp | SOD | |
| 26 | 7 | SOD | Pp | 4 | 83 | 2 | 15 | 62.8 | | | | 55.01 | 1.14 | | | 7 | Pp | SOD | |
| 27 | 7 | SOD | Pp | 4 | 76 | 3 | 3 | 46.6 | | | | 55.01 | 0.85 | | | 7 | Pp | SOD | |
| 28 | 7 | SOD | Pp | 4 | 41 | 4 | 18 | 57.8 | | | | 55.01 | 1.05 | | | 7 | Pp | SOD | x |
| 29 | 7 | SOD | Pp | 4 | 35 | 5 | 10 | 47.9 | | | | 55.01 | 0.87 | | | 7 | Pp | SOD | x |
| 30 | 7 | SOD | Pp | 4 | 32 | 6 | 4 | 63.8 | | | | 55.01 | 1.16 | | | 7 | Pp | SOD | |
| 31 | 7 | SOD | Pp | 4 | 40 | 7 | 20 | 52.1 | | | | 55.01 | 0.95 | | | 7 | Pp | SOD | |
| 32 | 7 | SOD | Pp | 4 | 84 | 8 | 22 | 55.2 | 55.98 | | | 55.01 | 1.00 | 1.018 | | 7 | Pp | SOD | |
| 33 | 7 | SOD | Pp | 5 | 58 | 1 | 10 | 68.2 | | | | 55.01 | 1.24 | | | 7 | Pp | SOD | |
| 34 | 7 | SOD | Pp | 5 | 74 | 2 | 14 | 69.2 | | | | 55.01 | 1.26 | | | 7 | Pp | SOD | |
| 35 | 7 | SOD | Pp | 5 | 29 | 3 | 13 | 70.7 | | | | 55.01 | 1.29 | | | 7 | Pp | SOD | |
| 36 | 7 | SOD | Pp | 5 | 33 | 4 | 6 | 57.0 | | | | 55.01 | 1.04 | | | 7 | Pp | SOD | |
| 37 | 7 | SOD | Pp | 5 | 96 | 5 | 3 | 59.3 | | | | 55.01 | 1.08 | | | 7 | Pp | SOD | |
| 38 | 7 | SOD | Pp | 5 | 90 | 6 | 1 | 45.5 | | | | 55.01 | 0.83 | | | 7 | Pp | SOD | |
| 39 | 7 | SOD | Pp | 5 | 3 | 7 | 21 | 71.0 | | | | 55.01 | 1.29 | | | 7 | Pp | SOD | |
| 40 | 7 | SOD | Pp | 5 | 88 | 8 | 21 | 65.7 | 63.33 | 61.17 | | 55.01 | 1.19 | 1.151 | 1.112 | 7 | Pp | SOD | 61.21 |
| 41 | 7 | SOD | Np | 1 | 23 | 1 | 5 | 0.8 | | | | 55.01 | 0.01 | | | 7 | Np | SOD | |
| 42 | 7 | SOD | Np | 1 | 82 | 2 | 4 | 52.0 | | | | 55.01 | 0.95 | | | 7 | Np | SOD | |
| 43 | 7 | SOD | Np | 1 | 15 | 5 | 18 | 47.1 | | | | 55.01 | 0.86 | | | 7 | Np | SOD | |
| 44 | 7 | SOD | Np | 1 | 56 | 6 | 11 | 55.6 | 38.88 | | | 55.01 | 1.01 | 0.707 | | 7 | Np | SOD | |
| 45 | 7 | SOD | Np | 2 | 48 | 3 | 6 | 47.6 | | | | 55.01 | 0.87 | | | 7 | Np | SOD | |
| 46 | 7 | SOD | Np | 2 | 39 | 4 | 10 | 51.1 | | | | 55.01 | 0.93 | | | 7 | Np | SOD | |
| 47 | 7 | SOD | Np | 2 | 26 | 7 | 4 | 52.6 | | | | 55.01 | 0.96 | | | 7 | Np | SOD | |
| 48 | 7 | SOD | Np | 2 | 70 | 8 | 7 | 56.4 | 51.93 | | | 55.01 | 1.03 | 0.944 | | 7 | Np | SOD | |
| 49 | 7 | SOD | Np | 3 | 67 | 1 | 24 | 67.0 | | | | 55.01 | 1.22 | | | 7 | Np | SOD | |
| 50 | 7 | SOD | Np | 3 | 52 | 2 | 10 | 76.2 | | | | 55.01 | 1.39 | | | 7 | Np | SOD | |
| 51 | 7 | SOD | Np | 3 | 81 | 5 | 13 | 1.6 | | | | 55.01 | 0.03 | | | 7 | Np | SOD | |
| 52 | 7 | SOD | Np | 3 | 80 | 6 | 12 | 1.4 | 36.55 | | | 55.01 | 0.03 | 0.664 | | 7 | Np | SOD | |
| 53 | 7 | SOD | Np | 4 | 79 | 3 | 7 | NA | | | | NA | NA | | | 7 | Np | SOD | |
| 54 | 7 | SOD | Np | 4 | 59 | 4 | 15 | 0.8 | | | | 55.01 | 0.01 | | | 7 | Np | SOD | |
| 55 | 7 | SOD | Np | 4 | 1 | 7 | 16 | 0.4 | | | | 55.01 | 0.01 | | | 7 | Np | SOD | |
| 56 | 7 | SOD | Np | 4 | 99 | 8 | 2 | 46.6 | 15.93 | | | 55.01 | 0.85 | 0.290 | | 7 | Np | SOD | |

## Supplemental Table S1

| | | | | | | | | | | | | |
|---|---|---|---|---|---|---|---|---|---|---|---|---|
| 36.69 | 1.84 | | | | 58.75 | 1.15 | | | | 7 | Pp | SOD |
| 36.69 | 1.77 | | | | 58.75 | 1.11 | | | | 7 | Pp | SOD |
| 36.69 | 1.81 | | | | 58.75 | 1.13 | | | | 7 | Pp | SOD |
| 36.69 | 2.07 | | | | 58.75 | 1.29 | | | | 7 | Pp | SOD |
| 36.69 | 1.92 | 1.80 | | | 58.75 | 1.20 | 1.122 | | | 7 | Pp | SOD |
| 36.69 | 1.86 | | | | 58.75 | 1.16 | | | | 7 | Pp | SOD |
| 36.69 | 1.84 | | | | 58.75 | 1.15 | | | | 7 | Pp | SOD |
| 36.69 | 1.65 | | | | 58.75 | 1.03 | | | | 7 | Pp | SOD |
| 36.69 | 1.53 | | | | 58.75 | 0.95 | | | | 7 | Pp | SOD |
| 36.69 | 1.65 | | | | 58.75 | 1.03 | | | | 7 | Pp | SOD |
| NA | NA | | | | NA | NA | | | | 7 | Pp | SOD |
| 36.69 | 1.96 | | | | 58.75 | 1.23 | | | | 7 | Pp | SOD |
| 36.69 | 1.99 | 1.78 | | | 58.75 | 1.25 | 1.114 | | | 7 | Pp | SOD |
| 36.69 | 1.20 | | | | 58.75 | 0.75 | | | | 7 | Pp | SOD |
| 36.69 | 1.75 | | | | 58.75 | 1.09 | | | | 7 | Pp | SOD |
| 36.69 | 1.83 | | | | 58.75 | 1.14 | | | | 7 | Pp | SOD |
| 36.69 | 1.54 | | | | 58.75 | 0.96 | | | | 7 | Pp | SOD |
| 36.69 | 1.37 | | | | 58.75 | 0.86 | | | | 7 | Pp | SOD |
| NA | NA | | | | NA | NA | | | | 7 | Pp | SOD |
| 36.69 | 1.59 | | | | 58.75 | 0.99 | | | | 7 | Pp | SOD |
| 36.69 | 1.25 | 1.50 | | | 58.75 | 0.78 | 0.940 | | | 7 | Pp | SOD |
| 36.69 | 1.68 | | | | 58.75 | 1.05 | | | | 7 | Pp | SOD |
| 36.69 | 1.71 | | | | 58.75 | 1.07 | | | | 7 | Pp | SOD |
| 36.69 | 1.27 | | | | 58.75 | 0.79 | | | | 7 | Pp | SOD |
| 36.69 | 1.58 | | | | 58.75 | 0.98 | | | | 7 | Pp | SOD |
| 36.69 | 1.31 | | | | 58.75 | 0.82 | | | | 7 | Pp | SOD |
| 36.69 | 1.74 | | | | 58.75 | 1.09 | | | | 7 | Pp | SOD |
| 36.69 | 1.42 | | | | 58.75 | 0.89 | | | | 7 | Pp | SOD |
| 36.69 | 1.50 | 1.53 | | | 58.75 | 0.94 | 0.953 | | | 7 | Pp | SOD |
| 36.69 | 1.86 | | | | 58.75 | 1.16 | | | | 7 | Pp | SOD |
| 36.69 | 1.89 | | | | 58.75 | 1.18 | | | | 7 | Pp | SOD |
| 36.69 | 1.93 | | | | 58.75 | 1.20 | | | | 7 | Pp | SOD |
| 36.69 | 1.55 | | | | 58.75 | 0.97 | | | | 7 | Pp | SOD |
| 36.69 | 1.62 | | | | 58.75 | 1.01 | | | | 7 | Pp | SOD |
| 36.69 | 1.24 | | Generation 7 | | 58.75 | 0.77 | | Generation 7 | | 7 | Pp | SOD |
| 36.69 | 1.93 | | Average | StDev | 58.75 | 1.21 | | Average | StDev | 7 | Pp | SOD |
| 36.69 | 1.79 | 1.73 | 1.67 | 0.14 | 58.75 | 1.12 | 1.078 | 1.041 | 0.088 | 7 | Pp | SOD |
| | | | | | | | | | | 7 | Np | SOD |
| | | | | | | | | | | 7 | Np | SOD |
| | | | | | | | | | | 7 | Np | SOD |
| | | | | | | | | | | 7 | Np | SOD |
| | | | | | | | | | | 7 | Np | SOD |
| | | | | | | | | | | 7 | Np | SOD |
| | | | | | | | | | | 7 | Np | SOD |
| | | | | | | | | | | 7 | Np | SOD |
| | | | | | | | | | | 7 | Np | SOD |
| | | | | | | | | | | 7 | Np | SOD |
| | | | | | | | | | | 7 | Np | SOD |
| | | | | | | | | | | 7 | Np | SOD |
| | | | | | | | | | | 7 | Np | SOD |
| | | | | | | | | | | 7 | Np | SOD |
| | | | | | | | | | | 7 | Np | SOD |
| | | | | | | | | | | 7 | Np | SOD |

# Supplemental Table S1

| | | | | | | | | | | | | | | | | | | | |
|---|---|---|---|---|---|---|---|---|---|---|---|---|---|---|---|---|---|---|---|
| 57 | 7 | SOD | Np | 5 | 19 | 1 | 19 | 47.9 | | | | 55.01 | 0.87 | | | 7 | Np | SOD | |
| 58 | 7 | SOD | Np | 5 | 31 | 3 | 25 | 38.0 | | | | 55.01 | 0.69 | | | 7 | Np | SOD | |
| 59 | 7 | SOD | Np | 5 | 65 | 5 | 8 | 53.6 | | | | 55.01 | 0.97 | | | 7 | Np | SOD | |
| 60 | 7 | SOD | Np | 5 | 44 | 7 | 3 | 0.5 | 35.00 | 35.66 | | 55.01 | 0.01 | 0.636 | 0.648 | 7 | Np | SOD | 36.69 |
| 71 | 7 | SOD | Null | NA | 13 | 1 | 3 | 55.8 | | | | 55.01 | 1.01 | | | 7 | Null | SOD | |
| 72 | 7 | SOD | Null | NA | 51 | 2 | 18 | 52.0 | | | | 55.01 | 0.95 | | | 7 | Null | SOD | |
| 73 | 7 | SOD | Null | NA | 22 | 3 | 11 | 47.1 | | | | 55.01 | 0.86 | | | 7 | Null | SOD | |
| 74 | 7 | SOD | Null | NA | 24 | 4 | 9 | 55.6 | | | | 55.01 | 1.01 | | | 7 | Null | SOD | |
| 75 | 7 | SOD | Null | NA | 38 | 5 | 4 | 47.6 | | | | 55.01 | 0.87 | | | 7 | Null | SOD | |
| 76 | 7 | SOD | Null | NA | 37 | 6 | 3 | 51.1 | | | | 55.01 | 0.93 | | | 7 | Null | SOD | |
| 77 | 7 | SOD | Null | NA | 54 | 7 | 12 | 52.6 | | | | 55.01 | 0.96 | | | 7 | Null | SOD | |
| 78 | 7 | SOD | Null | NA | 78 | 8 | 8 | 56.4 | | | | 55.01 | 1.03 | | | 7 | Null | SOD | |
| 79 | 7 | SOD | Null | NA | 86 | 1 | 6 | 59.1 | | | | 55.01 | 1.07 | | | 7 | Null | SOD | |
| 80 | 7 | SOD | Null | NA | 4 | 2 | 20 | 57.9 | | | | 55.01 | 1.05 | | | 7 | Null | SOD | |
| 81 | 7 | SOD | Null | NA | 43 | 3 | 17 | 67.7 | | | | 55.01 | 1.23 | | | 7 | Null | SOD | |
| 82 | 7 | SOD | Null | NA | 17 | 4 | 2 | 55.8 | | | | 55.01 | 1.01 | | | 7 | Null | SOD | |
| 83 | 7 | SOD | Null | NA | 73 | 5 | 12 | 62.3 | | | | 55.01 | 1.13 | | | 7 | Null | SOD | |
| 84 | 7 | SOD | Null | NA | 71 | 6 | 13 | 74.1 | | | | 55.01 | 1.35 | | | 7 | Null | SOD | |
| 85 | 7 | SOD | Null | NA | 21 | 7 | 22 | 35.5 | | | | 55.01 | 0.65 | | | 7 | Null | SOD | |
| 86 | 7 | SOD | Null | NA | 61 | 8 | 20 | 59.9 | | | | 55.01 | 1.09 | | | 7 | Null | SOD | |
| 87 | 7 | SOD | Null | NA | 34 | 1 | 4 | 64.9 | | | | 55.01 | 1.18 | | | 7 | Null | SOD | |
| 88 | 7 | SOD | Null | NA | 98 | 2 | 5 | 59.0 | | | | 55.01 | 1.07 | | | 7 | Null | SOD | |
| 89 | 7 | SOD | Null | NA | 91 | 3 | 5 | 64.0 | | | | 55.01 | 1.16 | | | 7 | Null | SOD | |
| 90 | 7 | SOD | Null | NA | 66 | 4 | 19 | 58.0 | | | | 55.01 | 1.05 | | | 7 | Null | SOD | |
| 91 | 7 | SOD | Null | NA | 95 | 5 | 2 | 58.2 | | | | 55.01 | 1.06 | | | 7 | Null | SOD | |
| 92 | 7 | SOD | Null | NA | 62 | 6 | 9 | 71.2 | | | | 55.01 | 1.29 | | | 7 | Null | SOD | |
| 93 | 7 | SOD | Null | NA | 75 | 7 | 25 | 59.5 | | | | 55.01 | 1.08 | | | 7 | Null | SOD | |
| 94 | 7 | SOD | Null | NA | 2 | 8 | 18 | 70.8 | | | | 55.01 | 1.29 | | | 7 | Null | SOD | |
| 95 | 7 | SOD | Null | NA | 30 | 1 | 22 | 68.2 | | | | 55.01 | 1.24 | | | 7 | Null | SOD | |
| 96 | 7 | SOD | Null | NA | 94 | 3 | 14 | 56.5 | | | | 55.01 | 1.03 | | | 7 | Null | SOD | |
| 97 | 7 | SOD | Null | NA | 72 | 4 | 25 | 52.8 | | | | 55.01 | 0.96 | | | 7 | Null | SOD | |
| 98 | 7 | SOD | Null | NA | 68 | 5 | 22 | 67.6 | | | | 55.01 | 1.23 | | | 7 | Null | SOD | |
| 99 | 7 | SOD | Null | NA | 57 | 6 | 7 | 62.7 | | | Grand Average | 55.01 | 1.14 | | | 7 | Null | SOD | |
| 100 | 7 | SOD | Null | NA | 47 | 8 | 17 | 58.5 | 58.75 | | 55.01 | 55.01 | 1.06 | 1.068 | | 7 | Null | SOD | 58.75 |
| 101 | 7 | ALU | Pp | 1 | 161 | 1 | 2 | 88.4 | | | | 80.09 | 1.10 | | | 7 | Pp | ALU | |
| 102 | 7 | ALU | Pp | 1 | 174 | 2 | 7 | 100.5 | | | | 80.09 | 1.25 | | | 7 | Pp | ALU | |
| 103 | 7 | ALU | Pp | 1 | 137 | 3 | 24 | 93.9 | | | | 80.09 | 1.17 | | | 7 | Pp | ALU | |
| 104 | 7 | ALU | Pp | 1 | 112 | 4 | 23 | 84.1 | | | | 80.09 | 1.05 | | | 7 | Pp | ALU | |
| 105 | 7 | ALU | Pp | 1 | 156 | 5 | 5 | 80.7 | | | | 80.09 | 1.01 | | | 7 | Pp | ALU | |
| 106 | 7 | ALU | Pp | 1 | 193 | 6 | 16 | 86.6 | | | | 80.09 | 1.08 | | | 7 | Pp | ALU | |
| 107 | 7 | ALU | Pp | 1 | 149 | 7 | 14 | 100.1 | | | | 80.09 | 1.25 | | | 7 | Pp | ALU | |
| 108 | 7 | ALU | Pp | 1 | 101 | 8 | 5 | 94.7 | 91.13 | | | 80.09 | 1.18 | 1.138 | | 7 | Pp | ALU | |
| 109 | 7 | ALU | Pp | 2 | 167 | 1 | 1 | 63.7 | | | | 80.09 | 0.80 | | | 7 | Pp | ALU | |
| 110 | 7 | ALU | Pp | 2 | 190 | 2 | 22 | 102.1 | | | | 80.09 | 1.27 | | | 7 | Pp | ALU | |
| 111 | 7 | ALU | Pp | 2 | 154 | 3 | 9 | NA | | | | NA | NA | | | 7 | Pp | ALU | |
| 112 | 7 | ALU | Pp | 2 | 148 | 4 | 16 | 86.9 | | | | 80.09 | 1.08 | | | 7 | Pp | ALU | |
| 113 | 7 | ALU | Pp | 2 | 183 | 5 | 25 | 80.6 | | | | 80.09 | 1.01 | | | 7 | Pp | ALU | |
| 114 | 7 | ALU | Pp | 2 | 189 | 6 | 8 | 120.4 | | | | 80.09 | 1.50 | | | 7 | Pp | ALU | |
| 115 | 7 | ALU | Pp | 2 | 160 | 7 | 1 | 93.9 | | | | 80.09 | 1.17 | | | 7 | Pp | ALU | |
| 116 | 7 | ALU | Pp | 2 | 110 | 8 | 25 | 70.3 | 88.27 | | | 80.09 | 0.88 | 1.102 | | 7 | Pp | ALU | |
| 117 | 7 | ALU | Pp | 3 | 192 | 1 | 8 | 85.5 | | | | 80.09 | 1.07 | | | 7 | Pp | ALU | |
| 118 | 7 | ALU | Pp | 3 | 103 | 2 | 17 | 86.5 | | | | 80.09 | 1.08 | | | 7 | Pp | ALU | |
| 119 | 7 | ALU | Pp | 3 | 171 | 3 | 2 | 46.1 | | | | 80.09 | 0.58 | | | 7 | Pp | ALU | |

## Supplemental Table S1

| | | | | | | | | | | | | |
|---|---|---|---|---|---|---|---|---|---|---|---|---|
| | | | | | | | | | | 7 | Np | SOD |
| | | | | | | | | | | 7 | Np | SOD |
| | | | | | | | | | | 7 | Np | SOD |
| | | | | | | | | | | 7 | Np | SOD |
| | | | | | | | | | | 7 | Null | SOD |
| | | | | | | | | | | 7 | Null | SOD |
| | | | | | | | | | | 7 | Null | SOD |
| | | | | | | | | | | 7 | Null | SOD |
| | | | | | | | | | | 7 | Null | SOD |
| | | | | | | | | | | 7 | Null | SOD |
| | | | | | | | | | | 7 | Null | SOD |
| | | | | | | | | | | 7 | Null | SOD |
| | | | | | | | | | | 7 | Null | SOD |
| | | | | | | | | | | 7 | Null | SOD |
| | | | | | | | | | | 7 | Null | SOD |
| | | | | | | | | | | 7 | Null | SOD |
| | | | | | | | | | | 7 | Null | SOD |
| | | | | | | | | | | 7 | Null | SOD |
| | | | | | | | | | | 7 | Null | SOD |
| | | | | | | | | | | 7 | Null | SOD |
| | | | | | | | | | | 7 | Null | SOD |
| | | | | | | | | | | 7 | Null | SOD |
| | | | | | | | | | | 7 | Null | SOD |
| | | | | | | | | | | 7 | Null | SOD |
| | | | | | | | | | | 7 | Null | SOD |
| | | | | | | | | | | 7 | Null | SOD |
| | | | | | | | | | | 7 | Null | SOD |
| | | | | | | | | | | 7 | Null | SOD |
| | | | | | | | | | | 7 | Null | SOD |
| | | | | | | | | | | 7 | Null | SOD |
| | | | | | | | | | | 7 | Null | SOD |
| | | | | | | | | | | 7 | Null | SOD |
| | | | | | | | | | | 7 | Null | SOD |
| | | | | | | | | | | 7 | Null | SOD |
| 64.83 | 1.36 | | | | 80.30 | 1.10 | | | | 7 | Pp | ALU |
| 64.83 | 1.55 | | | | 80.30 | 1.25 | | | | 7 | Pp | ALU |
| 64.83 | 1.45 | | | | 80.30 | 1.17 | | | | 7 | Pp | ALU |
| 64.83 | 1.30 | | | | 80.30 | 1.05 | | | | 7 | Pp | ALU |
| 64.83 | 1.24 | | | | 80.30 | 1.00 | | | | 7 | Pp | ALU |
| 64.83 | 1.34 | | | | 80.30 | 1.08 | | | | 7 | Pp | ALU |
| 64.83 | 1.54 | | | | 80.30 | 1.25 | | | | 7 | Pp | ALU |
| 64.83 | 1.46 | 1.41 | | | 80.30 | 1.18 | 1.135 | | | 7 | Pp | ALU |
| 64.83 | 0.98 | | | | 80.30 | 0.79 | | | | 7 | Pp | ALU |
| 64.83 | 1.58 | | | | 80.30 | 1.27 | | | | 7 | Pp | ALU |
| NA | NA | | | | NA | NA | | | | 7 | Pp | ALU |
| 64.83 | 1.34 | | | | 80.30 | 1.08 | | | | 7 | Pp | ALU |
| 64.83 | 1.24 | | | | 80.30 | 1.00 | | | | 7 | Pp | ALU |
| 64.83 | 1.86 | | | | 80.30 | 1.50 | | | | 7 | Pp | ALU |
| 64.83 | 1.45 | | | | 80.30 | 1.17 | | | | 7 | Pp | ALU |
| 64.83 | 1.08 | 1.36 | | | 80.30 | 0.88 | 1.099 | | | 7 | Pp | ALU |
| 64.83 | 1.32 | | | | 80.30 | 1.06 | | | | 7 | Pp | ALU |
| 64.83 | 1.33 | | | | 80.30 | 1.08 | | | | 7 | Pp | ALU |
| 64.83 | 0.71 | | | | 80.30 | 0.57 | | | | 7 | Pp | ALU |

# Supplemental Table S1

| | | | | | | | | | | | | | | | | | | | |
|---|---|---|---|---|---|---|---|---|---|---|---|---|---|---|---|---|---|---|---|
| 120 | 7 | ALU | Pp | 3 | 150 | 4 | 14 | 87.9 | | | | 80.09 | 1.10 | | | 7 | Pp | ALU | |
| 121 | 7 | ALU | Pp | 3 | 186 | 5 | 6 | 82.8 | | | | 80.09 | 1.03 | | | 7 | Pp | ALU | |
| 122 | 7 | ALU | Pp | 3 | 133 | 6 | 19 | 93.1 | | | | 80.09 | 1.16 | | | 7 | Pp | ALU | |
| 123 | 7 | ALU | Pp | 3 | 115 | 7 | 6 | 69.3 | | | | 80.09 | 0.87 | | | 7 | Pp | ALU | |
| 124 | 7 | ALU | Pp | 3 | 117 | 8 | 13 | 92.0 | 80.40 | | | 80.09 | 1.15 | 1.004 | | 7 | Pp | ALU | |
| 125 | 7 | ALU | Pp | 4 | 119 | 1 | 7 | 89.0 | | | | 80.09 | 1.11 | | | 7 | Pp | ALU | |
| 126 | 7 | ALU | Pp | 4 | 168 | 2 | 1 | 79.6 | | | | 80.09 | 0.99 | | | 7 | Pp | ALU | |
| 127 | 7 | ALU | Pp | 4 | 132 | 3 | 19 | 98.8 | | | | 80.09 | 1.23 | | | 7 | Pp | ALU | |
| 128 | 7 | ALU | Pp | 4 | 155 | 4 | 1 | 79.9 | | | | 80.09 | 1.00 | | | 7 | Pp | ALU | |
| 129 | 7 | ALU | Pp | 4 | 172 | 5 | 15 | 85.2 | | | | 80.09 | 1.06 | | | 7 | Pp | ALU | |
| 130 | 7 | ALU | Pp | 4 | 108 | 6 | 23 | 72.6 | | | | 80.09 | 0.91 | | | 7 | Pp | ALU | |
| 131 | 7 | ALU | Pp | 4 | 147 | 7 | 2 | 84.5 | | | | 80.09 | 1.06 | | | 7 | Pp | ALU | |
| 132 | 7 | ALU | Pp | 4 | 130 | 8 | 3 | 85.5 | 84.39 | | | 80.09 | 1.07 | 1.054 | | 7 | Pp | ALU | |
| 133 | 7 | ALU | Pp | 5 | 143 | 1 | 12 | 93.0 | | | | 80.09 | 1.16 | | | 7 | Pp | ALU | |
| 134 | 7 | ALU | Pp | 5 | 184 | 2 | 23 | 95.3 | | | | 80.09 | 1.19 | | | 7 | Pp | ALU | |
| 135 | 7 | ALU | Pp | 5 | 153 | 3 | 18 | 83.3 | | | | 80.09 | 1.04 | | | 7 | Pp | ALU | |
| 136 | 7 | ALU | Pp | 5 | 165 | 4 | 7 | 88.3 | | | | 80.09 | 1.10 | | | 7 | Pp | ALU | |
| 137 | 7 | ALU | Pp | 5 | 134 | 5 | 7 | 111.2 | | | | 80.09 | 1.39 | | | 7 | Pp | ALU | |
| 138 | 7 | ALU | Pp | 5 | 173 | 6 | 14 | 97.2 | | | | 80.09 | 1.21 | | | 7 | Pp | ALU | |
| 139 | 7 | ALU | Pp | 5 | 116 | 7 | 15 | 91.1 | | | | 80.09 | 1.14 | | | 7 | Pp | ALU | |
| 140 | 7 | ALU | Pp | 5 | 105 | 8 | 9 | 98.3 | 94.71 | 87.78 | | 80.09 | 1.23 | 1.183 | 1.096 | 7 | Pp | ALU | 87.77 |
| 141 | 7 | ALU | Np | 1 | 195 | 1 | 25 | 79.0 | | | | 80.09 | 0.99 | | | 7 | Np | ALU | |
| 142 | 7 | ALU | Np | 1 | 158 | 2 | 9 | 52.0 | | | | 80.09 | 0.65 | | | 7 | Np | ALU | |
| 143 | 7 | ALU | Np | 1 | 164 | 5 | 24 | 47.1 | | | | 80.09 | 0.59 | | | 7 | Np | ALU | |
| 144 | 7 | ALU | Np | 1 | 142 | 6 | 15 | 55.6 | 58.43 | | | 80.09 | 0.69 | 0.729 | | 7 | Np | ALU | |
| 145 | 7 | ALU | Np | 2 | 185 | 3 | 15 | 47.6 | | | | 80.09 | 0.59 | | | 7 | Np | ALU | |
| 146 | 7 | ALU | Np | 2 | 178 | 4 | 17 | 51.1 | | | | 80.09 | 0.64 | | | 7 | Np | ALU | |
| 147 | 7 | ALU | Np | 2 | 125 | 7 | 5 | 52.6 | | | | 80.09 | 0.66 | | | 7 | Np | ALU | |
| 148 | 7 | ALU | Np | 2 | 175 | 8 | 19 | 56.4 | 51.93 | | | 80.09 | 0.70 | 0.648 | | 7 | Np | ALU | |
| 149 | 7 | ALU | Np | 3 | 106 | 1 | 20 | 70.5 | | | | 80.09 | 0.88 | | | 7 | Np | ALU | |
| 150 | 7 | ALU | Np | 3 | 139 | 2 | 21 | 43.0 | | | | 80.09 | 0.54 | | | 7 | Np | ALU | |
| 151 | 7 | ALU | Np | 3 | 191 | 5 | 16 | 78.5 | | | | 80.09 | 0.98 | | | 7 | Np | ALU | |
| 152 | 7 | ALU | Np | 3 | 159 | 6 | 10 | 63.8 | 63.95 | | | 80.09 | 0.80 | 0.798 | | 7 | Np | ALU | |
| 153 | 7 | ALU | Np | 4 | 123 | 3 | 12 | 77.7 | | | | 80.09 | 0.97 | | | 7 | Np | ALU | |
| 154 | 7 | ALU | Np | 4 | 187 | 4 | 22 | 76.6 | | | | 80.09 | 0.96 | | | 7 | Np | ALU | |
| 155 | 7 | ALU | Np | 4 | 157 | 7 | 23 | 81.5 | | | | 80.09 | 1.02 | | | 7 | Np | ALU | |
| 156 | 7 | ALU | Np | 4 | 163 | 8 | 10 | 88.1 | 80.98 | | | 80.09 | 1.10 | 1.011 | | 7 | Np | ALU | |
| 157 | 7 | ALU | Np | 5 | 113 | 2 | 16 | 59.7 | | | | 80.09 | 0.75 | | | 7 | Np | ALU | |
| 158 | 7 | ALU | Np | 5 | 180 | 4 | 21 | 72.8 | | | | 80.09 | 0.91 | | | 7 | Np | ALU | |
| 159 | 7 | ALU | Np | 5 | 198 | 6 | 5 | 72.8 | | | | 80.09 | 0.91 | | | 7 | Np | ALU | |
| 160 | 7 | ALU | Np | 5 | 182 | 8 | 6 | 70.1 | 68.85 | 64.83 | | 80.09 | 0.88 | 0.860 | 0.809 | 7 | Np | ALU | 64.83 |
| 171 | 7 | ALU | Null | NA | 129 | 1 | 21 | 66.1 | | | | 80.09 | 0.83 | | | 7 | Null | ALU | |
| 172 | 7 | ALU | Null | NA | 152 | 2 | 6 | 83.0 | | | | 80.09 | 1.04 | | | 7 | Null | ALU | |
| 173 | 7 | ALU | Null | NA | 162 | 3 | 1 | 86.3 | | | | 80.09 | 1.08 | | | 7 | Null | ALU | |
| 174 | 7 | ALU | Null | NA | 145 | 4 | 5 | 68.1 | | | | 80.09 | 0.85 | | | 7 | Null | ALU | |
| 175 | 7 | ALU | Null | NA | 109 | 5 | 9 | 83.0 | | | | 80.09 | 1.04 | | | 7 | Null | ALU | |
| 176 | 7 | ALU | Null | NA | 196 | 6 | 25 | 90.6 | | | | 80.09 | 1.13 | | | 7 | Null | ALU | |
| 177 | 7 | ALU | Null | NA | 200 | 7 | 8 | 58.7 | | | | 80.09 | 0.73 | | | 7 | Null | ALU | |
| 178 | 7 | ALU | Null | NA | 120 | 8 | 4 | 82.6 | | | | 80.09 | 1.03 | | | 7 | Null | ALU | |
| 179 | 7 | ALU | Null | NA | 136 | 1 | 18 | 59.6 | | | | 80.09 | 0.74 | | | 7 | Null | ALU | |
| 180 | 7 | ALU | Null | NA | 131 | 2 | 8 | 88.4 | | | | 80.09 | 1.10 | | | 7 | Null | ALU | |
| 181 | 7 | ALU | Null | NA | 141 | 3 | 10 | 83.3 | | | | 80.09 | 1.04 | | | 7 | Null | ALU | |
| 182 | 7 | ALU | Null | NA | 102 | 4 | 13 | 71.3 | | | | 80.09 | 0.89 | | | 7 | Null | ALU | |

# Supplemental Table S1

| | | | | | | | | | | | | |
|---|---|---|---|---|---|---|---|---|---|---|---|---|
| 64.83 | 1.36 | | | | 80.30 | 1.09 | | | | 7 | Pp | ALU |
| 64.83 | 1.28 | | | | 80.30 | 1.03 | | | | 7 | Pp | ALU |
| 64.83 | 1.44 | | | | 80.30 | 1.16 | | | | 7 | Pp | ALU |
| 64.83 | 1.07 | | | | 80.30 | 0.86 | | | | 7 | Pp | ALU |
| 64.83 | 1.42 | 1.24 | | | 80.30 | 1.15 | 1.001 | | | 7 | Pp | ALU |
| 64.83 | 1.37 | | | | 80.30 | 1.11 | | | | 7 | Pp | ALU |
| 64.83 | 1.23 | | | | 80.30 | 0.99 | | | | 7 | Pp | ALU |
| 64.83 | 1.52 | | | | 80.30 | 1.23 | | | | 7 | Pp | ALU |
| 64.83 | 1.23 | | | | 80.30 | 0.99 | | | | 7 | Pp | ALU |
| 64.83 | 1.31 | | | | 80.30 | 1.06 | | | | 7 | Pp | ALU |
| 64.83 | 1.12 | | | | 80.30 | 0.90 | | | | 7 | Pp | ALU |
| 64.83 | 1.30 | | | | 80.30 | 1.05 | | | | 7 | Pp | ALU |
| 64.83 | 1.32 | 1.30 | | | 80.30 | 1.06 | 1.051 | | | 7 | Pp | ALU |
| 64.83 | 1.43 | | | | 80.30 | 1.16 | | | | 7 | Pp | ALU |
| 64.83 | 1.47 | | | | 80.30 | 1.19 | | | | 7 | Pp | ALU |
| 64.83 | 1.28 | | | | 80.30 | 1.04 | | | | 7 | Pp | ALU |
| 64.83 | 1.36 | | | | 80.30 | 1.10 | | | | 7 | Pp | ALU |
| 64.83 | 1.72 | | | | 80.30 | 1.38 | | | | 7 | Pp | ALU |
| 64.83 | 1.50 | | Generation 7 | | 80.30 | 1.21 | | Generation 7 | | 7 | Pp | ALU |
| 64.83 | 1.41 | | Average | StDev | 80.30 | 1.13 | | Average | StDev | 7 | Pp | ALU |
| 64.83 | 1.52 | 1.46 | 1.35 | 0.09 | 80.30 | 1.22 | 1.179 | 1.093 | 0.070 | 7 | Pp | ALU |
| | | | | | | | | | | 7 | Np | ALU |
| | | | | | | | | | | 7 | Np | ALU |
| | | | | | | | | | | 7 | Np | ALU |
| | | | | | | | | | | 7 | Np | ALU |
| | | | | | | | | | | 7 | Np | ALU |
| | | | | | | | | | | 7 | Np | ALU |
| | | | | | | | | | | 7 | Np | ALU |
| | | | | | | | | | | 7 | Np | ALU |
| | | | | | | | | | | 7 | Np | ALU |
| | | | | | | | | | | 7 | Np | ALU |
| | | | | | | | | | | 7 | Np | ALU |
| | | | | | | | | | | 7 | Np | ALU |
| | | | | | | | | | | 7 | Np | ALU |
| | | | | | | | | | | 7 | Np | ALU |
| | | | | | | | | | | 7 | Np | ALU |
| | | | | | | | | | | 7 | Np | ALU |
| | | | | | | | | | | 7 | Np | ALU |
| | | | | | | | | | | 7 | Np | ALU |
| | | | | | | | | | | 7 | Np | ALU |
| | | | | | | | | | | 7 | Np | ALU |
| | | | | | | | | | | 7 | Null | ALU |
| | | | | | | | | | | 7 | Null | ALU |
| | | | | | | | | | | 7 | Null | ALU |
| | | | | | | | | | | 7 | Null | ALU |
| | | | | | | | | | | 7 | Null | ALU |
| | | | | | | | | | | 7 | Null | ALU |
| | | | | | | | | | | 7 | Null | ALU |
| | | | | | | | | | | 7 | Null | ALU |
| | | | | | | | | | | 7 | Null | ALU |
| | | | | | | | | | | 7 | Null | ALU |
| | | | | | | | | | | 7 | Null | ALU |
| | | | | | | | | | | 7 | Null | ALU |

# Supplemental Table S1

| | | | | | | | | | | | | | | | | | | | |
|---|---|---|---|---|---|---|---|---|---|---|---|---|---|---|---|---|---|---|---|
| 183 | 7 | ALU | Null | NA | 177 | 5 | 17 | 85.6 | | | | 80.09 | 1.07 | | | 7 | Null | ALU | |
| 184 | 7 | ALU | Null | NA | 169 | 6 | 17 | 110.8 | | | | 80.09 | 1.38 | | | 7 | Null | ALU | |
| 185 | 7 | ALU | Null | NA | 121 | 7 | 19 | 77.7 | | | | 80.09 | 0.97 | | | 7 | Null | ALU | |
| 186 | 7 | ALU | Null | NA | 127 | 8 | 1 | 81.7 | | | | 80.09 | 1.02 | | | 7 | Null | ALU | |
| 187 | 7 | ALU | Null | NA | 166 | 1 | 23 | 65.8 | | | | 80.09 | 0.82 | | | 7 | Null | ALU | |
| 188 | 7 | ALU | Null | NA | 194 | 2 | 12 | 78.5 | | | | 80.09 | 0.98 | | | 7 | Null | ALU | |
| 189 | 7 | ALU | Null | NA | 181 | 3 | 16 | 97.4 | | | | 80.09 | 1.22 | | | 7 | Null | ALU | |
| 190 | 7 | ALU | Null | NA | 144 | 4 | 4 | 89.8 | | | | 80.09 | 1.12 | | | 7 | Null | ALU | |
| 191 | 7 | ALU | Null | NA | 122 | 5 | 23 | 89.1 | | | | 80.09 | 1.11 | | | 7 | Null | ALU | |
| 192 | 7 | ALU | Null | NA | 176 | 6 | 6 | NA | | | | NA | NA | | | 7 | Null | ALU | |
| 193 | 7 | ALU | Null | NA | 111 | 7 | 17 | 83.4 | | | | 80.09 | 1.04 | | | 7 | Null | ALU | |
| 194 | 7 | ALU | Null | NA | 128 | 8 | 11 | 55.4 | | | | 80.09 | 0.69 | | | 7 | Null | ALU | |
| 195 | 7 | ALU | Null | NA | 197 | 2 | 2 | 73.3 | | | | 80.09 | 0.92 | | | 7 | Null | ALU | |
| 196 | 7 | ALU | Null | NA | 188 | 3 | 20 | 75.9 | | | | 80.09 | 0.95 | | | 7 | Null | ALU | |
| 197 | 7 | ALU | Null | NA | 151 | 4 | 8 | 98.8 | | | | 80.09 | 1.23 | | | 7 | Null | ALU | |
| 198 | 7 | ALU | Null | NA | 140 | 5 | 14 | 61.9 | | | | 80.09 | 0.77 | | | 7 | Null | ALU | |
| 199 | 7 | ALU | Null | NA | 135 | 7 | 9 | 103.4 | | | Grand Average | 80.09 | 1.29 | | | 7 | Null | ALU | |
| 200 | 7 | ALU | Null | NA | 179 | 8 | 14 | 79.3 | 80.30 | | 80.09 | 80.09 | 0.99 | 1.003 | | 7 | Null | ALU | 80.30 |
| 1 | 8 | SOD | Pp | 1 | 5 | 1 | 7 | 35.7 | | | | 96.14 | 0.37 | | | 8 | Pp | SOD | |
| 2 | 8 | SOD | Pp | 1 | 33 | 2 | 2 | 121.2 | | | | 96.14 | 1.26 | | | 8 | Pp | SOD | |
| 3 | 8 | SOD | Pp | 1 | 100 | 3 | 5 | NA | | | | NA | NA | | | 8 | Pp | SOD | |
| 4 | 8 | SOD | Pp | 1 | 75 | 4 | 15 | 96.0 | | | | 96.14 | 1.00 | | | 8 | Pp | SOD | |
| 5 | 8 | SOD | Pp | 1 | 4 | 5 | 10 | 119.1 | | | | 96.14 | 1.24 | | | 8 | Pp | SOD | |
| 6 | 8 | SOD | Pp | 1 | 88 | 6 | 13 | NA | | | | NA | NA | | | 8 | Pp | SOD | |
| 7 | 8 | SOD | Pp | 1 | 81 | 7 | 14 | 122.9 | | | | 96.14 | 1.28 | | | 8 | Pp | SOD | |
| 8 | 8 | SOD | Pp | 1 | 79 | 8 | 9 | 110.0 | 100.82 | | | 96.14 | 1.14 | 1.049 | | 8 | Pp | SOD | |
| 9 | 8 | SOD | Pp | 2 | 92 | 1 | 21 | 114.7 | | | | 96.14 | 1.19 | | | 8 | Pp | SOD | |
| 10 | 8 | SOD | Pp | 2 | 44 | 2 | 3 | 116.3 | | | | 96.14 | 1.21 | | | 8 | Pp | SOD | |
| 11 | 8 | SOD | Pp | 2 | 97 | 3 | 8 | 72.9 | | | | 96.14 | 0.76 | | | 8 | Pp | SOD | |
| 12 | 8 | SOD | Pp | 2 | 83 | 4 | 14 | 121.2 | | | | 96.14 | 1.26 | | | 8 | Pp | SOD | |
| 13 | 8 | SOD | Pp | 2 | 8 | 5 | 6 | NA | | | | NA | NA | | | 8 | Pp | SOD | |
| 14 | 8 | SOD | Pp | 2 | 55 | 6 | 9 | 100.8 | | | | 96.14 | 1.05 | | | 8 | Pp | SOD | |
| 15 | 8 | SOD | Pp | 2 | 64 | 7 | 17 | 126.7 | | | | 96.14 | 1.32 | | | 8 | Pp | SOD | |
| 16 | 8 | SOD | Pp | 2 | 25 | 8 | 10 | 99.4 | 107.43 | | | 96.14 | 1.03 | 1.117 | | 8 | Pp | SOD | |
| 17 | 8 | SOD | Pp | 3 | 42 | 1 | 17 | 116.8 | | | | 96.14 | 1.21 | | | 8 | Pp | SOD | |
| 18 | 8 | SOD | Pp | 3 | 96 | 2 | 16 | 110.6 | | | | 96.14 | 1.15 | | | 8 | Pp | SOD | |
| 19 | 8 | SOD | Pp | 3 | 31 | 3 | 16 | 76.5 | | | | 96.14 | 0.80 | | | 8 | Pp | SOD | |
| 20 | 8 | SOD | Pp | 3 | 39 | 4 | 8 | 110.2 | | | | 96.14 | 1.15 | | | 8 | Pp | SOD | |
| 21 | 8 | SOD | Pp | 3 | 13 | 5 | 8 | NA | | | | NA | NA | | | 8 | Pp | SOD | |
| 22 | 8 | SOD | Pp | 3 | 63 | 6 | 11 | 93.9 | | | | 96.14 | 0.98 | | | 8 | Pp | SOD | |
| 23 | 8 | SOD | Pp | 3 | 12 | 7 | 11 | 97.4 | | | | 96.14 | 1.01 | | | 8 | Pp | SOD | |
| 24 | 8 | SOD | Pp | 3 | 53 | 8 | 13 | 120.2 | 103.66 | | | 96.14 | 1.25 | 1.078 | | 8 | Pp | SOD | |
| 25 | 8 | SOD | Pp | 4 | 37 | 1 | 6 | 106.8 | | | | 96.14 | 1.11 | | | 8 | Pp | SOD | |
| 26 | 8 | SOD | Pp | 4 | 59 | 2 | 17 | 109.1 | | | | 96.14 | 1.13 | | | 8 | Pp | SOD | |
| 27 | 8 | SOD | Pp | 4 | 45 | 3 | 18 | 86.9 | | | | 96.14 | 0.90 | | | 8 | Pp | SOD | |
| 28 | 8 | SOD | Pp | 4 | 18 | 4 | 19 | 91.4 | | | | 96.14 | 0.95 | | | 8 | Pp | SOD | |
| 29 | 8 | SOD | Pp | 4 | 93 | 5 | 21 | 105.1 | | | | 96.14 | 1.09 | | | 8 | Pp | SOD | |
| 30 | 8 | SOD | Pp | 4 | 76 | 6 | 16 | 105.3 | | | | 96.14 | 1.10 | | | 8 | Pp | SOD | |
| 31 | 8 | SOD | Pp | 4 | 10 | 7 | 24 | 116.4 | | | | 96.14 | 1.21 | | | 8 | Pp | SOD | |
| 32 | 8 | SOD | Pp | 4 | 28 | 8 | 14 | 122.2 | 105.40 | | | 96.14 | 1.27 | 1.096 | | 8 | Pp | SOD | |
| 33 | 8 | SOD | Pp | 5 | 99 | 1 | 23 | 119.3 | | | | 96.14 | 1.24 | | | 8 | Pp | SOD | |
| 34 | 8 | SOD | Pp | 5 | 51 | 2 | 4 | 127.7 | | | | 96.14 | 1.33 | | | 8 | Pp | SOD | |
| 35 | 8 | SOD | Pp | 5 | 98 | 3 | 22 | 103.4 | | | | 96.14 | 1.08 | | | 8 | Pp | SOD | |

# Supplemental Table S1

| | | | | | | | | | | | | |
|---|---|---|---|---|---|---|---|---|---|---|---|---|
| | | | | | | | | | | 7 | Null | ALU |
| | | | | | | | | | | 7 | Null | ALU |
| | | | | | | | | | | 7 | Null | ALU |
| | | | | | | | | | | 7 | Null | ALU |
| | | | | | | | | | | 7 | Null | ALU |
| | | | | | | | | | | 7 | Null | ALU |
| | | | | | | | | | | 7 | Null | ALU |
| | | | | | | | | | | 7 | Null | ALU |
| | | | | | | | | | | 7 | Null | ALU |
| | | | | | | | | | | 7 | Null | ALU |
| | | | | | | | | | | 7 | Null | ALU |
| | | | | | | | | | | 7 | Null | ALU |
| | | | | | | | | | | 7 | Null | ALU |
| | | | | | | | | | | 7 | Null | ALU |
| | | | | | | | | | | 7 | Null | ALU |
| | | | | | | | | | | 7 | Null | ALU |
| | | | | | | | | | | 7 | Null | ALU |
| | | | | | | | | | | 7 | Null | ALU |
| 68.54 | 0.52 | | | | 99.37 | 0.36 | | | | 8 | Pp | SOD |
| 68.54 | 1.77 | | | | 99.37 | 1.22 | | | | 8 | Pp | SOD |
| NA | NA | | | | NA | NA | | | | 8 | Pp | SOD |
| 68.54 | 1.40 | | | | 99.37 | 0.97 | | | | 8 | Pp | SOD |
| 68.54 | 1.74 | | | | 99.37 | 1.20 | | | | 8 | Pp | SOD |
| NA | NA | | | | NA | NA | | | | 8 | Pp | SOD |
| 68.54 | 1.79 | | | | 99.37 | 1.24 | | | | 8 | Pp | SOD |
| 68.54 | 1.60 | 1.47 | | | 99.37 | 1.11 | 1.015 | | | 8 | Pp | SOD |
| 68.54 | 1.67 | | | | 99.37 | 1.15 | | | | 8 | Pp | SOD |
| 68.54 | 1.70 | | | | 99.37 | 1.17 | | | | 8 | Pp | SOD |
| 68.54 | 1.06 | | | | 99.37 | 0.73 | | | | 8 | Pp | SOD |
| 68.54 | 1.77 | | | | 99.37 | 1.22 | | | | 8 | Pp | SOD |
| NA | NA | | | | NA | NA | | | | 8 | Pp | SOD |
| 68.54 | 1.47 | | | | 99.37 | 1.01 | | | | 8 | Pp | SOD |
| 68.54 | 1.85 | | | | 99.37 | 1.28 | | | | 8 | Pp | SOD |
| 68.54 | 1.45 | 1.57 | | | 99.37 | 1.00 | 1.081 | | | 8 | Pp | SOD |
| 68.54 | 1.70 | | | | 99.37 | 1.18 | | | | 8 | Pp | SOD |
| 68.54 | 1.61 | | | | 99.37 | 1.11 | | | | 8 | Pp | SOD |
| 68.54 | 1.12 | | | | 99.37 | 0.77 | | | | 8 | Pp | SOD |
| 68.54 | 1.61 | | | | 99.37 | 1.11 | | | | 8 | Pp | SOD |
| NA | NA | | | | NA | NA | | | | 8 | Pp | SOD |
| 68.54 | 1.37 | | | | 99.37 | 0.94 | | | | 8 | Pp | SOD |
| 68.54 | 1.42 | | | | 99.37 | 0.98 | | | | 8 | Pp | SOD |
| 68.54 | 1.75 | 1.51 | | | 99.37 | 1.21 | 1.043 | | | 8 | Pp | SOD |
| 68.54 | 1.56 | | | | 99.37 | 1.07 | | | | 8 | Pp | SOD |
| 68.54 | 1.59 | | | | 99.37 | 1.10 | | | | 8 | Pp | SOD |
| 68.54 | 1.27 | | | | 99.37 | 0.87 | | | | 8 | Pp | SOD |
| 68.54 | 1.33 | | | | 99.37 | 0.92 | | | | 8 | Pp | SOD |
| 68.54 | 1.53 | | | | 99.37 | 1.06 | | | | 8 | Pp | SOD |
| 68.54 | 1.54 | | | | 99.37 | 1.06 | | | | 8 | Pp | SOD |
| 68.54 | 1.70 | | | | 99.37 | 1.17 | | | | 8 | Pp | SOD |
| 68.54 | 1.78 | 1.54 | | | 99.37 | 1.23 | 1.061 | | | 8 | Pp | SOD |
| 68.54 | 1.74 | | | | 99.37 | 1.20 | | | | 8 | Pp | SOD |
| 68.54 | 1.86 | | | | 99.37 | 1.29 | | | | 8 | Pp | SOD |
| 68.54 | 1.51 | | | | 99.37 | 1.04 | | | | 8 | Pp | SOD |

# Supplemental Table S1

| | | | | | | | | | | | | | | | | | | | |
|---|---|---|---|---|---|---|---|---|---|---|---|---|---|---|---|---|---|---|---|
| 36 | 8 | SOD | Pp | 5 | 67 | 4 | 3 | 136.0 | | | | 96.14 | 1.41 | | | 8 | Pp | SOD | |
| 37 | 8 | SOD | Pp | 5 | 36 | 5 | 12 | 146.2 | | | | 96.14 | 1.52 | | | 8 | Pp | SOD | |
| 38 | 8 | SOD | Pp | 5 | 21 | 6 | 7 | 116.7 | | | | 96.14 | 1.21 | | | 8 | Pp | SOD | |
| 39 | 8 | SOD | Pp | 5 | 66 | 7 | 22 | 118.2 | | | | 96.14 | 1.23 | | | 8 | Pp | SOD | |
| 40 | 8 | SOD | Pp | 5 | 70 | 8 | 5 | 98.2 | 120.71 | 107.60 | | 96.14 | 1.02 | 1.256 | 1.119 | 8 | Pp | SOD | 108.09 |
| 41 | 8 | SOD | Np | 1 | 29 | 1 | 4 | 1.5 | | | | 96.14 | 0.02 | | | 8 | Np | SOD | |
| 42 | 8 | SOD | Np | 1 | 41 | 2 | 23 | 75.1 | | | | 96.14 | 0.78 | | | 8 | Np | SOD | |
| 43 | 8 | SOD | Np | 1 | 71 | 5 | 15 | 99.4 | | | | 96.14 | 1.03 | | | 8 | Np | SOD | |
| 44 | 8 | SOD | Np | 1 | 52 | 6 | 3 | 93.8 | 67.45 | | | 96.14 | 0.98 | 0.702 | | 8 | Np | SOD | |
| 45 | 8 | SOD | Np | 2 | 61 | 3 | 9 | 72.0 | | | | 96.14 | 0.75 | | | 8 | Np | SOD | |
| 46 | 8 | SOD | Np | 2 | 22 | 4 | 13 | 98.2 | | | | 96.14 | 1.02 | | | 8 | Np | SOD | |
| 47 | 8 | SOD | Np | 2 | 50 | 7 | 9 | 65.4 | | | | 96.14 | 0.68 | | | 8 | Np | SOD | |
| 48 | 8 | SOD | Np | 2 | 16 | 8 | 18 | 64.4 | 75.00 | | | 96.14 | 0.67 | 0.780 | | 8 | Np | SOD | |
| 49 | 8 | SOD | Np | 3 | 72 | 1 | 12 | NA | | | | NA | NA | | | 8 | Np | SOD | |
| 50 | 8 | SOD | Np | 3 | 82 | 2 | 25 | 90.8 | | | | 96.14 | 0.94 | | | 8 | Np | SOD | |
| 51 | 8 | SOD | Np | 3 | 1 | 5 | 20 | 111.1 | | | | 96.14 | 1.16 | | | 8 | Np | SOD | |
| 52 | 8 | SOD | Np | 3 | 87 | 6 | 4 | 119.9 | 107.27 | | | 96.14 | 1.25 | 1.116 | | 8 | Np | SOD | |
| 53 | 8 | SOD | Np | 4 | 94 | 3 | 20 | 69.5 | | | | 96.14 | 0.72 | | | 8 | Np | SOD | |
| 54 | 8 | SOD | Np | 4 | 68 | 4 | 9 | 107.0 | | | | 96.14 | 1.11 | | | 8 | Np | SOD | |
| 55 | 8 | SOD | Np | 4 | 62 | 7 | 10 | 0.8 | | | | 96.14 | 0.01 | | | 8 | Np | SOD | |
| 56 | 8 | SOD | Np | 4 | 49 | 8 | 23 | 89.8 | 66.78 | | | 96.14 | 0.93 | 0.695 | | 8 | Np | SOD | |
| 57 | 8 | SOD | Np | 5 | 17 | 1 | 25 | 1.1 | | | | 96.14 | 0.01 | | | 8 | Np | SOD | |
| 58 | 8 | SOD | Np | 5 | 40 | 3 | 15 | 65.1 | | | | 96.14 | 0.68 | | | 8 | Np | SOD | |
| 59 | 8 | SOD | Np | 5 | 24 | 5 | 9 | 1.1 | | | | 96.14 | 0.01 | | | 8 | Np | SOD | |
| 60 | 8 | SOD | Np | 5 | 57 | 7 | 25 | 76.2 | 35.88 | 70.47 | | 96.14 | 0.79 | 0.373 | 0.733 | 8 | Np | SOD | 68.54 |
| 71 | 8 | SOD | Null | NA | 74 | 1 | 2 | 81.3 | | | | 96.14 | 0.85 | | | 8 | Null | SOD | |
| 72 | 8 | SOD | Null | NA | 60 | 2 | 22 | 111.9 | | | | 96.14 | 1.16 | | | 8 | Null | SOD | |
| 73 | 8 | SOD | Null | NA | 69 | 3 | 13 | 82.6 | | | | 96.14 | 0.86 | | | 8 | Null | SOD | |
| 74 | 8 | SOD | Null | NA | 11 | 4 | 6 | 86.1 | | | | 96.14 | 0.90 | | | 8 | Null | SOD | |
| 75 | 8 | SOD | Null | NA | 46 | 5 | 14 | 70.6 | | | | 96.14 | 0.73 | | | 8 | Null | SOD | |
| 76 | 8 | SOD | Null | NA | 48 | 6 | 23 | 93.2 | | | | 96.14 | 0.97 | | | 8 | Null | SOD | |
| 77 | 8 | SOD | Null | NA | 26 | 7 | 13 | 108.9 | | | | 96.14 | 1.13 | | | 8 | Null | SOD | |
| 78 | 8 | SOD | Null | NA | 6 | 8 | 21 | 100.3 | | | | 96.14 | 1.04 | | | 8 | Null | SOD | |
| 79 | 8 | SOD | Null | NA | 35 | 1 | 14 | 119.0 | | | | 96.14 | 1.24 | | | 8 | Null | SOD | |
| 80 | 8 | SOD | Null | NA | 90 | 2 | 15 | 96.6 | | | | 96.14 | 1.00 | | | 8 | Null | SOD | |
| 81 | 8 | SOD | Null | NA | 23 | 3 | 3 | 92.5 | | | | 96.14 | 0.96 | | | 8 | Null | SOD | |
| 82 | 8 | SOD | Null | NA | 65 | 4 | 20 | 72.5 | | | | 96.14 | 0.75 | | | 8 | Null | SOD | |
| 83 | 8 | SOD | Null | NA | 9 | 5 | 2 | 86.2 | | | | 96.14 | 0.90 | | | 8 | Null | SOD | |
| 84 | 8 | SOD | Null | NA | 77 | 6 | 10 | 102.1 | | | | 96.14 | 1.06 | | | 8 | Null | SOD | |
| 85 | 8 | SOD | Null | NA | 58 | 7 | 16 | 108.4 | | | | 96.14 | 1.13 | | | 8 | Null | SOD | |
| 86 | 8 | SOD | Null | NA | 38 | 8 | 25 | 105.1 | | | | 96.14 | 1.09 | | | 8 | Null | SOD | |
| 87 | 8 | SOD | Null | NA | 30 | 1 | 22 | NA | | | | NA | NA | | | 8 | Null | SOD | |
| 88 | 8 | SOD | Null | NA | 32 | 2 | 14 | 110.1 | | | | 96.14 | 1.15 | | | 8 | Null | SOD | |
| 89 | 8 | SOD | Null | NA | 43 | 3 | 14 | 107.6 | | | | 96.14 | 1.12 | | | 8 | Null | SOD | |
| 90 | 8 | SOD | Null | NA | 47 | 4 | 12 | 91.2 | | | | 96.14 | 0.95 | | | 8 | Null | SOD | |
| 91 | 8 | SOD | Null | NA | 15 | 5 | 11 | 114.4 | | | | 96.14 | 1.19 | | | 8 | Null | SOD | |
| 92 | 8 | SOD | Null | NA | 78 | 6 | 1 | 99.1 | | | | 96.14 | 1.03 | | | 8 | Null | SOD | |
| 93 | 8 | SOD | Null | NA | 73 | 7 | 3 | 108.0 | | | | 96.14 | 1.12 | | | 8 | Null | SOD | |
| 94 | 8 | SOD | Null | NA | 54 | 8 | 2 | 100.7 | | | | 96.14 | 1.05 | | | 8 | Null | SOD | |
| 95 | 8 | SOD | Null | NA | 80 | 2 | 11 | 97.0 | | | | 96.14 | 1.01 | | | 8 | Null | SOD | |
| 96 | 8 | SOD | Null | NA | 20 | 3 | 25 | 95.8 | | | | 96.14 | 1.00 | | | 8 | Null | SOD | |
| 97 | 8 | SOD | Null | NA | 95 | 4 | 23 | 119.3 | | | | 96.14 | 1.24 | | | 8 | Null | SOD | |
| 98 | 8 | SOD | Null | NA | 14 | 5 | 24 | 115.4 | | | | 96.14 | 1.20 | | | 8 | Null | SOD | |

# Supplemental Table S1

| | | | | | | | | | | | | |
|---|---|---|---|---|---|---|---|---|---|---|---|---|
| 68.54 | 1.98 | | | | 99.37 | 1.37 | | | | 8 | Pp | SOD |
| 68.54 | 2.13 | | | | 99.37 | 1.47 | | | | 8 | Pp | SOD |
| 68.54 | 1.70 | | Generation 8 | | 99.37 | 1.17 | | Generation 8 | | 8 | Pp | SOD |
| 68.54 | 1.72 | | Average | StDev | 99.37 | 1.19 | | Average | StDev | 8 | Pp | SOD |
| 68.54 | 1.43 | 1.76 | 1.57 | 0.11 | 99.37 | 0.99 | 1.215 | 1.083 | 0.078 | 8 | Pp | SOD |
| | | | | | | | | | | 8 | Np | SOD |
| | | | | | | | | | | 8 | Np | SOD |
| | | | | | | | | | | 8 | Np | SOD |
| | | | | | | | | | | 8 | Np | SOD |
| | | | | | | | | | | 8 | Np | SOD |
| | | | | | | | | | | 8 | Np | SOD |
| | | | | | | | | | | 8 | Np | SOD |
| | | | | | | | | | | 8 | Np | SOD |
| | | | | | | | | | | 8 | Np | SOD |
| | | | | | | | | | | 8 | Np | SOD |
| | | | | | | | | | | 8 | Np | SOD |
| | | | | | | | | | | 8 | Np | SOD |
| | | | | | | | | | | 8 | Np | SOD |
| | | | | | | | | | | 8 | Np | SOD |
| | | | | | | | | | | 8 | Np | SOD |
| | | | | | | | | | | 8 | Np | SOD |
| | | | | | | | | | | 8 | Np | SOD |
| | | | | | | | | | | 8 | Np | SOD |
| | | | | | | | | | | 8 | Np | SOD |
| | | | | | | | | | | 8 | Np | SOD |
| | | | | | | | | | | 8 | Null | SOD |
| | | | | | | | | | | 8 | Null | SOD |
| | | | | | | | | | | 8 | Null | SOD |
| | | | | | | | | | | 8 | Null | SOD |
| | | | | | | | | | | 8 | Null | SOD |
| | | | | | | | | | | 8 | Null | SOD |
| | | | | | | | | | | 8 | Null | SOD |
| | | | | | | | | | | 8 | Null | SOD |
| | | | | | | | | | | 8 | Null | SOD |
| | | | | | | | | | | 8 | Null | SOD |
| | | | | | | | | | | 8 | Null | SOD |
| | | | | | | | | | | 8 | Null | SOD |
| | | | | | | | | | | 8 | Null | SOD |
| | | | | | | | | | | 8 | Null | SOD |
| | | | | | | | | | | 8 | Null | SOD |
| | | | | | | | | | | 8 | Null | SOD |
| | | | | | | | | | | 8 | Null | SOD |
| | | | | | | | | | | 8 | Null | SOD |
| | | | | | | | | | | 8 | Null | SOD |
| | | | | | | | | | | 8 | Null | SOD |
| | | | | | | | | | | 8 | Null | SOD |
| | | | | | | | | | | 8 | Null | SOD |
| | | | | | | | | | | 8 | Null | SOD |
| | | | | | | | | | | 8 | Null | SOD |
| | | | | | | | | | | 8 | Null | SOD |
| | | | | | | | | | | 8 | Null | SOD |
| | | | | | | | | | | 8 | Null | SOD |
| | | | | | | | | | | 8 | Null | SOD |

# Supplemental Table S1

| | | | | | | | | | | | | | | | | | | | |
|---|---|---|---|---|---|---|---|---|---|---|---|---|---|---|---|---|---|---|---|
| 99 | 8 | SOD | Null | NA | 3 | 7 | 15 | 80.6 | | | Grand Average | 96.14 | 0.84 | | | 8 | Null | SOD | |
| 100 | 8 | SOD | Null | NA | 86 | 8 | 7 | 125.3 | 99.37 | | 96.14 | 96.14 | 1.30 | 1.034 | | 8 | Null | SOD | 99.37 |
| 101 | 8 | ALU | Pp | 1 | 179 | 1 | 3 | 164.6 | | | | 125.44 | 1.31 | | | 8 | Pp | ALU | |
| 102 | 8 | ALU | Pp | 1 | 171 | 2 | 24 | 171.8 | | | | 125.44 | 1.37 | | | 8 | Pp | ALU | |
| 103 | 8 | ALU | Pp | 1 | 166 | 3 | 11 | 123.7 | | | | 125.44 | 0.99 | | | 8 | Pp | ALU | |
| 104 | 8 | ALU | Pp | 1 | 177 | 4 | 5 | 153.3 | | | | 125.44 | 1.22 | | | 8 | Pp | ALU | |
| 105 | 8 | ALU | Pp | 1 | 114 | 5 | 16 | NA | | | | NA | NA | | | 8 | Pp | ALU | |
| 106 | 8 | ALU | Pp | 1 | 162 | 6 | 14 | 162.2 | | | | 125.44 | 1.29 | | | 8 | Pp | ALU | |
| 107 | 8 | ALU | Pp | 1 | 138 | 7 | 23 | 128.1 | | | | 125.44 | 1.02 | | | 8 | Pp | ALU | |
| 108 | 8 | ALU | Pp | 1 | 144 | 8 | 11 | 133.0 | 148.10 | | | 125.44 | 1.06 | 1.181 | | 8 | Pp | ALU | |
| 109 | 8 | ALU | Pp | 2 | 116 | 1 | 18 | 152.0 | | | | 125.44 | 1.21 | | | 8 | Pp | ALU | |
| 110 | 8 | ALU | Pp | 2 | 189 | 2 | 19 | 143.0 | | | | 125.44 | 1.14 | | | 8 | Pp | ALU | |
| 111 | 8 | ALU | Pp | 2 | 115 | 3 | 12 | 121.1 | | | | 125.44 | 0.97 | | | 8 | Pp | ALU | |
| 112 | 8 | ALU | Pp | 2 | 174 | 4 | 22 | 143.1 | | | | 125.44 | 1.14 | | | 8 | Pp | ALU | |
| 113 | 8 | ALU | Pp | 2 | 194 | 5 | 4 | 129.2 | | | | 125.44 | 1.03 | | | 8 | Pp | ALU | |
| 114 | 8 | ALU | Pp | 2 | 106 | 6 | 19 | 145.5 | | | | 125.44 | 1.16 | | | 8 | Pp | ALU | |
| 115 | 8 | ALU | Pp | 2 | 185 | 7 | 4 | 148.5 | | | | 125.44 | 1.18 | | | 8 | Pp | ALU | |
| 116 | 8 | ALU | Pp | 2 | 196 | 8 | 16 | 130.1 | 139.06 | | | 125.44 | 1.04 | 1.109 | | 8 | Pp | ALU | |
| 117 | 8 | ALU | Pp | 3 | 182 | 1 | 24 | 115.3 | | | | 125.44 | 0.92 | | | 8 | Pp | ALU | |
| 118 | 8 | ALU | Pp | 3 | 170 | 2 | 9 | 149.0 | | | | 125.44 | 1.19 | | | 8 | Pp | ALU | |
| 119 | 8 | ALU | Pp | 3 | 130 | 3 | 4 | 138.2 | | | | 125.44 | 1.10 | | | 8 | Pp | ALU | |
| 120 | 8 | ALU | Pp | 3 | 186 | 4 | 16 | 149.9 | | | | 125.44 | 1.20 | | | 8 | Pp | ALU | |
| 121 | 8 | ALU | Pp | 3 | 145 | 5 | 18 | 136.5 | | | | 125.44 | 1.09 | | | 8 | Pp | ALU | |
| 122 | 8 | ALU | Pp | 3 | 140 | 6 | 22 | 138.8 | | | | 125.44 | 1.11 | | | 8 | Pp | ALU | |
| 123 | 8 | ALU | Pp | 3 | 190 | 7 | 20 | 129.0 | | | | 125.44 | 1.03 | | | 8 | Pp | ALU | |
| 124 | 8 | ALU | Pp | 3 | 118 | 8 | 12 | 100.7 | 132.18 | | | 125.44 | 0.80 | 1.054 | | 8 | Pp | ALU | |
| 125 | 8 | ALU | Pp | 4 | 131 | 1 | 20 | 143.3 | | | | 125.44 | 1.14 | | | 8 | Pp | ALU | |
| 126 | 8 | ALU | Pp | 4 | 175 | 2 | 5 | 121.8 | | | | 125.44 | 0.97 | | | 8 | Pp | ALU | |
| 127 | 8 | ALU | Pp | 4 | 164 | 3 | 2 | 143.1 | | | | 125.44 | 1.14 | | | 8 | Pp | ALU | |
| 128 | 8 | ALU | Pp | 4 | 111 | 4 | 4 | 162.5 | | | | 125.44 | 1.30 | | | 8 | Pp | ALU | |
| 129 | 8 | ALU | Pp | 4 | 103 | 5 | 1 | 134.5 | | | | 125.44 | 1.07 | | | 8 | Pp | ALU | |
| 130 | 8 | ALU | Pp | 4 | 119 | 6 | 18 | 170.3 | | | | 125.44 | 1.36 | | | 8 | Pp | ALU | |
| 131 | 8 | ALU | Pp | 4 | 153 | 7 | 21 | 139.1 | | | | 125.44 | 1.11 | | | 8 | Pp | ALU | |
| 132 | 8 | ALU | Pp | 4 | 133 | 8 | 15 | NA | 144.94 | | | NA | NA | 1.155 | | 8 | Pp | ALU | |
| 133 | 8 | ALU | Pp | 5 | 135 | 1 | 10 | 156.8 | | | | 125.44 | 1.25 | | | 8 | Pp | ALU | |
| 134 | 8 | ALU | Pp | 5 | 110 | 2 | 7 | 172.4 | | | | 125.44 | 1.37 | | | 8 | Pp | ALU | |
| 135 | 8 | ALU | Pp | 5 | 134 | 3 | 21 | 162.0 | | | | 125.44 | 1.29 | | | 8 | Pp | ALU | |
| 136 | 8 | ALU | Pp | 5 | 125 | 4 | 1 | 157.6 | | | | 125.44 | 1.26 | | | 8 | Pp | ALU | |
| 137 | 8 | ALU | Pp | 5 | 143 | 5 | 17 | NA | | | | NA | NA | | | 8 | Pp | ALU | |
| 138 | 8 | ALU | Pp | 5 | 122 | 6 | 24 | 149.1 | | | | 125.44 | 1.19 | | | 8 | Pp | ALU | |
| 139 | 8 | ALU | Pp | 5 | 107 | 7 | 19 | 141.3 | | | | 125.44 | 1.13 | | | 8 | Pp | ALU | |
| 140 | 8 | ALU | Pp | 5 | 137 | 8 | 4 | NA | 156.53 | 144.16 | | NA | NA | 1.248 | 1.149 | 8 | Pp | ALU | 143.34 |
| 141 | 8 | ALU | Np | 1 | 188 | 1 | 15 | 93.4 | | | | 125.44 | 0.74 | | | 8 | Np | ALU | |
| 142 | 8 | ALU | Np | 1 | 113 | 2 | 21 | 82.6 | | | | 125.44 | 0.66 | | | 8 | Np | ALU | |
| 143 | 8 | ALU | Np | 1 | 173 | 5 | 19 | 90.5 | | | | 125.44 | 0.72 | | | 8 | Np | ALU | |
| 144 | 8 | ALU | Np | 1 | 161 | 6 | 25 | 90.8 | 89.33 | | | 125.44 | 0.72 | 0.712 | | 8 | Np | ALU | |
| 145 | 8 | ALU | Np | 2 | 124 | 3 | 6 | 89.5 | | | | 125.44 | 0.71 | | | 8 | Np | ALU | |
| 146 | 8 | ALU | Np | 2 | 157 | 4 | 11 | 133.7 | | | | 125.44 | 1.07 | | | 8 | Np | ALU | |
| 147 | 8 | ALU | Np | 2 | 109 | 7 | 2 | 138.4 | | | | 125.44 | 1.10 | | | 8 | Np | ALU | |
| 148 | 8 | ALU | Np | 2 | 121 | 8 | 6 | 111.3 | 118.23 | | | 125.44 | 0.89 | 0.942 | | 8 | Np | ALU | |
| 149 | 8 | ALU | Np | 3 | 148 | 1 | 8 | 83.3 | | | | 125.44 | 0.66 | | | 8 | Np | ALU | |
| 150 | 8 | ALU | Np | 3 | 123 | 2 | 20 | 85.9 | | | | 125.44 | 0.68 | | | 8 | Np | ALU | |
| 151 | 8 | ALU | Np | 3 | 104 | 5 | 7 | 84.8 | | | | 125.44 | 0.68 | | | 8 | Np | ALU | |

## Supplemental Table S1

| | | | | | | | | | | | | |
|---|---|---|---|---|---|---|---|---|---|---|---|---|
| | | | | | | | | | | 8 | Null | SOD |
| | | | | | | | | | | 8 | Null | SOD |
| 90.19 | 1.83 | | | | 126.31 | 1.30 | | | | 8 | Pp | ALU |
| 90.19 | 1.90 | | | | 126.31 | 1.36 | | | | 8 | Pp | ALU |
| 90.19 | 1.37 | | | | 126.31 | 0.98 | | | | 8 | Pp | ALU |
| 90.19 | 1.70 | | | | 126.31 | 1.21 | | | | 8 | Pp | ALU |
| NA | NA | | | | NA | NA | | | | 8 | Pp | ALU |
| 90.19 | 1.80 | | | | 126.31 | 1.28 | | | | 8 | Pp | ALU |
| 90.19 | 1.42 | | | | 126.31 | 1.01 | | | | 8 | Pp | ALU |
| 90.19 | 1.47 | 1.64 | | | 126.31 | 1.05 | 1.173 | | | 8 | Pp | ALU |
| 90.19 | 1.69 | | | | 126.31 | 1.20 | | | | 8 | Pp | ALU |
| 90.19 | 1.59 | | | | 126.31 | 1.13 | | | | 8 | Pp | ALU |
| 90.19 | 1.34 | | | | 126.31 | 0.96 | | | | 8 | Pp | ALU |
| 90.19 | 1.59 | | | | 126.31 | 1.13 | | | | 8 | Pp | ALU |
| 90.19 | 1.43 | | | | 126.31 | 1.02 | | | | 8 | Pp | ALU |
| 90.19 | 1.61 | | | | 126.31 | 1.15 | | | | 8 | Pp | ALU |
| 90.19 | 1.65 | | | | 126.31 | 1.18 | | | | 8 | Pp | ALU |
| 90.19 | 1.44 | 1.54 | | | 126.31 | 1.03 | 1.101 | | | 8 | Pp | ALU |
| 90.19 | 1.28 | | | | 126.31 | 0.91 | | | | 8 | Pp | ALU |
| 90.19 | 1.65 | | | | 126.31 | 1.18 | | | | 8 | Pp | ALU |
| 90.19 | 1.53 | | | | 126.31 | 1.09 | | | | 8 | Pp | ALU |
| 90.19 | 1.66 | | | | 126.31 | 1.19 | | | | 8 | Pp | ALU |
| 90.19 | 1.51 | | | | 126.31 | 1.08 | | | | 8 | Pp | ALU |
| 90.19 | 1.54 | | | | 126.31 | 1.10 | | | | 8 | Pp | ALU |
| 90.19 | 1.43 | | | | 126.31 | 1.02 | | | | 8 | Pp | ALU |
| 90.19 | 1.12 | 1.47 | | | 126.31 | 0.80 | 1.046 | | | 8 | Pp | ALU |
| 90.19 | 1.59 | | | | 126.31 | 1.13 | | | | 8 | Pp | ALU |
| 90.19 | 1.35 | | | | 126.31 | 0.96 | | | | 8 | Pp | ALU |
| 90.19 | 1.59 | | | | 126.31 | 1.13 | | | | 8 | Pp | ALU |
| 90.19 | 1.80 | | | | 126.31 | 1.29 | | | | 8 | Pp | ALU |
| 90.19 | 1.49 | | | | 126.31 | 1.06 | | | | 8 | Pp | ALU |
| 90.19 | 1.89 | | | | 126.31 | 1.35 | | | | 8 | Pp | ALU |
| 90.19 | 1.54 | | | | 126.31 | 1.10 | | | | 8 | Pp | ALU |
| NA | NA | 1.61 | | | NA | NA | 1.148 | | | 8 | Pp | ALU |
| 90.19 | 1.74 | | | | 126.31 | 1.24 | | | | 8 | Pp | ALU |
| 90.19 | 1.91 | | | | 126.31 | 1.36 | | | | 8 | Pp | ALU |
| 90.19 | 1.80 | | | | 126.31 | 1.28 | | | | 8 | Pp | ALU |
| 90.19 | 1.75 | | | | 126.31 | 1.25 | | | | 8 | Pp | ALU |
| NA | NA | | | | NA | NA | | | | 8 | Pp | ALU |
| 90.19 | 1.65 | | Generation 8 | | 126.31 | 1.18 | | Generation 8 | | 8 | Pp | ALU |
| 90.19 | 1.57 | | Average | StDev | 126.31 | 1.12 | | Average | StDev | 8 | Pp | ALU |
| NA | NA | 1.74 | 1.60 | 0.10 | NA | NA | 1.239 | 1.141 | 0.073 | 8 | Pp | ALU |
| | | | | | | | | | | 8 | Np | ALU |
| | | | | | | | | | | 8 | Np | ALU |
| | | | | | | | | | | 8 | Np | ALU |
| | | | | | | | | | | 8 | Np | ALU |
| | | | | | | | | | | 8 | Np | ALU |
| | | | | | | | | | | 8 | Np | ALU |
| | | | | | | | | | | 8 | Np | ALU |
| | | | | | | | | | | 8 | Np | ALU |
| | | | | | | | | | | 8 | Np | ALU |
| | | | | | | | | | | 8 | Np | ALU |
| | | | | | | | | | | 8 | Np | ALU |

# Supplemental Table S1

| | | | | | | | | | | | | | | | | | | | |
|---|---|---|---|---|---|---|---|---|---|---|---|---|---|---|---|---|---|---|---|
| 152 | 8 | ALU | Np | 3 | 158 | 6 | 17 | 82.3 | 84.08 | | | 125.44 | 0.66 | 0.670 | | 8 | Np | ALU | |
| 153 | 8 | ALU | Np | 4 | 191 | 3 | 19 | 106.4 | | | | 125.44 | 0.85 | | | 8 | Np | ALU | |
| 154 | 8 | ALU | Np | 4 | 165 | 4 | 7 | 61.3 | | | | 125.44 | 0.49 | | | 8 | Np | ALU | |
| 155 | 8 | ALU | Np | 4 | 129 | 7 | 6 | 83.1 | | | | 125.44 | 0.66 | | | 8 | Np | ALU | |
| 156 | 8 | ALU | Np | 4 | 167 | 8 | 1 | NA | 83.60 | | | NA | NA | 0.666 | | 8 | Np | ALU | |
| 157 | 8 | ALU | Np | 5 | 141 | 2 | 6 | 77.4 | | | | 125.44 | 0.62 | | | 8 | Np | ALU | |
| 158 | 8 | ALU | Np | 5 | 126 | 4 | 17 | 102.0 | | | | 125.44 | 0.81 | | | 8 | Np | ALU | |
| 159 | 8 | ALU | Np | 5 | 151 | 6 | 5 | 63.0 | | | | 125.44 | 0.50 | | | 8 | Np | ALU | |
| 160 | 8 | ALU | Np | 5 | 193 | 8 | 8 | 53.9 | 74.08 | 89.86 | | 125.44 | 0.43 | 0.591 | 0.716 | 8 | Np | ALU | 90.19 |
| 171 | 8 | ALU | Null | NA | 181 | 1 | 16 | 119.4 | | | | 125.44 | 0.95 | | | 8 | Null | ALU | |
| 172 | 8 | ALU | Null | NA | 105 | 2 | 13 | NA | | | | NA | NA | | | 8 | Null | ALU | |
| 173 | 8 | ALU | Null | NA | 149 | 3 | 10 | 125.8 | | | | 125.44 | 1.00 | | | 8 | Null | ALU | |
| 174 | 8 | ALU | Null | NA | 163 | 4 | 18 | 128.2 | | | | 125.44 | 1.02 | | | 8 | Null | ALU | |
| 175 | 8 | ALU | Null | NA | 198 | 5 | 23 | 139.4 | | | | 125.44 | 1.11 | | | 8 | Null | ALU | |
| 176 | 8 | ALU | Null | NA | 187 | 6 | 20 | 128.8 | | | | 125.44 | 1.03 | | | 8 | Null | ALU | |
| 177 | 8 | ALU | Null | NA | 147 | 7 | 12 | 139.7 | | | | 125.44 | 1.11 | | | 8 | Null | ALU | |
| 178 | 8 | ALU | Null | NA | 127 | 8 | 17 | 95.0 | | | | 125.44 | 0.76 | | | 8 | Null | ALU | |
| 179 | 8 | ALU | Null | NA | 132 | 1 | 9 | 109.9 | | | | 125.44 | 0.88 | | | 8 | Null | ALU | |
| 180 | 8 | ALU | Null | NA | 197 | 2 | 8 | 111.1 | | | | 125.44 | 0.89 | | | 8 | Null | ALU | |
| 181 | 8 | ALU | Null | NA | 180 | 3 | 7 | 119.6 | | | | 125.44 | 0.95 | | | 8 | Null | ALU | |
| 182 | 8 | ALU | Null | NA | 117 | 4 | 21 | 122.8 | | | | 125.44 | 0.98 | | | 8 | Null | ALU | |
| 183 | 8 | ALU | Null | NA | 101 | 5 | 13 | 144.0 | | | | 125.44 | 1.15 | | | 8 | Null | ALU | |
| 184 | 8 | ALU | Null | NA | 108 | 6 | 21 | 144.1 | | | | 125.44 | 1.15 | | | 8 | Null | ALU | |
| 185 | 8 | ALU | Null | NA | 128 | 7 | 7 | 151.3 | | | | 125.44 | 1.21 | | | 8 | Null | ALU | |
| 186 | 8 | ALU | Null | NA | 184 | 8 | 20 | 110.9 | | | | 125.44 | 0.88 | | | 8 | Null | ALU | |
| 187 | 8 | ALU | Null | NA | 176 | 1 | 5 | 131.3 | | | | 125.44 | 1.05 | | | 8 | Null | ALU | |
| 188 | 8 | ALU | Null | NA | 139 | 2 | 10 | 125.0 | | | | 125.44 | 1.00 | | | 8 | Null | ALU | |
| 189 | 8 | ALU | Null | NA | 160 | 3 | 1 | 131.6 | | | | 125.44 | 1.05 | | | 8 | Null | ALU | |
| 190 | 8 | ALU | Null | NA | 168 | 4 | 25 | 118.6 | | | | 125.44 | 0.95 | | | 8 | Null | ALU | |
| 191 | 8 | ALU | Null | NA | 159 | 5 | 3 | 136.1 | | | | 125.44 | 1.08 | | | 8 | Null | ALU | |
| 192 | 8 | ALU | Null | NA | 199 | 6 | 6 | 117.4 | | | | 125.44 | 0.94 | | | 8 | Null | ALU | |
| 193 | 8 | ALU | Null | NA | 183 | 7 | 8 | 135.4 | | | | 125.44 | 1.08 | | | 8 | Null | ALU | |
| 194 | 8 | ALU | Null | NA | 146 | 8 | 22 | 136.1 | | | | 125.44 | 1.08 | | | 8 | Null | ALU | |
| 195 | 8 | ALU | Null | NA | 150 | 2 | 18 | 92.7 | | | | 125.44 | 0.74 | | | 8 | Null | ALU | |
| 196 | 8 | ALU | Null | NA | 152 | 3 | 24 | 117.2 | | | | 125.44 | 0.93 | | | 8 | Null | ALU | |
| 197 | 8 | ALU | Null | NA | 102 | 4 | 2 | 123.9 | | | | 125.44 | 0.99 | | | 8 | Null | ALU | |
| 198 | 8 | ALU | Null | NA | 192 | 5 | 25 | 140.4 | | | | 125.44 | 1.12 | | | 8 | Null | ALU | |
| 199 | 8 | ALU | Null | NA | 200 | 6 | 2 | 148.9 | | | Grand Average | 125.44 | 1.19 | | | 8 | Null | ALU | |
| 200 | 8 | ALU | Null | NA | 156 | 8 | 19 | 118.3 | 126.31 | | 125.44 | 125.44 | 0.94 | 1.007 | | 8 | Null | ALU | 126.31 |

## Supplemental Table S1

| | | | | | | | | | | | | |
|---|---|---|---|---|---|---|---|---|---|---|---|---|
| | | | | | | | | | | 8 | Np | ALU |
| | | | | | | | | | | 8 | Np | ALU |
| | | | | | | | | | | 8 | Np | ALU |
| | | | | | | | | | | 8 | Np | ALU |
| | | | | | | | | | | 8 | Np | ALU |
| | | | | | | | | | | 8 | Np | ALU |
| | | | | | | | | | | 8 | Np | ALU |
| | | | | | | | | | | 8 | Np | ALU |
| | | | | | | | | | | 8 | Np | ALU |
| | | | | | | | | | | 8 | Null | ALU |
| | | | | | | | | | | 8 | Null | ALU |
| | | | | | | | | | | 8 | Null | ALU |
| | | | | | | | | | | 8 | Null | ALU |
| | | | | | | | | | | 8 | Null | ALU |
| | | | | | | | | | | 8 | Null | ALU |
| | | | | | | | | | | 8 | Null | ALU |
| | | | | | | | | | | 8 | Null | ALU |
| | | | | | | | | | | 8 | Null | ALU |
| | | | | | | | | | | 8 | Null | ALU |
| | | | | | | | | | | 8 | Null | ALU |
| | | | | | | | | | | 8 | Null | ALU |
| | | | | | | | | | | 8 | Null | ALU |
| | | | | | | | | | | 8 | Null | ALU |
| | | | | | | | | | | 8 | Null | ALU |
| | | | | | | | | | | 8 | Null | ALU |
| | | | | | | | | | | 8 | Null | ALU |
| | | | | | | | | | | 8 | Null | ALU |
| | | | | | | | | | | 8 | Null | ALU |
| | | | | | | | | | | 8 | Null | ALU |
| | | | | | | | | | | 8 | Null | ALU |
| | | | | | | | | | | 8 | Null | ALU |
| | | | | | | | | | | 8 | Null | ALU |
| | | | | | | | | | | 8 | Null | ALU |
| | | | | | | | | | | 8 | Null | ALU |
| | | | | | | | | | | 8 | Null | ALU |
| | | | | | | | | | | 8 | Null | ALU |
| | | | | | | | | | | 8 | Null | ALU |
| | | | | | | | | | | 8 | Null | ALU |
| | | | | | | | | | | 8 | Null | ALU |
