# Supplementary material for: Artificial Selection on Microbiomes To Breed Microbiomes That Confer Salt Tolerance to Plants
Source: mSystems. 2021 Nov 30;6(6):e01125-21. doi: 10.1128/mSystems.01125-21 (PMC8631316; doi:10.1128/mSystems.01125-21)
Supplement: TEXT S2 [file msystems.01125-21-t0002.pdf]

SUPPLEMENTAL MATERIAL: STATISTICAL ANALYSES

Statistical Analyses: Plant Biomass, Generations 1-8: We performed all analyses in R v3.3.1. We assessed differences in above-ground plant biomass (dry weight) among treatments of Generations 1-8 by fitting the data to a generalized linear mixed model with a gamma error distribution. Line was entered as a random effect; generation, treatment, and their interaction were entered as fixed effects. Statistical significance of fixed effects in the GLMMs were assessed with likelihood ratio tests and Tukey tests employed for post-host comparisons of treatment means. Selection of the appropriate error distribution for the GLMMs was evaluated by visual inspection of Q-Q plots, and homoscedasticity was assessed using plots of the residuals of the model against the fitted values. Because plants were short-cycled in Generations 1-8 (i.e., grown long enough so plants produce typically 9-15 leaves, too short to develop flowers), plants did not produce any seeds, and therefore only above-ground plant biomass (dry weight) could be compared between treatments of Generations 1-8.

Statistical Analyses: Total Seed Weight, Generation 9: Because plants were grown long enough to flower in Generation 9, we compared total seed weight per plant among microbiome-selection treatments (plant present; microbiomes were differentially transplanted from plants of Generation 8 to seeds of Generation 9), Fallow-Soil Control (no plant present; microbiomes were harvested from fallow soil of

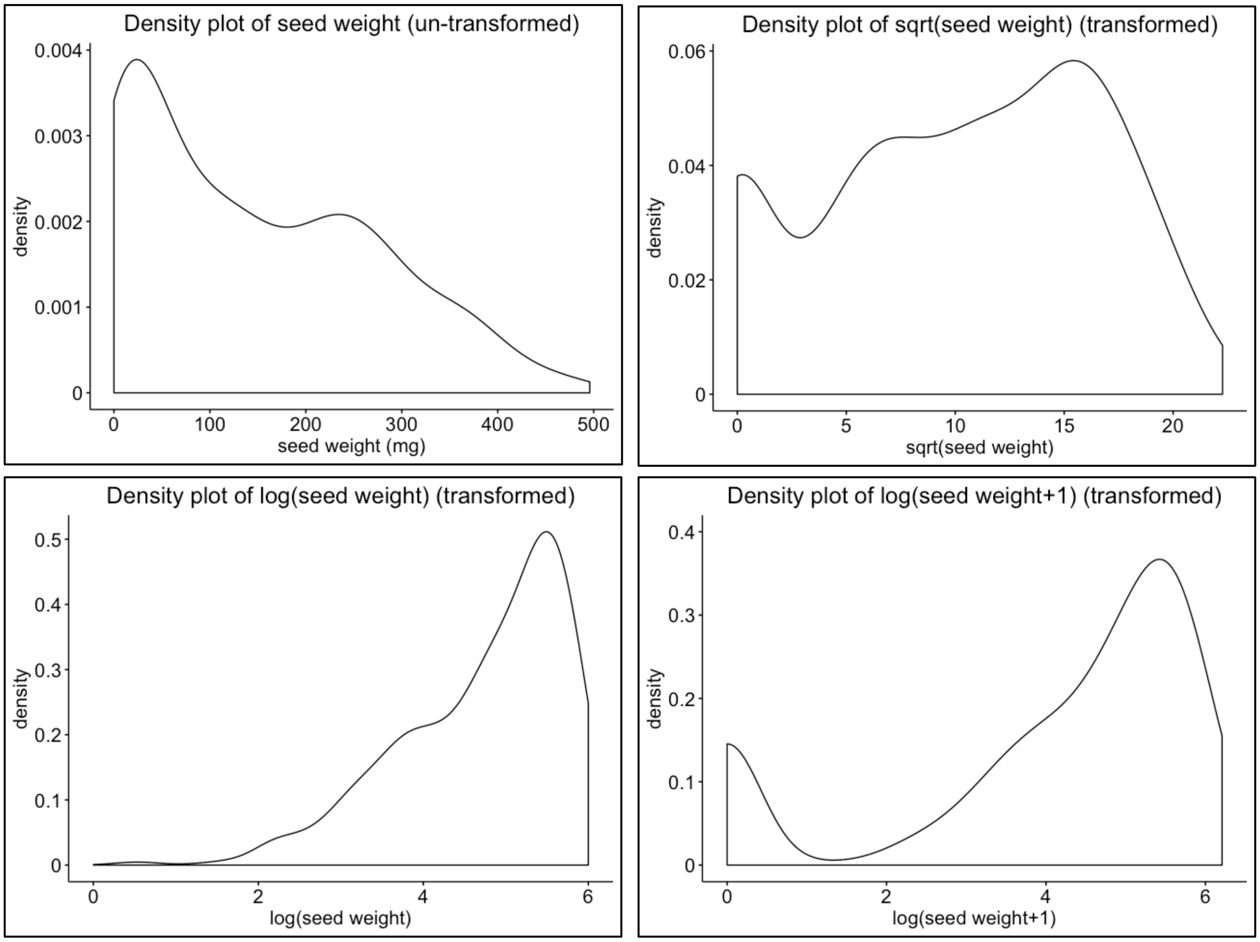

Figure S5. Top-left: untransformed seed-weight data in milligram (mg), indicating a skewed distribution, with many plants producing no or few seeds because of the extreme salt stress during Generation 9. **Top-right:** square-root transformed seed-weight data. **Bottom-left:** log-transformed seed-weight data, excluding seed-weights of zero because log(0) is not defined. **Bottom-right:** log-transformed seed-weight+1 data. None of the three transformations generated a distribution that approximated normality, and we therefore used non-parametric tests to evaluate differences in seed production between treatments.

Generation 8 for transfer to seeds of Generation 9), and Null-Control (no initial microbiome inoculation, microbes establish in microbiomes when microbes "rain in" from the air). Because plants were strongly salt-stressed in Generation 9 and many plants therefore did not flower or only produced very few seeds, the distribution of data was not normal (Figure S5 top-left). We therefore attempted several data-transformations to achieve approximate normality, including *square-root(seed weight)* transformation (Figure S5 top-right), *log(seed weight)* transformation [excluding the plants that generated zero seeds because log(0) is undefined; Figure S5 bottom-left], and *log(seed weight +1)* transformation (making it possible to retain the plants that produced zero seeds, because seed-weight values of all plants was increased by 1mg; Figure S5 bottom-right). None of these transformations generated a distribution that approximated normality (Figures S5b-d), and we therefore used Kruskal-Wallis tests for non-parametric evaluation of differences between treatments in Generation 9; and we used Mann-Whitney U-tests for non-parametric post-hoc comparisons between treatment means, correcting p-values using the false discovery rate. All tests were two-tailed with alpha=0.05.
